# Impacts of medical and non-medical cannabis on the health of older adults: Findings from a scoping review of the literature

**DOI:** 10.1371/journal.pone.0281826

**Published:** 2023-02-17

**Authors:** Dianna Wolfe, Kim Corace, Claire Butler, Danielle Rice, Becky Skidmore, Yashila Patel, Premika Thayaparan, Alan Michaud, Candyce Hamel, Andra Smith, Gary Garber, Amy Porath, David Conn, Melanie Willows, Hanan Abramovici, Kednapa Thavorn, Salmaan Kanji, Brian Hutton

**Affiliations:** 1 Ottawa Hospital Research Institute, Ottawa, Canada; 2 Institute of Mental Health Research at The Royal, University of Ottawa, Ottawa, Canada; 3 Department of Family Medicine, Faculty of Medicine, University of Ottawa, Ottawa, Canada; 4 School of Psychology, Faculty of Social Sciences, University of Ottawa, Ottawa, Canada; 5 Department of Medicine, Faculty of Medicine, University of Ottawa, Ottawa, Canada; 6 School of Epidemiology and Public Health, University of Ottawa, Ottawa, Canada; 7 Canadian Center for Substance Use and Addiction, Ottawa, Canada; 8 Department of Psychiatry, University of Toronto, Toronto, Canada; 9 Baycrest Health Sciences, Toronto, Canada; 10 Health Canada, Office of Cannabis Science and Surveillance, Ottawa, Canada; University of Turin, ITALY

## Abstract

**Background:**

Cannabis legalization has enabled increased consumption in older adults. Age-related mental, physical, and physiological changes may lead to differences in effects of cannabis in older adults compared to younger individuals.

**Objective:**

To perform a scoping review to map the evidence regarding the health effects of cannabis use for medical and non-medical purposes in older adults.

**Methods:**

Electronic databases (MEDLINE, Embase, PsycINFO, Cochrane Library) were searched for systematic reviews (SRs), randomized controlled trials (RCTs) and non-randomized/observational studies (NRSs) assessing the health effects and associations of cannabis use (medical or non-medical) in adults ≥ 50 years of age. Included studies met age-related inclusion criteria or involved a priori identified health conditions common among older adults. Records were screened using a liberal accelerated approach and data charting was performed independently by two reviewers. Descriptive summaries, structured tables, effect direction plots and bubble plots were used to synthesize study findings.

**Findings:**

From 31,393 citations, 133 publications describing 134 unique studies (26 SRs, 36 RCTs, 72 NRSs) were included. Medical cannabis had inconsistent therapeutic effects in specific patient conditions (e.g., end-stage cancer, dementia), with a number of studies suggesting possible benefits while others found no benefit. For medical cannabis, harmful associations outnumbered beneficial, and RCTs reported more negative effects than NRSs. Cannabis use was associated with greater frequencies of depression, anxiety, cognitive impairment, substance use and problematic substance use, accidents/injuries, and acute healthcare use. Studies often were small, did not consistently assess harms, and did not adjust for confounding.

**Discussion:**

The effects of medical cannabis are inconsistent within specific patient conditions. For older adults, generally, the available evidence suggests cannabis use may be associated with greater frequencies of mental health issues, substance use, and acute healthcare use, and the benefit-to-risk ratio is unclear. Studies with a balanced assessment of benefits and harms may guide appropriate public health messaging to balance the marketing pressures of cannabis to older adults.

## Introduction

Legalization of cannabis has increased access for consumers in a growing number of countries, including Canada and the United States. In the first year following legalization in Canada, the proportion of older adults who reported using cannabis in the previous three months increased significantly over pre-legalisation estimates, from 10.0 to 11.9% in those 45 to 64 years of age, and from 4.1 to 5.9% in those 65 years and older [1]. The proportion of adults 65 years and older who reported daily use of cannabis also rose significantly, from 1.6 to 2.6%, the greatest increase in any age group [1]. More than a half of those over 65 years of age reported using cannabis strictly for medical reasons, while a quarter of those over 65 years of age reported trying cannabis for the first time in the previous three months [2]. Prior to legalization (2008) in the US, 95% of adults over 50 that had consumed cannabis in the past year had initiated use before the age of 30, suggesting that a substantial number may have used cannabis over the long term rather than initiating at middle age for medical purposes [3]. More recent US surveys (2015–17) support this finding: more than 75% of adults over 50 who consumed cannabis in the past year had used continuously for at least three years, while less than 6% had initiated use for the first time in the past year [4]. Recent data suggest 6–7% of older adults use cannabis, and amongst them approximately 75% use it for medical purposes [5]. Older adults generally suffer from more chronic health conditions than younger adults (e.g., chronic pain, insomnia) [6, 7] and, thus, may be attracted to cannabis for medical purposes [3, 8] by marketing efforts that exaggerate the medical and “wellness” benefits of cannabis, while minimizing its harms [9], and by word-of-mouth anecdotal “evidence.” Legalization, increased access, and non-evidence-based marketing may plausibly increase the proportion of older adults who consume cannabis for both medical and non-medical purposes [10]. Past work has suggested medical cannabinoids may offer certain benefits from consumption when conventional treatment does not help for conditions that include neuropathic pain, chemotherapy induced nausea and vomiting, spasticity (from multiple sclerosis and spinal cord injury), palliative and end-of-life pain [11]. However, negative outcomes may also become increasingly prevalent, including increased risks of cannabis use disorder and problematic cannabis use, cognitive impairments, drug interactions due to polypharmacy, adverse reactions due to multiple health conditions, and injuries and visits to emergency departments (EDs).

Several age-related mental, physical, physiological, and pharmacokinetic changes may contribute to critical differences in the health effects of cannabis between older and younger adults. Declines in cognitive functioning, including executive function, memory, and attention, as well as structural changes to both the grey and white matter of the brain occur with age. Cannabis use has been associated with worsening of these outcomes [12–15]. Polypharmacy is highly prevalent in older adults and may increase susceptibility to drug interactions with cannabis [16–19], while the physiologic changes of aging may alter cannabis pharmacokinetics and pharmacodynamics [8, 20, 21], potentially further increasing the risk of adverse drug effects and interactions.

Currently, Canadian guidelines support the use of cannabis for a limited number of conditions in all age ranges, when standard treatments are ineffective (e.g., neuropathic and palliative pain, chemotherapy-induced nausea and vomiting, spasticity in multiple sclerosis and spinal cord injury) [11]. A greater understanding of the beneficial and harmful health effects of cannabis for both medical and non-medical consumption in older adults is needed, as well as targeted dissemination of evidence-based education and information to physicians, health care providers or practitioners, public health organizations, and the general public, including older adults and their caregivers and family members. We carried out a scoping review of the literature to evaluate the depth of the available evidence regarding impacts and associations of use of cannabis for medical and non-medical purposes on the health of older adults, with the intent of mapping the evidence and identifying priority areas for future research.

## Review methods

A scoping review protocol was developed a priori, guided by established scoping review methodology [22–25], and was registered with the Open Science Framework (doi 10.17605/OSF.IO/5JTAQ), and published [26]. Protocol amendments are described along with rationale in **S1 Text**.

### Key questions

The following review question was addressed:

What evidence exists regarding the beneficial and harmful effects of cannabis for medical and non-medical purposes in older adults?

Findings within different categories of the following subgroups, concepts, and contexts were of interest: older adult age categories (e.g., 50–64 years, 65+ years); sex or gender; race or ethnicity; mental or physical comorbidities; frailty; co-use of prescription or non-prescription drugs, alcohol, or illicit substances; consumption method (e.g., smoking, vaporizing, oils, edibles); residential setting (e.g., community, long-term care, hospital); employment status; marital status; accommodation status (e.g., alone, shared, shelter).

### Study eligibility criteria

**Table 1** provides a summary of study eligibility criteria.

**Table 1:** Study eligibility criteria.

PCC framework domain	Element	Criteria details
Participants	Age	≥ 80% of the study sample aged 50 years and older. Formulae were used to determine age eligibility when studies included adults of all ages (see the “Additional Details of Scoping Review Methods” section below for details).
		Disease conditions that affect mainly older adults were used as proxies for age in cases where the age of participants was not explicitly reported: end-stage cancer, Alzheimer’s disease or dementia, and Parkinson’s disease.
		Chemotherapy for any indication was not considered a proxy for age because patients did not necessarily have end-stage cancer.
	“Current” cannabis use	As defined by the study, but not more than one year in the past.
		Evaluations of the effects of age of initiation of cannabis use, lifetime/ever cannabis use, or previous cannabis use, where the older adult was no longer using cannabis, were excluded.
	Other characteristics	Individuals of any sex/gender or race were of interest. Healthy individuals as well as those with physical or mental health conditions, whether acute or chronic, were of interest.
Concept	Interventions	Cannabis must have been the intervention or exposure in the study.
		**Types of use:** medical purposes (overseen by a physician or other health provider or self-medicated; hereafter “*medical cannabis*”) or non-medical purposes (hereafter “*non-medical cannabis*”), of any type, with any mode of consumption (e.g., pills/capsules, smoking, vaporizing, oils, edibles).
		**Cannabis types:** whole-plant/loose-leaf cannabis; purified whole-plant extracts (e.g., Nabidiolex^®^ (purified cannabidiol (CBD)), Tetrabinex^®^ (purified delta-9-tetrahydrocannabinol (THC)), Sativex^®^ (purified 1:1 THC:CBD)); cannabinoid derivatives, developed through modification of molecular structure (e.g., nabilone); and other cannabinoids (e.g., dronabinol), whether found in the cannabis plant or elsewhere and that interact with the endocannabinoid system [27].
		**Comparisons:** use vs no use (or placebo), types of use, types of cannabis, modes of consumption, doses, etc.
		Analyses comparing age categories amongst those who use cannabis were not of interest because cannabis was not the exposure of interest.
	Outcomes	Any physical health, mental health, physical brain structure, pharmacokinetic, and global quality of life outcomes, well as measures related to the use or problematic use of other drugs and alcohol. Examples are provided in the “Additional Details of Scoping Review Methods” section below. We excluded single-arm studies that only reported prevalence or incidence of cannabis use in older adults and those assessing cannabis use as an outcome (however, cannabis use disorder (CUD) as a mental health outcome was eligible).
Context	Setting, geography, time period, follow-up duration	Current cannabis consumption in all settings, in any geographic area, and including all periods of time and durations of follow-up. Consumption of other illicit drugs or substances, or prescribed pharmaceuticals was allowed.
	Study design	Systematic reviews (including overviews of reviews), randomized controlled trials (RCT), non-randomized/observational studies (NRS) (*note that observational studies are included in the NRS acronym*; additional design-specific criteria are provided in the “Additional Details of Scoping Review Methods” section below). Regarding studies included in systematic reviews, we included those that met our a priori eligibility criteria (if not already captured by our search).
		We excluded qualitative studies, diagnostic test accuracy studies, studies developing or validating diagnostic criteria for CUD or other cannabis-related mental health disorders, editorials, letters, commentaries, abstracts, case reports, case series under 25 patients, and narrative reviews.
	Language	Only English and French publications were considered for reasons of timeliness and cost.

### Description of methods

**Table 2** provides a brief description of the methods, with complete methods described in **S2 Text**. Of note, where possible, we have summarized findings according to their potential to reflect causal effects. Therefore, findings from RCTs and cohort studies for which causation may be inferred have been differentiated from associations reported in cross-sectional and case-control studies.

**Table 2:** Methods in brief.

Review stage	Details
Literature search	Ovid MEDLINE^®^, including Epub Ahead of Print and In-Process & Other Non-Indexed Citations, Embase Classic+Embase, and PsycINFO, using the Ovid Platform, and the Cochrane Library on Wiley
	Filters applied for study designs of interest
	No language or date restrictions
	Search strategies in **S3 Text**
	Peer reviewed using Peer Review of Electronic Search Strategies (PRESS) Checklist [28]
	Searches run 14 June 2019 and updated to 30 November 2020
	Bibliographies of included systematic reviews screened
	For feasibility, no grey literature searching was performed
Study selection	Citations collated and duplicates removed in EndNote^a^, with unique records uploaded to online systematic review management software (DistillerSR^®^^b^)
	Two levels of citation screening: (1) title/abstract and (2) full text. Pilot screening at each level.
	Liberal accelerated approach for Level 1, using DistillerSR’s^®^ artificial intelligence (AI) active machine learning to prioritize references
	Dual independent screening for Level 2, with conflict resolution through discussion
	Once an estimated recall of 95% of included studies was achieved, the AI reviewer was assigned to exclude the remaining citations
	A human reviewer screened all citations excluded by the AI reviewer, with conflicts resolved by a second human reviewer
Data charting	Data charting in DistillerSR^®^, using standardized piloted forms.
	Dual independent data extraction, with conflict resolution through discussion
	Study characteristics, demographic data, and outcome data extracted
	Results data limited to direction of effect, statistical significance, and type of analysis (uni- or multivariable). Raw data without an analysis were not extracted.
	Direction of effect: based upon the effect estimate relative to the null value (e.g., for harmful outcomes, any value < 1 was “beneficial” and any value > 1 was “harmful”)
	Statistical significance: as reported in the study, whether through confidence interval, standard error, p-value, or a statement of significance
Risk of bias	Not performed for primary studies, as per scoping review methodology
	Quality of systematic reviews appraised using the AMSTAR-2 tool [29] by two reviewers, independently, with consensus through discussion
Data synthesis/ mapping/ reporting	Descriptive statistics of the available data and narrative summaries of results, complimented by tables and figures
	Separate syntheses for each patient condition
	Study design (RCT, cohort, case-control, cross-sectional) and analysis type (adjusted or unadjusted) in NRSs considered in narrative summaries to reflect level of evidence and potential for causal effects. The words “effects” or “impacts” were used to imply potential causation in RCTs and cohort studies, while “associations” and “correlations” were used to imply non-causal relationships between exposures and outcomes in cross-sectional and case-control studies.
	Bubble plots and direction of effect figures used to visualize evidence
	Use of “people-first” language to communicate results in a non-stigmatizing way [30]
	Reported was structured according to the Preferred Reporting Items for Systematic Reviews and Meta-analysis extension for scoping reviews (PRISMA-ScR) statement [31] (**S4 Text**).

^a^ Thomson Reuters. EndNote X7.

^b^ *DistillerSR*. Version 2.35. Evidence Partners; 2021. Accessed May 2019 to November 2021. https://www.evidencepartners.com.

## Results

### Extent of literature identified

**Fig 1:**
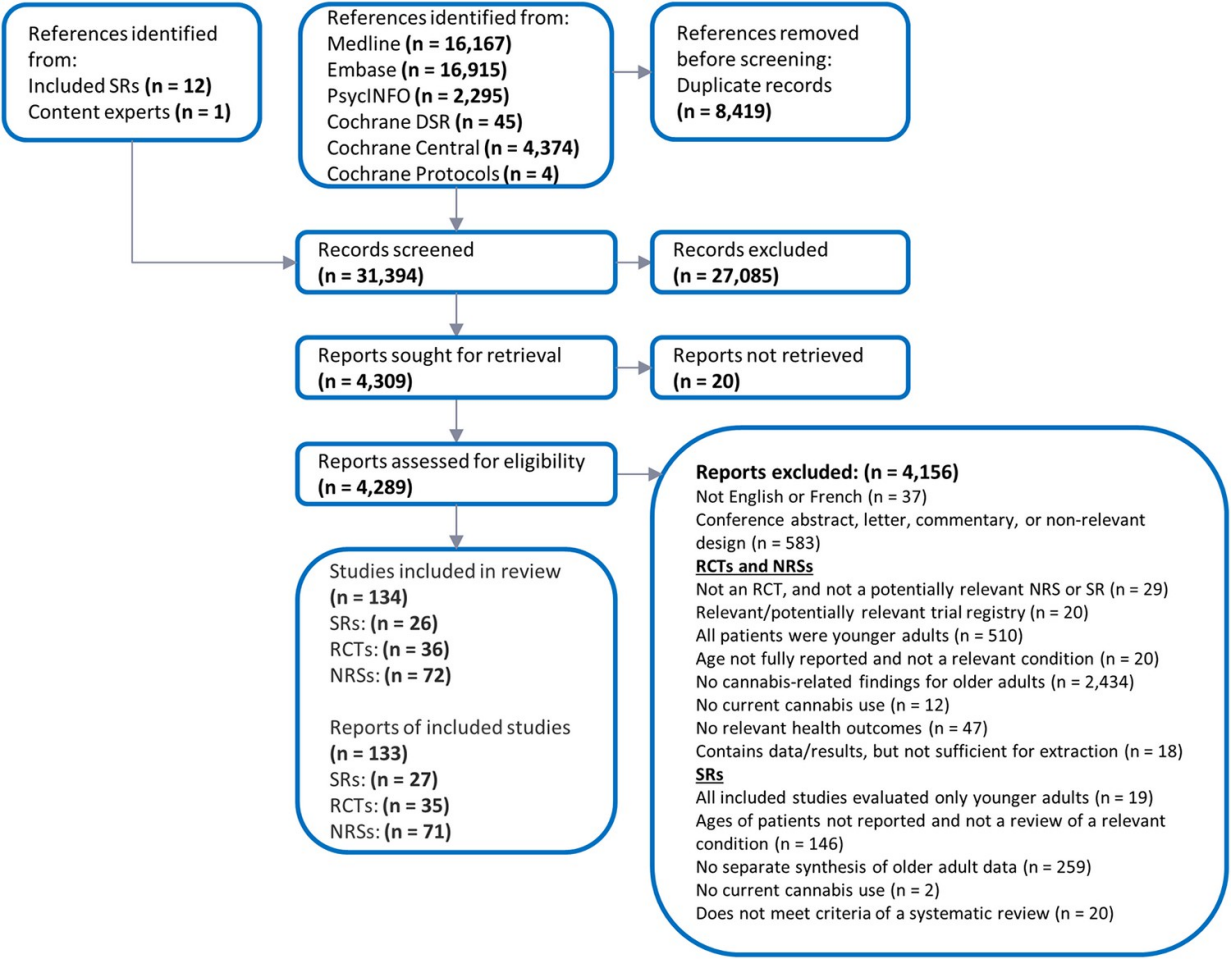
Overview of publication selection process.

**Fig 2:**
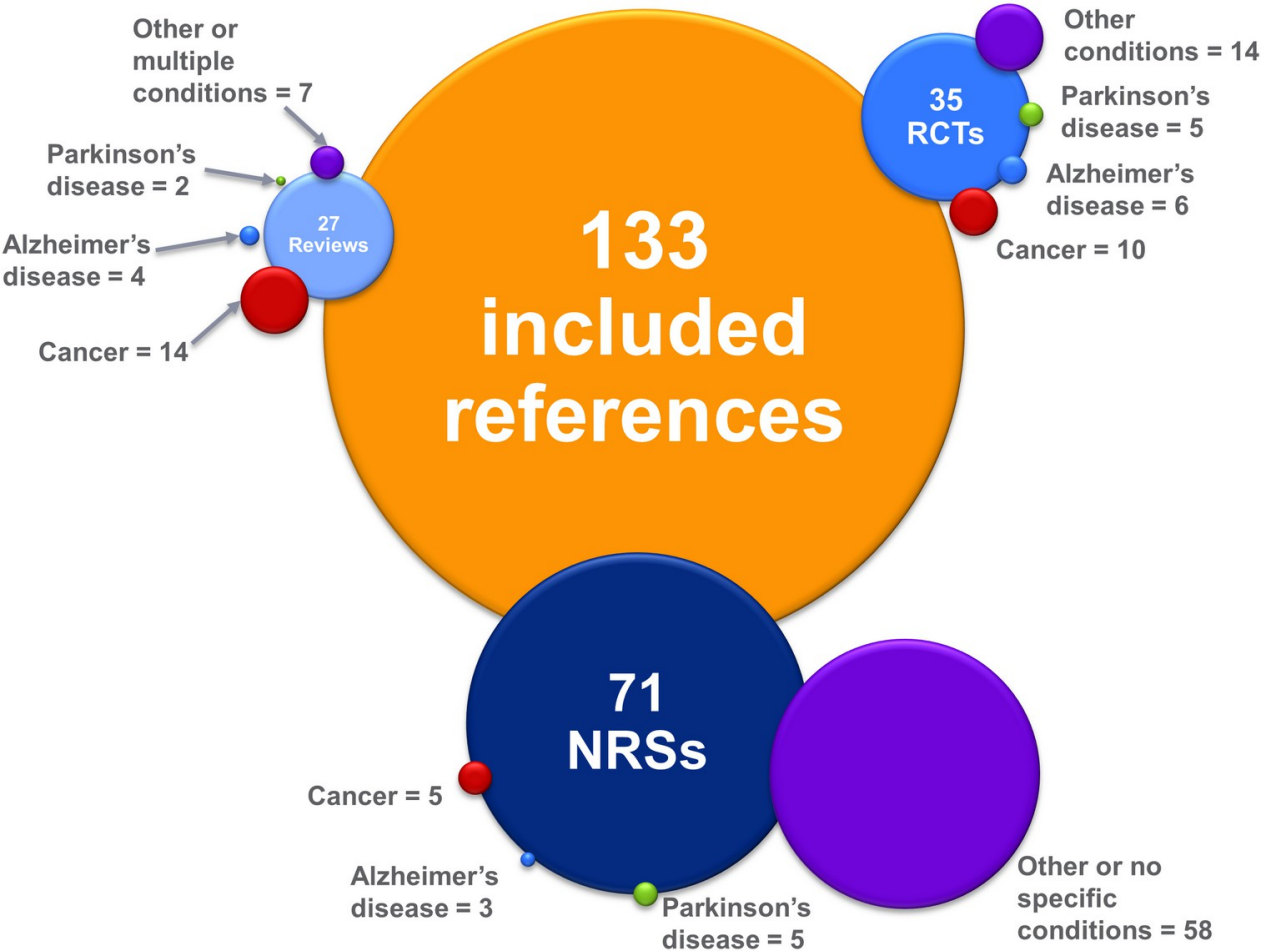
Visual representation of included evidence by study design and patient condition. An overview of the included number of publications is shown; some publications included more than one study. The size of each bubble is relative to the number of publications it represents.

Amongst more than 31,000 citations screened, 133 publications were included: 4 overviews of reviews reported in 5 publications [32–36]; 22 systematic reviews [37–58]; 36 RCTs reported in 35 publications [20, 59–92]; and 72 NRSs reported in 71 publications [4, 93–162]. **Fig 1** summarizes the study selection process, while **Fig 2** provides a visual representation of the evidence base; the listing of citations excluded during full text screening is provided in **S5 Text**. Detailed evidence tables presenting key information regarding each study have been provided in **S6 Text** due to the considerable length of these data.

Three of four overviews of reviews focused on cancer [32–35], while one focused on the use of medical cannabis in any condition, including cancer, Alzheimer’s disease, and Parkinson’s disease [36]. None of the overviews focused specifically on cannabis use in older adults (i.e., they focused on patients of all ages, but provided at least one synthesis for older adults or a relevant condition), and none included the most recent systematic reviews. Thus, no further synthesis of these publications was undertaken.

All but one of the systematic reviews focused on the efficacy and safety of cannabis in the treatment of one or more medical conditions [37–43, 45–58]; the other evaluated associations between cannabis use in older adults and the incidence of cancer [44]. All but one review rated low or critically low on AMSTAR-2 assessment; the final review rated moderate [57]. Twenty-two of the 105 included primary study publications (21%) were synthesized in at least one of the included systematic reviews, including 17 of 35 included RCTs [60, 62–66, 70–72, 74, 76, 80, 82, 83, 86, 87, 89] (49%) and 5 of 70 included NRSs (7%) [101, 137, 148, 156, 158]. Given the low coverage of primary studies of interest to our scoping review in the included systematic reviews and the relatively low quality of the included reviews, we have prioritized reporting evidence from primary studies over that from systematic reviews in the following sections.

### Interventions and exposures evaluated in the included primary studies

Cannabis interventions were generally well reported in RCTs. Sativex^®^ (n = 8) [62, 67, 70, 76, 78, 82, 80], nabilone (n = 7) [61, 68, 72, 77, 79, 83, 85], dronabinol (Marinol^®^) (n = 5) [63, 74, 75, 89, 92], and Namisol^®^ (n = 5) [20, 60, 86–88] were the primary cannabis products studied, with two studies evaluating unnamed synthetic CBD products [69, 91]. Eight RCTs studied other natural whole plant or extracts [59, 64–66, 71, 73, 84, 90], and one studied an unknown medical-grade CBD powder [66]. Most RCTs evaluated medical cannabis use, overseen by a physician (n = 27); the remaining six studies conducted experiments in laboratory settings, evaluating the immediate effects of cannabis [20, 59, 69, 80, 83, 90]. Nineteen RCTs used a parallel-group design [61–64, 66, 68, 70, 71, 73–76, 78, 79, 81, 82, 85, 86] and 17 used a cross-over design [20, 59, 60, 65, 67, 69, 72, 77, 80, 83, 84, 87–92].

Cannabis interventions were poorly reported in NRSs, with 46 of 72 studies (64%) not reporting a specific cannabis intervention. Many of these were large population-based cross-sectional studies, using data from national surveys (e.g., National Surveys on Drug Use and Health (NSDUH)). Four other NRSs studied dronabinol [120, 121, 156, 158], three studied nabilone [101, 133, 155], and seventeen studied natural whole plant or extracts [93, 94, 97–99, 102, 119, 129, 131, 137, 147–151, 154, 161]. Many NRSs (n = 28) did not explicitly report the type of cannabis use of interest (i.e., medical or non-medical) [95, 96, 102, 103, 105, 107, 113, 115, 117–119, 122–124, 126–128, 134, 139, 141–143, 145, 146, 157, 159–161]. Fourteen other studies explicitly stated that all cannabis use, whether for medical or non-medical purposes, was evaluated [4, 97, 100, 104, 106, 108–110, 112, 116, 132, 135, 138, 144]. Fifteen NRSs focused strictly on medical use, overseen by a physician [94, 98, 99, 101, 111, 120, 121, 131, 133, 136, 148, 149, 152, 156, 158]; four focused strictly on non-prescribed medical use (i.e., taken for a perceived or actual medical condition, not for recreational purposes) [150, 151, 154, 155]; and ten focused strictly on non-medical/recreational use [93, 114, 125, 129, 130, 137, 147, 153, 162]. The remaining NRS studied medical use of any kind [140].

### Clinical findings by population

**Fig 3:**
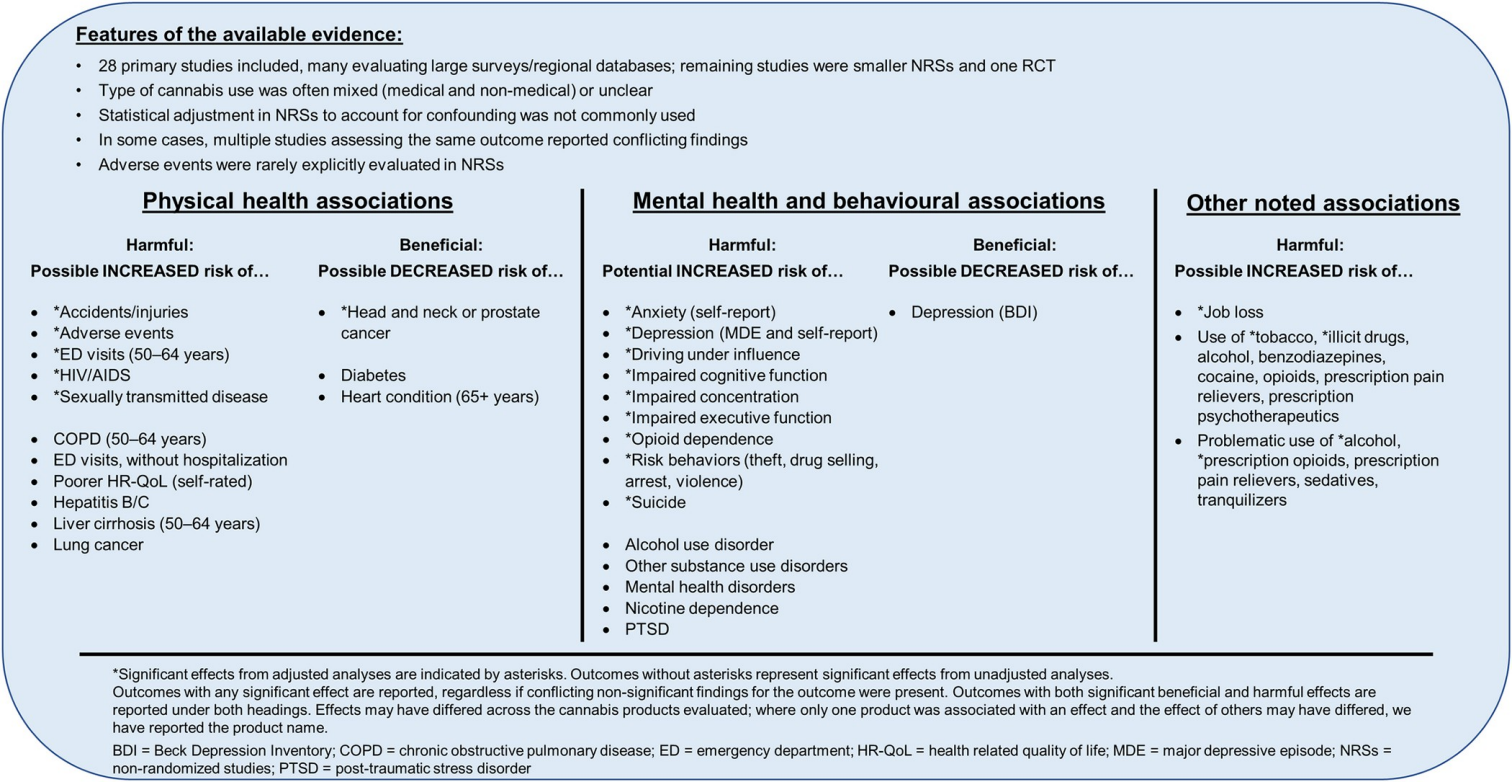
Synopsis of findings reported in population-based studies and studies of healthy older adults.

**Fig 4:**
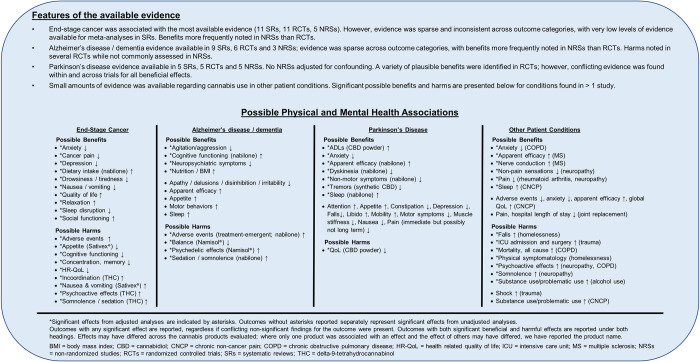
Synopsis of findings reported in studies of cannabis use in unique patient conditions and populations.

In the following sections, we provide detailed descriptions of the included evidence regarding cannabis use in older adults, organized according to the selection criteria of the studies. Studies that selected participants based on specific clinical indications (i.e., end-stage cancer, Alzheimer’s disease/dementia, Parkinson’s disease, chronic non-cancer pain, multiple sclerosis, etc.) have been summarized under corresponding headings, while studies that did not select participants based on a clinical indication or that selected only healthy participants have been summarized under the heading “Population-based studies and studies of healthy older adults.” We provide summaries of key findings associated with healthy older adults and the older general public in **Fig 3**, while **Fig 4** provides a synopsis of findings pertaining to unique clinical conditions and populations. In the main text that follows, where possible, we have prioritized findings from studies from which potential causal inferences could be drawn over findings from other designs (i.e., RCTs and cohort studies have been summarized before findings from cross-sectional and case-control studies), in particular within the summary of population-based studies and studies of healthy older adults, where most of the identified evidence was derived from cross-sectional and case-control designs. Additionally, we have prioritized reporting of findings from RCTs and NRSs that adjusted for confounding to focus on the least biased results. To ensure complete mapping of all available evidence, results from unadjusted analyses have also been included; however, they have been summarized more succinctly. As noted above, we were unable to fully separate the reporting of findings for medical versus non-medical cannabis use as many studies did not clearly report the intent of use or involved a mixture of both usage types; findings are thus reported according to population condition to provide an organized synthesis of the literature that will be intuitive to readers. Medical cannabis use can be assumed for sections reporting findings for specific patient medical conditions; for all other sections, we have noted the cannabis use type (medical, non-medical, or mixed), where this information was available.

### Population-based studies and studies of healthy older adults

Studies included in this section either sampled only healthy older adults [20, 153, 162] or did not sample participants based on a specific health condition [4, 20, 44, 95, 96, 100, 102–107, 109, 113, 115, 124, 128, 129, 132, 134, 135, 139, 143, 144, 146, 147]. A quarter of all primary studies contributed evidence in this section (n = 28). More than half of these studies evaluated data from large population-based surveys or national/regional databases (n = 15), either as cross-sectional studies at a single point in time [95, 100, 105, 107] or sequential designs that analyzed survey data over multiple years [4, 102, 104, 106, 116, 124, 128, 134, 139, 144, 146]. One systematic review reported on the association between cannabis use in older adults and the incidence of cancer [44]; no systematic reviews explored associations of cannabis use with other outcomes in healthy older adults or the older general public. Generally, cannabis use type could not easily be determined, with 10 primary studies including a mix of medical and non-medical use, and another 11 studies reporting no data related to type of use (**Table 3**). The majority of primary studies (n = 21) reported non-industry funding. Detailed effect direction plots of evidence from all study designs are provided in **S7 Text**.

**Table 3:** Characteristics of population-based studies and studies of healthy older adults.

Number of studies	Designs	Primary studies (n = 28)	
		Type of cannabis use	Funding
29	Systematic review: 1 [44]	Non-medical: 5 [129, 147, 153, 162]	Non-industry: 21 [20, 95, 102, 103, 106, 109, 113, 116, 124, 128, 129, 132, 134, 135, 139, 143, 146, 147, 153, 162]
	RCTs: 1 [20]	Mixed use: 10 [4, 100, 104, 106, 107, 109, 116, 132, 135, 144]	Mixed (industry and non-industry): 1 [96]
	Prospective cohort: 1 [96]	Immediate effects in lab: 1 [20]	Not funded: 2 [104, 107]
	Retrospective cohort: 2 [103, 135]	Not reported: 11 [95, 102, 103, 105, 113, 124, 128, 134, 139, 143, 146]	Not reported: 4 [4, 100, 105, 144]
	Case-control: 2 [129, 147]		
	Sequential: 11 [4, 102, 104, 105, 116, 124, 128, 134, 139, 144, 146]		
	Cross-sectional: 11 [95, 100, 106, 107, 109, 113, 132, 143, 153, 162]		

A small proportion of primary studies used a RCT or cohort design from which potential causal inferences could be made (n = 4; 14%). Interpretations derived from the cross-sectional, sequential survey, and case-control designs were restricted to associations between cannabis use and the outcomes assessed and have been reported following the RCT and cohort study findings. Additionally, statistical adjustment for confounding in all NRSs was uncommon, increasing the risk of confounding bias. Consequently, the focus of this summary is on findings from the RCT and cohort studies that controlled confounding. Adjusted findings from cross-sectional, sequential survey, and case-control studies have been summarized separately, while salient unadjusted findings from all designs have been summarized more briefly.

#### RCTs and cohort studies

**Fig 5:**
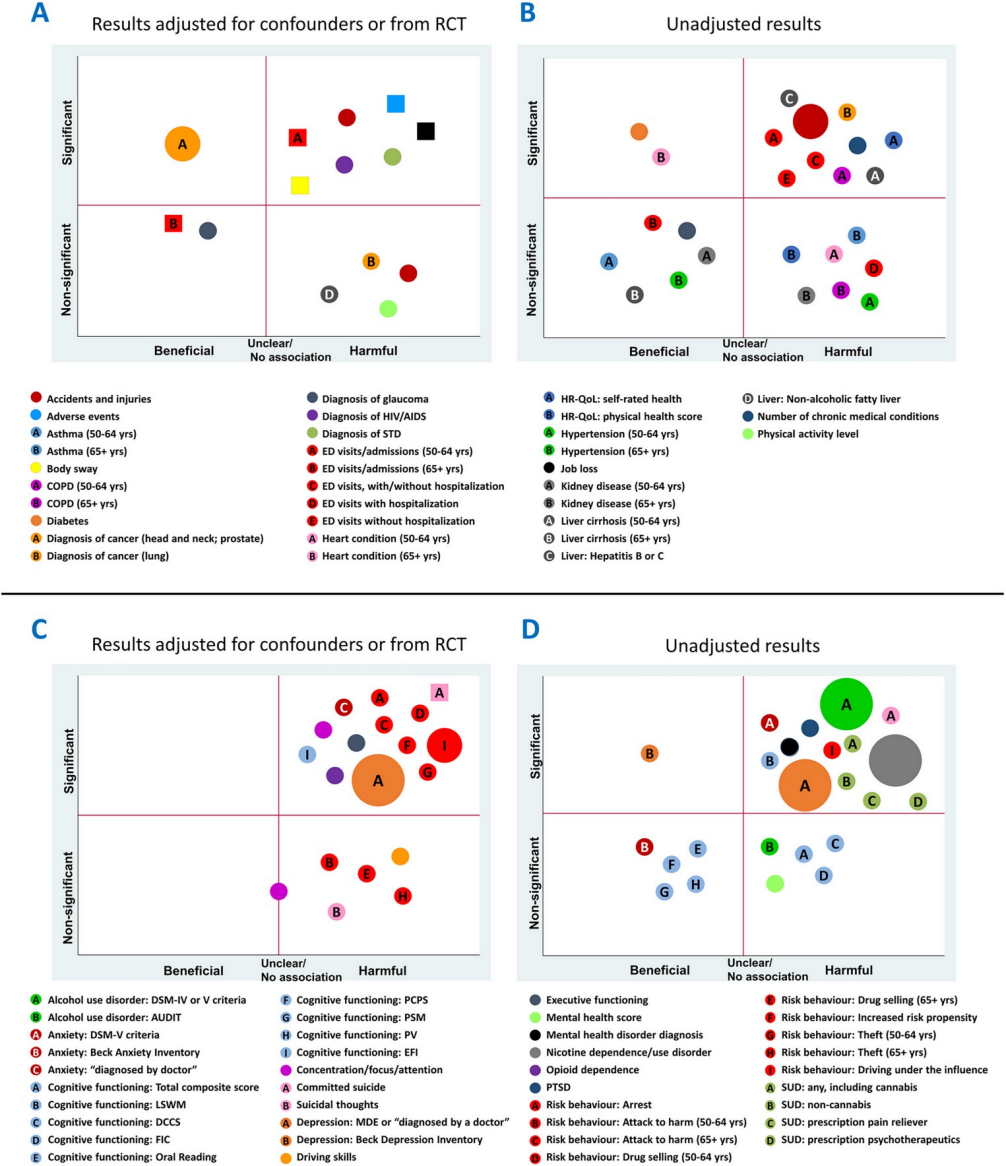
Bubble plots of findings from population-based studies and studies of healthy older adults. **Findings at a glance:** Observed associations of cannabis use with physical health outcomes (Panels A, B) and mental health outcomes (Panels C, D), with separate plots for adjusted analyses/RCTs and unadjusted analyses. Some associations were derived from studies with cross-sectional and case-control designs (circles), while others were derived from cohort studies and RCT (squares). Quadrants of plots represent significant and non-significant beneficial and harmful effects. Each bubble represents an outcome definition for which at least one study reported an effect within a plot quadrant. Bubble size represents the number of studies that reported that outcome definition in the quadrant. Letters within bubbles denote subgroups or different outcome definitions within an outcome group.

Four primary studies used either a RCT or cohort design in which potential causal effects could be assessed [20, 96, 103, 135]. Harmful effects of cannabis on health outcomes reported in these studies outnumbered beneficial effects (**Fig 5, square symbols**). A retrospective cohort study found significant adjusted associations between mixed cannabis use and suicide in male Veterans Health Administration (VHA) patients [103]; in female patients, the effect of mixed cannabis use on the risk of suicide became non-significant when adjusted for psychiatric illness [103]. Concerns regarding an increased potential for falls and injuries in older adult consumers led to a small RCT (n = 12) evaluating the immediate effects of Namisol^®^ (synthetic THC) in the laboratory. Namisol^®^ was found to cause a statistically significant but not clinically significant increase in body sway compared to both baseline non-use and placebo, and its use was also associated with significantly greater numbers of adverse events (AEs) both compared to placebo and in a dose-dependent manner [20]. A retrospective cohort study found in adjusted analyses that 50–64-year-olds who used cannabis daily had a significantly increased hazard of acute care use compared to no cannabis use; however, less frequent use did not significantly increase the hazard of acute care use. No significant effects of cannabis use compared to no use on acute care use were found in those 65+ years of age [135]. In a large prospective cohort study in France, the risk of job loss was significantly higher in adults who used cannabis than in those who did not in an adjusted analysis [96]. This relationship was reported to be dose dependent. The intent of cannabis use (medical versus non-medical) of interest in the study was not reported, and was described in terms of categories of exposure (never used, no consumption in prior 12 months, use less than once a month, use once a month or more); given the study also examined associations with alcohol and tobacco, there is reason to hypothesize the authors’ interests related to non-medical cannabis use [96].

The pharmacokinetics of oral THC have been evaluated in a lab-based RCT of older adults who smoked fewer than one cannabis cigarette per week [20]. Substantial inter-individual variation in plasma concentrations of THC and its metabolites was identified, a finding that is in line with previous studies of participants of various ages [20]. For some older adults, the time (t_max_) when the maximum plasma concentration (C_max_) of THC was reached was delayed beyond 120 minutes (whereas t_max_ in younger adults in other studies has been reported as 60 minutes or less [163]). However, the value of C_max_ was similar to that published for younger adults [163], if reached within 120 minutes [20]. The pharmacodynamic effects of THC first occurred within 20 minutes of dosing, with maximal effects occurring between 55 and 120 minutes [20]. As THC dose increased, there were significant increases in body sway; however, the magnitude of these effects was low and unlikely to increase the risk of falls [20]. No pharmacokinetic studies were identified that evaluated drug interactions with cannabis in this population.

#### Cross-sectional and case-control studies

Cross-sectional, sequential survey, and case-control studies assessed associations of cannabis use with the presence of numerous physical and mental health outcomes (**Fig 5A–5D, round symbols**). Results reported from adjusted analyses **(Fig 5A and 5C, round symbols)** have been summarized below.

Accidents and injuries were found to be significantly positively associated with cannabis use in adjusted analyses, with outcome definitions varying substantially from study to study. Baseline data from a prospective cohort study of participants 65–79 years of age found a statistically significant adjusted association between cannabis use and past-year car accidents and citations [113], while a larger sequential survey of adults ≥ 55 years of age found no significant adjusted association between past-year use and past-year vehicle collisions [134]; neither study reported type of cannabis use. Another large cross-sectional study of mixed (medical and non-medical) cannabis use found no significant adjusted associations between either dose (the number of joints smoked in a day) or frequency of use and either (1) past-year emergency department (ED) visits or (2) past-year injuries that caused a person to seek medical help or cut down on usual activities for more than half a day [107].

Regarding other physical health conditions reported from adjusted analyses in cross-sectional designs, cannabis use (characterized as any past-year use of marijuana or hashish) was found to be significantly positively associated with past-year diagnoses of HIV/AIDS in those 50–64 years of age (but not those ≥ 65 years of age) and diagnoses of sexually transmitted diseases in all ages of older adults [146]. Non-alcoholic fatty liver disease was also positively associated with cannabis use (including current use of Indian hemp, marijuana and other varieties of cannabis and cannabinoids) in individuals with and without cannabis dependence, respectively, compared to those who do not use cannabis, although the observed association did not reach statistical significance [95]. A non-significant negative association of cannabis use with glaucoma was reported in a sequential survey (i.e., fewer people with glaucoma reported using cannabis five or more times a month than people without glaucoma) [124].

Associations of cannabis use with cancer were assessed in two case-control [129, 147] and one cross-sectional [143] studies. Cannabis use was reported to have a significant negative association with two forms of cancer (i.e., cannabis use was lower in those diagnosed with cancer): head and neck squamous cell carcinoma (non-medical use; case-control study) [129] and prostate cancer in older African American men, diagnosed with prostate-specific antigen levels (use type not reported; cross-sectional study; n = 644) [143]. A second case-control study found non-medical use of hashish/kiff trended toward a positive association with diagnoses of lung cancer (i.e., more incident cases of lung cancer), but the effect did not reach statistical significance [147]. When use of the tobacco product snuff was included with hashish and kiff, the positive association became statistically significant [147].

A variety of mental health and behavioral outcomes was assessed in the included cross-sectional and sequential survey studies in adjusted analyses (Fig 5C). In multiple studies, cannabis use was found to be significantly positively associated with (1) diagnoses of depression (one study of mixed use and two studies did not report type of use) [102, 106, 146] and (2) driving under the influence of alcohol or other substances [113, 146]. Significant adjusted positive associations were reported in single survey studies between mixed use of cannabis and impaired cognitive functioning (Executive Function Index [EFI] subscale) [100], executive functioning [100], and attention [100]; and cannabis use of an unreported type and diagnosis of anxiety [146]; increased risk propensity [146]; and illegal high-risk behaviours, such as drug selling, theft, arrest, and attacking others with intention to harm [146]. However, analyses stratified by age suggested that engagement in these illegal high-risk behaviours was only significantly positively associated with cannabis use in those 50–64 years of age, but not those ≥ 65 years [146]. In a survey study that did not report type of use, a non-significant positive association was found between cannabis use and past-year suicidal thoughts [105], and amongst those who used cannabis, a significant positive correlation was found between past-year suicidal thoughts and the frequency of cannabis use [105].

Physical health findings assessed in only unadjusted analyses in cross-sectional and case-control studies (**Fig 5B**) included significant positive associations with past-year injuries (mixed use) [107], ED visits (mixed use) [107, 116], self-rated health (health-related quality of life [HR-QoL]; mixed use) [107], liver cirrhosis (≥ 50 years) and hepatitis B or C (mixed use) [116], and chronic obstructive pulmonary disease (COPD; ≥ 50 years; mixed use) [116]. Mixed use of cannabis was also found to have significant negative unadjusted associations with “ever” diagnoses of diabetes (≥ 50 years) [116] and heart conditions (≥ 65 years) [116]. Mental health findings assessed with only unadjusted analyses (**Fig 5D**) included significant positive associations with diagnoses of mental health disorders (bipolar, manic, and hypomanic disorders) [107] and post-traumatic stress disorder (PTSD; mixed use) [107]; driving under the influence (DUI; mixed use) [4]; and impaired cognitive functioning (non-EFI measures; non-medical use) [153, 162]. Many metrics of cognitive function were measured; however, none were significantly associated with cannabis use once univariable analyses were subjected to false discovery rate (FDR) correction [153, 162]. Unadjusted analyses demonstrated non-significant associations between cannabis use and numerous other physical and mental health conditions, and we refer readers to **Fig 5B** and **S3 Dataset** for further details.

Comparisons of overall brain structure and cortical and sub-cortical grey matter measures between older adults who used non-medical cannabis and those who did not were reported in a small cross-sectional study [153, 162]. After adjustment for age and baseline depression symptoms, there were no differences in total cerebrospinal fluid (CSF), grey matter, or white matter volumes between the two groups; however, some regional volumes (i.e., left putamen, left and right palladium) were found to be significantly greater in those who used cannabis [162]. Only left putamen volume remained significantly different between the two groups, after FDR correction [162]. Preliminary findings also suggested that amongst those who used non-medical cannabis, dose (i.e., past-90-day estimated THC dose), frequency of use (i.e., number of days used in the past 90 days) and the duration of use (i.e., estimated years of use and short-term vs long-term) were significantly negatively associated with regional volumes [153] (see **S13 Text**).

Many cross-sectional and sequential survey studies provided evidence regarding associations of cannabis use with the use and problematic use of other substances, as well as other substance use disorders: (eight studies of mixed use [4, 104, 106, 109, 110, 116, 132, 144], one of non-medical use [162], and four studies did not report type of use [102, 107, 128, 146]). All reported adjusted associations with cannabis use were significant and positive, including associations with the use of tobacco (use type not reported) [146] and illicit non-cannabis substances (i.e., cocaine, hallucinogens, opioids, etc.; use type not reported) [146], binge alcohol use (use type not reported) [146], misuse of prescription pain relievers (use type not reported) [102] and prescription opioids (mixed use) [144], and opioid dependence (mixed use) [144]. Where reported, age stratified analyses demonstrated similar findings across older adult age categories (use type not reported) [146]. Amongst older adults with past-year mixed cannabis use, both dose (number of joints) and frequency of use (> once a month vs ≤ once a month use) were significantly associated with lifetime occurrence of any substance use disorder in adjusted analyses [106]. As well, compared to medical use, non-medical use was positively associated with either cannabis abuse or dependence; however, the association was not statistically significant [4].

In unadjusted analyses, significant positive associations were found between cannabis use and the use of several other substances, including alcohol (mixed use) [104], cocaine (mixed use) [116], prescription opioids (use type not reported) [128], any prescription pain reliever (mixed use) [104], non-cannabis prescription psychotherapeutics (e.g., tranquilizers, stimulants, sedatives; mixed use) [104], and prescription benzodiazepines (mixed use) [132]. Significant unadjusted positive associations were also identified with the misuse of the following prescription drugs: any prescription drug (mixed use) [109], sedatives (mixed use) [116], tranquilizers (mixed use) [116], and non-cannabis prescription psychotherapeutics (mixed use) [104]. Significant unadjusted positive associations were also found with the following use disorders and dependences: any substance use disorder (including CUD; mixed use) [104], alcohol use disorder (DSM-IV and -5 criteria; mixed use) [104, 107, 116], nicotine dependence (mixed use) [104], non-cannabis illicit drug use disorder (mixed use) [104, 107], prescription pain reliever use disorder (mixed use) [104], and non-cannabis prescription psychotherapeutic use disorder (mixed use) [104]. Amongst older adults who used cannabis, in unadjusted analyses, frequency of use (mixed use) in the past year (100–365 days vs 1–99 days) was significantly associated with past-year CUD or abuse, while findings for duration of use (continued use for 24 months vs initiation or re-initiation in past year) were not significant [4]. Similarly, overall duration of non-medical use (short-term vs lifetime use) was not associated with timeline follow-back measures of alcohol use, Alcohol Use Disorder Identification Test (AUDIT) total score, or the Marijuana Dependence Scale in unadjusted analyses [153]. Regarding types of use, medical cannabis use was significantly positively associated with substitution of cannabis for a prescription drug compared to non-medical use in an unadjusted analysis [109].

### End-stage cancer

Sixteen systematic reviews [32–36, 39, 41, 45, 46, 50–52, 54–58], eleven RCTs [63, 64, 68, 70, 71, 74–77, 81, 85], and five NRSs [98, 133, 138, 152, 161] provided data regarding the effects of cannabis in end-stage cancer patients. **Table 4** reports their key study characteristics. Effect direction plots provided in **S8 Text** provide a graphical summary of study-specific findings.

**Table 4:** Characteristics of studies evaluating the impacts of cannabis use in individuals with end-stage cancer.

Number of studies	Designs	Primary studies	
		Cannabis use type	Funding
32	Overviews of reviews: 4 (in 5 publications) [32–36]	Medical, overseen by physician: 14 [63, 64, 68, 70, 71, 74–77, 81, 85, 98, 133, 152]	Non-industry: 5 [63, 64, 68, 74, 75]
	Systematic review: 12 [39, 41, 45, 46, 50–52, 54–58]	Mixed use: 1 [138]	Industry: 4 [70, 76, 133]
	RCTs: 11 [63, 64, 68, 70, 71, 74–77, 81, 85]	Not reported/Unclear: 1 [161]	Not funded: 1 [85]
	Prospective cohort: 3 [98, 133, 161]		Not reported: 6 [71, 77, 98, 138, 152, 161]
	Retrospective cohort: 1 [152]		
	Cross-sectional: 1 [138]		

**Fig 6:**
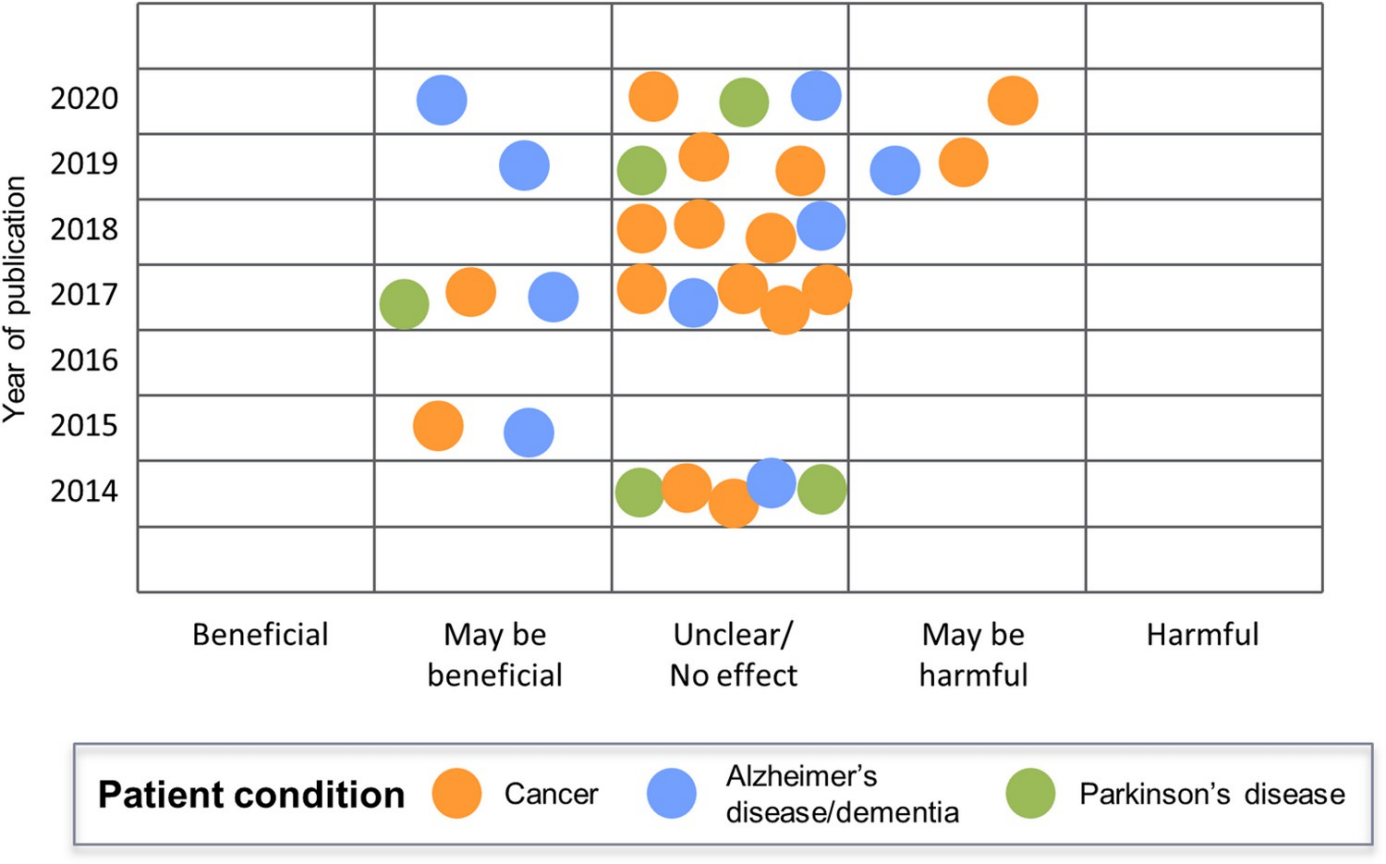
Overall conclusions in systematic reviews regarding the use of cannabis in specific patient conditions, by year of publication.

Across all outcome categories, evidence was sparse and inconsistent. Many primary studies focused on the effect of cannabis on cancer pain, with meta-analyses of RCTs reported in systematic reviews often demonstrating beneficial effects that did not reach statistical significance [39, 41, 46, 51, 57]. These potential improvements in analgesia were offset by several recent meta-analyses that suggested significant increases in AEs [46] and somnolence [39], as well as significantly decreased health-related quality of life (HR-QoL) [50] for patients with cancer using cannabis medications. For all meta-analyses, where reported, the level of evidence for these findings was reported to be very low, indicating that interpretation of the results should be made with caution (see **S8 Text** for a summary of information from systematic reviews). The six most recent systematic reviews/overviews published from 2019–20 concluded that cannabis use in end-stage cancer patients results in potentially harmful [39, 50] or no/unclear effects (**Fig 6**) [36, 41, 45, 46], while older reviews (2014–18) concluded potentially beneficial [56, 57] and unclear/no effects [32–35, 51, 52, 54, 55]. Regarding systematic review quality, one older review [57] was assessed to be of moderate quality based on AMSTAR-2 criteria, while all others were assessed to be of low or critically low quality.

Primary studies that evaluated cannabis effects in patients with cancer demonstrated trends within each design: generally, RCTs demonstrated few significant benefits for patients [63, 64, 68, 70, 71, 74–77, 81, 85], while cohort studies reported significant benefits with greater frequency [98, 133, 152, 161] (see effect direction plots provided in **S8 Text**). Differences between RCTs and cohort studies were most apparent regarding mental health outcomes. Many significant mental health harms were reported in those using cannabis within RCTs that included significantly decreased cognitive functioning, concentration, and memory with either Sativex^®^ or THC extract, respectively [76]; significantly increased psychoactive effects and sedation with THC extract [71]; and significantly decreased anorexia-specific quality of life (QoL) with dronabinol [74]. In addition to these harms, few significant mental health benefits were found: a significantly higher proportion of patients taking dronabinol reported relaxation to be “pleasant” [63] and significantly more patients taking THC extract reported increased social functioning compared to placebo [76]. Conversely, in cohort studies, only significant mental health benefits were identified: nabilone significantly decreased anxiety and significantly increased QoL in adjusted analyses [133] and loose-leaf cannabis significantly decreased anxiety and depression and significantly increased QoL in unadjusted analyses of matched data [161].

Gastrointestinal endpoints were commonly reported in the included RCTs and cohort studies. Five RCTs reported the effects of cannabis on nausea in end-stage cancer patients and found no significant benefits with the use of dronabinol [63], nabilone [68, 77], Sativex^®^ [76], THC:CBD extract [64], or THC extract [64, 76] compared to placebo. Conversely, two cohort studies identified significantly improved nausea with nabilone [133] or loose-leaf cannabis [161], when confounding was controlled through matching or multivariable modeling. Similarly, the consumption of anti-emetic medication (a proxy for nausea or vomiting) was no different in nabilone or placebo recipients in an RCT [68]; however, nabilone was found to significantly reduce the proportion of patients taking anti-emetics in multivariable adjusted models in a prospective cohort study [133]. A recent systematic review conducted meta-analyses of nausea and vomiting data, respectively, reported in four RCTs (n = 1,095 patients) and found non-significant harmful effects for both outcomes [39]. Inconsistent findings for overall nausea and vomiting were found within and across the RCTs included in our review: THC significantly decreased the proportion of patients experiencing any nausea or vomiting on day 1 of administration but not on days 2–4 [71]; after two weeks, THC had no significant effect on a nausea and vomiting score, but THC:CBD significantly worsened it [76]; after six weeks, there were no significant differences between THC extract, THC:CBD extract, or placebo in the frequency of nausea and vomiting [64]. No cohort studies evaluated overall nausea and vomiting as an outcome. No clear and consistent findings were identified for appetite, dietary intake, or weight outcomes. Dronabinol was found to significantly improve measures of appetite and protein intake, but had no significant effects on other measures of dietary intake in an RCT [63]. Similarly, nabilone significantly increased the intake of carbohydrates [85], but had no effect on many other measures of appetite, dietary intake, and weight change in RCTs or adjusted analyses in cohort studies [68, 85, 133]; in another RCT, Sativex^®^ significantly decreased appetite scores, but had no effect on another measure of appetite loss [76]. As well, Sativa-derived THC and THC:CBD extracts and loose-leaf cannabis had no significant effects on appetite in two RCTs [64, 76] and a prospective cohort study [161]. In meta-analyses reported in two systematic reviews that included the same three RCTs, pooling of various cannabis products demonstrated non-significant beneficial impacts on appetite [50, 51].

Several other endpoints were reported in primary studies of patients with end-stage cancer, with few statistically significant effects being reported. A variety of sleep measures were evaluated in five RCTs [63, 68, 70, 76], with significant benefits found only for sleep disruption scores for Sativex^®^ [70] and the proportion of patients reporting sleep to be “pleasant” for dronabinol [63]; non-significant beneficial or harmful effects were reported for all other sleep outcomes. Incoordination was significantly increased in patients taking THC extract compared to placebo in one trial [71], but no significant effect was found in patients taking synthetic THC compared to placebo in another trial [74]. A recent systematic review presented a meta-analysis of four studies reporting dizziness data and found a non-significant trend toward increased dizziness (I^2^ = 0%) [39]. Two of the RCTs were not included in our review due to a failure to meet all eligibility criteria [164, 165], and the other two RCTs were included in our review but did not have data extracted for the dizziness outcome because only raw data were reported [70, 76]. We included a different RCT reporting an analysis of frequency of vertigo that found also no significant difference between groups [64].

### Alzheimer’s disease/dementia

Six RCTs [60, 72, 86–89] and three NRSs [148, 156, 158] evaluated the effects of medical cannabis use in patients with Alzheimer’s disease/dementia, in addition to nine systematic reviews that reported at least one synthesis of Alzheimer’s disease/dementia studies (i.e., some of these systematic reviews included multiple patient conditions, not just Alzheimer’s disease/dementia) [32, 36–38, 47, 49, 51, 53, 58]. Key study characteristics are reported in **Table 5**, while detailed effect direction tables summarizing their findings have been provided in **S9 Text**.

**Table 5:** Characteristics of studies evaluating the impacts of cannabis use in individuals with Alzheimer’s disease or dementia.

Number of studies	Designs	Primary studies	
		Cannabis use type	Funding
18	Systematic review: 9 [32, 36–38, 47, 49, 51, 53, 58]	Medical, overseen by physician: 9 [60, 72, 86–89, 148, 156, 158]	Non-industry: 5 [60, 72, 86–88]
	RCTs: 6 [60, 72, 86–89]		Mixed (industry and non-industry): 1 [89]
	Non-randomized trial: 2 [148, 156]		Not reported: 3 [148, 156, 158]
	Retrospective cohort: 1 [158]		

Most primary studies evaluated dronabinol [89, 156, 158], nabilone [72], or Namisol^®^ [60, 86–88] in pill form, while one study evaluated a generic THC extract in oil form [148]. Evidence for Alzheimer’s disease/dementia was sparse and inconsistent: few significant clinical benefits were reported in RCTs and many more in the non-randomized trials and cohort study, and several significant harms were reported in RCTs but none in the non-randomized trials and cohort study. Two recent systematic reviews published in 2019 and 2020 concluded, based on the totality of evidence reviewed, that cannabis use may be beneficial in Alzheimer’s disease/dementia patients [34, 47]; however, a third review (2020) suggested that there were no clear benefits or harms [37], and another from 2019 concluded that cannabis use may be harmful [49] (**Fig 6**).

Multiple related RCTs from the Netherlands [60, 86–88] evaluated Namisol^®^ use in patients with Alzheimer’s disease/dementia and observed no significant physical or mental health benefits. Instead, the studies identified several significant harms, mainly related to worsening measures of balance [60, 87, 88] and psychedelic effects [60], despite no difference from placebo in the total number of AEs reported [60, 86, 88]. Although not evaluated as extensively in the literature, dronabinol [89, 156, 158], nabilone [72], and a THC extract [148] were all associated with significantly improved agitation and aggression in individual NRSs, although the analysis of THC extract recipients was unadjusted. When seven studies [86, 87, 89, 156, 166, 167] in a systematic review [49] were pooled in a meta-analysis of cannabis effects on agitation, no significant effect was found; however, the meta-analysis had very high heterogeneity (I^2^ = 86%). When the meta-analysis was stratified by cannabis product, pooling of three studies [86, 87] that evaluated Namisol^®^ demonstrated no significant effects (I^2^ = 0%), while pooling of four studies [89, 156, 166, 167]—two of which did not meet inclusion criteria for our review [166, 167]—that evaluated synthetic cannabinoids (dronabinol or nabilone) trended toward a favourable but non-significant response. However, the latter meta-analysis had significant heterogeneity (I^2^ = 90%) and imprecision, suggesting that the studies were too different in some aspects to be pooled. Similarly, in individual studies, dronabinol (NRS, unadjusted analysis) [156], nabilone (RCT) [72], and THC extract (NRS, unadjusted analysis) [148] were observed to have potentially significant benefits on neuropsychiatric symptoms, but not Namisol^®^ (RCTs)^®^ [86, 87]. A meta-analysis of five studies [86, 87, 156, 166, 167] in a systematic review [49] found no significant effects on neuropsychiatric symptoms, possibly due to relatively heavier weighting of the two Namisol^®^ RCTs [86, 87] with non-significant effects. Although there was no statistical heterogeneity in this meta-analysis, there were concerns regarding imprecision. Aberrant motor behaviour and motor activity may also significantly improve with administration of dronabinol [156] or THC extract [148] (both NRSs, unadjusted analyses), while dronabinol (one RCT [89]; and unadjusted analyses in one NRS [156] and one cohort study [158]) and nabilone (RCT) [72] may also improve some measures of nutrition. However, a meta-analysis of three studies [87, 89, 166] (one of which did not meet our inclusion criteria [166]) in a systematic review [49] did not find a significant effect on body mass index (BMI), possibly due to heterogeneity (I^2^ = 62%). When stratified by baseline BMI (high and low), no significant effect of cannabis was found in those with a high baseline BMI (two studies [86, 166]); however, cannabis significantly increased BMI in those with a low baseline BMI (one study [89]). Regarding potential harms, a meta-analysis in a systematic review [49] identified significantly greater risk of sedation or somnolence when four studies [86, 87, 89, 166] evaluating dronabinol, nabilone, and Namisol^®^ were pooled. As well, in one RCT, a significantly higher number of patients receiving nabilone experienced treatment-emergent AEs, but cognition scores were significantly better in those receiving nabilone compared to placebo [72]. No included studies compared AEs in patients receiving either dronabinol or THC extract, respectively, to a placebo group, meaning that the harmful effects of dronabinol and THC extract in this vulnerable patient group may not have been sufficiently evaluated.

### Parkinson’s disease

Five RCTs [65, 66, 69, 79, 83] and five NRSs (two prospective cohort [131, 149] and three cross-sectional studies [97, 154, 160]) evaluated the impacts of cannabis use in individuals with Parkinson’s disease, while five systematic reviews reported at least one synthesis [36, 42, 48, 53, 58]. Key study characteristics are reported in **Table 6**, and **S10 Text** presents tables of detailed effect direction plots.

**Table 6:** Characteristics of studies evaluating the impacts of cannabis use in individuals with Parkinson’s disease.

Number of studies	Designs	Primary studies	
		Use type	Funding
15	Systematic review: 5 [36, 42, 48, 53, 58]	Medical, overseen by physician: 5 [65, 66, 79, 131, 149]	Non-industry: 3 [69, 83, 154]
	RCTs: 5 [65, 66, 69, 79, 83]	Medical, not prescribed: 1 [154]	Mixed (industry and non-industry): 1 [79]
	Prospective cohort: 2 [131, 149]	Mixed use: 1 [97]	Not funded: 2 [66, 149]
	Cross-sectional: 3 [97, 154, 160]	Immediate effects in lab: 2 [69, 83]	Not reported: 4 [65, 97, 131, 160]
		Not reported/Unclear: 1 [160]	

Two RCTs randomized subjects to nabilone or placebo in pill form [79, 83], two to oral CBD (synthetic [69] and an unclear formulation [66] as pills), and one to a natural THC:CBD extract (2:1, Cannador, Berlin) [65]. Other natural products of unclear formulation that were smoked, vaped, or used as oil were evaluated in cohort and cross-sectional studies [97, 131, 149, 154]. One cross-sectional study that compared cannabis to other analgesics did not report details of the cannabis products used [160]. Another survey compared the effects of whole plant comparisons (i.e., fresh vs dried, flowers vs leaves) and frequency of use amongst those who used cannabis [154]. Adverse events were reported in RCTs but not in NRSs. Almost all effects and associations reported in NRSs were beneficial; however, given that none of the NRSs controlled for confounders and that the resulting potential risk of bias in the reported size and significance of effects would be high, our main summary focuses on the findings of the five RCTs, with findings from NRSs presented more briefly below.

The majority of findings reported in the five RCTs were non-significant for all outcome types (see effect direction plots provided in **S10 Text**). Similarly, most systematic reviews that summarized the impacts of cannabis use in Parkinson’s disease failed to make clear conclusions [36, 42, 48, 58], including two published in 2019 [48] and 2020 [42], although one SR from 2017 suggested possible benefits of use [53] (**Fig 6**). When significance was reached in RCTs, the effect of cannabis was typically beneficial. However, for all outcomes, significant beneficial effects were offset by one or more non-significant effects either in the same study for differing outcome definitions or in other studies. Thus, equivocal evidence was found for all reported outcomes due to lack of consensus within and across RCTs. Significant beneficial impacts of cannabis were found for dyskinesia (one [83] of three RCTs [65, 69, 83] evaluating the outcome), tremors (one RCT reporting both significant and non-significant effects [69]), non-motor symptoms (one RCT reporting both significant and non-significant effects [79]), apparent efficacy (one [79] of three RCTs [65, 66, 79]), activities of daily living (ADLs; one [66] of two RCTs [65, 66]), sleep (one [79] of two RCTs [65, 79]), and anxiety (two RCTs reporting both significant and non-significant effects within the study [69, 79]). Quality of life was significantly reduced in one [66] of three RCTs [65, 66, 79] that assessed this outcome. Many outcomes demonstrated no significant effect of cannabis in the included RCTs: motor symptoms [66, 79, 83]; mobility [65, 66]; pain/discomfort [65, 66, 69, 79]; mental and physical sedation [69, 79]; heart rate/blood pressure/EKG [69, 79]; blood tests [66, 79]; cognitive functioning [66, 69, 79]; emotional functioning [66]; depression [79]; social functioning [66]; suicidal behaviour/ideation [79]; inappropriate behaviour [79]; mentation, behaviour, and mood [66]; stigma [66]; overall AEs [79]; serious AEs (SAEs) [79]; complication of therapy [66]; response to levodopa treatment [83]; and neuronal viability [66].

Variability in outcome definitions does not fully explain the differences in effects found within and across RCTs. For example, there was high diversity in dyskinesia outcome measures, including a tapping test [69]; the Rush Dyskinesia scale [65, 83]; the Unified Parkinson’s Disease Rating Scale (UPDRS), questions 32–34 [65]; the Bain scale [65]; and numerous measures of duration of dyskinesia with respect to “on” and “off” periods [65, 83]. All were found to be non-significant, except for the Rush Dyskinesia scale in one [83] of the two studies [65, 83] reporting it. In another example, there was no variability in the QoL outcome definition across three studies, and yet one reported a significant harmful effect (n = 21; CBD powder) [66], while the other two reported non-significant beneficial effects [n = 19 (Cannador extract) [65] and 38 (nabilone) [79], respectively]. Inconsistencies in effects and significance were present despite the same outcome definition and similar sample sizes, indicating other heterogeneity, such as cannabis products, may have influenced the effects.

Briefly, in the five included NRSs [97, 131, 149, 154, 160], none of which adjusted for confounding, whole-plant cannabis use was found to have significant beneficial effects and associations for almost all reported outcomes, with little conflicting evidence across studies. Significant beneficial effects and associations were reported for dyskinesia (cohort study [131] and cross-sectional study [97]), muscle spasticity (cohort study [131] and cross-sectional study [97]), tremors (cohort study [131] and cross-sectional study [97]), pain (two cohort studies [131, 149] and a cross-sectional study [97]), apparent efficacy (cohort study [149] and cross-sectional study [97]), motor symptoms (cohort study) [131], and physical health symptoms (cohort) [131]. In a telephone survey, significant positive associations were reported between whole-plant cannabis use and mobility [97], sleep [97], appetite or food intake [97], libido [97], and attention [97], and significant negative associations were reported with nausea [97], constipation [97], depression [97], and balance or falls [97]. Only non-significant associations were found between whole-plant cannabis use and posture [131], urination [97], and memory [97]. In those who used cannabis, improvement in dyskinesia was not associated with fresh vs dried product or use of flowers vs leaves; however, frequency of use ≥ once a day was associated with dyskinesia improvement over < once a day use [154]. One small prospective cohort study that evaluated pain with both short- and long-term follow-up times found significant reductions in pain measured by either the Pain Rating Index (PRI) or a visual analog scale (VAS) at 30 minutes post-treatment compared to pre-treatment, but non-significant reductions in the same measures after ≥ 10 weeks of continued cannabis use [149]. Two other studies used different outcome measures and found significant reductions in pain at 30 minutes post-use (prospective cohort study) [131] and after 3–84 months of treatment (telephone survey) [97]. Another survey found no association between type of analgesic used (e.g., cannabis, paracetamol, NSAIDs, pregabalin) and self-reported response to analgesia in PD patients with non-low-back pain [160].

### Other indications

This review also identified evidence regarding effects of cannabis use in older adults with a variety of other conditions and indications beyond those specified a priori. In the sections below, we provide brief summaries of this evidence, grouped by indication. Direction of effect plots for these indications can be found in **S11 Text**.

#### Chronic non-cancer pain

One non-randomized trial [99], two prospective cohort studies [101, 111], and three cross-sectional studies [140, 150, 151] assessed cannabis use for general chronic non-cancer pain [99, 101, 111, 140, 150, 151]; one additional cross-sectional study included older adults who used medical cannabis of whom 61% had a pain-related condition (chronic non-cancer or cancer-related) [110], and one RCT included patients with rheumatoid arthritis [62]. All significant physical health, mental health, and AE-related effects and associations were beneficial; however, almost all were reported from unadjusted analyses in NRSs. Evidence from the single RCT suggested significant improvements in several pain measures [62] for rheumatoid arthritis patients taking Sativex^®^, while a single adjusted analysis in a cross-sectional study demonstrated a significant negative association between use of whole-plant cannabis and waking at night in those with chronic non-cancer pain, generally, although no significant associations were found for other indicators of insomnia such as sleep initiation and early awakening [150]. A single cross-sectional study reported drug- and alcohol-related outcomes in patients with chronic non-cancer pain and found significant associations between cannabis use and misuse of prescription opioids, hazardous alcohol use, and nicotine use in unadjusted analyses; however, only an increased risk of misuse of prescription opioids remained significant after adjustment for confounding [140].

Unadjusted analyses found significant improvements in apparent efficacy and pain outcomes (one non-randomized trial and two cohort studies) [99, 101, 111], sleep (one RCT and one cohort study) [62, 101], vitality (cohort study) [101], global QoL (non-randomized trial) [99], anxiety (cohort study) [101], and overall AEs (cross-sectional study) [140]. Non-significant effects were also reported for these outcomes, potentially due to differences in patient characteristics or outcome definitions. A cross-sectional study found non-significant positive associations in unadjusted analyses between mixed cannabis use (compared to medical use) and past-year opioid use and benzodiazepine use, and non-significant negative associations with measures of global QoL and physical and mental health [110]. Many other non-significant effects were reported from unadjusted analyses of physical health, mental health, AE-related, and drug-related outcomes across studies of chronic non-cancer pain (**S11 Text**).

#### Joint replacement

Patients receiving joint replacements were included in three retrospective cohort studies that evaluated the effects of cannabis use [112, 120, 122]. Patients who self-reported using cannabis [122] or who were administered 10 mg dronabinol [120] had no significant differences in measures of analgesic use in hospital, respectively, compared to patients who did not use cannabis or dronabinol in unadjusted analyses; however, compared to non-use, dronabinol significantly reduced hospital length of stay [120], while self-reported cannabis use did not [122]. Patients who screened positively for urine THC pre-operatively had significantly poorer pre-anaesthetic health scores and were significantly more likely to screen positively for opioids pre-operatively than patients who screened negatively for THC in unadjusted analyses [112]. However, adjusted analyses demonstrated no significant difference between patients who screened positive or negative for urine THC in post-operative complications, 90-day readmissions, reoperations, or deaths up to 90 days post-op [112]. Many other outcomes with non-significant effects were reported in the three studies.

#### Neuropathy

Three RCTs evaluated the impacts of cannabis use on peripheral neuropathy [91] and diabetic neuropathy [82, 90]. One trial of diabetic neuropathy patients found significant improvements in pain scores with high-dose THC extracts (28 mg) but not with lower doses (4 or 16 mg) compared to placebo, although other measures of pain (e.g., proportion of patients achieving ≥ 30% pain reduction) were not significantly affected, and the benefits were offset by significant increases in somnolence and psychoactive effects [90]. Another trial of patients with peripheral neuropathy found significant improvements in measures of some non-pain sensations but not others with CBD topical cream [91]. A third trial found no significant differences in any measured physical health or mental health outcomes in diabetic neuropathy patients randomized to either Sativex^®^ or placebo [82].

#### Chronic obstructive pulmonary disease

The effects of cannabis in patients with COPD were evaluated in two RCTs [59, 80] and one retrospective cohort study [155]. No significant impacts were identified on measures of spirometry when Sativex^®^ was compared to placebo in a RCT [80]. Comparisons of high-dose vs low-dose THC, with or without CBD, also suggested no effects on spirometry [80], but identified significantly negative psychoactive effects [59] and “bad drug effects [59],” as well as significantly higher risks of hospitalization for COPD or pneumonia [155] and all-cause mortality [155] in adjusted analyses. One RCT identified a significant benefit of high-dose compared to low-dose THC for “good drug effects” and one self-reported measure of anxiety, but no significant difference between groups for another self-reported measure of anxiety/relaxation [59].

#### Multiple sclerosis

Two RCTs randomized patients with multiple sclerosis to Sativex^®^ and placebo [67, 78]. Significant benefits in apparent efficacy [78] and some nerve conduction measures [67] were reported; however, no significant effects were found for pain scores [67], muscle spasticity [78], strength [78], gait velocity [78], or sleep [78].

#### Trauma

Trauma patients who used cannabis were compared to those who did not in two retrospective cohort studies [114, 127]. In adjusted analyses, cannabis use in older adults did not impact mortality [127] or the need for intubation [127]; however, it was significantly associated with ICU admission [127] and need for an operation due to the trauma [127]. Unadjusted analyses suggested significant associations of non-medical cannabis use with lower blood pressure and higher heart rate (potentially early signs of shock) [114], and non-significant associations with consciousness/coma [114], injury severity [114], and length of stay in the hospital or ICU [114].

#### Cardiac conditions

Two retrospective cohort studies evaluated the impact of cannabis use [123] and misuse [125] in patients with cardiac conditions. Adjusted analyses suggested that older adults who used cannabis who suffered an acute myocardial infarction have significantly reduced risks of shock (ages 50–69 years) and mortality (ages 50–59 only), respectively, compared to those who did not use cannabis [123]. No significant effects were found at any age >50 years on the risks of mechanical ventilation [123], ventricular tachycardia/fibrillation/cardiac arrest [123], or a composite of death, mechanical ventilation, cardiac arrest, placement of an intra-aortic balloon pump, or shock [123]. In unadjusted analyses, patients undergoing percutaneous coronary interventions who misused cannabis were at significantly greater risk of post-intervention bleeding complications, if they were between the ages of 66 and 75 years, but not in younger or older age categories [125]. No other post-intervention AEs were associated with cannabis use, including vascular complications, stroke/transient ischemic attack, or death [125].

#### Homelessness

Past-six-month use of non-medical cannabis in people without housing was evaluated in two cohort studies that used the same sample of participants [93, 142]. A significantly higher risk of moderate-to-high physical symptomatology was found with moderate-risk cannabis use (i.e., Alcohol, Smoking and Substance Involvement Screening Test (ASSIST) score of ≥ 4) compared to no moderate-risk cannabis use after adjustment for confounders [142]. The risk of falling was significantly increased in people without housing who consumed cannabis non-medically in an unadjusted analysis [93].

#### Alcohol use

One case-control [157] and two cross-sectional studies [115, 130] assessed impacts of cannabis use in participants who consumed alcohol. All reported adjusted analysis findings. Co-use of cannabis and alcohol was associated with increased prevalences of past-year prescription drug misuse (non-medical cannabis use) [130] and past-month binge drinking [115], and decreased odds of alcohol-related liver cirrhosis in those 50–59 years of age, but not over 60 years [157].

#### Additional stand-alone conditions and indications

Six RCTs [20, 61, 73, 81, 84, 92], five cohort studies [121, 136, 137, 145, 159], and one cross-sectional study [141] evaluated the effects of cannabis use on a variety of other patient conditions and indications and reported a mix of findings.

A small cross-over study randomizing healthy older adults who did not use cannabis to three doses of Namisol^®^ and placebo found significantly greater overall AEs for all doses compared to placebo and for high-dose (6.5 mg) compared to either lower dose (3 or 5 mg) [20]. No significant effects were found on balance or concentration, and substantial variability was found between participants in pharmacokinetic parameters (see section on “Cannabis use in healthy older adults and the older general public”).

In other studies, significantly beneficial effects were found in

- Ever and former tobacco use (spirometry outcomes; non-medical use only; cohort study, adjusted findings) [137],
- Amyotrophic lateral sclerosis (apparent efficacy, pain score, and muscle spasticity; RCT) [81],
- Diabetes (apparent efficacy and various diabetic blood tests; RCT) [73],
- Failed back surgery syndrome (pain scores, sleep, mobility, physical health symptoms, QoL, social functioning, and mood; cohort study, unadjusted findings) [136] and
- Older adults with sedentary lifestyles (BMI; cohort study, adjusted findings) [159].

Significant harmful effects were found for the following indications:

- Diabetes: a high-dose cannabis preparation (10 mg Tetrahydrocannabivarin + 200 mg CBD) was found to significantly increase the risk of depression in an RCT [73]
- Intraocular hypertension: 40 mg CBD significantly increased both blood pressure (at 60 and 90 minutes) and intraocular pressure, respectively, while low-dose THC (5 mg) significantly increased heart rate at 90 minutes but had no effect on intraocular pressure in an RCT [84].
- Surgery: pain scores were significantly increased for those taking high-dose Nabilone (2 mg) compared to those taking 1 mg Nabilone, ketoprofen, or placebo, respectively in an RCT [61].
- Non-traumatic aneurysmal subarachnoid hemorrhage: significantly higher risk of hospital readmission was found if patients had current CUD compared to those who did not in an adjusted analysis in a cohort study [145].
- People who are HIV-positive and taking HIV medication: cannabis use had no significant association with response to HIV therapy in unadjusted analyses, despite being significantly associated with reduced HIV drug adherence in a cross-sectional study [141].

Only non-significant effects were found for the following conditions:

- Cervical dystonia: dronabinol had no significant effects on pain scores, apparent efficacy, torticollis severity or activities of daily life scores in an RCT [92].
- Reduced appetite due to chronic disease: dronabinol had no significant effects on appetite, food intake, weight, or blood albumin levels in unadjusted analyses in a cohort study [121].

Many other non-significant effects were reported in the above studies.

### Clinical subgroup findings

As per our a priori objectives, data for subpopulations of interest were captured; for brevity, we refer readers to **S12 Text**, where we provide both descriptive text and tables of findings pertaining to all subgroups. Additionally, in earlier sections, we have reported subgroup findings where they provided additional context of the data being described. Overall, data related to the subgroups of sex, older adult age group (i.e., 50–64 years, 65+ years, etc.), residential setting (e.g., ambulatory versus inpatient care), and illicit drug use were identified. As well, some studies focused entirely on some subpopulations of interest that have been summarized as “patient conditions” in the main text, including existing physical and mental health conditions (i.e., end-stage cancer, Alzheimer’s disease, Parkinson’s disease, etc.), accommodation status (i.e., homelessness [93, 142]), and use of other substances (i.e., alcohol [115, 130, 157], tobacco [137], prescription opioids [117], and heroin [118]). We were unable to locate information related to the following key subpopulations of interest: race/ethnic groups, frailty, employment status, marital status, or other accommodation statuses (e.g., alone, shared).

### Cannabis comparisons amongst individuals who use cannabis

Similar to subgroup data, we planned a priori to gather data on comparisons of cannabis consumption other than use vs no use/placebo (i.e., comparisons of use amongst those who used cannabis). For brevity, we direct readers to **S13 Text**, where we provide a descriptive synthesis of findings with corresponding tables. These data pertain to comparisons of use type (i.e., medical vs non-medical), dose (e.g., grams per month; number of joints per month), THC and CBD concentrations, frequency of use, duration of use, cannabis plant characteristics (e.g., flowers vs leaves, fresh vs dried), and use of other substances. Data were identified for a variety of patient conditions. We were unable to locate comparisons of consumption methods (e.g., smoking, vaporising, edibles), which was an a priori comparison of interest.

## Discussion

Legalization of cannabis in many jurisdictions around the world has the potential to lead to greater availability and accessibility of cannabis products for all age groups [10]. As North American baby boomers born in the 1950s and 1960s age, attitudes toward cannabis use in the older adult cohort have changed, leading to higher rates of use in this demographic [2, 116, 168, 169]. However, the effects of cannabis use in older adults have not been well defined, beyond cannabis use for specific health conditions common to older adults, such as cancer pain, chronic non-cancer pain, and palliative medicine [32, 33, 46, 51, 55]. Previously only one published systematic review of cannabis effects focused specifically on older adults; however, it was limited to medical cannabis use [58]. In the current review, more than 130 studies were found that contribute to the current evidence regarding positive and negative health effects and associations with cannabis use for both medical and non-medical purposes in older adults.

While a large number of studies were mapped in our review, we caution readers not to over-interpret the available data. Many findings were supported by single studies only, were derived from unadjusted analyses in non-randomized designs, were reported only in cross-sectional or case-control studies that are minimally informative for causal inferences, or may have limited biological plausibility. Furthermore, although we prioritized the reporting of findings from adjusted analyses in NRSs, it should be noted that we did not assess the level of adjustment made for each finding (e.g., adjustment for all critical confounders), and consequently, even adjusted results may still be biased by residual confounding. As well, there was limited assessment of harms in the included NRSs. For these reasons, the benefit-to-risk ratio of cannabis use is unclear. Therefore, we recommend that readers consider the nature and potential limitations of the primary research sources underlying the benefits, harms, and associations mapped in this scoping review, prior to making decisions about prescribing or consuming cannabis.

A recent Canadian study found > 50 reasons for authorization of medical cannabis across all ages, with the majority not supported by evidence of long-term efficacy [10]. While we identified studies that reported benefits of medical cannabis, such as alleviation of cancer pain and improved QoL in cancer patients, the extent of evidence for specific patient conditions was generally sparse. Additionally, while benefits were occasionally observed in RCTs, these were often accompanied by an observed increased risk of one or more harms associated with cannabis use. Benefits were more often noted in NRSs; however, few of these studies also included evaluations of harms. Where significant associations were identified, often there was conflicting evidence within and/or across studies. Conflicting findings may reflect heterogeneity of patient demographics, sample sizes, cannabis interventions, outcome definitions, follow-up times, residual confounding, or other factors. While there may be potential for medical cannabis to offer benefits to some patients with conditions such as end-stage cancer, Alzheimer’s disease, and Parkinson’s disease, the quantity and quality of evidence is limited at this time. Similar conclusions were observed in recent systematic reviews reporting findings for cannabis use for end-stage cancer [39, 41, 46, 50, 51], Alzheimer’s disease/dementia [37, 38, 47, 49, 51], and Parkinson’s disease [48]. None of these systematic reviews could recommend cannabis use for these conditions. It should be noted that meta-analyses reported in the included systematic reviews often pooled data from differing cannabis products. Different cannabis products may have differing effects; therefore, pooling data from multiple products in meta-analyses may result in high heterogeneity and reduced likelihood of identifying significant effects. Similarly, as cannabis includes many different cannabinoids wherein the type and route of administration impact side effects, it is also challenging to concisely summarize side effect data. While there exists some evidence of benefit of cannabis for certain conditions in the general adult population > 18 years of age (e.g., anxiety, PTSD), there was limited evidence mapped in our review for these and many other conditions, indicating that focused research in older adults is needed. A recent narrative review suggested that medical cannabis may be considered as a “last-resort” therapy or adjunct/replacement treatment, when all else fails, in cases of Parkinson’s disease or dementia for some symptoms, although the clinical data are still incomplete [170]. Of note, none of the publications included in our review explicitly reported harms related to drug interactions, medical cannabis misuse, or withdrawal. Additional rigorous research of the effects of cannabis in these and other conditions common to older adults remains vital, and physicians, health providers, patients, caregivers, and other stakeholders may wish to proceed cautiously in using cannabis for medical purposes at this time. Development of tailored dissemination strategies to inform and educate clinicians, healthcare professionals, pharmacists, caregivers, and patients may be important to help reduce the risks associated with the use of cannabis for medical purposes in older adults.

Cannabis use for non-medical purposes, specifically, was studied infrequently in the mapped studies. Several observational studies explored the effects of non-medical (sometimes referred to as “recreational”) cannabis use. In these studies, although non-medical cannabis use was found not to compromise lung function in individuals who used tobacco [137], detrimental effects of non-medical use were identified, albeit in unadjusted analyses: older people without housing that consumed cannabis were more likely to fall [93] and older trauma patients were more likely to demonstrate early signs of shock, if they consumed cannabis [114]. Of greater prevalence in the literature were observational studies that either evaluated associations with any cannabis use (i.e., medical or non-medical; n = 14 studies) or that didn’t report the type of use (n = 28 studies), with more than half of these studies analyzing large national survey data (i.e., the older general public). In studies of the older general public, no significant associations with beneficial physical or mental health outcomes were reported from adjusted analyses, except possibly reduced future incidence of head/neck [129] and prostate cancers [143]. As illustrated in our bubble plots, associations with harmful physical and mental health outcomes greatly outnumbered beneficial outcomes in RCTs and in adjusted analyses reported in cohort studies. It should be emphasized that associations from cross-sectional and sequential studies do not denote causality, and that cannabis use may simply be more prevalent in older adults with pre-existing physical and mental health conditions. However, the available evidence suggests that any cannabis use may be detrimental to the physical and mental well-being of older adults, although the clinical significance of these findings is unknown and many potentially beneficial outcomes (e.g., pain reduction) were not reported in studies of the older general public.

Our scoping review found very few studies that specifically examined cannabis use in older adults, relative to the volume of research available across all age groups. It also identified several important gaps in the evidence. First, we identified limited findings regarding our a priori subgroups of interest within the population of older adults. We found occasional studies that differentiated some effects of cannabis by sex [61, 64, 130] and age [116, 125, 135, 146, 157]; two related studies that explored whether the effects of Namisol^®^ in Alzheimer’s patients varied by their residential setting [86, 87]; a single study reporting cannabis effects on spirometry in individuals who formerly used tobacco amongst those who ever used tobacco [137]; and no studies reporting cannabis effects in different categories of accommodation status, marital status, gender, or frailty. We also identified few studies that made comparisons other than cannabis use vs no use/placebo, such as comparing alternative means of cannabis consumption in older adults (e.g., vaporizing, oils, edibles), doses, types of cannabinoids, cannabinoid ratios, frequencies of use, etc. These comparisons may be especially salient to the older adult population, where variations in potency and use patterns may influence health risks and benefits. We also were met with a severe lack of data for several key outcomes, including physical brain outcomes, measures of pharmacokinetics, and drug interactions. Given the plausible effects of cannabis on measures of this nature, additional research is needed to address these gaps.

### Limitations

Certain limitations of the review should be noted. First, given the extremely large volume of citations identified in our initial searches, we used study design filters to reduce search yield by only retrieving publications pertaining to the study designs of interest. These filters use comprehensive controlled vocabulary and free text to identify pertinent study designs, but it is possible that some records of potential interest may not have used the relevant vocabulary at the title, abstract, keyword and indexing level, in which case they could have been overlooked. Second, in determining eligibility of systematic reviews, our criteria defining “systematic review” were very strict, and relevant reviews may have been excluded due to poor reporting of their study methods. Regarding primary studies, our eligibility criteria were somewhat restricted in that we did not include studies reporting raw data only, with no analyses (e.g., studies that reported the numbers of patients with AEs but that did not analyze these data). As a result, single-arm studies were excluded, except if they reported pharmacokinetic data (of which we found none), and raw AE data in included studies were not charted. As well, it should be noted that scoping reviews differ from systematic reviews in several key areas. First, the objective of a scoping review is to map the available evidence on a topic not to meta-analyze or fully synthesize the evidence. As such, we provide a high-level overview of all peer-reviewed evidence, without syntheses. Second, risk of bias assessments are not conducted in scoping reviews, and so the quality of the studies underlying our findings is not known.

Regarding limitations of the available literature, many reviews and primary studies did not report age data in sufficient detail to allow determination of eligibility. As well, many primary studies of all ages of adults evaluated age-related differences, using “age” as a covariate in multivariable models; however, these were excluded because the cannabis effect for the older adult age group(s) could not be determined. It should be noted that many RCTs and cohort studies were of small sample size, which may limit generalizability and confidence in their findings. As well, adjustment for confounding was not conducted in many NRSs, further reducing confidence in their findings. The cross-sectional design of many studies provided minimal information regarding causality, and these designs were especially prevalent in studies of populations without medical conditions, limiting the ability to infer the impacts of cannabis use in the general older adult population. Criteria to define ‘current cannabis use’ also varied between studies, thereby complicating the ability to compare reported effects between studies. As can be seen in the effect direction plots provided in the supplements to this review, considerable variability exists in the measures used to capture clinical outcomes of interest. A certain degree of divergence in findings between RCTs and NRSs was noted in some cases, and patterns in outcome assessment that varied across study designs also complicates interpretations (i.e., a common omission of harms endpoints in NRSs). In general, there was a lack of overlapping treatment/exposure comparisons across multiple studies, likely due in large part to a lack of standardized cannabis interventions for specific conditions and the broad range of non-medical/recreational products available (and variations in route of administration and other factors). Taking these challenges into consideration, while there is a desire for strong interpretations of benefits with cannabis use, the general public, patients and clinicians must be aware that the evidence remains weak at this time. While lack of or weak evidence does not necessarily correlate to a lack of benefits, at this time further research in older adults that addresses these limitations is urgently needed. We are hopeful that research funders may find this mapping of evidence helpful in guiding future funding opportunities.

## Conclusions

The current scoping review mapped more than 130 studies that provide evidence regarding the benefits, harms, and associations with health outcomes of cannabis consumption in older adults. The nature of these studies was diverse in terms of populations studied, reasons for consumption, and health effects measured. Within the general population of older adults, the limited evidence base suggests that the harms of cannabis use may outweigh the benefits. Support regarding the benefits of cannabis use in older adults for medical reasons such as end-stage cancer, Alzheimer’s disease/dementia, Parkinson’s disease and other indications appears limited at this time: evidence is often inconsistent across studies within specific patient conditions. The health effects of cannabis consumption in older adults require further study, with a balanced assessment of both benefits and harms, to guide appropriate public health messaging to balance the marketing pressures of cannabis to the older adults.

## Supporting information

S1 Dataset Final data extracted from systematic reviews. (XLSX)

S2 Dataset Final data extracted from RCTs. (XLSX)

S3 Dataset Final data extracted from observational and non-randomized studies. (XLSX)

S1 Text Protocol amendments with rationale. (DOCX)

S2 Text Complete description of the methods. (DOCX)

S3 Text Literature search strategies. (DOCX)

S4 Text PRISMA ScR checklist. (DOCX)

S5 Text Studies excluded at full-text screening. (DOCX)

S6 Text Study characteristics–Evidence tables by study design. (DOCX)

S7 Text Effect direction plots–Healthy adults and general population. (DOCX)

S8 Text Effect direction plots–End stage cancer. (DOCX)

S9 Text Effect direction plots–Alzheimer’s disease and dementia. (DOCX)

S10 Text Effect direction plots–Parkinson’s disease. (DOCX)

S11 Text Effect direction plots, other patient conditions. (DOCX)

S12 Text Summary of available subgroup data. (DOCX)

S13 Text Summary table of cannabis comparisons for older adults who used cannabis. (DOCX)
