# Supplementary material for: Impacts of medical and non-medical cannabis on the health of older adults: Findings from a scoping review of the literature
Source: PLoS One. 2023 Feb 17;18(2):e0281826. doi: 10.1371/journal.pone.0281826 (PMC9937508; doi:10.1371/journal.pone.0281826)
Supplement: S1 Text — (DOCX) [file pone.0281826.s004.docx]

S1 Text: Protocol Amendments with Rationale

Draft search yields prior to protocol development indicated an extremely high volume of potential literature to screen. As a result, we elected to stratify study selection by study design, with the anticipation that we could iteratively evaluate the included evidence and develop stop rules for screening, if possible. Systematic reviews were prioritized because they provide a synthesis of the primary literature. Had we identified numerous systematic reviews specific to cannabis use in older adults that included a high volume of the primary literature, we could potentially screen a lower volume of the primary literature, potentially only the most recent evidence that may not have been captured by the available systematic reviews. However, in the original 2019 search results, we identified scant evidence in the systematic review literature that was specific to older adult cannabis use. Consequently, we were forced to screen all available primary literature and did not employ a sequential approach. Similarly, we did not prioritize data charting by study design.

During screening of primary studies, we recognized ambiguity in our protocol regarding inclusion criteria for single-arm NRSs. To be eligible for inclusion, primary studies must have reported a cannabis comparison (i.e., have two or more arms), but we also stipulated that single-arm studies were eligible. Our intention was to capture pharmacokinetic data that may have been reported in single-arm studies; however, we did not specify this. The protocol should have excluded all single-arm studies except those reporting pharmacokinetic results for older adults. These criteria were adopted for both screening levels.

As reported in the main text, the breadth of evidence available in the included systematic reviews was shallow compared to that available in the primary study literature. As a result, we prioritized reporting detailed findings from primary studies, while from the systematic reviews we only reported (1) meta-analysis findings if they directly pertained to results for primary studies that we reported in the main text and (2) overall author conclusions for the three relevant patient conditions (i.e., end-stage cancer, Alzheimer’s disease/dementia, Parkinson’s disease). As well, although we assessed the quality of the included systematic reviews using the AMSTAR-2 tool, because reviews were not prioritized for reporting, we did not report quality assessment findings in the main text. These data have been provided in a supplemental data file.

During the review process, measures of global quality of life (measures that included both health-related quality of life and mental health parameters) and measures related to the use and problematic use of other drugs and alcohol, and risk-taking behaviors were identified, and the team decided to collect data regarding these outcomes as well given their relevance.
