# Supplementary material for: Impacts of medical and non-medical cannabis on the health of older adults: Findings from a scoping review of the literature
Source: PLoS One. 2023 Feb 17;18(2):e0281826. doi: 10.1371/journal.pone.0281826 (PMC9937508; doi:10.1371/journal.pone.0281826)
Supplement: S2 Text — (DOCX) [file pone.0281826.s005.docx]

S2 Text: Complete description of the methods

Given the reflexive and iterative nature of scoping reviews, amendments to the registered protocol have been noted in the summary of our methods below and further described, along with rationale, in **S2 Text**.

Study eligibility criteria

We based our eligibility criteria on the Participants – Concept – Context (PCC) framework^24^.

***Participants:***

- Participant age: ≥ 80% of the study sample was required to be adults aged 50 years and older; formulae were used to determine age eligibility when studies included adults of all ages (see the “Additional Details of Scoping Review Methods” section below for details). Disease conditions that affect mainly older adults were used as proxies for age in cases where the age of participants was not explicitly reported; these were end-stage cancer, Alzheimer’s disease or dementia (AD/D), and Parkinson’s disease. Chemotherapy for any indication was not considered a proxy for age because patients did not necessarily have end-stage cancer.
- “Current” cannabis use: current cannabis use was not explicitly defined a priori, except that use must not have been more than one year in the past. Evaluations of the effects of age of initiation of cannabis use, lifetime/ever cannabis use, or previous cannabis use, where the older adult was no longer using, were excluded.
- Other characteristics: Individuals of any sex/gender or race were of interest. Regarding health status, healthy individuals as well as those with physical or mental health conditions, whether acute or chronic, were of interest.

***Concept:***

- Interventions: Cannabis must have been the intervention or exposure in the study.
  - Types of use: Cannabis use could be medical (overseen by a physician or self-medicated) or non-medical, of any type, with any mode of consumption (e.g., pills, smoking, vaporizing, oils, edibles).
  - Cannabis types: whole-plant/loose-leaf cannabis; purified whole-plant extracts (e.g., Nabidiolex (purified cannabidiol (CBD)), Tetrabinex (purified delta-9-tetrahydrocannabinol (THC)), Sativex^®^ (purified 1:1 THC:CBD)); synthetic cannabinoids, such as synthetic THC (e.g., dronabinol, nabilone), CBD, and their derivatives, developed through modification of the molecular structure; and other cannabinoids (i.e., not THC or CBD), whether found in the cannabis plant or elsewhere and that interact with the endocannabinoid system.^27^
  - Comparisons: use vs no use (or placebo), types of use, types of cannabis, modes of consumption, doses, etc. Analyses comparing age categories amongst individuals who used cannabis were not of interest because cannabis was not the exposure of interest.
- Outcomes: Beneficial and harmful effects of cannabis use on physical and mental health, physical brain structure, and pharmacokinetic outcomes were of a priori interest*;* examples are provided in the “Additional Details of Scoping Review Methods” section below. During the review process, measures of global quality of life (measures that included both health-related quality of life and mental health parameters) as well as measures related to the use or problematic use of other drugs and alcohol, and risk-taking behaviors emerged and were collected. We excluded single-arm studies that only reported prevalence or incidence of cannabis use in older adults and those assessing cannabis use as an outcome (however, cannabis use disorder (CUD) as a mental health outcome was eligible).

***Context:***

Studies of interest were those that focused on current cannabis consumption, in all settings, in any geographic area, and including all periods of time and durations of follow-up. Consumption of other illicit or prescribed pharmaceuticals was allowed. Regarding study design, we sought studies that were systematic reviews (including overviews of reviews), randomized controlled trials (RCT), non-randomized/observational studies (NRS) (note that observational studies are included in the NRS acronym; additional design-specific criteria are provided in the “Additional Details of Scoping Review Methods” section below)*.* Only English and French publications were considered for reasons of timeliness and cost. We excluded qualitative studies, diagnostic test accuracy studies, studies developing or validating diagnostic criteria for CUD or other cannabis-related mental health disorders, editorials, letters, commentaries, abstracts, case reports, case series under 25 patients, and narrative reviews*.*

Information sources and search strategy

Using the OVID platform, we searched Ovid MEDLINE®, including Epub Ahead of Print and In-Process & Other Non-Indexed Citations, Embase Classic+Embase, and PsycINFO. We also searched the Cochrane Library on Wiley. The initial searches were undertaken on 14 June 2019 and updated regularly to 30 November 2020. Search strategies utilized a combination of controlled vocabulary (e.g., “*cannabis,*” *“cannabinoids,*” “*marijuana use*”) and keywords (e.g., “*marijuana,*” “*CBD,*” “*Sativex*”). Filters for the research designs of interest were applied to the Ovid searches to restrict volume (>120,000 records were found in preliminary searches). Our final searches included a qualitative study design filter, but we did not incorporate the records retrieved using this filter due to volume. Vocabulary and syntax were adjusted across the databases as needed. When possible, animal-only, opinion pieces and case studies were removed. Conference abstracts were removed from Embase and Cochrane CENTRAL. The search strategies have been provided in **S3 Text**. The final search strategy was peer reviewed by another senior information specialist using the Peer Review of Electronic Search Strategies (PRESS) Checklist^171^. We also screened the bibliographies of included systematic reviews as well as any excluded systematic reviews that, during full-text screening, had been flagged by reviewers as having at least one potentially relevant primary study that had not been substantively synthesized.

Study selection process and data management

Online systematic review management software (*DistillerSR.*Version 2.35. Evidence Partners; 2021. Accessed May 2019 to November 2021. [https://www.evidencepartners.com](https://www.evidencepartners.com/)) was used for database management and study selection. Within each study design stratum, two levels of screening were conducted using a priori developed screening forms. Initially, titles and abstracts were screened, with potentially relevant references progressing to full-text screening. A pilot exercise of a random sample of references was conducted prior to screening at each level to ensure high inter-rater reliability. A liberal accelerated approach was used for both levels of screening, with one reviewer required to include a reference and agreement of two reviewers required to exclude^172^. Eligibility of included studies was further scrutinized during the data extraction process if relevance was questioned by the reviewers. Given the large volume of search results (> 30,000 records), we used DistillerSR’s® artificial intelligence active machine learning feature to implement prioritized screening during review of titles/abstracts of references found through the RCT and NRS searches. Following a training set of citations that contained key words (e.g., “cancer,” “Alzheimer’s,” “dementia,” “elder,” “geriatric,” “senior”) to increase the likelihood of identifying potentially relevant references, the remaining titles/abstracts were presented to reviewers in an order of perceived highest to lowest relevance (based on a classification algorithm). This helped to expedite identification of potentially relevant citations to be screened at full text. After every 200 citations were fully screened (i.e., included by one reviewer or excluded by two reviewers), the remaining unscreened citations were re-ordered based on their new scores. Once an estimated recall of > 95% of included studies was achieved, the AI reviewer was assigned to exclude the remaining citations (27% of NRSs and 32% of RCTs). At the time this was run, the highest prediction score that a citation was relevant was 0.038 for NRSs and 0.0482 for RCTs. A human reviewer screened all citations excluded by the AI reviewer, and any conflicts progressed to full-text screening. Disagreements at full-text screening were resolved by discussion or by consultation of a third reviewer. Further details of the screening processes have been provided in the “Additional Details of Scoping Review Methods” section below.

Data charting and quality appraisal

A standardized data charting form was developed in DistillerSR^®^. The form was refined during the data charting process as reviewers became more familiar with the content area, in keeping with the iterative and reflexive nature of scoping reviews. For each study design, charting forms were piloted with random samples of three articles, until reviewers were confident in the extraction process^24^. All data were extracted independently by two reviewers, followed by conflict resolution; a third reviewer was consulted to resolve any remaining conflicts. Briefly, the following data were collected: publication characteristics, population demographics (e.g., sex, age, race, employment status); cannabis consumption information (e.g., medical/non-medical use, types consumed, methods of consumption, dose); comparator data, where available; outcome data, including definition, direction of health effect, and statistical significance; subgroup information related to sex, gender, ethnicity, employment status, marital status, presence of specific physical or mental health conditions, and co-use of other substances; and summaries of key findings or recommendations. Outcomes reported as raw data, such as adverse events, were not extracted unless a statistical comparison, either univariable or multivariable, was made between the cannabis and comparator groups. Direction of effect was based upon the value of the effect estimate relative to null (e.g., for harmful outcomes such as presence of a mental health condition, any value < 1 was considered beneficial and any value > 1 was considered harmful). Statistical significance was as reported in the study, whether through a confidence interval, standard error, p-value, or a statement of significance.

Although quality assessment of included studies is generally not conducted in scoping reviews, we appraised the quality of the included systematic reviews because we anticipated that they would provide a comprehensive summary and map of the available primary literature. Quality appraisal of the included systematic reviews was conducted using the AMSTAR-2 tool^29^ by two reviewers, independently. Conflicts were resolved by discussion or consultation with a third reviewer. The AMSTAR-2 rating was performed to assess the entirety of each review and was not limited strictly to the sections pertaining to older adults, which may be important in reviews that included both older and younger adults. In keeping with scoping review methodology^24,173^, formal assessment of the risk of bias in primary studies was not undertaken.

Synthesis and presentation of the results

Data were synthesized using descriptive summaries, complimented by both tables and figures that presented characteristics of the included studies and summaries of clinical findings, overall and within each study. Separate syntheses were prepared for each patient condition (i.e., healthy older adults, end-stage cancer, Alzheimer’s disease/dementia, Parkinson’s disease, and other conditions) to structure reporting of the evidence. Cannabis use in healthy older adults and the older adult general public was considered separate from data related to medical conditions because it mapped the effects of cannabis use generally in older adults, rather than assessing cannabis as a treatment option or risk factor within a specific condition. Within sections that pertain to each patient condition, findings have been described according to study design (i.e., systematic reviews, RCTs, NRS) to reflect their associated level of evidence. For primary studies (RCTs, NRS), we mapped the extent of the clinical findings for cannabis use in healthy older adults for each reported outcome using bubble plots, with four dimensions: (1) **direction of effect** (*beneficial, no effect, harmful*) on the x-axis, (2) **statistical significance** reported in the study on the y-axis, (3) **outcome category** by bubble colour, and (4) **number of studies** reporting the finding as the bubble size. For systematic reviews, we used a single bubble plot to map authors’ conclusions that were reported over time regarding the use of cannabis in older adults with end-stage cancer, Alzheimer’s disease/dementia, and Parkinson’s disease. Each bubble corresponded to a review conclusion and was mapped with three dimensions: (1) **direction of effect** (*beneficial, may be beneficial, unclear/no effect, may be harmful, harmful*) on the x-axis, (2) **year of publication** on the y-axis, and (3) the **condition of interest** (end-stage cancer, Alzheimer’s disease/dimension, Parkinson’s disease) represented by the colour of the bubble. More detailed clinical findings of the beneficial or harmful effects of cannabis use reported in each study have been presented by patient condition in tabular form, with colour coding of cells within the table to reflect the direction of effect and statistical significance of each reported outcome.
