# Supplementary material for: Impacts of medical and non-medical cannabis on the health of older adults: Findings from a scoping review of the literature
Source: PLoS One. 2023 Feb 17;18(2):e0281826. doi: 10.1371/journal.pone.0281826 (PMC9937508; doi:10.1371/journal.pone.0281826)
Supplement: S3 Text — (DOCX) [file pone.0281826.s006.docx]

S3 Text: Literature Search Strategies

Ovid Multifile

*Results downloaded by study design*

Database: Embase Classic+Embase, Ovid MEDLINE(R) ALL, PsycINFO

Search Strategy:

--------------------------------------------------------------------------------

1 Cannabis/ (47495)

2 exp Cannabinoids/ (82168)

3 Marijuana Abuse/ (10414)

4 exp "Marijuana Use"/ (14205)

5 Marijuana Smoking/ (7547)

6 ("c.indica" or "c.sativa" or cannabi* or bhang or cannador or cbd or charas or eucannabinolide* or ganja or ganjah or hash or hashish or hemp or marihuana* or marijuana*).tw,kf. (136307)

7 (epidiolex or gwp 42003p or gwp42003p or nabidiolex).tw,kf. (165)

8 (dronabinol or thc or tetrahydrocannabinol* or ea 1477 or ea1477 or marinol or qcd 84924 or syndros or tetrabinex or tetranabinex).tw,kf. (24944)

9 (cesamet or nabilone).tw,kf. (980)

10 (deltanyne or "abbott 40566" or namisol or dronabinolum or "QCD 84924" or "CCRIS 4726").tw,kf. (27)

11 (nabiximol? or "gw 1000" or gw1000 or "sab 378" or sab378 or sativex).tw,kf. (1051)

12 (13956-29-1 or 19GBJ60SN5 or UNII-19GBJ60SN5 or ZYN002).rn,nm. (4792)

13 or/1-12 [CANNABIS] (170440)

14 exp Animals/ not (exp Animals/ and Humans/) (18644981)

15 13 not 14 [ANIMAL-ONLY REMOVED] (107460)

16 (comment or editorial or news or newspaper article).pt. (1925333)

17 (letter not (letter and randomized controlled trial)).pt. (2095036)

18 (case reports not (meta analysis or systematic review or controlled clinical trial or randomized controlled trial or pragmatic clinical trial or comparative study or observational study)).pt. (2004170)

19 (case report* or case study or case studies).ti. not (meta analysis or systematic review or controlled clinical trial or randomized controlled trial or pragmatic clinical trial or comparative study or observational study).pt. (663964)

20 15 not (16 or 17 or 18 or 19) [OPINION PIECES AND CASE REPORTS REMOVED] (100764)

21 limit 20 to yr="2000-current" (73265)

22 limit 21 to systematic reviews [Limit not valid in Embase; records were retained] (27344)

23 meta analysis.pt. (101916)

24 exp meta-analysis as topic/ (57777)

25 (meta-analy* or metanaly* or metaanaly* or met analy* or integrative research or integrative review* or integrative overview* or research integration or research overview* or collaborative review*).tw,kf. (390259)

26 systematic review.pt. (108141)

27 (systematic review* or systematic overview* or evidence-based review* or evidence-based overview* or (evidence adj3 (review* or overview*)) or meta-review* or meta-overview* or meta-synthes* or "review of reviews" or technology assessment* or HTA or HTAs).tw,kf. (463297)

28 exp Technology assessment, biomedical/ (24286)

29 (cochrane or health technology assessment or evidence report).jw. (38400)

30 (network adj (MA or MAs)).tw,kf. (22)

31 (NMA or NMAs).tw,kf. (4843)

32 indirect* compar*.tw,kf. (5076)

33 (indirect treatment* adj1 compar*).tw,kf. (743)

34 (mixed treatment* adj1 compar*).tw,kf. (1324)

35 (multiple treatment* adj1 compar*).tw,kf. (373)

36 (multi-treatment* adj1 compar*).tw,kf. (5)

37 simultaneous* compar*.tw,kf. (2468)

38 mixed comparison?.tw,kf. (69)

39 or/23-38 (800271)

40 21 and 39 (1997)

41 22 or 40 [SRs/MAs] (27946)

42 (controlled clinical trial or randomized controlled trial or pragmatic clinical trial).pt. (572724)

43 clinical trials as topic.sh. (187311)

44 exp Randomized Controlled Trials as Topic/ (288536)

45 (randomi#ed or randomly or RCT or placebo*).tw,kf. (2335285)

46 ((singl* or doubl* or trebl* or tripl*) adj (mask* or blind* or dumm*)).tw,kf. (420362)

47 trial.ti. (508136)

48 or/42-47 (2954693)

49 21 and 48 [RCTS] (5692)

50 controlled clinical trial.pt. (93114)

51 Controlled Clinical Trial/ or Controlled Clinical Trials as Topic/ (570179)

52 (control* adj2 trial*).tw,kf. (617028)

53 Non-Randomized Controlled Trials as Topic/ (10634)

54 (nonrandom* or non-random* or quasi-random* or quasi-experiment*).tw,kf. (132446)

55 (nRCT or nRCT or non-RCT).tw,kf. (711)

56 Controlled Before-After Studies/ (214315)

57 (control* adj3 ("before and after" or "before after")).tw,kf. (10168)

58 Interrupted Time Series Analysis/ (206526)

59 time series.tw,kf. (66342)

60 (pre- adj3 post-).tw,kf. (235965)

61 (pretest adj3 posttest).tw,kf. (18126)

62 Historically Controlled Study/ (224687)

63 (control* adj2 stud$3).tw,kf. (541147)

64 Control Groups/ (125964)

65 (control* adj2 group$1).tw,kf. (1235725)

66 trial.ti. (508136)

67 or/50-66 (3429145)

68 21 and 67 [NON-RCTS] (5253)

69 exp Cohort Studies/ (2339324)

70 cohort?.tw,kf. (1463046)

71 Retrospective Studies/ (1167471)

72 (longitudinal or prospective or retrospective).tw,kf. (3107066)

73 ((followup or follow-up) adj (study or studies)).tw,kf. (130207)

74 Observational study.pt. (63067)

75 (observation$2 adj (study or studies)).tw,kf. (252334)

76 ((population or population-based) adj (study or studies or analys#s)).tw,kf. (40939)

77 ((multidimensional or multi-dimensional) adj (study or studies)).tw,kf. (371)

78 Comparative Study.pt. (1832457)

79 ((comparative or comparison) adj (study or studies)).tw,kf. (263158)

80 exp Case-Control Studies/ (1157656)

81 ((case-control* or case-based or case-comparison) adj (study or studies)).tw,kf. (233312)

82 Cross-Sectional Studies/ (471160)

83 (cross-section* or crosssection*).tw,kf. (872886)

84 or/69-83 (8136354)

85 21 and 84 [OBSERVATIONAL STUDIES] (16576)

86 Qualitative Research/ (119321)

87 Interview/ (220855)

88 interview*.mp. (1208572)

89 (theme* or thematic).mp. (343177)

90 qualitative.af. (890060)

91 Nursing Methodology Research/ (30884)

92 questionnaire*.mp. (1946849)

93 ethnological research.mp. (29)

94 ethnograph*.mp. (49249)

95 ethnonursing.af. (363)

96 phenomenol*.af. (165044)

97 (grounded adj (theor* or study or studies or research or analys#s)).af. (78152)

98 (life stor* or women* stor*).mp. (6741)

99 (emic or etic or hermeneutic* or heuristic* or semiotic*).af. or (data adj1 saturat*).tw. or participant observ*.tw. (141511)

100 (social construct* or (postmodern* or post-structural*) or (post structural* or poststructural*) or post modern* or post-modern* or feminis* or interpret*).mp. (1234791)

101 (action research or cooperative inquir* or co operative inquir* or co-operative inquir*).mp. (17587)

102 (humanistic or existential or experiential or paradigm*).mp. (453439)

103 (field adj (study or studies or research)).tw. (43618)

104 human science.tw. (1162)

105 biographical method.tw. (103)

106 theoretical sampl*.af. (2385)

107 ((purpos* adj4 sampl*) or (focus adj group*)).af. (187764)

108 (account or accounts or unstructured or open-ended or open ended or text* or narrative*).mp. (1692021)

109 (life world or life-world or conversation analys#s or personal experience* or theoretical saturation).mp. (83469)

110 ((lived or life) adj experience*).mp. (67595)

111 observational method*.af. (4965)

112 content analys#s.af. (111622)

113 (constant adj (comparative or comparison)).af. (14797)

114 ((discourse* or discurs*) adj3 analys#s).tw. (13048)

115 narrative analys#s.af. (9134)

116 (heidegger* or colaizzi* or spiegelberg* or van manen* or van kaam* or merleau ponty* or husserl* or foucault* or (corbin adj2 strauss*) or glaser*).tw. (17479)

117 mixed method*.tw,kf. (59394)

118 or/86-117 (6731223)

119 21 and 118 [QUALITATIVE STUDIES] (18948)

120 41 or 49 or 68 or 85 or 119 [ALL STUDY DESIGNS] (51617)

121 120 use medall [MEDLINE RECORDS] (15296)

122 cannabis/ (47495)

123 exp cannabinoid/ (69101)

124 cannabis addiction/ (9169)

125 exp "cannabis use"/ (9828)

126 cannabis addiction/ (9169)

127 cannabis sativa/ (8710)

128 ("c.indica" or "c.sativa" or cannabi* or bhang or cannador or cbd or charas or eucannabinolide* or ganja or ganjah or hash or hashish or hemp or marihuana* or marijuana*).tw,kw. (137428)

129 (epidiolex or gwp 42003p or gwp42003p or nabidiolex).tw,kw. (165)

130 (dronabinol or thc or tetrahydrocannabinol* or ea 1477 or ea1477 or marinol or qcd 84924 or syndros or tetrabinex or tetranabinex).tw,kw. (25257)

131 (cesamet or nabilone).tw,kw. (994)

132 (deltanyne or "abbott 40566" or namisol or dronabinolum or "QCD 84924" or "CCRIS 4726").tw,kw. (27)

133 (nabiximol? or "gw 1000" or gw1000 or "sab 378" or sab378 or sativex).tw,kw. (1065)

134 (13956-29-1 or 19GBJ60SN5 or UNII-19GBJ60SN5 or ZYN002).rn. (4792)

135 or/122-134 [CANNABIS] (170185)

136 exp animal/ or exp animal experimentation/ or exp animal model/ or exp animal experiment/ or nonhuman/ or exp vertebrate/ (50781287)

137 exp human/ or exp human experimentation/ or exp human experiment/ (38859218)

138 136 not 137 (11923763)

139 135 not 138 [ANIMAL-ONLY REMOVED] (137736)

140 editorial.pt. (1097437)

141 letter.pt. not (letter.pt. and randomized controlled trial/) (2089965)

142 (case report* or case study or case studies).ti. not (meta-analysis/ or "systematic review"/ or randomized controlled trial/ or controlled clinical trial/ or controlled study/ or time series analysis/ or cohort analysis/ or retrospective study/ or longitudinal study/ or prospective study/ or exp comparative study/ or observational study/ or exp case control study/ or cross-sectional study/) (647156)

143 conference abstract.pt. (3431216)

144 139 not (140 or 141 or 142 or 143) [OPINION PIECES, CASE REPORTS AND CONFERENCE ABSTRACTS REMOVED] (119477)

145 limit 144 to yr="2000-current" (92411)

146 meta-analysis/ (270414)

147 "systematic review"/ (315194)

148 "meta analysis (topic)"/ (39946)

149 (meta-analy* or metanaly* or metaanaly* or met analy* or integrative research or integrative review* or integrative overview* or research integration or research overview* or collaborative review*).tw,kw. (393194)

150 (systematic review* or systematic overview* or evidence-based review* or evidence-based overview* or (evidence adj3 (review* or overview*)) or meta-review* or meta-overview* or meta-synthes* or "review of reviews" or technology assessment* or HTA or HTAs).tw,kw. (466673)

151 biomedical technology assessment/ (23175)

152 (cochrane or health technology assessment or evidence report).jw. (38400)

153 (network adj (MA or MAs)).tw,kw. (22)

154 (NMA or NMAs).tw,kw. (4861)

155 indirect* compar*.tw,kw. (5142)

156 (indirect treatment* adj1 compar*).tw,kw. (747)

157 (mixed treatment* adj1 compar*).tw,kw. (1348)

158 (multiple treatment* adj1 compar*).tw,kw. (379)

159 (multi-treatment* adj1 compar*).tw,kw. (5)

160 simultaneous* compar*.tw,kw. (2468)

161 mixed comparison?.tw,kw. (70)

162 or/146-161 (866448)

163 145 and 162 [REVIEWS] (3255)

164 randomized controlled trial/ or controlled clinical trial/ (1312582)

165 "clinical trial (topic)"/ (101842)

166 "randomized controlled trial (topic)"/ (161564)

167 (randomi#ed or randomly or RCT or placebo*).tw,kw. (2337327)

168 ((singl* or doubl* or trebl* or tripl*) adj (mask* or blind* or dumm*)).tw,kw. (420518)

169 trial.ti. (508136)

170 or/164-169 (3097657)

171 145 and 170 [RCTS] (8045)

172 controlled clinical trial/ (556331)

173 "controlled clinical trial (topic)"/ (10130)

174 (control* adj2 trial*).tw,kw. (621025)

175 (nonrandom* or non-random* or quasi-random* or quasi-experiment*).tw,kw. (132673)

176 (nRCT or nRCT or non-RCT).tw,kw. (712)

177 (control* adj3 ("before and after" or "before after")).tw,kw. (10173)

178 time series analysis/ (23336)

179 time series.tw,kw. (67117)

180 pretest posttest control group design/ (388)

181 (pre- adj3 post-).tw,kw. (235993)

182 (pretest adj3 posttest).tw,kw. (18129)

183 controlled study/ (6716025)

184 (control* adj2 stud$3).tw,kw. (542644)

185 control group/ (125964)

186 (control* adj2 group$1).tw,kw. (1235490)

187 trial.ti. (508136)

188 or/172-187 (8859133)

189 145 and 188 [NON-RCTS] (17011)

190 cohort analysis/ (715040)

191 cohort?.tw,kw. (1465324)

192 retrospective study/ (1538399)

193 longitudinal study/ (251196)

194 prospective study/ (1032457)

195 (longitudinal or prospective or retrospective).tw,kw. (3112453)

196 follow up/ (1449056)

197 ((followup or follow-up) adj (study or studies)).tw,kw. (132022)

198 observational study/ (232318)

199 (observation$2 adj (study or studies)).tw,kw. (252950)

200 population research/ (99977)

201 ((population or population-based) adj (study or studies or analys#s)).tw,kw. (48961)

202 ((multidimensional or multi-dimensional) adj (study or studies)).tw,kw. (372)

203 exp comparative study/ (3195620)

204 ((comparative or comparison) adj (study or studies)).tw,kw. (261567)

205 exp case control study/ (1157656)

206 ((case-control* or case-based or case-comparison) adj (study or studies)).tw,kw. (234775)

207 cross-sectional study/ (598732)

208 (cross-section* or crosssection*).tw,kw. (874926)

209 or/190-208 (10061900)

210 145 and 209 [OBSERVATIONAL STUDIES] (23802)

211 exp qualitative research/ (125164)

212 exp interview/ (285958)

213 interview*.mp. (1208572)

214 (theme* or thematic).mp. (343177)

215 qualitative.af. (890060)

216 nursing methodology research/ (30884)

217 questionnaire*.mp. (1946849)

218 ethnological research.mp. (29)

219 ethnograph*.mp. (49249)

220 ethnonursing.af. (363)

221 phenomenol*.af. (165044)

222 (grounded adj (theor* or study or studies or research or analys#s)).af. (78152)

223 (life stor* or women* stor*).mp. (6741)

224 (emic or etic or hermeneutic* or heuristic* or semiotic*).af. or (data adj1 saturat*).tw. or participant observ*.tw. (141511)

225 (social construct* or (postmodern* or post-structural*) or (post structural* or poststructural*) or post modern* or post-modern* or feminis* or interpret*).mp. (1234791)

226 (action research or cooperative inquir* or co operative inquir* or co-operative inquir*).mp. (17587)

227 (humanistic or existential or experiential or paradigm*).mp. (453439)

228 (field adj (study or studies or research)).tw. (43618)

229 human science.tw. (1162)

230 biographical method.tw. (103)

231 theoretical sampl*.af. (2385)

232 ((purpos* adj4 sampl*) or (focus adj group*)).af. (187764)

233 (account or accounts or unstructured or open-ended or open ended or text* or narrative*).mp. (1692021)

234 (life world or life-world or conversation analys#s or personal experience* or theoretical saturation).mp. (83469)

235 ((lived or life) adj experience*).mp. (67595)

236 observational method*.af. (4965)

237 content analys#s.af. (111622)

238 (constant adj (comparative or comparison)).af. (14797)

239 ((discourse* or discurs*) adj3 analys#s).tw. (13048)

240 narrative analys#s.af. (9134)

241 (heidegger* or colaizzi* or spiegelberg* or van manen* or van kaam* or merleau ponty* or husserl* or foucault* or (corbin adj2 strauss*) or glaser*).tw. (17479)

242 mixed method*.tw,kw. (59821)

243 or/211-242 (6740916)

244 145 and 243 [QUALITATIVE STUDIES] (23312)

245 163 or 171 or 189 or 210 or 244 [ALL STUDY DESIGNS] (50235)

246 245 use emczd [EMBASE RECORDS] (25234)

247 exp Cannabis/ (50180)

248 exp Cannabinoids/ (82168)

249 Marijuana Usage/ (2717)

250 ("c.indica" or "c.sativa" or cannabi* or bhang or cannador or cbd or charas or eucannabinolide* or ganja or ganjah or hash or hashish or hemp or marihuana* or marijuana*).tw. (135770)

251 (epidiolex or gwp 42003p or gwp42003p or nabidiolex).tw. (164)

252 (dronabinol or thc or tetrahydrocannabinol* or ea 1477 or ea1477 or marinol or qcd 84924 or syndros or tetrabinex or tetranabinex).tw. (24781)

253 (cesamet or nabilone).tw. (976)

254 (deltanyne or "abbott 40566" or namisol or dronabinolum or "QCD 84924" or "CCRIS 4726").tw. (26)

255 (nabiximol? or "gw 1000" or gw1000 or "sab 378" or sab378 or sativex).tw. (1040)

256 or/247-255 [CANNABIS] (167483)

257 limit 256 to yr="2000-current" (130969)

258 limit 257 to ("0830%2509%2509systematic review" or 1200 meta analysis or 1300 metasynthesis) [Limit not valid in Embase,Ovid MEDLINE(R),Ovid MEDLINE(R) Daily Update,Ovid MEDLINE(R) In-Process,Ovid MEDLINE(R) Publisher,PsycINFO; records were retained] (111558)

259 meta analysis/ (270414)

260 (meta-analy* or metanaly* or metaanaly* or met analy* or integrative research or integrative review* or integrative overview* or research integration or research overview* or collaborative review*).tw. (389001)

261 "systematic review"/ (315194)

262 (systematic review* or systematic overview* or evidence-based review* or evidence-based overview* or (evidence adj3 (review* or overview*)) or meta-review* or meta-overview* or meta-synthes* or "review of reviews" or technology assessment* or HTA or HTAs).tw. (461511)

263 (network adj (MA or MAs)).tw. (22)

264 (NMA or NMAs).tw. (4828)

265 indirect* compar*.tw. (5054)

266 (indirect treatment* adj1 compar*).tw. (725)

267 (mixed treatment* adj1 compar*).tw. (1268)

268 (multiple treatment* adj1 compar*).tw. (360)

269 (multi-treatment* adj1 compar*).tw. (5)

270 simultaneous* compar*.tw. (2468)

271 mixed comparison?.tw. (69)

272 or/259-271 (812071)

273 257 and 272 (3419)

274 258 or 273 [REVIEWS] (111954)

275 limit 257 to "0300 clinical trial" [Limit not valid in Embase,Ovid MEDLINE(R),Ovid MEDLINE(R) Daily Update,Ovid MEDLINE(R) In-Process,Ovid MEDLINE(R) Publisher; records were retained] (111684)

276 exp clinical trials/ (307191)

277 (randomi#ed or randomly or RCT or placebo*).tw. (2333296)

278 ((singl* or doubl* or trebl* or tripl*) adj (mask* or blind* or dumm*)).tw. (420266)

279 trial.ti. (508136)

280 or/276-279 (2731009)

281 257 and 280 (9190)

282 275 or 281 [RCTS] (112937)

283 (control* adj2 trial*).tw. (614562)

284 (nonrandom* or non-random* or quasi-random* or quasi-experiment*).tw. (132293)

285 (nRCT or nRCT or non-RCT).tw. (710)

286 (control* adj3 ("before and after" or "before after")).tw. (10162)

287 time series/ (23364)

288 time series.tw. (65885)

289 (pre- adj3 post-).tw. (235875)

290 (pretest adj3 posttest).tw. (18120)

291 (control* adj2 stud$3).tw. (540155)

292 experiment controls/ (907)

293 (control* adj2 group$1).tw. (1235372)

294 trial.ti. (508136)

295 or/283-294 (2834369)

296 257 and 295 [NON-RCTS] (7787)

297 limit 257 to ("0430 followup study" or "0450 longitudinal study" or "0451 prospective study" or "0453 retrospective study") [Limit not valid in Embase,Ovid MEDLINE(R),Ovid MEDLINE(R) Daily Update,Ovid MEDLINE(R) In-Process,Ovid MEDLINE(R) Publisher; records were retained] (113949)

298 cohort?.tw. (1461117)

299 exp longitudinal studies/ (267627)

300 retrospective studies/ (1167471)

301 (longitudinal or prospective or retrospective).tw. (3102128)

302 followup studies/ (628049)

303 ((followup or follow-up) adj (study or studies)).tw. (128850)

304 exp observation methods/ (5724)

305 (observation$2 adj (study or studies)).tw. (251563)

306 ((population or population-based) adj (study or studies or analys#s)).tw. (40399)

307 ((multidimensional or multi-dimensional) adj (study or studies)).tw. (371)

308 ((comparative or comparison) adj (study or studies)).tw. (258389)

309 ((case-control* or case-based or case-comparison) adj (study or studies)).tw. (232398)

310 (cross-section* or crosssection*).tw. (871894)

311 or/298-310 (6231761)

312 257 and 311 (21179)

313 297 or 312 [OBSERVATIONAL STUDIES] (116207)

314 interview*.mp. (1208572)

315 thematic analysis/ (12841)

316 qualitative.af. (890060)

317 questionnaire*.mp. (1946849)

318 ethnological research.mp. (29)

319 ethnograph*.mp. (49249)

320 ethnonursing.af. (363)

321 phenomenol*.af. (165044)

322 grounded theory/ (10857)

323 (grounded adj (theor* or study or studies or research or analys#s)).af. (78152)

324 exp life experiences/ (51775)

325 (life stor* or women* stor*).mp. (6741)

326 (emic or etic or hermeneutic* or heuristic* or semiotic*).af. or (data adj1 saturat*).tw. or participant observ*.tw. (141511)

327 (social construct* or (postmodern* or post-structural*) or (post structural* or poststructural*) or post modern* or post-modern* or feminis* or interpret*).mp. (1234791)

328 (action research or cooperative inquir* or co operative inquir* or co-operative inquir*).mp. (17587)

329 (humanistic or existential or experiential or paradigm*).mp. (453439)

330 (field adj (study or studies or research)).tw. (43618)

331 human science.tw. (1162)

332 biographical method.tw. (103)

333 theoretical sampl*.af. (2385)

334 ((purpos* adj4 sampl*) or (focus adj group*)).af. (187764)

335 (account or accounts or unstructured or open-ended or open ended or text* or narrative*).mp. (1692021)

336 (life world or life-world or conversation analys#s or personal experience* or theoretical saturation).mp. (83469)

337 ((lived or life) adj experience*).mp. (67595)

338 observational method*.af. (4965)

339 content analys#s.af. (111622)

340 (constant adj (comparative or comparison)).af. (14797)

341 ((discourse* or discurs*) adj3 analys#s).tw. (13048)

342 narrative analys#s.af. (9134)

343 (heidegger* or colaizzi* or spiegelberg* or van manen* or van kaam* or merleau ponty* or husserl* or foucault* or (corbin adj2 strauss*) or glaser*).tw. (17479)

344 mixed method*.tw. (59048)

345 or/314-344 (6637919)

346 257 and 345 [QUALITATIVE STUDIES] (26711)

347 274 or 282 or 296 or 313 or 346 [ALL STUDY DESIGNS] (122429)

348 347 use medall,emczd (111433)

349 347 not 348 [PSYCINFO RECORDS] (10996)

350 121 or 246 or 349 [ALL STUDY DESIGNS - ALL DATABASES] (51526)

351 41 use medall [MEDLINE REVIEWS] (1328)

352 163 use emczd [EMBASE REVIEWS] (1765)

353 274 use medall,emczd (111433)

354 274 not 353 [PSYCINFO REVIEWS] (521)

355 351 or 352 or 354 [REVIEWS - ALL DATABASES] (3614)

356 remove duplicates from 355 (2316)

357 356 use medall [MEDLINE UNIQUE REVIEWS] (1314)

358 356 use emczd [EMBASE UNIQUE REVIEWS] (853)

359 356 not (357 or 358) [PSYCINFO UNIQUE REVIEWS] (149)

360 49 use medall [MEDLINE RCTS] (2763)

361 171 use emczd [EMBASE RCTS] (4106)

362 282 use medall,emczd (111433)

363 282 not 362 [PSYCINFO RCTS] (1504)

364 360 or 361 or 363 [RCTS - ALL DATABASES] (8373)

365 limit 364 to yr="2012-current" (4980)

366 remove duplicates from 365 (2954)

367 364 not 365 (3393)

368 remove duplicates from 367 (2013)

369 366 or 368 [TOTAL UNIQUE RCTS] (4967)

370 369 use medall [MEDLINE UNIQUE RCTS] (2750)

371 369 use emczd [EMBASE UNIQUE RCTS] (1882)

372 369 not (370 or 371) [PSYCINFO UNIQUE RCTS] (335)

373 68 use medall [MEDLINE NRCTS] (2158)

374 189 use emczd [EMBASE NRCTS] (13618)

375 296 use medall,emczd (6500)

376 296 not 375 [PSYCINFO NRCTS] (1287)

377 373 or 374 or 376 [NRCTS - ALL DATABASES] (17063)

378 85 use medall [MEDLINE OBSERVATIONAL STUDIES] (9035)

379 210 use emczd [EMBASE OBSERVATIONAL STUDIES] (11319)

380 313 use medall,emczd (111433)

381 313 not 380 [PSYCINFO OBSERVATIONAL STUDIES] (4774)

382 378 or 379 or 381 [OBSERVATIONAL STUDIES - ALL DATABASES] (25128)

383 377 or 382 [NRCTS, OBSERVATIONAL STUDIES - ALL DATABASES] (35913)

384 limit 383 to yr="2018-current" (5256)

385 remove duplicates from 384 (3491)

386 limit 383 to yr="2016-2017" (5807)

387 remove duplicates from 386 (3556)

388 limit 383 to yr="2014-2015" (5231)

389 remove duplicates from 388 (3216)

390 limit 383 to yr="2012-2013" (4289)

391 remove duplicates from 390 (2631)

392 limit 383 to yr="2009-2011" (5305)

393 remove duplicates from 392 (3349)

394 limit 383 to yr="2005-2008" (5699)

395 remove duplicates from 394 (3749)

396 limit 383 to yr="2000-2004" (4326)

397 remove duplicates from 396 (2919)

398 385 or 387 or 389 or 391 or 393 or 395 or 397 [TOTAL UNIQUE NRCTS, OBSERVATIONAL STUDIES] (22911)

399 398 use medall [MEDLINE UNIQUE NRCTS, OBSERVATIONAL STUDIES] (10251)

400 398 use emczd [EMBASE UNIQUE NRCTS, OBSERVATIONAL STUDIES] (11254)

401 398 not (399 or 400) [PSYCINFO UNIQUE NRCTS, OBSERVATIONAL STUDIES] (1406)

402 119 use medall [MEDLINE QUALITATIVE STUDIES] (6904)

403 244 use emczd [EMBASE QUALITATIVE STUDIES] (9063)

404 346 use medall,emczd (18972)

405 346 not 404 [PSYCINFO QUALITATIVE STUDIES] (7739)

406 402 or 403 or 405 [QUALITATIVE STUDIES - ALL DATABASES] (23706)

407 limit 406 to yr="2017-current" (4900)

408 remove duplicates from 407 (3033)

409 limit 406 to yr="2014-2016" (5464)

410 remove duplicates from 409 (3388)

411 limit 406 to yr="2010-2013" (5532)

412 remove duplicates from 411 (3350)

413 limit 406 to yr="2005-2009" (4995)

414 remove duplicates from 413 (3056)

415 limit 406 to yr="2000-2004" (2816)

416 remove duplicates from 415 (1737)

417 408 or 410 or 412 or 414 or 416 [TOTAL UNIQUE QUALITATIVE STUDIES - ALL DATABASES] (14563)

418 417 use medall [MEDLINE UNIQUE QUALITATIVE STUDIES] (6877)

419 417 use emczd [EMBASE UNIQUE QUALITATIVE STUDIES] (3597)

420 417 not (418 or 419) [PSYCINFO UNIQUE QUALITATIVE STUDIES] (4089)

421 366 or 368 (4967)

422 remove duplicates from 421 (4964)

423 422 use medall (2749)

424 422 use emczd (1880)

425 422 not (423 or 424) (335)

***************************

Cochrane

Search Name: Cannabis - Final

Date Run: 14/06/2019 13:59:36

ID Search Hits

#1 [mh Cannabis] 290

#2 [mh Cannabinoids] 731

#3 [mh "Marijuana Abuse"] 524

#4 [mh "Marijuana Use"] 284

#5 [mh "Marijuana Smoking"] 276

#6 ("c.indica" or "c.sativa" or cannabi* or bhang or cannador or cbd or charas or eucannabinolide* or ganja or ganjah or hash or hashish or hemp or marihuana* or marijuana*):ti,ab,kw 4028

#7 (epidiolex or gwp 42003p or gwp42003p or nabidiolex):ti,ab,kw 30

#8 (dronabinol or thc or tetrahydrocannabinol* or ea 1477 or ea1477 or marinol or qcd 84924 or syndros or tetrabinex or tetranabinex):ti,ab,kw 1387

#9 (cesamet or nabilone):ti,ab,kw 142

#10 (deltanyne or "abbott 40566" or namisol or dronabinolum or "QCD 84924" or "CCRIS 4726"):ti,ab,kw 16

#11 (nabiximol* or "gw 1000" or gw1000 or "sab 378" or sab378 or sativex):ti,ab,kw 167

#12 {or #1-#11} with Publication Year from 2000 to 2019, in Trials 3638

#13 {or #1-#11} in Cochrane Reviews, Cochrane Protocols 45

#14 #12 OR #13 3683

Reviews – 42

Protocols – 3

Trials – 3638
