# Supplementary material for: Impacts of medical and non-medical cannabis on the health of older adults: Findings from a scoping review of the literature
Source: PLoS One. 2023 Feb 17;18(2):e0281826. doi: 10.1371/journal.pone.0281826 (PMC9937508; doi:10.1371/journal.pone.0281826)
Supplement: S5 Text — (DOCX) [file pone.0281826.s008.docx]

S5: Studies Excluded at Full-text Screening, by Reason

Publications excluded during full-text screening have been reported below, stratified by the study design of the search in which they were found and by reason for exclusion. The designs of some studies may have been misclassified within the original literature databases and so may appear under the incorrect design below.

Systematic reviews and overviews of reviews

Not published in English or French

Al-Khalil, O. Cannabinoids in medical procedures - Review and meta-analysis. Praxis 2015. 104 (20) 1103-1104.

Arias, Ruth, Alberich, Susana, Zorrilla, Inaki, Gonzalez-Pinto, Ana. Impact of cannabis use on the progression of bipolar disorder. Psiquiatria Biologica 2020. 27 (2) 54-60.

Bugra, H., Rapp, C., Studerus, E., Aston, J., Borgwardt, S., and Riecher-Rossler, A. [Can cannabis use increase the risk for schizophrenic psychoses?]. Fortschritte der Neurologie-Psychiatrie 2012. 80 (11) 635-643.

Hoch, Eva, Friemel, Chris, Schneider, Miriam, Pogarell, Oliver, Hasan, Alkomiet, Preuss, Ulrich W., Ca, PRis Projektgruppe. [Efficacy and safety of medicinal cannabis: results of the CaPRis study]. Wirksamkeit und Sicherheit von Cannabisarzneimitteln: Ergebnisse der CaPRis-Studie. 2019. 62 (7) 825-829.

Jonsson, Arnar Jan, Birgisdottir, Hera, and Sigurdsson, Engilbert. [Does the use of cannabis increase the risk for psychosis and the development of schizophrenia?]. Laeknabladid 2014. 100 (9) 443-451.

Olcina Rodriguez, Jose, Lopez Briz, Eduardo, and Olcina Dominguez, Pablo. [Cardiovascular safety of the recreational use of cannabis associated to sildenafil: Systematic review]. Revista internacional de andrologia 2019.

Rojas-Jara, Claudio, Polanco-Carrasco, Roberto, Cisterna, Alejandra, Hernandez, Vanesa, Miranda, Fernanda, Moreno, Andrea, Alarcon, Luis. Medicinal use of cannabis: A review of the evidence. Terapia Psicologica 2019. 37 (2) 166-180.

Conference abstract, letter, commentary, or other non-relevant design

Anonymous. Cannabinoids not very effective for chronic non-cancer pain. Drug and Therapeutics Bulletin 2019. 57 (11) 165.

Anonymous. Medical cannabis for psychiatric disorders: A systematic review. Revue Medicale Suisse 2020. 16 (695) 1124.

Anonymous. Psychiatric Effects of Cannabis Use. The American journal of nursing 2020. 120 (7) 18.

Ayati, Zahra, Sarris, Jerome, Chang, Dennis, Emami, Seyed A., Rahimi, Roja. Herbal medicines and phytochemicals for obsessive-compulsive disorder. Phytotherapy research : PTR 2020. 34 (8) 1889-1901.

Berger, Amnon A., Keefe, Joseph, Winnick, Ariel, Gilbert, Elasaf, Eskander, Jonathan P., Yazdi, Cyrus, Kaye, Alan D., Viswanath, Omar, Urits, Ivan. Cannabis and cannabidiol (CBD) for the treatment of fibromyalgia. Best practice & research. Clinical anaesthesiology 2020. 34 (3) 617-631.

Black, Nicola, Campbell, Gabrielle, Tran, Lucy T., Farrell, Michael, Hall, Wayne, Degenhardt, Louisa. "Cannabinoids for the treatment of mental disorders": Author's reply. The Lancet Psychiatry 2020. 7 (2) 127-128.

Branas, P., Jordan, R., Fry-Smith, A., Burls, A., and Hyde, C. Treatments for fatigue in multiple sclerosis: a rapid and systematic review. Health technology assessment (Winchester, England) 2000. 4 (27) 1-61.

Campos, Alline C., Guimaraes, Francisco S., Ferreira-Junior, Nilson C., Del-Bel, Elaine, Zimmermann, Patricia M. da R., Brum Junior, Liberato, Hallak, Jaime E., Crippa, Jose A., Zuardi, Antonio W. Biological bases for a possible effect of cannabidiol in parkinson's disease. Brazilian Journal of Psychiatry 2020. 42 (2) 218-224.

Cannabinoids have limited impact on MS symptoms. Clinical Pharmacist 2019. 11 (1).

Cannabis derivatives and pain. Prescrire International 2009. 18 (103) 226.

Corliss, M., Zhang, Y., Broadbent, J., and Salazar-Grueso, E. A meta-analysis of spasticity and pain: implications for botulinum toxin treatment. Pain medicine (Malden, Mass.) 2009. 10 (1) 273‐.

Correia-Sa, Ines, Paiva, Anita, Carvalho, Claudia M., Vieira-Coelho, Maria A. Cutaneous endocannabinoid system: Does it have a role on skin wound healing bearing fibrosis?. Pharmacological Research 2020. 159, 104862.

Davis, M. P. Systematic review of adverse effects of medical cannabinoids. Journal of Pain and Palliative Care Pharmacotherapy 2008. 22 (4) 316-317.

Davis, Mellar P. Oral nabilone capsules in the treatment of chemotherapy-induced nausea and vomiting and pain. Expert Opinion on Investigational Drugs 2008. 17 (1) 85-95.

Farkhondeh, Tahereh, Khan, Haroon, Aschner, Michael, Samini, Fariborz, Pourbagher-Shahri, Ali M., Aramjoo, Hamed, Roshanravan, Babak, Hoyte, Christopher, Mehrpour, Omid, Samarghandian, Saeed. Impact of Cannabis-Based Medicine on Alzheimer's Disease by Focusing on the Amyloid beta-Modifications: A Systematic Study. CNS & neurological disorders drug targets 2020. 19 (5) 334-343.

Graham, Myfanwy, Lucas, Catherine J., Schneider, Jennifer, Martin, Jennifer H., Hall, Wayne. Translational hurdles with cannabis medicines. Pharmacoepidemiology and Drug Safety 2020.

Gray, Roland W. IS MARIJUANA MEDICINE?. Tennessee medicine : journal of the Tennessee Medical Association 2016. 109 (3) 26-28.

Jarjou'i, Amir, Izbicki, Gabriel. Medical cannabis in asthmatic patients. Israel Medical Association Journal 2020. 22 (4) 232-235.

Maher, D. P. and Cohen, S. P. Medical marijuana research for chronic pain. The Lancet Psychiatry 2017. 4 (7) 513-515.

Nathan, N. Unraveling the Mystery of THC: Cannabinoids and Neuropathic Pain. Anesthesia and analgesia 2017. 125 (5) 1428.

Oberbarnscheidt, Thersilla, Miller, Norman S. The Impact of Cannabidiol on Psychiatric and Medical Conditions. Journal of clinical medicine research 2020. 12 (7) 393-403.

Osazuwa-Peters, Nosayaba, Adjei-Boakye, Eric, Loux, Travis M., Varvares, Mark A., and Schootman, Mario. Insufficient Evidence to Support or Refute the Association between Head and Neck Cancer and Marijuana Use. The journal of evidence-based dental practice 2016. 16 (2) 127-129.

Parsai, S., Herman, R., and Johnson, S. Systematic literature review of randomized controlled trials to evaluate the efficacy of medical marijuana for analgesia. Pharmacotherapy 2014. 34 (10) e287‐.

Santibanez, R. A., Sepehry, A. A., and Hsiung, G. YR. Cannabis and Alzheimer's disease: a systematic review of the evidence. Alzheimer's and dementia.Conference: alzheimer's association international conference, AAIC 2017.United kingdom 2017. 13 (7) 614.

Schonke, Milena, Martinez-Tellez, Borja, Rensen, Patrick C. N. Role of the endocannabinoid system in the regulation of the skeletal muscle response to exercise. Current Opinion in Pharmacology 2020. 52, 52-60.

Seftel, Allen D. Re: Relationship between Cannabis Use and Erectile Dysfunction: A Systematic Review and Meta-Analysis. The Journal of urology 2020. 203 (6) 1044.

Shayesteh, Mohammad R. H., Haghi-Aminjan, Hamed, Mousavi, Mohammad J., Momtaz, Saeideh, Abdollahi, Mohammad. The Protective Mechanism of Cannabidiol in Cardiac Injury: A Systematic Review of Non-Clinical Studies. Current pharmaceutical design 2019. 25 (22) 2499-2507.

Tagne, Alex Mabou, Cosentino, Marco, Marino, Franca, Pacchetti, Barbara, Sodergren, Mikael. Cannabidiol for Viral Diseases: Hype or Hope?. Cannabis and Cannabinoid Research 2020. 5 (2) 121-131.

Erratum of reviews that were not included

Anonymous. Correction to Lancet Psychiatry 2019; 6: 995-1010 (The Lancet Psychiatry (2019) 6(12) (995-1010), (S2215036619304018), (10.1016/S2215-0366(19)30401-8)). The Lancet Psychiatry 2020. 7 (1) e3.

Anonymous. Correction: Analgesic efficacy of cannabinoids for acute pain management after surgery: a systematic review and meta-analysis. Regional anesthesia and pain medicine 2020. 45 (11) e3.

Anonymous. Erratum: Analgesic efficacy of cannabinoids for acute pain management after surgery: A systematic review and meta-analysis (Reg Anesth Pain Med (2020) 45 (509-519) DOI: 10.1136/rapm-2020-101340). Regional Anesthesia and Pain Medicine 2020. 45 (11) e3.

Anonymous. Erratum: Association between marijuana use and risk of cancer: A systematic review and meta-analysis (JAMA Network Open (2019) 2:11 (e1916318) DOI: 10.1001/jamanetworkopen.2019.16318). JAMA Network Open 2020. 3 (1) e1921065.

Black, Nicola, Stockings, Emily, Campbell, Gabrielle, Tran, Lucy T., Zagic, Dino, Hall, Wayne D., Farrell, Michael, Degenhardt, Louisa. "Cannabinoids for the treatment of mental disorders and symptoms of mental disorders: A systematic review and meta-analysis": Correction. The Lancet Psychiatry 2020. 7 (1) e3.

Hoch, E., Preuss, U. W., Ferri, M., Simon, R. Erratum: Digital interventions for problematic cannabis users in non-clinical Settings: Findings from a systematic review and meta-analysis (European Addiction Research (2016) 22 (233-242) DOI: 10.1159/000445716). European Addiction Research 2016. 22 (5).

Rogeberg, Ole, Elvik, Rune, White, Michael. Erratum: Correction to: 'The effects of cannabis intoxication on motor vehicle collision revisited and revised' (2016) (Addiction (Abingdon, England) (2016) 111 8 (1348-1359)). Addiction (Abingdon, England) 2018. 113 (5) 967-969.

Age criteria not met by any included primary study

Barkin, Jodie A., Nemeth, Zsuzsanna, Saluja, Ashok K., and Barkin, Jamie S. Cannabis-Induced Acute Pancreatitis: A Systematic Review. Pancreas 2017. 46 (8) 1035-1038.

Chinuck, R. S., Fortnum, H., and Baldwin, D. R. Appetite stimulants in cystic fibrosis: a systematic review. Journal of human nutrition and dietetics : the official journal of the British Dietetic Association 2007. 20 (6) 526-537.

Docter, Shgufta, Khan, Moin, Gohal, Chetan, Ravi, Bheeshma, Bhandari, Mohit, Gandhi, Rajiv, Leroux, Timothy. Cannabis Use and Sport: A Systematic Review. Sports health 2020. 12 (2) 189-199.

Elias, Dina, Plurad, David, Bender, Miriam. Relationship of Time of Injury Marijuana Exposure and Traumatic Brain Injury: A Systematic Review. Journal of trauma nursing : the official journal of the Society of Trauma Nurses 2020. 27 (6) 360-368.

Gurney, J., Shaw, C., Stanley, J., Signal, V., and Sarfati, D. Cannabis exposure and risk of testicular cancer: a systematic review and meta-analysis. BMC Cancer 2015. 15, 897.

Kraan, T., Velthorst, E., Koenders, L., Zwaart, K., Ising, H. K., van den Berg, D., de Haan, L., and van der Gaag, M. Cannabis use and transition to psychosis in individuals at ultra-high risk: review and meta-analysis. Psychological Medicine 2016. 46 (4) 673-681.

Lattanzi, Simona, Brigo, Francesco, Trinka, Eugen, Zaccara, Gaetano, Striano, Pasquale, Del Giovane, Cinzia, Silvestrini, Mauro. Adjunctive Cannabidiol in Patients with Dravet Syndrome: A Systematic Review and Meta-Analysis of Efficacy and Safety. CNS drugs 2020. 34 (3) 229-241.

Lisowska, Agnieszka, Makarewicz-Wujec, Magdalena, and Kozlowska-Wojciechowska, Malgorzata. Can "legal highs" trigger myocardial infarction? Patients' characteristics based on published cases. Substance use & misuse 2017. 52 (13) 1712-1720.

Lorenzetti, Valentina, Chye, Yann, Silva, Pedro, Solowij, Nadia, and Roberts, Carl A. Does regular cannabis use affect neuroanatomy? An updated systematic review and meta-analysis of structural neuroimaging studies. European Archives of Psychiatry and Clinical Neuroscience 2019. 269 (1) 59-71.

Marshall, Max and Rathbone, John. Early intervention for psychosis. The Cochrane database of systematic reviews 2011. (6) CD004718.

Pamplona, Fabricio A., da Silva, Lorenzo Rolim, and Coan, Ana Carolina. Potential Clinical Benefits of CBD-Rich Cannabis Extracts Over Purified CBD in Treatment-Resistant Epilepsy: Observational Data Meta-analysis. Frontiers in Neurology 2018. 9, 759.

Prud'homme, Melissa, Cata, Romulus, and Jutras-Aswad, Didier. Cannabidiol as an Intervention for Addictive Behaviors: A Systematic Review of the Evidence. Substance abuse : research and treatment 2015. 9, 33-38.

Rapp, Charlotte, Bugra, Hilal, Riecher-Rossler, Anita, Tamagni, Corinne, and Borgwardt, Stefan. Effects of cannabis use on human brain structure in psychosis: a systematic review combining in vivo structural neuroimaging and post mortem studies. Current Pharmaceutical Design 2012. 18 (32) 5070-5080.

Rodrigues, Larissa Alencar, Caroba, Mariana Emanuele Silva, Taba, Fernando Kengy, Filev, Renato, Gallassi, Andrea Donatti. Evaluation of the potential use of cannabidiol in the treatment of cocaine use disorder: A systematic review. Pharmacology, biochemistry, and behavior 2020. 196, 172982.

Stone, Nicole L., Murphy, Alexandra J., England, Timothy J., O'Sullivan, Saoirse E. A systematic review of minor phytocannabinoids with promising neuroprotective potential. British journal of pharmacology 2020. 177 (19) 4330-4352.

Tan, C., Hatam, N., and Treasure, T. Bullous disease of the lung and cannabis smoking: Insufficient evidence for a causative link. Journal of the Royal Society of Medicine 2006. 99 (2) 77-80.

Trinh, Kien V., Diep, Dion, and Robson, Hannah. Marijuana and Its Effects on Athletic Performance: A Systematic Review. Clinical journal of sport medicine : official journal of the Canadian Academy of Sport Medicine 2018. 28 (4) 350-357.

Underner, M., Perriot, J., Peiffer, G., Urban, T., Jaafari, N. [Acute eosinophilic pneumonia and illicit psychoactive substance use]. Pneumonies aigues a eosinophiles et usage de substances psychoactives illicites. 2020. 37 (1) 34-44.

Zarifi, Ceyda and Vyas, Shuchi. Spice-y Kidney Failure: A Case Report and Systematic Review of Acute Kidney Injury Attributable to the Use of Synthetic Cannabis. The Permanente journal 2017. 21

Age data not reported and not a review of a relevant patient condition

Abdallah, Faraj W., Hussain, Nasir, Weaver, Tristan, Brull, Richard. Analgesic efficacy of cannabinoids for acute pain management after surgery: a systematic review and meta-analysis. Regional anesthesia and pain medicine 2020. 45 (7) 509-519.

Baldinger, Reto, Katzberg, Hans Dieter, and Weber, Markus. Treatment for cramps in amyotrophic lateral sclerosis/motor neuron disease. The Cochrane database of systematic reviews 2012. (4) CD004157.

Belbasis, L., Kohler, C. A., Stefanis, N., Stubbs, B., van Os, J., Vieta, E., Seeman, M. V., Arango, C., Carvalho, A. F., and Evangelou, E. Risk factors and peripheral biomarkers for schizophrenia spectrum disorders: an umbrella review of meta-analyses. Acta Psychiatrica Scandinavica 2018. 137 (2) 88-97.

Berg, Marthe Van den, John, Mary, Black, Melissa, Semprini, Alex, Oldfield, Karen, Glass, Michelle, Braithwaite, Irene. Cannabis-based medicinal products in arthritis, a painful conundrum. The New Zealand medical journal 2020. 133 (1515) 35-45.

Blondino, Courtney T., Gormley, Mirinda Ann, Taylor, DaShaunda D. H., Lowery, Elizabeth, Clifford, James S., Burkart, Benjamin, Graves, Whitney C., Lu, Juan, Prom-Wormley, Elizabeth C. The Influence of Co-Occurring Substance Use on the Effectiveness of Opiate Treatment Programs According to Intervention Type. Epidemiologic reviews 2020. 42 (1) 57-78.

Brown, Danielle, Watson, Michael, Schloss, Janet. Pharmacological evidence of medicinal cannabis in oncology: a systematic review. Supportive Care in Cancer 2019. 27 (9) 3195-3207.

Buccelli, C., Della, Casa E., Paternoster, M., Niola, M., and Pieri, M. Gender differences in drug abuse in the forensic toxicological approach. Forensic Science International 2016. 265, 89-95.

Cademartori, Mariana G., Correa, Marcos B., Chisini, Luiz A., Conde, Marcus C. M., Francia, Alejandro, Mederos, Matias, Grazioli, Guillermo. Is the use of Cannabis associated with periodontitis? A systematic review and meta-analysis. Journal of periodontal research 2019. 54 (4) 311-317.

Cameron, Erinn C., Hemingway, Samantha L. Cannabinoids for fibromyalgia pain: A critical review of recent studies (2015-2019). Journal of Cannabis Research 2020. 2 (1) 19.

Casajuana Kogel, C., Lopez-Pelayo, H., Balcells-Olivero, M. M., Colom, J., Gual, A. Psychoactive constituents of cannabis and their clinical implications: A systematic review. Adicciones 2018. 30 (2) 140-151.

Chan, Carolyn Jessica. Efficacy of plant based cannabis in reducing pain in patients with chronic pain: A meta analysis. Dissertation Abstracts International: Section B: The Sciences and Engineering 2020. 81 (10-B) No-Specified.

Charron, Catherine B., Leung, Janice M. The Safety and Efficacy of Marijuana in Persons Living with HIV. AIDS reviews 2019. 21 (2) 84-92.

Charron, Jeremie, Carey, Vincent, Marcotte L'heureux, Viviane, Roy, Philippe, Comtois, Alain S., Ferland, Pierre-Marc. Acute effects of cannabis consumption on exercise performance: a systematic and umbrella review. The Journal of sports medicine and physical fitness 2021. 61 (4) 551-561.

Cooper, Ziva D., Abrams, Donald I. Considering abuse liability and neurocognitive effects of cannabis and cannabis-derived products when assessing analgesic efficacy: a comprehensive review of randomized-controlled studies. American Journal of Drug and Alcohol Abuse 2019. 45 (6) 580-595.

Desmarais, Anna, Smiddy, Stephen, Reddy, Sneha, El-Dallal, Mohammed, Erlich, Jonathan, Feuerstein, Joseph D. Evidence supporting the benefits of marijuana for Crohn's disease and ulcerative colitis is extremely limited: a meta-analysis of the literature. Annals of gastroenterology 2020. 33 (5) 495-499.

Dos Santos, Rafael G., Guimaraes, Francisco S., Crippa, Jose Alexandre S., Hallak, Jaime E. C., Rossi, Giordano Novak, Rocha, Juliana Mendes, Zuardi, Antonio W. Serious adverse effects of cannabidiol (CBD): a review of randomized controlled trials. Expert opinion on drug metabolism & toxicology 2020. 16 (6) 517-526.

El Abdellati, Kawtar, De Picker, Livia, Morrens, Manuel. Antipsychotic Treatment Failure: A Systematic Review on Risk Factors and Interventions for Treatment Adherence in Psychosis. Frontiers in neuroscience 2020. 14, 531763.

Els, Charl, Jackson, Tanya D., Aidoo, Henry, Wyatt, Graeme, Sowah, Daniel, Chao, Danny, Hoffman, Harold, Milen, Mathew, Straube, Sebastian, Tsuyuki, Ross T., Kunyk, Diane, Stewart-Patterson, Chris, Dick, Bruce D., Farnan, Paul. Impact of Cannabis Use on Road Traffic Collisions and Safety at Work: Systematic Review and Meta-analysis. Canadian Journal of Addiction 2019. 10 (1) 8-15.

Fischer, Benedikt, Russell, Cayley, Sabioni, Pamela, van den Brink, Wim, Le Foll, Bernard, Hall, Wayne, Rehm, Jurgen, and Room, Robin. Lower-Risk Cannabis Use Guidelines: A Comprehensive Update of Evidence and Recommendations. American journal of public health 2017. 107 (8) e1-e12.

Fitzcharles, Mary Ann, Ste-Marie, Peter A., Hauser, Winfried, Clauw, Daniel J., Jamal, Shahin, Karsh, Jacob, Landry, Tara, Leclercq, Sharon, Mcdougall, Jason J., Shir, Yoram, Shojania, Kam, and Walsh, Zach. Efficacy, Tolerability, and Safety of Cannabinoid Treatments in the Rheumatic Diseases: A Systematic Review of Randomized Controlled Trials. Arthritis care & research 2016. 68 (5) 681-688.

Hassan, Samah, Zheng, Qingping, Rizzolo, Erica, Tezcanli, Evrim, Bhardwaj, Sukriti, Cooley, Kieran. Does Integrative Medicine Reduce Prescribed Opioid Use for Chronic Pain? A Systematic Literature Review. Pain medicine (Malden, Mass.) 2020. 21 (4) 836-859.

Hindocha, C., Cousijn, J., Rall, M., Bloomfield, M. A. P. The Effectiveness of Cannabinoids in the Treatment of Posttraumatic Stress Disorder (PTSD): A Systematic Review. Journal of dual diagnosis 2020. 16 (1) 120-139.

Johal, Herman, Devji, Tahira, Chang, Yaping, Simone, Jonathan, Vannabouathong, Christopher, Bhandari, Mohit. Cannabinoids in Chronic Non-Cancer Pain: A Systematic Review and Meta-Analysis. Clinical medicine insights. Arthritis and musculoskeletal disorders 2020. 13, 1179544120906461.

Larsen, Christian, Shahinas, Jorida. Dosage, Efficacy and Safety of Cannabidiol Administration in Adults: A Systematic Review of Human Trials. Journal of clinical medicine research 2020. 12 (3) 129-141.

Lecomte, Tania, Potvin, Stephane, Samson, Crystal, Francoeur, Audrey, Hache-Labelle, Catherine, Gagne, Sarah, Boucher, Johemie, Bouchard, Marianne, Mueser, Kim T. Predicting and preventing symptom onset and relapse in schizophrenia-A metareview of current empirical evidence. Journal of abnormal psychology 2019. 128 (8) 840-854.

Lim, Sin Yin, Sharan, Satish, Woo, Sukyung. Model-Based Analysis of Cannabidiol Dose-Exposure Relationship and Bioavailability. Pharmacotherapy 2020. 40 (4) 291-300.

Macklis, Paul C., Dulmage, Brittany, Evans, Brady, Rosenbach, Misha, Gudjonsson, Johann E., Kaffenberger, Benjamin H. Cutaneous Adverse Events in Newly Approved FDA Non-cancer Drugs: A Systematic Review. Drugs in R&D 2020. 20 (3) 171-187.

Madden, Kim, George, Annie, van der Hoek, Niek J., Borim, Felipe Moreira, Mammen, George, Bhandari, Mohit. Cannabis for pain in orthopedics: a systematic review focusing on study methodology. Canadian journal of surgery. Journal canadien de chirurgie 2019. 62 (6) 369-380.

McBrien, Heather, Luo, Candice, Sanger, Nitika, Zielinski, Laura, Bhatt, Meha, Zhu, Xi Ming, Marsh, David C., Thabane, Lehana, Samaan, Zainab. Cannabis use during methadone maintenance treatment for opioid use disorder: a systematic review and meta-analysis. CMAJ open 2019. 7 (4) E665-E673.

Mohiuddin, Mohammed, Blyth, Fiona M., Degenhardt, Louisa, Di Forti, Marta, Eccleston, Christopher, Haroutounian, Simon, Moore, Andrew, Rice, Andrew S. C., Wallace, Mark, Park, Rex, Gilron, Ian. General risks of harm with cannabinoids, cannabis, and cannabis-based medicine possibly relevant to patients receiving these for pain management: an overview of systematic reviews. Pain 2020.

Moore, Theresa H. M., Zammit, Stanley, Lingford-Hughes, Anne, Barnes, Thomas R. E., Jones, Peter B., Burke, Margaret, and Lewis, Glyn. Cannabis use and risk of psychotic or affective mental health outcomes: a systematic review. Lancet (London, England) 2007. 370 (9584) 319-328.

Norton, C., Czuber-Dochan, W., Artom, M., Sweeney, L., and Hart, A. Systematic review: interventions for abdominal pain management in inflammatory bowel disease. Alimentary pharmacology & therapeutics 2017. 46 (2) 115-125.

Ogunbiyi, M. Olabisi, Hindocha, Chandni, Freeman, Tom P., Bloomfield, Michael A. P. Acute and chronic effects of DELTA9-tetrahydrocannabinol (THC) on cerebral blood flow: A systematic review. Progress in neuro-psychopharmacology & biological psychiatry 2020. 101, 109900.

Orsolini, Laura, Chiappini, Stefania, Volpe, Umberto, Berardis, Domenico De, Latini, Roberto, Papanti, Gabriele Duccio, Corkery, John, Martin. Use of Medicinal Cannabis and Synthetic Cannabinoids in Post-Traumatic Stress Disorder (PTSD): A Systematic Review. Medicina (Kaunas, Lithuania) 2019. 55 (9).

Patel, Shweta, Khan, Sahar, M, Saipavankumar, Hamid, Pousettef. The Association Between Cannabis Use and Schizophrenia: Causative or Curative? A Systematic Review. Cureus 2020. 12 (7) e9309.

Pinto, Jairo Vinicius, Saraf, Gayatri, Frysch, Christian, Vigo, Daniel, Keramatian, Kamyar, Chakrabarty, Trisha, Lam, Raymond W., Kauer-Sant'Anna, Marcia, Yatham, Lakshmi N. Cannabidiol as a Treatment for Mood Disorders: A Systematic Review. 2020. 65 (4) 213-227.

Potvin, S., Blanchet, P., and Stip, E. Substance abuse is associated with increased extrapyramidal symptoms in schizophrenia: A meta-analysis. Schizophrenia Research 2009. 113 (2-3) 181-188.

Poyatos, Lourdes, Perez-Acevedo, Ana Pilar, Papaseit, Esther, Perez-Mana, Clara, Martin, Soraya, Hladun, Olga, Siles, Adria, Torrens, Marta, Busardo, Francesco Paolo, Farre, Magi. Oral Administration of Cannabis and DELTA-9-tetrahydrocannabinol (THC) Preparations: A Systematic Review. Medicina (Kaunas, Lithuania) 2020. 56 (6).

Rosager, Emilie Vangsgaard, Moller, Christian, Sjogren, Magnus. Treatment studies with cannabinoids in anorexia nervosa: a systematic review. Eating and weight disorders : EWD 2021. 26 (2) 407-415.

Rosewall, Tara, Feuz, Carina, Bayley, Andrew. Cannabis and Radiation Therapy: A Scoping Review of Human Clinical Trials. Journal of medical imaging and radiation sciences 2020. 51 (2) 342-349.

Sankaranarayanan, Anoop, Wilding, Helen, Neill, Erica, Castle, David. A critical systematic review of evidence for cannabinoids in the treatment of schizophrenia. Psychiatric Annals 2018. 48 (5) 214-223.

Schaiquevich, Paula, Riva, Natalia, Maldonado, Cecilia, Vazquez, Marta, Caceres-Guido, Paulo. Farmacologia clinica de cannabidiol en epilepsias refractarias, Clinical pharmacology of cannabidiol in refractory epilepsy. Farmacia hospitalaria : organo oficial de expresion cientifica de la Sociedad Espanola de Farmacia Hospitalaria 2020. 44 (5) 222-229.

Shakespeare, D. T., Boggild, M., and Young, C. Anti-spasticity agents for multiple sclerosis. The Cochrane database of systematic reviews 2003. (4) CD001332.

Sharpe, Lara, Sinclair, Justin, Kramer, Andrew, De Manincor, Michael, Sarris, Jerome. Cannabis, a cause for anxiety? A critical appraisal of the anxiogenic and anxiolytic properties. Journal of Translational Medicine 2020. 18 (1) 374.

Suarez-Pinilla, Paula, Lopez-Gil, Jose, and Crespo-Facorro, Benedicto. Immune system: a possible nexus between cannabinoids and psychosis. Brain, Behavior, and Immunity 2014. 40, 269-282.

Sultan, Salahaden R., Millar, Sophie A., O'Sullivan, Saoirse E., and England, Timothy J. A Systematic Review and Meta-Analysis of the In Vivo Haemodynamic Effects of DELTA8-Tetrahydrocannabinol. Pharmaceuticals (Basel, Switzerland) 2018. 11 (1).

Taylor, C., Birch, B. Cannabinoids in Urology. Which Benign Conditions Might They Be Appropriate to Treat: A Systematic Review. Urology 2021. 148, 8-25.

Ton, Joey, Perry, Danielle, Thomas, Betsy, Allan, G. Michael, Lindblad, Adrienne J., McCormack, James, Kolber, Michael R., Garrison, Scott, Moe, Samantha, Craig, Rodger, Dugre, Nicolas, Chan, Karenn, Finley, Caitlin R., Ting, Rhonda, Korownyk, Christina S. PEER umbrella systematic review of systematic reviews: Management of osteoarthritis in primary care. Canadian family physician Medecin de famille canadien 2020. 66 (3) e89-e98.

Vaitla, Pradeep K., Thongprayoon, Charat, Hansrivijit, Panupong, Kanduri, Swetha R., Kovvuru, Karthik, Rivera, Franco H. Cabeza, Cato, Liam D., Garla, Vishnu, Watthanasuntorn, Kanramon, Wijarnpreecha, Karn, Chewcharat, Api, Aeddula, Narothama Reddy, Bathini, Tarun, Koller, Felicitas L., Matemavi, Praise, Cheungpasitporn, Wisit. Epidemiology of cannabis use and associated outcomes among kidney transplant recipients: A meta-analysis. Journal of evidence-based medicine 2020.

van der Steur, Sanne J., Batalla, Albert, Bossong, Matthijs G. Factors Moderating the Association Between Cannabis Use and Psychosis Risk: A Systematic Review. Brain sciences 2020. 10 (2).

van, Os J., Linscott, R. J., Myin-Germeys, I., Delespaul, P., and Krabbendam, L. A systematic review and meta-analysis of the psychosis continuum: Evidence for a psychosis proneness-persistence-impairment model of psychotic disorder. Psychological Medicine 2009. 39 (2) 179-195.

Vassos, Evangelos, Sham, Pak, Kempton, Matthew, Trotta, Antonella, Stilo, Simona A., Gayer-Anderson, Charlotte, Di Forti, Marta, Lewis, Cathryn M., Murray, Robin M., Morgan, Craig. The Maudsley environmental risk score for psychosis. Psychological medicine 2020. 50 (13) 2213-2220.

Wong, Eugenia, Ranapurwala, Shabbar I. Cardiovascular Risk Associated with Medical Use of Opioids and Cannabinoids: A Systematic Review. Current Cardiovascular Risk Reports 2019. 13 (10) 30.

Wong, Stanley Sau Ching, Chan, Wing Shing, Cheung, Chi Wai. Analgesic Effects of Cannabinoids for Chronic Non-cancer Pain: a Systematic Review and Meta-Analysis with Meta-Regression. Journal of neuroimmune pharmacology : the official journal of the Society on NeuroImmune Pharmacology 2020. 15 (4) 801-829.

Wrege, Johannes, Schmidt, Andre, Walter, Anna, Smieskova, Renata, Bendfeldt, Kerstin, Radue, Ernst Wilhelm, Lang, Undine E., and Borgwardt, Stefan. Effects of cannabis on impulsivity: a systematic review of neuroimaging findings. Current Pharmaceutical Design 2014. 20 (13) 2126-2137.

Yazdanian, Mohsen, Armoon, Bahram, Noroozi, Alireza, Mohammadi, Rasool, Bayat, Amir-Hosein, Ahounbar, Elahe, Higgs, Peter, Nasab, Hormoz Sanaei, Bayani, Azadeh, Hemmat, Morteza. Dental caries and periodontal disease among people who use drugs: a systematic review and meta-analysis. BMC oral health 2020. 20 (1) 44.

Zimmermann, Martha, Chong, Adrienne K., Vechiu, Catalina, Papa, Anthony. Modifiable risk and protective factors for anxiety disorders among adults: A systematic review. Psychiatry research 2020. 285, 112705.

A mix of older and younger adult studies, but no separate synthesis for older adults

Abo Youssef, Nadim, Schneider, Marc P., Mordasini, Livio, Ineichen, Benjamin V., Bachmann, Lucas M., Chartier-Kastler, Emmanuel, Panicker, Jalesh N., and Kessler, Thomas M. Cannabinoids for treating neurogenic lower urinary tract dysfunction in patients with multiple sclerosis: a systematic review and meta-analysis. BJU international 2017. 119 (4) 515-521.

Akgun, Katja, Essner, Ute, Seydel, Cordula, and Ziemssen, Tjalf. Daily Practice Managing Resistant Multiple Sclerosis Spasticity With Delta-9-Tetrahydrocannabinol: Cannabidiol Oromucosal Spray: A Systematic Review of Observational Studies. Journal of central nervous system disease 2019. 11, 1179573519831997.

Akram, Hina, Mokrysz, Claire, and Curran, H. Valerie. What are the psychological effects of using synthetic cannabinoids? A systematic review. Journal of psychopharmacology (Oxford, England) 2019. 33 (3) 271-283.

Alharbi, F. F. and El-Guebaly, N. Cannabis and amphetamine-type stimulant-induced psychoses: A systematic overview. Addictive Disorders and their Treatment 2016. 15 (4) 190-200.

Allen, Mark S. and Walter, Emma E. Health-Related Lifestyle Factors and Sexual Dysfunction: A Meta-Analysis of Population-Based Research. The journal of sexual medicine 2018. 15 (4) 458-475.

Allende-Salazar, Ruben F. and Rada, Gabriel. Are cannabinoids an effective treatment for chronic non-cancer pain?. Medwave 2017. 17 (Suppl2) e6972.

Amaniti, Aikaterini, Sardeli, Chrysanthi, Fyntanidou, Varvara, Papakonstantinou, Panagiota, Dalakakis, Ioannis, Mylonas, Antonios, Sapalidis, Konstantinos, Kosmidis, Christoforos, Katsaounis, Athanasios, Giannakidis, Dimitrios, Koulouris, Charilaos, Aidoni, Zoi, Michalopoulos, Nikolaos, Zarogoulidis, Paul, Kesisoglou, Isaak, Ioannidis, Aris, Vagionas, Anastasios, Romanidis, Konstantinos, Oikonomou, Panagoula, Grosomanidis, Vasilios. Pharmacologic and Non-Pharmacologic Interventions for HIV-Neuropathy Pain. A Systematic Review and a Meta-Analysis. Medicina (Kaunas, Lithuania) 2019. 55 (12).

Amlung, Michael, Vedelago, Lana, Acker, John, Balodis, Iris, and MacKillop, James. Steep delay discounting and addictive behavior: a meta-analysis of continuous associations. Addiction (Abingdon, England) 2017. 112 (1) 51-62.

Anderson, Laura Jane, Flynn, Asher, and Pilgrim, Jennifer Lucinda. A global epidemiological perspective on the toxicology of drug-facilitated sexual assault: A systematic review. Journal of forensic and legal medicine 2017. 47, 46-54.

Andreae, Michael H., Carter, George M., Shaparin, Naum, Suslov, Kathryn, Ellis, Ronald J., Ware, Mark A., Abrams, Donald I., Prasad, Hannah, Wilsey, Barth, Indyk, Debbie, Johnson, Matthew, and Sacks, Henry S. Inhaled Cannabis for Chronic Neuropathic Pain: A Meta-analysis of Individual Patient Data. The journal of pain : official journal of the American Pain Society 2015. 16 (12) 1221-1232.

Armentano, Paul. Cannabis and psychomotor performance: a rational review of the evidence and implications for public policy. Drug Testing and Analysis 2013. 5 (1) 52-56.

Artukoglu, Bekir Berker, Beyer, Chad, Zuloff-Shani, Adi, Brener, Ephraim, and Bloch, Michael Howard. Efficacy of Palmitoylethanolamide for Pain: A Meta-Analysis. Pain Physician 2017. 20 (5) 353-362.

Asbridge, Mark, Hayden, Jill A., and Cartwright, Jennifer L. Acute cannabis consumption and motor vehicle collision risk: systematic review of observational studies and meta-analysis. BMJ (Clinical research ed.) 2012. 344, e536.

Asgarian, Fatemeh Sadat, Namdari, Mahshid, Soori, Hamid. Prevalence of mortality in cannabis consumer motorcyclists: meta-analysis of international studies. International journal of injury control and safety promotion 2020. 27 (2) 136-143.

Assi, Sulaf, Gulyamova, Nargilya, Ibrahim, Kinda, Kneller, Paul, and Osselton, David. Profile, effects, and toxicity of novel psychoactive substances: A systematic review of quantitative studies. Human psychopharmacology 2017. 32 (3).

Assi, Sulaf, Gulyamova, Nargilya, Ibrahim, Kinda, Kneller, Paul, and Osselton, David. Profile, effects, and toxicity of novel psychoactive substances: A systematic review of quantitative studies. Human psychopharmacology 2017. 32 (3).

Aviram, J. and Samuelly-Leichtag, G. Efficacy of Cannabis-Based Medicines for Pain Management: A Systematic Review and Meta-Analysis of Randomized Controlled Trials. Pain Physician 2017. 20 (6) E755-E796.

Bacchus, Loraine J., Ranganathan, Meghna, Watts, Charlotte, and Devries, Karen. Recent intimate partner violence against women and health: a systematic review and meta-analysis of cohort studies. BMJ Open 2018. 8 (7) e019995.

Badowski, Melissa and Pandit, Neha Sheth. Pharmacologic management of human immunodeficiency virus wasting syndrome. Pharmacotherapy 2014. 34 (8) 868-881.

Bahji, A. and Mazhar, M. N. Treatment of cannabis dependence with synthetic cannabinoids: A systematic review. Canadian Journal of Addiction 2016. 7 (4) 8-13.

Bahji, Anees, Meyyappan, Arthi Chinna, Hawken, Emily R. Efficacy and acceptability of cannabinoids for anxiety disorders in adults: A systematic review & meta-analysis. Journal of psychiatric research 2020. 129, 257-264.

Bahji, Anees, Stephenson, Callum, Tyo, Richard, Hawken, Emily R., Seitz, Dallas P. Prevalence of Cannabis Withdrawal Symptoms Among People With Regular or Dependent Use of Cannabinoids: A Systematic Review and Meta-analysis. JAMA network open 2020. 3 (4) e202370.

Baldacchino, Alexander, Hughes, Zoe, Kehoe, Michael, Blair, Hannah, Teh, Ying, Windeatt, Stacey, and Crome, Ilana B. Cannabis psychosis: examining the evidence for a distinctive psychopathology in a systematic and narrative review. The American journal on addictions 2012. 21 Suppl 1, S88-S98.

Barbetta, Carlo, Currow, David C., and Johnson, Miriam J. Non-opioid medications for the relief of chronic breathlessness: current evidence. Expert review of respiratory medicine 2017. 11 (4) 333-341.

Bartoli, Francesco, Crocamo, Cristina, and Carra, Giuseppe. Cannabis use disorder and suicide attempts in bipolar disorder: A meta-analysis. Neuroscience and Biobehavioral Reviews 2019.

Batalla, A., Crippa, J. A., Busatto, G. F., Guimaraes, F. S., Zuardi, A. W., Valverde, O., Atakan, Z., McGuire, P. K., Bhattacharyya, S., and Martin-Santos, R. Neuroimaging studies of acute effects of THC and CBD in humans and animals: a systematic review. Current Pharmaceutical Design 2014. 20 (13) 2168-2185.

Batalla, Albert, Bhattacharyya, Sagnik, Yucel, Murat, Fusar-Poli, Paolo, Crippa, Jose Alexandre, Nogue, Santiago, Torrens, Marta, Pujol, Jesus, Farre, Magi, and Martin-Santos, Rocio. Structural and functional imaging studies in chronic cannabis users: a systematic review of adolescent and adult findings. PLoS ONE 2013. 8 (2) e55821.

Batalla, Albert, Janssen, Hella, Bossong, Matthijs G., Gangadin, Shiral S. The potential of cannabidiol as a treatment for psychosis and addiction: Who benefits most? A systematic review. Journal of Clinical Medicine 2019. 8 (7) 1058.

Batalla, Albert, Janssen, Hella, Gangadin, Shiral S., Bossong, Matthijs G. The Potential of Cannabidiol as a Treatment for Psychosis and Addiction: Who Benefits Most? A Systematic Review. Journal of clinical medicine 2019. 8 (7).

Behm, Kate and Morgan, Prue. The effect of symptom-controlling medication on gait outcomes in people with multiple sclerosis: a systematic review. Disability and rehabilitation 2018. 40 (15) 1733-1744.

Belladelli, Federico, Del Giudice, Francesco, Kasman, Alex, Kold Jensen, Tina, Jorgensen, Niels, Salonia, Andrea, Eisenberg, Michael L. The association between cannabis use and testicular function in men: A systematic review and meta-analysis. Andrology 2021. 9 (2) 503-510.

Benze, G., Geyer, A., Alt-Epping, B., and Nauck, F. [Treatment of nausea and vomiting with 5HT3 receptor antagonists, steroids, antihistamines, anticholinergics, somatostatinantagonists, benzodiazepines and cannabinoids in palliative care patients : a systematic review]. Schmerz (Berlin, Germany) 2012. 26 (5) 481-499.

Berry, Meredith S. and Johnson, Matthew W. Does being drunk or high cause HIV sexual risk behavior? A systematic review of drug administration studies. Pharmacology, biochemistry, and behavior 2018. 164, 125-138.

Bhagavan, Chiranth, Kung, Stacey, Doppen, Marjan, John, Mary, Vakalalabure, Iva, Oldfield, Karen, Braithwaite, Irene, Newton-Howes, Giles. Cannabinoids in the Treatment of Insomnia Disorder: A Systematic Review and Meta-Analysis. CNS drugs 2020. 34 (12) 1217-1228.

Biasutti, Wade R., Leffers, Kurt S. H., Callaghan, Russell C. Systematic Review of Cannabis Use and Risk of Occupational Injury. Substance use & misuse 2020. 55 (11) 1733-1745.

Black, Nicola, Stockings, Emily, Campbell, Gabrielle, Tran, Lucy T., Zagic, Dino, Hall, Wayne D., Farrell, Michael, Degenhardt, Louisa. Cannabinoids for the treatment of mental disorders and symptoms of mental disorders: a systematic review and meta-analysis. The lancet. Psychiatry 2019. 6 (12) 995-1010.

Blest-Hopley, Grace, Giampietro, Vincent, and Bhattacharyya, Sagnik. Residual effects of cannabis use in adolescent and adult brains - A meta-analysis of fMRI studies. Neuroscience and Biobehavioral Reviews 2018. 88, 26-41.

Blest-Hopley, Grace, Giampietro, Vincent, Bhattacharyya, Sagnik. A Systematic Review of Human Neuroimaging Evidence of Memory-Related Functional Alterations Associated with Cannabis Use Complemented with Preclinical and Human Evidence of Memory Performance Alterations. Brain sciences 2020. 10 (2).

Blithikioti, Chrysanthi, Miquel, Laia, Batalla, Albert, Rubio, Belen, Maffei, Giovanni, Herreros, Ivan, Gual, Antoni, Verschure, Paul, and Balcells-Olivero, Mercedes. Cerebellar alterations in cannabis users: A systematic review. Addiction Biology 2019.

Bogaty, Sophia E. R., Lee, Rico S. C., Hickie, Ian B., and Hermens, Daniel F. Meta-analysis of neurocognition in young psychosis patients with current cannabis use. Journal of psychiatric research 2018. 99, 22-32.

Borgan, Faith, Beck, Katherine, Butler, Emma, McCutcheon, Robert, Veronese, Mattia, Vernon, Anthony, and Howes, Oliver D. The effects of cannabinoid 1 receptor compounds on memory: a meta-analysis and systematic review across species. Psychopharmacology 2019.

Borges, Guilherme, Bagge, Court, and Orozco, Ricardo. A literature review and meta-analyses of cannabis use and suicidality. Journal of Affective Disorders 2016. 195, 63-74.

Botsford, Sabrina L., Yang, Sharon, George, Tony P. Cannabis and Cannabinoids in Mood and Anxiety Disorders: Impact on Illness Onset and Course, and Assessment of Therapeutic Potential. The American journal on addictions 2020. 29 (1) 9-26.

Bouso, J. C., dos Santos, R. G., Alcazar-Corcoles, M. A., and Hallak, J. E. C. Serotonergic psychedelics and personality: A systematic review of contemporary research. Neuroscience and Biobehavioral Reviews 2018. 87, 118-132.

Bowtell, Meghan, Ratheesh, Aswin, McGorry, Patrick, Killackey, Eoin, and O'Donoghue, Brian. Clinical and demographic predictors of continuing remission or relapse following discontinuation of antipsychotic medication after a first episode of psychosis. A systematic review. Schizophrenia Research 2017.

Bowtell, Meghan, Ratheesh, Aswin, McGorry, Patrick, Killackey, Eoin, and O'Donoghue, Brian. Clinical and demographic predictors of continuing remission or relapse following discontinuation of antipsychotic medication after a first episode of psychosis. A systematic review. Schizophrenia Research 2017.

Boychuk, Darrell G., Goddard, Greg, Mauro, Giovanni, and Orellana, Maria F. The effectiveness of cannabinoids in the management of chronic nonmalignant neuropathic pain: a systematic review. Journal of oral & facial pain and headache 2015. 29 (1) 7-14.

Brabete, Andreea C., Greaves, Lorraine, Hemsing, Natalie, Stinson, Julie. Sex- and Gender-Based Analysis in Cannabis Treatment Outcomes: A Systematic Review. International journal of environmental research and public health 2020. 17 (3).

Breet, Elsie, Goldstone, Daniel, and Bantjes, Jason. Substance use and suicidal ideation and behaviour in low- and middle-income countries: a systematic review. BMC public health 2018. 18 (1) 549.

Brown, Danielle, Watson, Michael, and Schloss, Janet. Pharmacological evidence of medicinal cannabis in oncology: a systematic review. Supportive care in cancer : official journal of the Multinational Association of Supportive Care in Cancer 2019.

Broyd, Samantha J., van Hell, Hendrika H., Beale, Camilla, Yucel, Murat, and Solowij, Nadia. Acute and Chronic Effects of Cannabinoids on Human Cognition-A Systematic Review. Biological Psychiatry 2016. 79 (7) 557-567.

Bunse, Tilmann, Wobrock, Thomas, Strube, Wolfgang, Padberg, Frank, Palm, Ullrich, Falkai, Peter, and Hasan, Alkomiet. Motor cortical excitability assessed by transcranial magnetic stimulation in psychiatric disorders: a systematic review. Brain stimulation 2014. 7 (2) 158-169.

Burns, Jonathan K. Cannabis use and duration of untreated psychosis: a systematic review and meta-analysis. Current Pharmaceutical Design 2012. 18 (32) 5093-5104.

Calabria, Bianca, Degenhardt, Louisa, Hall, Wayne, and Lynskey, Michael. Does cannabis use increase the risk of death? Systematic review of epidemiological evidence on adverse effects of cannabis use. Drug and alcohol review 2010. 29 (3) 318-330.

Campeny, E., Lopez-Pelayo, H., Nutt, D., Blithikioti, C., Oliveras, C., Nuno, L., Maldonado, R., Florez, G., Arias, F., Fernandez-Artamendi, S., Villalbi, J. R., Sellares, J., Ballbe, M., Rehm, J., Balcells-Olivero, M. M., Gual, A. The blind men and the elephant: Systematic review of systematic reviews of cannabis use related health harms. European neuropsychopharmacology : the journal of the European College of Neuropsychopharmacology 2020. 33, 1-35.

Carney, R., Cotter, J., Firth, J., Bradshaw, T., and Yung, A. R. Cannabis use and symptom severity in individuals at ultra high risk for psychosis: a meta-analysis. Acta Psychiatrica Scandinavica 2017. 136 (1) 5-15.

Carrigan, Nicole and Barkus, Emma. A systematic review of the relationship between psychological disorders or substance use and self-reported cognitive failures. Cognitive Neuropsychiatry 2016. 21 (6) 539-564.

Carvalho, Antonelly Cassio Alves de, Souza, Gabriela Achete de, Marqui, Samylla Vaz de, Guiguer, Elen Landgraf, Araujo, Adriano Cressoni, Rubira, Claudio Jose, Goulart, Ricardo de Alvares, Flato, Uri Adrian Prync, Bueno, Patricia Cincotto Dos Santos, Buchaim, Rogerio Leone, Barbalho, Sandra M. Cannabis and Canabidinoids on the Inflammatory Bowel Diseases: Going Beyond Misuse. International journal of molecular sciences 2020. 21 (8).

Casajuana Koguel, Cristina, Lopez-Pelayo, Hugo, Balcells-Olivero, M. Mercedes, Colom, Joan, and Gual, Antoni. Psychoactive constituents of cannabis and their clinical implications: a systematic review. Adicciones 2018. 30 (2) 140-151.

Casajuana, Koguel C., Lopez-Pelayo, H., Balcells-Olivero, M. M., Colom, J., and Gual, A. Constituyentes psicoactivos del cannabis y sus implicaciones clinicas: una revision sistematica, Psychoactive constituents of cannabis and their clinical implications: a systematic review. Adicciones 2018. 30 (2) 140-151.

Castaneto, Marisol S., Wohlfarth, Ariane, Desrosiers, Nathalie A., Hartman, Rebecca L., Gorelick, David A., and Huestis, Marilyn A. Synthetic cannabinoids pharmacokinetics and detection methods in biological matrices. Drug metabolism reviews 2015. 47 (2) 124-174.

Chaaban, Toufic. Acute eosinophilic pneumonia associated with non-cigarette smoking products: a systematic review. Advances in respiratory medicine 2020. 88 (2) 142-146.

Charlet, Katrin, Rosenthal, Annika, Lohoff, Falk W., Heinz, Andreas, and Beck, Anne. Imaging resilience and recovery in alcohol dependence. Addiction (Abingdon, England) 2018. 113 (10) 1933-1950.

Chesney, Edward, Oliver, Dominic, Green, Alastair, Sovi, Simina, Wilson, Jack, Englund, Amir, Freeman, Tom P., McGuire, Philip. Adverse effects of cannabidiol: a systematic review and meta-analysis of randomized clinical trials. Neuropsychopharmacology : official publication of the American College of Neuropsychopharmacology 2020. 45 (11) 1799-1806.

Chisini, Luiz A., Cademartori, Mariana G., Francia, Alejandro, Mederos, Matias, Grazioli, Guillermo, Conde, Marcus C. M., and Correa, Marcos B. Is the use of Cannabis associated with periodontitis? A systematic review and meta-analysis. Journal of periodontal research 2019.

Choenni, Vandhana, Hammink, Alice, and van de Mheen, Dike. Association Between Substance Use and the Perpetration of Family Violence in Industrialized Countries: A Systematic Review. Trauma, violence & abuse 2017. 18 (1) 37-50.

Chow, Ronald, Valdez, Crystal, Chow, Natalie, Zhang, Daniel, Im, James, Sodhi, Emily, Lock, Michael. Oral cannabinoid for the prophylaxis of chemotherapy-induced nausea and vomiting-a systematic review and meta-analysis. Supportive care in cancer : official journal of the Multinational Association of Supportive Care in Cancer 2020. 28 (5) 2095-2103.

Clark, Thomas M., Jones, Jessica M., Hall, Alexis G., Tabner, Sara A., and Kmiec, Rebecca L. Theoretical Explanation for Reduced Body Mass Index and Obesity Rates in Cannabis Users. Cannabis and Cannabinoid Research 2018. 3 (1) 259-271.

Coentre, Ricardo, Talina, Miguel Cotrim, Gois, Carlos, and Figueira, Maria Luisa. Depressive symptoms and suicidal behavior after first-episode psychosis: A comprehensive systematic review. Psychiatry Research 2017. 253, 240-248.

Cohen, Koby and Weinstein, Aviv. The Effects of Cannabinoids on Executive Functions: Evidence from Cannabis and Synthetic Cannabinoids-A Systematic Review. Brain sciences 2018. 8 (3).

Colizzi, Marco and Bhattacharyya, Sagnik. Cannabis use and the development of tolerance: a systematic review of human evidence. Neuroscience and Biobehavioral Reviews 2018. 93, 1-25.

Colizzi, Marco, McGuire, Philip, Pertwee, Roger G., and Bhattacharyya, Sagnik. Effect of cannabis on glutamate signalling in the brain: A systematic review of human and animal evidence. Neuroscience and Biobehavioral Reviews 2016. 64, 359-381.

Contreras, Tania, Bravo-Soto, Gonzalo, and Rada, Gabriel. Do cannabinoids constitute a therapeutic alternative for insomnia?. Medwave 2018. 18 (1) e7152.

Cookey, Jacob, Bernier, Denise, and Tibbo, Philip G. White matter changes in early phase schizophrenia and cannabis use: an update and systematic review of diffusion tensor imaging studies. Schizophrenia Research 2014. 156 (2-3) 137-142.

Cotter, Jayme. Efficacy of Crude Marijuana and Synthetic Delta-9-Tetrahydrocannabinol as Treatment for Chemotherapy-Induced Nausea and Vomiting: A Systematic Literature Review. Oncology nursing forum 2009. 36 (3) 345-352.

Couch, Daniel G., Maudslay, Henry, Doleman, Brett, Lund, Jonathan N., and O'Sullivan, Saoirse E. The Use of Cannabinoids in Colitis: A Systematic Review and Meta-Analysis. Inflammatory Bowel Diseases 2018. 24 (4) 680-697.

Courts, Julie, Maskill, Virginia, Gray, Andrew, and Glue, Paul. Signs and symptoms associated with synthetic cannabinoid toxicity: systematic review. Australasian psychiatry : bulletin of Royal Australian and New Zealand College of Psychiatrists 2016. 24 (6) 598-601.

Crippa, Jose As, Derenusson, Guilherme N., Chagas, Marcos Hn, Atakan, Zerrin, Martin-Santos, Rocio, Zuardi, Antonio W., and Hallak, Jaime Ec. Pharmacological interventions in the treatment of the acute effects of cannabis: a systematic review of literature. Harm reduction journal 2012. 9, 7.

Curtis, Adrienne, Clarke, Carl E., and Rickards, Hugh E. Cannabinoids for Tourette's Syndrome. The Cochrane database of systematic reviews 2009. (4) CD006565.

da Rovare, Victoria P., Magalhaes, Gabriel P. A., Jardini, Guilherme D. A., Beraldo, Matheus L., Gameiro, Mariel O., Agarwal, Arnav, Luvizutto, Gustavo Jose, Paula-Ramos, Lucas, Camargo, Samira Esteves Afonso, de Oliveira, Luciane Dias, Bazan, Rodrigo, and El Dib, Regina. Cannabinoids for spasticity due to multiple sclerosis or paraplegia: A systematic review and meta-analysis of randomized clinical trials. Complementary therapies in medicine 2017. 34, 170-185.

de Carvalho Reis, Renandro, Almeida, Kelson James, da Silva Lopes, Luciano, de Melo Mendes, Cintia Maria, Bor-Seng-Shu, Edson. Efficacy and adverse event profile of cannabidiol and medicinal cannabis for treatment-resistant epilepsy: Systematic review and meta-analysis. Epilepsy & behavior : E&B 2020. 102, 106635.

de Carvalho, M. F. F., Dourado, M. R., Fernandes, I. B., Araujo, C. T. P., Mesquita, A. T., and Ramos-Jorge, M. L. Head and neck cancer among marijuana users: a meta-analysis of matched case-control studies. Archives of oral biology 2015. 60 (12) 1750-1755.

De Vita, Martin J., Moskal, Dezarie, Maisto, Stephen A., and Ansell, Emily B. Association of Cannabinoid Administration With Experimental Pain in Healthy Adults: A Systematic Review and Meta-analysis. JAMA Psychiatry 2018. 75 (11) 1118-1127.

Degenhardt, Louisa, Ferrari, Alize J., Calabria, Bianca, Hall, Wayne D., Norman, Rosana E., McGrath, John, Flaxman, Abraham D., Engell, Rebecca E., Freedman, Greg D., Whiteford, Harvey A., and Vos, Theo. The global epidemiology and contribution of cannabis use and dependence to the global burden of disease: results from the GBD 2010 study. PLoS ONE 2013. 8 (10) e76635.

Dellazizzo, L., Potvin, S., Beaudoin, M., Luigi, M., Dou, B. Y., Giguere, C.-E., and Dumais, A. Cannabis use and violence in patients with severe mental illnesses: A meta-analytical investigation. Psychiatry Research 2019. 274, 42-48.

Denier, Niklaus, Walter, Marc, Bendfeldt, Kerstin, Lang, Undine, and Borgwardt, Stefan. Resting state abnormalities in psychosis compared to acute cannabinoids and opioids challenges: a systematic review of functional imaging studies. Current Pharmaceutical Design 2012. 18 (32) 5081-5092.

Deshpande, Amol, Mailis-Gagnon, Angela, Zoheiry, Nivan, and Lakha, Shehnaz Fatima. Efficacy and adverse effects of medical marijuana for chronic noncancer pain: Systematic review of randomized controlled trials. Canadian family physician Medecin de famille canadien 2015. 61 (8) e372-e381.

Devinsky, Orrin, Thiele, Elizabeth A., Wright, Stephen, Checketts, Daniel, Morrison, Gilmour, Dunayevich, Eduardo, Knappertz, Volker. Cannabidiol efficacy independent of clobazam: Meta-analysis of four randomized controlled trials. Acta neurologica Scandinavica 2020. 142 (6) 531-540.

Doeve, Benthe H., van de Meeberg, Maartje M., van Schaik, Fiona D. M., Fidder, Herma H. A Systematic Review With Meta-Analysis of the Efficacy of Cannabis and Cannabinoids for Inflammatory Bowel Disease: What Can We Learn From Randomized and Nonrandomized Studies?. Journal of clinical gastroenterology 2020.

Donde, Clement, Achim, Amelie M., Brunelin, Jerome, Poulet, Emmanuel, Mondino, Marine, Haesebaert, Frederic. A meta-analysis of craving studies in schizophrenia spectrum disorders. Schizophrenia research 2020. 222, 49-57.

Dosenovic, Svjetlana, Jelicic Kadic, Antonia, Miljanovic, Maja, Biocic, Marina, Boric, Krste, Cavar, Marija, Markovina, Nikolina, Vucic, Katarina, and Puljak, Livia. Interventions for Neuropathic Pain: An Overview of Systematic Reviews. Anesthesia and analgesia 2017. 125 (2) 643-652.

Dualibe, Aline Limieri and Osorio, Flavia L. Bipolar Disorder and Early Emotional Trauma: A Critical Literature Review on Indicators of Prevalence Rates and Clinical Outcomes. Harvard review of psychiatry 2017. 25 (5) 198-208.

Duperrouzel, Jacqueline C., Granja, Karen, Pacheco-Colon, Ileana, Gonzalez, Raul. Adverse Effects of Cannabis Use on Neurocognitive Functioning: A Systematic Review of Meta- Analytic Studies. Journal of dual diagnosis 2020. 16 (1) 43-57.

Elvik, Rune. Risk of road accident associated with the use of drugs: a systematic review and meta-analysis of evidence from epidemiological studies. Accident; analysis and prevention 2013. 60, 254-267.

Ergul, Merziye, Nodehi Moghadam, Afsun, Soh, Rachel. The effectiveness of interventions targeting spasticity on functional clinical outcomes in patients with multiple sclerosis: a systematic review of clinical trials. European Journal of Physiotherapy 2020.

Escelsior, Andrea, Belvederi Murri, Martino, Corsini, Giovanni Pietro, Serafini, Gianluca, Aguglia, Andrea, Zampogna, Domenico, Cattedra, Simone, Nebbia, Jacopo, Trabucco, Alice, Prestia, Davide, Olcese, Martina, Barletta, Elena, Pereira da Silva, Beatriz, Amore, Mario. Cannabinoid use and self-injurious behaviours: A systematic review and meta-analysis. Journal of affective disorders 2021. 278, 85-98.

Farooqui, Muhammad T., Khan, Muhammad A., Cholankeril, George, Khan, Zubair, Mohammed Abdul, Mubeen K., Li, Andrew A., Shah, Neha, Wu, Lin, Haq, Khwaja, Solanki, Shantanu, Kim, Donghee, and Ahmed, Aijaz. Marijuana is not associated with progression of hepatic fibrosis in liver disease: a systematic review and meta-analysis. European journal of gastroenterology & hepatology 2019. 31 (2) 149-156.

Farris, Megan S., Shakeel, Mohammed K., Addington, Jean. Cannabis use in individuals at clinical high-risk for psychosis: a comprehensive review. Social psychiatry and psychiatric epidemiology 2020. 55 (5) 527-537.

Figueiredo, Pedro Rafael, Tolomeo, Serenella, Steele, J. Douglas, Baldacchino, Alexander. Neurocognitive consequences of chronic cannabis use: a systematic review and meta-analysis. Neuroscience and biobehavioral reviews 2020. 108, 358-369.

Fischer, Benedikt, Russell, Cayley, Sabioni, Pamela, van den Brink, Wim, Le Foll, Bernard, Hall, Wayne, Rehm, Jurgen, and Room, Robin. Lower-Risk Cannabis Use Guidelines: A Comprehensive Update of Evidence and Recommendations. American journal of public health 2017. 107 (8) e1-e12.

Fitzcharles, Mary Ann, Ste-Marie, Peter A., Hauser, Winfried, Clauw, Daniel J., Jamal, Shahin, Karsh, Jacob, Landry, Tara, Leclercq, Sharon, Mcdougall, Jason J., Shir, Yoram, Shojania, Kam, and Walsh, Zach. Efficacy, Tolerability, and Safety of Cannabinoid Treatments in the Rheumatic Diseases: A Systematic Review of Randomized Controlled Trials. Arthritis care & research 2016. 68 (5) 681-688.

Foglia, E., Schoeler, T., Klamerus, E., Morgan, K., and Bhattacharyya, S. Cannabis use and adherence to antipsychotic medication: a systematic review and meta-analysis. Psychological Medicine 2017. 47 (10) 1691-1705.

Fond, Guillaume, Lancon, Christophe, Auquier, Pascal, and Boyer, Laurent. [Prevalence of major depression in France in the general population and in specific populations from 2000 to 2018: A systematic review of the literature]. Presse medicale (Paris, France : 1983) 2019. 48 (4) 365-375.

Freeman, Abigail M., Petrilli, Katherine, Lees, Rachel, Hindocha, Chandni, Mokrysz, Claire, Curran, H. Valerie, Saunders, Rob, Freeman, Tom P. How does cannabidiol (CBD) influence the acute effects of delta-9-tetrahydrocannabinol (THC) in humans? A systematic review. Neuroscience and biobehavioral reviews 2019. 107, 696-712.

French, Clare E., Coope, Caroline M., McGuinness, Luke A., Beck, Charles R., Newitt, Sophie, Ahyow, Lauren, Hickman, Matt, Oliver, Isabel. Cannabis use and the risk of tuberculosis: a systematic review. BMC public health 2019. 19 (1) 1006.

Friedman, D. and Devinsky, O. Cannabinoids in the treatment of epilepsy. New England Journal of Medicine 2015. 373 (11) 1048-1058.

Fu, Xiying, Wang, Yanqiao, Wang, Can, Wu, Huijie, Li, Jinyao, Li, Ming, Ma, Qianqian, and Yang, Wei. A mixed treatment comparison on efficacy and safety of treatments for spasticity caused by multiple sclerosis: a systematic review and network meta-analysis. Clinical rehabilitation 2018. 32 (6) 713-721.

Fusar-Poli, Laura, Cavone, Vito, Tinacci, Silvia, Concas, Ilaria, Petralia, Antonino, Signorelli, Maria Salvina, Diaz-Caneja, Covadonga M., Aguglia, Eugenio. Cannabinoids for People with ASD: A Systematic Review of Published and Ongoing Studies. Brain sciences 2020. 10 (9).

Gandhi, Shreyas, Vasisth, Gaurav, and Kapoor, Anil. Systematic review of the potential role of cannabinoids as antiproliferative agents for urological cancers. Canadian Urological Association journal = Journal de l'Association des urologues du Canada 2017. 11 (3-4) E138-E142.

Garcia, Karen and Rada, Gabriel. Do cannabinoids have a role to play in Tourette's syndrome?. Medwave 2016. 16 (Suppl5) e6793.

Gates, Peter J., Albertella, Lucy, and Copeland, Jan. The effects of cannabinoid administration on sleep: a systematic review of human studies. Sleep Medicine Reviews 2014. 18 (6) 477-487.

Gates, Peter, Albertella, Lucy, and Copeland, Jan. Cannabis withdrawal and sleep: A systematic review of human studies. Substance abuse 2016. 37 (1) 255-269.

Geoffroy, Pierre Alexis, Etain, Bruno, and Houenou, Josselin. Gene x environment interactions in schizophrenia and bipolar disorder: evidence from neuroimaging. Frontiers in Psychiatry 2013. 4, 136.

Ghabrash, Maykel Farag, Coronado-Montoya, Stephanie, Aoun, John, Gagne, Andree-Anne, Mansour, Flavi, Ouellet-Plamondon, Clairelaine, Trepanier, Annie, Jutras-Aswad, Didier. Cannabidiol for the treatment of psychosis among patients with schizophrenia and other primary psychotic disorders: A systematic review with a risk of bias assessment. Psychiatry research 2020. 286, 112890.

Gibbs, Melanie, Winsper, Catherine, Marwaha, Steven, Gilbert, Eleanor, Broome, Matthew, and Singh, Swaran P. Cannabis use and mania symptoms: a systematic review and meta-analysis. Journal of Affective Disorders 2015. 171, 39-47.

Giorgetti, Arianna, Busardo, Francesco Paolo, Tittarelli, Roberta, Auwarter, Volker, Giorgetti, Raffaele. Post-Mortem Toxicology: A Systematic Review of Death Cases Involving Synthetic Cannabinoid Receptor Agonists. Frontiers in psychiatry 2020. 11, 464.

Gloss, David and Vickrey, Barbara. Cannabinoids for epilepsy. The Cochrane database of systematic reviews 2014. (3) CD009270.

Goldenberg, Matthew, IsHak, Waguih William, and Danovitch, Itai. Quality of life and recreational cannabis use. The American journal on addictions 2017. 26 (1) 8-25.

Gomez Ochoa, S. A. Stroke and cannabis use in patients with no cardiovascular risk factors: A systematic review of case reports. Neurologia 2017.

Gorey, Claire, Kuhns, Lauren, Smaragdi, Eleni, Kroon, Emese, and Cousijn, Janna. Age-related differences in the impact of cannabis use on the brain and cognition: a systematic review. European Archives of Psychiatry and Clinical Neuroscience 2019. 269 (1) 37-58.

Gray, R., Bressington, D., Hughes, E., and Ivanecka, A. A systematic review of the effects of novel psychoactive substances 'legal highs' on people with severe mental illness. Journal of psychiatric and mental health nursing 2016. 23 (5) 267-281.

Guillouard, M., Authier, N., Pereira, B., Soubrier, M., Mathieu, S. Cannabis use assessment and its impact on pain in rheumatologic diseases: a systematic review and meta-analysis. Rheumatology (Oxford, England) 2021. 60 (2) 549-556.

Guinguis, Rami, Ruiz, Maria Isabel, and Rada, Gabriel. Is cannabidiol an effective treatment for schizophrenia?. Medwave 2017. 17 (7) e7010.

Gunderson, Erik W., Haughey, Heather M., Ait-Daoud, Nassima, Joshi, Amruta S., and Hart, Carl L. "Spice" and "K2" herbal highs: a case series and systematic review of the clinical effects and biopsychosocial implications of synthetic cannabinoid use in humans. The American journal on addictions 2012. 21 (4) 320-326.

Gunning, Boudewijn, Mazurkiewicz-Beldzinska, Maria, Chin, Richard F. M., Bhathal, Hari, Nortvedt, Charlotte, Dunayevich, Eduardo, Checketts, Daniel. Cannabidiol in conjunction with clobazam: analysis of four randomized controlled trials. Acta neurologica Scandinavica 2021. 143 (2) 154-163.

Haden, Mark, Archer, John R. H., Dargan, Paul I., and Wood, David M. MDMB-CHMICA: Availability, Patterns of Use, and Toxicity Associated With This Novel Psychoactive Substance. Substance use & misuse 2017. 52 (2) 223-232.

Hasan, Alkomiet, von Keller, Rupert, Friemel, Chris Maria, Hall, Wayne, Schneider, Miriam, Koethe, Dagmar, Leweke, F. Markus, Strube, Wolfgang, Hoch, Eva. Cannabis use and psychosis: a review of reviews. European archives of psychiatry and clinical neuroscience 2020. 270 (4) 403-412.

Hellem, Tracy, Shi, Xianfeng, Latendresse, Gwen, and Renshaw, Perry F. The Utility of Magnetic Resonance Spectroscopy for Understanding Substance Use Disorders: A Systematic Review of the Literature. Journal of the American Psychiatric Nurses Association 2015. 21 (4) 244-275.

Herzog, Samuel, Shanahan, Marian, Grimison, Peter, Tran, Anh, Wong, Nicole, Lintzeris, Nicholas, Simes, John, Stockler, Martin, and Morton, Rachael L. Systematic Review of the Costs and Benefits of Prescribed Cannabis-Based Medicines for the Management of Chronic Illness: Lessons from Multiple Sclerosis. PharmacoEconomics 2018. 36 (1) 67-78.

Hindley, Guy, Beck, Katherine, Borgan, Faith, Ginestet, Cedric E., McCutcheon, Robert, Kleinloog, Daniel, Ganesh, Suhas, Radhakrishnan, Rajiv, D'Souza, Deepak Cyril, Howes, Oliver D. Psychiatric symptoms caused by cannabis constituents: a systematic review and meta-analysis. The lancet. Psychiatry 2020. 7 (4) 344-353.

Hobbs, Melissa, Kalk, Nicola J., Morrison, Paul D., and Stone, James M. Spicing it up - synthetic cannabinoid receptor agonists and psychosis - a systematic review. European neuropsychopharmacology : the journal of the European College of Neuropsychopharmacology 2018. 28 (12) 1289-1304.

Holitzki, Hannah, Dowsett, Laura E., Spackman, Eldon, Noseworthy, Tom, and Clement, Fiona. Health effects of exposure to second- and third-hand marijuana smoke: a systematic review. CMAJ open 2017. 5 (4) E814-E822.

Hostiuc, Sorin, Moldoveanu, Alin, Negoi, Ionut, and Drima, Eduard. The Association of Unfavorable Traffic Events and Cannabis Usage: A Meta-Analysis. Frontiers in Pharmacology 2018. 9, 99.

Houze, Berengere, El-Khatib, Hejar, and Arbour, Caroline. Reprint of: Efficacy, tolerability, and safety of non-pharmacological therapies for chronic pain: An umbrella review on various CAM approaches. Progress in neuro-psychopharmacology & biological psychiatry 2018. 87 (Pt B) 307-321.

Huestis, Marilyn A., Solimini, Renata, Pichini, Simona, Pacifici, Roberta, Carlier, Jeremy, Busardo, Francesco Paolo. Cannabidiol adverse effects and toxicity. Current Neuropharmacology 2019. 17 (10) 974-989.

Huntley, A. and Ernst, E. Herbal medicines for asthma: a systematic review. Thorax 2000. 55 (11) 925-929.

Ilic, Boris, Svab, Vesna, Sedic, Biserka, Kovacevic, Irena, Friganovic, Adriano, and Juric, Ena. Mental Health in Domesticated Immigrant Population - a Systematic Review. Psychiatria Danubina 2017. 29 (3) 273-281.

Iseger, Tabitha A. and Bossong, Matthijs G. A systematic review of the antipsychotic properties of cannabidiol in humans. Schizophrenia Research 2015. 162 (1-3) 153-161.

IsHak, Waguih William, Wen, Raymond Y., Naghdechi, Lancer, Vanle, Brigitte, Dang, Jonathan, Knosp, Michelle, Dascal, Julieta, Marcia, Lobsang, Gohar, Yasmine, Eskander, Lidia, Yadegar, Justin, Hanna, Sophia, Sadek, Antonious, Aguilar-Hernandez, Leslie, Danovitch, Itai, and Louy, Charles. Pain and Depression: A Systematic Review. Harvard review of psychiatry 2018. 26 (6) 352-363.

Jacobson, Maya R., Watts, Jeremy J., Boileau, Isabelle, Tong, Junchao, and Mizrahi, Romina. A systematic review of phytocannabinoid exposure on the endocannabinoid system: Implications for psychosis. European neuropsychopharmacology : the journal of the European College of Neuropsychopharmacology 2019. 29 (3) 330-348.

Jawahar, Rachel, Oh, Unsong, Yang, Shibing, and Lapane, Kate L. A systematic review of pharmacological pain management in multiple sclerosis. Drugs 2013. 73 (15) 1711-1722.

Jonas, Andrea M., Raj, Rishi. Vaping-Related Acute Parenchymal Lung Injury: A Systematic Review. Chest 2020. 158 (4) 1555-1565.

Jouanjus, Emilie, Raymond, Valentin, Lapeyre-Mestre, Maryse, and Wolff, Valerie. What is the Current Knowledge About the Cardiovascular Risk for Users of Cannabis-Based Products? A Systematic Review. Current atherosclerosis reports 2017. 19 (6) 26.

Jung, Flora, Lee, Yung, Manzoor, Saba, Hong, Dennis, Doumouras, Aristithes G. Effects of Perioperative Cannabis Use on Bariatric Surgical Outcomes: a Systematic Review. Obesity surgery 2021. 31 (1) 299-306.

Kafil, Tahir S., Nguyen, Tran M., MacDonald, John K., and Chande, Nilesh. Cannabis for the treatment of Crohn's disease. The Cochrane database of systematic reviews 2018. 11, CD012853.

Kafil, Tahir S., Nguyen, Tran M., MacDonald, John K., and Chande, Nilesh. Cannabis for the treatment of ulcerative colitis. The Cochrane database of systematic reviews 2018. 11, CD012954.

Kafil, Tahir S., Nguyen, Tran M., MacDonald, John K., Chande, Nilesh. Cannabis for the Treatment of Crohn's Disease and Ulcerative Colitis: Evidence From Cochrane Reviews. Inflammatory bowel diseases 2020. 26 (4) 502-509.

Kamp, Felicia, Proebstl, Lisa, Penzel, Nora, Adorjan, Kristina, Ilankovic, Andrej, Pogarell, Oliver, Koller, Gabi, Soyka, Michael, Falkai, Peter, Koutsouleris, Nikolaos, and Kambeitz, Joseph. Effects of sedative drug use on the dopamine system: a systematic review and meta-analysis of in vivo neuroimaging studies. Neuropsychopharmacology : official publication of the American College of Neuropsychopharmacology 2019. 44 (4) 660-667.

Karyadi, Kenny A., VanderVeen, J. Davis, and Cyders, Melissa A. A meta-analysis of the relationship between trait mindfulness and substance use behaviors. Drug and Alcohol Dependence 2014. 143, 1-10.

Katchan, Valeria, David, Paula, and Shoenfeld, Yehuda. Cannabinoids and autoimmune diseases: A systematic review. Autoimmunity reviews 2016. 15 (6) 513-528.

Keboa, Mark Tambe, Enriquez, Ninoska, Martel, Marc, Nicolau, Belinda, Macdonald, Mary Ellen. Oral Health Implications of Cannabis Smoking: A Rapid Evidence Review. Journal (Canadian Dental Association) 2020. 86, k2.

Kedzior, Karina Karolina and Laeber, Lisa Tabata. A positive association between anxiety disorders and cannabis use or cannabis use disorders in the general population--a meta-analysis of 31 studies. BMC Psychiatry 2014. 14, 136.

Keeley, P. W. Nausea and vomiting in people with cancer and other chronic diseases. BMJ clinical evidence 2009. 2009.

Kennedy, Michael C. Cannabis: Exercise performance and sport. A systematic review. Journal of science and medicine in sport 2017. 20 (9) 825-829.

Khan, Rabia, Raafey, Muhammad Abdur, Naveed, Sadiq, Mian, Nadeem, Fida, Ania, Aedma, Kapil Kiran. The therapeutic role of Cannabidiol in mental health: A systematic review. Journal of Cannabis Research 2020. 2 (1) 2.

Khoury, Julia Machado, Neves, Maila de Castro Lourenco das, Roque, Marco Antonio Valente, Queiroz, Daniela Alves de Brito, Correa de Freitas, Andre Augusto, de Fatima, Angelo, Moreira, Fabricio A., and Garcia, Frederico Duarte. Is there a role for cannabidiol in psychiatry?. The world journal of biological psychiatry : the official journal of the World Federation of Societies of Biological Psychiatry 2019. 20 (2) 101-116.

Klugah-Brown, Benjamin, Di, Xin, Zweerings, Jana, Mathiak, Klaus, Becker, Benjamin, Biswal, Bharat. Common and separable neural alterations in substance use disorders: A coordinate-based meta-analyses of functional neuroimaging studies in humans. Human brain mapping 2020. 41 (16) 4459-4477.

Kondo, Karli K., Morasco, Benjamin J., Nugent, Shannon M., Ayers, Chelsea K., O'Neil, Maya E., Freeman, Michele, Kansagara, Devan. Pharmacotherapy for the Treatment of Cannabis Use Disorder: A Systematic Review. Annals of internal medicine 2020. 172 (6) 398-412.

Kondo, Karli, Morasco, Benjamin J., Nugent, Shannon, Ayers, Chelsea, O'Neil, Maya E., Freeman, Michele, Paynter, Robin, Kansagara, Devan. 2019.

Kopelli, Eleftheria, Samara, Myrto, Siargkas, Antonios, Goulas, Antonis, Papazisis, Georgios, Chourdakis, Michail. The role of cannabidiol oil in schizophrenia treatment. a systematic review and meta-analysis. Psychiatry research 2020. 291, 113246.

Kosiba, Jesse D., Maisto, Stephen A., Ditre, Joseph W. Patient-reported use of medical cannabis for pain, anxiety, and depression symptoms: Systematic review and meta-analysis. Social science & medicine (1982) 2019. 233, 181-192.

Krzyzanowski, Daniel J., Purdon, Scot E. Duration of abstinence from cannabis is positively associated with verbal learning performance: A systematic review and meta-analysis. Neuropsychology 2020. 34 (3) 359-372.

Lake, Stephanie, St Pierre, Michelle. The relationship between cannabis use and patient outcomes in medication-based treatment of opioid use disorder: A systematic review. Clinical psychology review 2020. 82, 101939.

Lakhan, Shaheen E. and Rowland, Marie. Whole plant cannabis extracts in the treatment of spasticity in multiple sclerosis: A systematic review. BMC Neurology 2009. 9.

Langhorst, J., Wulfert, H., Lauche, R., Klose, P., Cramer, H., Dobos, G. J., and Korzenik, J. Systematic review of complementary and alternative medicine treatments in inflammatory bowel diseases. Journal of Crohn's & colitis 2015. 9 (1) 86-106.

Laprevote, Vincent, Schwan, Raymund, Schwitzer, Thomas, Rolland, Benjamin, and Thome, Johannes. Is There a Place for Off-Label Pharmacotherapy in Cannabis Use Disorder? A Review on Efficacy and Safety. Current Pharmaceutical Design 2015. 21 (23) 3298-3305.

Lattanzi, Simona, Brigo, Francesco, Cagnetti, Claudia, Trinka, Eugen, and Silvestrini, Mauro. Efficacy and Safety of Adjunctive Cannabidiol in Patients with Lennox-Gastaut Syndrome: A Systematic Review and Meta-Analysis. CNS Drugs 2018. 32 (10) 905-916.

Lattanzi, Simona, Brigo, Francesco, Trinka, Eugen, Zaccara, Gaetano, Cagnetti, Claudia, Del Giovane, Cinzia, and Silvestrini, Mauro. Efficacy and Safety of Cannabidiol in Epilepsy: A Systematic Review and Meta-Analysis. Drugs 2018. 78 (17) 1791-1804.

Lattanzi, Simona, Trinka, Eugen, Striano, Pasquale, Zaccara, Gaetano, Del Giovane, Cinzia, Nardone, Raffaele, Silvestrini, Mauro, Brigo, Francesco. Cannabidiol efficacy and clobazam status: A systematic review and meta-analysis. Epilepsia 2020. 61 (6) 1090-1098.

Le Bec, P. Y., Fatseas, M., Denis, C., Lavie, E., and Auriacombe, M. [Cannabis and psychosis: search of a causal link through a critical and systematic review]. L'Encephale 2009. 35 (4) 377-385.

Lee, Rico S. C., Hoppenbrouwers, Sylco, and Franken, Ingmar. A Systematic Meta-Review of Impulsivity and Compulsivity in Addictive Behaviors. Neuropsychology review 2019. 29 (1) 14-26.

Leung, Janni, Chan, Gary C. K., Hides, Leanne, Hall, Wayne D. What is the prevalence and risk of cannabis use disorders among people who use cannabis? a systematic review and meta-analysis. Addictive behaviors 2020. 109, 106479.

Lev-Ran, S., Roerecke, M., Le Foll, B., George, T. P., McKenzie, K., and Rehm, J. The association between cannabis use and depression: a systematic review and meta-analysis of longitudinal studies. Psychological Medicine 2014. 44 (4) 797-810.

Li, Mu Chen, Brady, Joanne E., Dimaggio, Charles J., Lusardi, Arielle R., Tzong, Keane Y., and Li, Guohua. Marijuana use and motor vehicle crashes. Epidemiologic Reviews 2012. 34, 65-72.

Linscott, R. J. and van Os, J. An updated and conservative systematic review and meta-analysis of epidemiological evidence on psychotic experiences in children and adults: on the pathway from proneness to persistence to dimensional expression across mental disorders. Psychological Medicine 2013. 43 (6) 1133-1149.

Liu, Yang, van den Wildenberg, Wery P. M., de Graaf, Ysanne, Ames, Susan L., Baldacchino, Alexander, Bo, Ragnhild, Cadaveira, Fernando, Campanella, Salvatore, Christiansen, Paul, Claus, Eric D., Colzato, Lorenza S., Filbey, Francesca M., Foxe, John J., Garavan, Hugh, Hendershot, Christian S., Hester, Robert, Jester, Jennifer M., Karoly, Hollis C., Kraplin, Anja, Kreusch, Fanny, Landro, Nils Inge, Littel, Marianne, Loeber, Sabine, London, Edythe D., Lopez-Caneda, Eduardo, Lubman, Dan I., Luijten, Maartje, Marczinski, Cecile A., Metrik, Jane, Montgomery, Catharine, Papachristou, Harilaos, Mi Park, Su, Paz, Andres L., Petit, Geraldine, Prisciandaro, James J., Quednow, Boris B., Ray, Lara A., Roberts, Carl A., Roberts, Gloria M. P., de Ruiter, Michiel B., Rupp, Claudia I., Steele, Vaughn R., Sun, Delin, Takagi, Michael, Tapert, Susan F., van Holst, Ruth J., Verdejo-Garcia, Antonio, Vonmoos, Matthias, Wojnar, Marcin, Yao, Yuanwei, Yucel, Murat, Zack, Martin, Zucker, Robert A., Huizenga, Hilde M., Wiers, Reinout W. Is (poly-) substance use associated with impaired inhibitory control? A mega-analysis controlling for confounders. Neuroscience and biobehavioral reviews 2019. 105, 288-304.

Liu, Yiyang, Williamson, Victoria, Setlow, Barry, Cottler, Linda B., and Knackstedt, Lori A. The importance of considering polysubstance use: lessons from cocaine research. Drug and Alcohol Dependence 2018. 192, 16-28.

Lopez, A. P., Roque, I. Figuls, Cuchi, G. U., Berenstein, E. G., Pasies, B. A., Alegre, M. B., and Herdman, M. Systematic review of megestrol acetate in the treatment of anorexia-cachexia syndrome. Journal of Pain and Symptom Management 2004. 27 (4) 360-369.

Lovell, Monica E., Akhurst, Jane, Padgett, Christine, Garry, Michael I., Matthews, Allison. Cognitive outcomes associated with long-term, regular, recreational cannabis use in adults: A meta-analysis. Experimental and clinical psychopharmacology 2020. 28 (4) 471-494.

Luijten, Maartje, Schellekens, Arnt F., Kuhn, Simone, Machielse, Marise W. J., and Sescousse, Guillaume. Disruption of Reward Processing in Addiction : An Image-Based Meta-analysis of Functional Magnetic Resonance Imaging Studies. JAMA Psychiatry 2017. 74 (4) 387-398.

Lutge, Elizabeth E., Gray, Andy, and Siegfried, Nandi. The medical use of cannabis for reducing morbidity and mortality in patients with HIV/AIDS. The Cochrane database of systematic reviews 2013. (4) CD005175.

Lynch, M. E. and Ware, Mark A. Cannabinoids for the Treatment of Chronic Non-Cancer Pain: An Updated Systematic Review of Randomized Controlled Trials. Journal of neuroimmune pharmacology : the official journal of the Society on NeuroImmune Pharmacology 2015. 10 (2) 293-301.

Lynch, Mary E. and Campbell, Fiona. Cannabinoids for treatment of chronic non-cancer pain; a systematic review of randomized trials. British Journal of Clinical Pharmacology 2011. 72 (5) 735-744.

Machado Rocha, F. C., Stefano, S. C., De Cassia Haiek, R., Rosa Oliveira, L. M. Q., and Da Silveira, D. X. Therapeutic use of Cannabis sativa on chemotherapy-induced nausea and vomiting among cancer patients: systematic review and meta-analysis. European journal of cancer care 2008. 17 (5) 431-443.

Madden, Kim, van der Hoek, Niek, Chona, Simrun, George, Annie, Dalchand, Tristiana, Baldawi, Hassan, Mammen, George, and Bhandari, Mohit. Cannabinoids in the Management of Musculoskeletal Pain: A Critical Review of the Evidence. JBJS reviews 2018. 6 (5) e7.

Malchow, Berend, Hasan, Alkomiet, Fusar-Poli, Paolo, Schmitt, Andrea, Falkai, Peter, and Wobrock, Thomas. Cannabis abuse and brain morphology in schizophrenia: a review of the available evidence. European Archives of Psychiatry and Clinical Neuroscience 2013. 263 (1) 3-13.

Mammen, George, Rueda, Sergio, Roerecke, Michael, Bonato, Sarah, Lev-Ran, Shaul, and Rehm, Jurgen. Association of Cannabis With Long-Term Clinical Symptoms in Anxiety and Mood Disorders: A Systematic Review of Prospective Studies. The Journal of clinical psychiatry 2018. 79 (4).

Marangoni, Ciro, Hernandez, Mariely, and Faedda, Gianni L. The role of environmental exposures as risk factors for bipolar disorder: A systematic review of longitudinal studies. Journal of Affective Disorders 2016. 193, 165-174.

Marconi, Arianna, Di Forti, Marta, Lewis, Cathryn M., Murray, Robin M., and Vassos, Evangelos. Meta-analysis of the Association Between the Level of Cannabis Use and Risk of Psychosis. Schizophrenia Bulletin 2016. 42 (5) 1262-1269.

Martin, Eric, Battaglini, Claudio. Health status of live theater actors: A systematic literature review. Medical Problems of Performing Artists 2019. 34 (1) 108-117.

Martinasek, Mary P., McGrogan, Jamie B., and Maysonet, Alisha. A Systematic Review of the Respiratory Effects of Inhalational Marijuana. Respiratory Care 2016. 61 (11) 1543-1551.

Martin-Sanchez, Eva, Furukawa, Toshiaki A., Taylor, Julian, and Martin, Jose Luis. Systematic review and meta-analysis of cannabis treatment for chronic pain. Pain medicine (Malden, Mass.) 2009. 10 (8) 1353-1368.

Matheson, S. L., Shepherd, A. M., and Carr, V. J. How much do we know about schizophrenia and how well do we know it? Evidence from the Schizophrenia Library. Psychological Medicine 2014. 44 (16) 3387-3405.

Matheson, Sandra L., Shepherd, Alana M., Laurens, Kristin R., and Carr, Vaughan J. A systematic meta-review grading the evidence for non-genetic risk factors and putative antecedents of schizophrenia. Schizophrenia Research 2011. 133 (1-3) 133-142.

McDonagh, Marian S., Selph, Shelley S., Buckley, David I., Holmes, Rebecca S., Mauer, Kimberly, Ramirez, Shaun, Hsu, Frances C., Dana, Tracy, Fu, Rochelle, Chou, Roger. 2020.

McLaren, Jennifer A., Silins, Edmund, Hutchinson, Delyse, Mattick, Richard P., and Hall, Wayne. Assessing evidence for a causal link between cannabis and psychosis: a review of cohort studies. The International journal on drug policy 2010. 21 (1) 10-19.

McLoughlin, Benjamin C., Pushpa-Rajah, Jonathan A., Gillies, Donna, Rathbone, John, Variend, Hannele, Kalakouti, Eliana, and Kyprianou, Katerina. Cannabis and schizophrenia. The Cochrane database of systematic reviews 2014. (10) CD004837.

McPartland, John M., Guy, Geoffrey W., and Di Marzo, Vincenzo. Care and feeding of the endocannabinoid system: a systematic review of potential clinical interventions that upregulate the endocannabinoid system. PLoS ONE 2014. 9 (3) e89566.

Mehta, Swati, McIntyre, Amanda, Janzen, Shannon, Loh, Eldon, Teasell, Robert, and Spinal Cord Injury Rehabilitation Evidence Team. Systematic Review of Pharmacologic Treatments of Pain After Spinal Cord Injury: An Update. Archives of physical medicine and rehabilitation 2016. 97 (8) 1381-1391.

Memedovich, K. Ally, Dowsett, Laura E., Spackman, Eldon, Noseworthy, Tom, and Clement, Fiona. The adverse health effects and harms related to marijuana use: an overview review. CMAJ open 2018. 6 (3) E339-E346.

Meng, Howard, Johnston, Bradley, Englesakis, Marina, Moulin, Dwight E., and Bhatia, Anuj. Selective Cannabinoids for Chronic Neuropathic Pain: A Systematic Review and Meta-analysis. Anesthesia and analgesia 2017. 125 (5) 1638-1652.

Merlin, Jessica S., Bulls, Hailey W., Vucovich, Lee A., Edelman, E Jennifer, and Starrels, Joanna L. Pharmacologic and non-pharmacologic treatments for chronic pain in individuals with HIV: a systematic review. AIDS care 2016. 28 (12) 1506-1515.

Meyer, Matthew J., Megyesi, Joseph, Meythaler, Jay, Murie-Fernandez, Manuel, Aubut, Jo Anne, Foley, Norine, Salter, Katherine, Bayley, Mark, Marshall, Shawn, and Teasell, Robert. Acute management of acquired brain injury part II: an evidence-based review of pharmacological interventions. Brain Injury 2010. 24 (5) 706-721.

Meza, Rodrigo, Pena, Javier, Garcia, Karen, Corsi, Oscar, and Rada, Gabriel. Are cannabinoids effective in multiple sclerosis?. Medwave 2017. 17 (Suppl1) e6865.

Millar, Sophie A., Stone, Nicole L., Yates, Andrew S., and O'Sullivan, Saoirse E. A Systematic Review on the Pharmacokinetics of Cannabidiol in Humans. Frontiers in Pharmacology 2018. 9, 1365.

Minozzi, Silvia, Davoli, Marina, Bargagli, Anna M., Amato, Laura, Vecchi, Simona, and Perucci, Carlo A. An overview of systematic reviews on cannabis and psychosis: discussing apparently conflicting results. Drug and alcohol review 2010. 29 (3) 304-317.

Montgomery, LaTrice, Bagot, Kara, Brown, Jennifer L., and Haeny, Angela M. The Association Between Marijuana Use and HIV Continuum of Care Outcomes: a Systematic Review. Current HIV/AIDS Reports 2019. 16 (1) 17-28.

Moore, R. Andrew, Fisher, Emma, Finn, David P., Finnerup, Nanna B., Gilron, Ian, Haroutounian, Simon, Krane, Elliot, Rice, Andrew S. C., Rowbotham, Michael, Wallace, Mark, Eccleston, Christopher. Cannabinoids, cannabis, and cannabis-based medicines for pain management: an overview of systematic reviews. Pain 2020.

Morales, Mariaignacia, Corsi, Oscar, and Pena, Jose. Are cannabinoids effective for the management of chemotherapy induced nausea and vomiting?. Medwave 2017. 17 (9) e7119.

Mucke, Martin, Phillips, Tudor, Radbruch, Lukas, Petzke, Frank, and Hauser, Winfried. Cannabis-based medicines for chronic neuropathic pain in adults. The Cochrane database of systematic reviews 2018. 3, CD012182.

Mullin, Katherine, Gupta, Pal, Compton, Michael T., Nielssen, Olav, Harris, Anthony, and Large, Matthew. Does giving up substance use work for patients with psychosis? A systematic meta-analysis. The Australian and New Zealand journal of psychiatry 2012. 46 (9) 826-839.

Mun, Chung Jung, Letzen, Janelle E., Peters, Erica N., Campbell, Claudia M., Vandrey, Ryan, Gajewski-Nemes, Julia, DiRenzo, Dana, Caufield-Noll, Christine, Finan, Patrick H. Cannabinoid effects on responses to quantitative sensory testing among individuals with and without clinical pain: a systematic review. Pain 2020. 161 (2) 244-260.

Murrie, Benjamin, Lappin, Julia, Large, Matthew, Sara, Grant. Transition of Substance-Induced, Brief, and Atypical Psychoses to Schizophrenia: A Systematic Review and Meta-analysis. Schizophrenia bulletin 2020. 46 (3) 505-516.

Nabata, Kylie J., Tse, Emmanuel K., Nightingale, Tom E., Lee, Amanda H. X., Eng, Janice J., Queree, Matthew, Walter, Matthias, Krassioukov, Andrei V. The Therapeutic Potential and Usage Patterns of Cannabinoids in People with Spinal Cord Injuries: A Systematic Review. Current neuropharmacology 2021. 19 (3) 402-432.

Nader, Danilo A. and Sanchez, Zila M. Effects of regular cannabis use on neurocognition, brain structure, and function: a systematic review of findings in adults. The American journal of drug and alcohol abuse 2018. 44 (1) 4-18.

Nageye, Fatuma and Cortese, Samuele. Beyond stimulants: a systematic review of randomised controlled trials assessing novel compounds for ADHD. Expert Review of Neurotherapeutics 2019.

Navarri, Xavier, Afzali, Mohammad H., Lavoie, Jacob, Sinha, Rajita, Stein, Dan J., Momenan, Reza, Veltman, Dick J., Korucuoglu, Ozlem, Sjoerds, Zsuzsika, van Holst, Ruth J., Hester, Rob, Orr, Catherine, Cousijn, Janna, Yucel, Murat, Lorenzetti, Valentina, Wiers, Reinout, Jahanshad, Neda, Glahn, David C., Thompson, Paul M., Mackey, Scott, Conrod, Patricia J. How do substance use disorders compare to other psychiatric conditions on structural brain abnormalities? A cross-disorder meta-analytic comparison using the ENIGMA consortium findings. Human brain mapping 2020.

Nawrot, Tim S., Perez, Laura, Kunzli, Nino, Munters, Elke, and Nemery, Benoit. Public health importance of triggers of myocardial infarction: a comparative risk assessment. Lancet (London, England) 2011. 377 (9767) 732-740.

Nielsen, Suzanne, Germanos, Rada, Weier, Megan, Pollard, John, Degenhardt, Louisa, Hall, Wayne, Buckley, Nicholas, and Farrell, Michael. The Use of Cannabis and Cannabinoids in Treating Symptoms of Multiple Sclerosis: a Systematic Review of Reviews. Current neurology and neuroscience reports 2018. 18 (2) 8.

Nielsen, Suzanne, Gowing, Linda, Sabioni, Pamela, and Le Foll, Bernard. Pharmacotherapies for cannabis dependence. The Cochrane database of systematic reviews 2019. 1, CD008940.

Ojo, Omorogieva, Wang, Xiao Hua, Ojo, Osarhumwese Osaretin, and Ibe, Jude. The Effects of Substance Abuse on Blood Glucose Parameters in Patients with Diabetes: A Systematic Review and Meta-Analysis. International journal of environmental research and public health 2018. 15 (12).

Okusanya, Babasola O., Asaolu, Ibitola O., Ehiri, John E., Kimaru, Linda Jepkoech, Okechukwu, Abidemi, Rosales, Cecilia. Medical cannabis for the reduction of opioid dosage in the treatment of non-cancer chronic pain: a systematic review. Systematic reviews 2020. 9 (1) 167.

O'Neil, Maya E., Nugent, Shannon M., Morasco, Benjamin J., Freeman, Michele, Low, Allison, Kondo, Karli, Zakher, Bernadette, Elven, Camille, Motu'apuaka, Makalapua, Paynter, Robin, and Kansagara, Devan. Benefits and Harms of Plant-Based Cannabis for Posttraumatic Stress Disorder: A Systematic Review. Annals of Internal Medicine 2017. 167 (5) 332-340.

Oomen, P. P., Van Hell, H. H., and Bossong, M. G. The acute effects of cannabis on human executive function. Behavioural Pharmacology 2018. 29 (7) 605-616.

Orsolini, Laura, Papanti, Gabriele Duccio, De Berardis, Domenico, Guirguis, Amira, Corkery, John Martin, and Schifano, Fabrizio. The "Endless Trip" among the NPS Users: Psychopathology and Psychopharmacology in the Hallucinogen-Persisting Perception Disorder. A Systematic Review. Frontiers in Psychiatry 2017. 8, 240.

Osborne, Ashleigh L., Solowij, Nadia, and Weston-Green, Katrina. A systematic review of the effect of cannabidiol on cognitive function: Relevance to schizophrenia. Neuroscience and Biobehavioral Reviews 2017. 72, 310-324.

Otero-Romero, Susana, Sastre-Garriga, Jaume, Comi, Giancarlo, Hartung, Hans Peter, Soelberg Sorensen, Per, Thompson, Alan J., Vermersch, Patrick, Gold, Ralf, and Montalban, Xavier. Pharmacological management of spasticity in multiple sclerosis: Systematic review and consensus paper. Multiple sclerosis (Houndmills, Basingstoke, England) 2016. 22 (11) 1386-1396.

Pacheco-Colon, Ileana, Limia, Jorge M., and Gonzalez, Raul. Nonacute effects of cannabis use on motivation and reward sensitivity in humans: A systematic review. Psychology of addictive behaviors : journal of the Society of Psychologists in Addictive Behaviors 2018. 32 (5) 497-507.

Papanti, Duccio, Schifano, Fabrizio, Botteon, Giulia, Bertossi, Francesca, Mannix, Jason, Vidoni, Daniela, Impagnatiello, Matteo, Pascolo-Fabrici, Elisabetta, and Bonavigo, Tommaso. "Spiceophrenia": a systematic overview of "spice"-related psychopathological issues and a case report. Human psychopharmacology 2013. 28 (4) 379-389.

Pasha, Ahmed K., Clements, Charlene Y., Reynolds, Charity A., Lopez, Maegan K., Lugo, Ciara A., Gonzalez, Yulisa, Shirazi, Farshad M., Abidov, Aiden. Cardiovascular Effects of Medical Marijuana: A Systematic Review. The American journal of medicine 2021. 134 (2) 182-193.

Patel, Rikinkumar S., Kamil, Saher H., Bachu, Ramya, Adikey, Archana, Ravat, Virendrasinh, Kaur, Mandeep, Tankersley, William E., Goyal, Hemant. Marijuana use and acute myocardial infarction: A systematic review of published cases in the literature. Trends in cardiovascular medicine 2020. 30 (5) 298-307.

Payne, Kelly S., Mazur, Daniel J., Hotaling, James M., and Pastuszak, Alexander W. Cannabis and Male Fertility: A Systematic Review. The Journal of urology 2019., 101097JU0000000000000248.

Pearson, Matthew R. A meta-analytic investigation of the associations between cannabis use and cannabis-related negative consequences. Psychology of addictive behaviors : journal of the Society of Psychologists in Addictive Behaviors 2019. 33 (3) 190-196.

Peng, Yuan Wei, Desapriya, Ediriweera, Chan, Herbert, R Brubacher, Jeffrey. "Residual blood THC levels in frequent cannabis users after over four hours of abstinence: A systematic review.". Drug and alcohol dependence 2020. 216, 108177.

Peters, Erica N., Budney, Alan J., and Carroll, Kathleen M. Clinical correlates of co-occurring cannabis and tobacco use: a systematic review. Addiction (Abingdon, England) 2012. 107 (8) 1404-1417.

Phillips, Tudor J. C., Cherry, Catherine L., Cox, Sarah, Marshall, Sarah J., and Rice, Andrew S. C. Pharmacological treatment of painful HIV-associated sensory neuropathy: a systematic review and meta-analysis of randomised controlled trials. PLoS ONE 2010. 5 (12) e14433.

Pidgeon, Connie and Rickards, Hugh. The pathophysiology and pharmacological treatment of Huntington disease. Behavioural Neurology 2013. 26 (4) 245-253.

Pinto, Jairo Vinicius, Medeiros, Leonardo Simao, Santana da Rosa, Gabriel, Santana de Oliveira, Carlos Eduardo, Crippa, Jose Alexandre de Souza, Passos, Ives Cavalcante, and Kauer-Sant'Anna, Marcia. The prevalence and clinical correlates of cannabis use and cannabis use disorder among patients with bipolar disorder: A systematic review with meta-analysis and meta-regression. Neuroscience and Biobehavioral Reviews 2019. 101, 78-84.

Pinto, Jairo Vinicius, Medeiros, Leonardo Simao, Santana da Rosa, Gabriel, Santana de Oliveira, Carlos Eduardo, Crippa, Jose Alexandre de Souza, Passos, Ives Cavalcante, Kauer-Sant'Anna, Marcia. The prevalence and clinical correlates of cannabis use and cannabis use disorder among patients with bipolar disorder: A systematic review with meta-analysis and meta-regression. Neuroscience and Biobehavioral Reviews 2019. 101, 78-84.

Pittler, Max H. and Ernst, Edzard. Complementary therapies for neuropathic and neuralgic pain: systematic review. The Clinical journal of pain 2008. 24 (8) 731-733.

Pizzol, Damiano, Demurtas, Jacopo, Stubbs, Brendon, Soysal, Pinar, Mason, Corina, Isik, Ahmet Turan, Solmi, Marco, Smith, Lee, Veronese, Nicola. Relationship Between Cannabis Use and Erectile Dysfunction: A Systematic Review and Meta-Analysis. American journal of men's health 2019. 13 (6) 1557988319892464.

Platt, Bradley, O'Driscoll, Ciaran, Curran, Valerie H., Rendell, Peter G., and Kamboj, Sunjeev K. The effects of licit and illicit recreational drugs on prospective memory: a meta-analytic review. Psychopharmacology 2019.

Porr, Caroline J., Rios, Patricia, Bajaj, Harpreet S., Egan, Aoife M., Huot, Celine, Batten, Ryan, Bishop, Lisa, Ryan, Devonne, Davis, Erin, Darvesh, Nazia, Rahman, Arifur, Asghari, Shabnam, Acheampong, Lily, Tricco, Andrea C. The effects of recreational cannabis use on glycemic outcomes and self-management behaviours in people with type 1 and type 2 diabetes: a rapid review. Systematic reviews 2020. 9 (1) 187.

Potvin, Stephane, Joyal, Christian C., Pelletier, Julie, and Stip, Emmanuel. Contradictory cognitive capacities among substance-abusing patients with schizophrenia: a meta-analysis. Schizophrenia Research 2008. 100 (1-3) 242-251.

Pradhan, Ravi R., Pradhan, Shashi R., Mandal, Shobha, and Pradhan, Dhiri R. A Systematic Review of Marijuana Use and Outcomes in Patients with Myocardial Infarction. Cureus 2018. 10 (9) e3333.

Pringsheim, Tamara, Doja, Asif, Gorman, Daniel, McKinlay, Duncan, Day, Lundy, Billinghurst, Lori, Carroll, Alan, Dion, Yves, Luscombe, Sandra, Steeves, Thomas, and Sandor, Paul. Canadian guidelines for the evidence-based treatment of tic disorders: pharmacotherapy. Canadian journal of psychiatry.Revue canadienne de psychiatrie 2012. 57 (3) 133-143.

Pringsheim, Tamara, Holler-Managan, Yolanda, Okun, Michael S., Jankovic, Joseph, Piacentini, John, Cavanna, Andrea E., Martino, Davide, Muller-Vahl, Kirsten, Woods, Douglas W., Robinson, Michael, Jarvie, Elizabeth, Roessner, Veit, and Oskoui, Maryam. Comprehensive systematic review summary: Treatment of tics in people with Tourette syndrome and chronic tic disorders. Neurology 2019. 92 (19) 907-915.

Qian, Yuli, Gurley, Bill J., Markowitz, John S. The Potential for Pharmacokinetic Interactions Between Cannabis Products and Conventional Medications. Journal of clinical psychopharmacology 2019. 39 (5) 462-471.

Qureshi, Abdul Rehman, Rana, Abdul Qayyum, Malik, Suleiman H., Rizvi, Syed Fayyaz, Akhter, Shakib, Vannabouathong, Christopher, Sarfraz, Zainab, and Rana, Ruqqiyah. Comprehensive Examination of Therapies for Pain in Parkinson's Disease: A Systematic Review and Meta-Analysis. Neuroepidemiology 2018. 51 (3-4) 190-206.

Ragazzi, Taciana C. C., Shuhama, Rosana, Menezes, Paulo R., and Del-Ben, Cristina M. Cannabis use as a risk factor for psychotic-like experiences: A systematic review of non-clinical populations evaluated with the Community Assessment of Psychic Experiences. Early Intervention in Psychiatry 2018. 12 (6) 1013-1023.

Rajanahally, S., Raheem, O., Rogers, M., Brisbane, W., Ostrowski, K., Lendvay, T., and Walsh, T. The relationship between cannabis and male infertility, sexual health, and neoplasm: a systematic review. Andrology 2019. 7 (2) 139-147.

Ravi, Divya, Ghasemiesfe, Mehrnaz, Korenstein, Deborah, Cascino, Thomas, and Keyhani, Salomeh. Associations Between Marijuana Use and Cardiovascular Risk Factors and Outcomes: A Systematic Review. Annals of Internal Medicine 2018. 168 (3) 187-194.

Raymundi, Ana Maria, Da Silva, Thiago R., Sohn, Jeferson M. B., Stern, Cristina A., Bertoglio, Leandro J. Effects of DELTA9-tetrahydrocannabinol on aversive memories and anxiety: A review from human studies. BMC Psychiatry 2020. 20 (1) 420.

Reid, Sam, Bhattacharyya, Sagnik. Antipsychotic treatment failure in patients with psychosis and co-morbid cannabis use: A systematic review. Psychiatry research 2019. 280, 112523.

Richards, Bethan L., Whittle, Samuel L., and Buchbinder, Rachelle. Neuromodulators for pain management in rheumatoid arthritis. The Cochrane database of systematic reviews 2012. 1, CD008921.

Richards, John R., Bing, Mary L., Moulin, Aimee K., Elder, Joshua W., Rominski, Robert T., Summers, Phillip J., and Laurin, Erik G. Cannabis use and acute coronary syndrome. Clinical toxicology (Philadelphia, Pa.) 2019., 1-11.

Richards, John R., Blohm, Eike, Toles, Kara A., Jarman, Angela F., Ely, Dylan F., Elder, Joshua W. The association of cannabis use and cardiac dysrhythmias: a systematic review. Clinical toxicology (Philadelphia, Pa.) 2020. 58 (9) 861-869.

Richards, John R., Gordon, Brent K., Danielson, Aaron R., and Moulin, Aimee K. Pharmacologic Treatment of Cannabinoid Hyperemesis Syndrome: A Systematic Review. Pharmacotherapy 2017. 37 (6) 725-734.

Risso, Constanza, Boniface, Sadie, Subbaraman, Meenakshi Sabina, Englund, Amir. Does cannabis complement or substitute alcohol consumption? A systematic review of human and animal studies. Journal of psychopharmacology (Oxford, England) 2020. 34 (9) 938-954.

Rocchetti, Matteo, Crescini, Alessandra, Borgwardt, Stefan, Caverzasi, Edgardo, Politi, Pierluigi, Atakan, Zerrin, and Fusar-Poli, Paolo. Is cannabis neurotoxic for the healthy brain? A meta-analytical review of structural brain alterations in non-psychotic users. Psychiatry and clinical neurosciences 2013. 67 (7) 483-492.

Rocha, Francisco Carlos Machado, Dos Santos Junior, Jair Guilherme, Stefano, Sergio Carlos, and da Silveira, Dartiu Xavier. Systematic review of the literature on clinical and experimental trials on the antitumor effects of cannabinoids in gliomas. Journal of neuro-oncology 2014. 116 (1) 11-24.

Rodriguez, Andres and Zavala, Cynthia. Cannabinoids for the treatment of cannabis abuse disorder. Medwave 2018. 18 (6) e7287.

Rogeberg, Ole and Elvik, Rune. The effects of cannabis intoxication on motor vehicle collision revisited and revised. Addiction (Abingdon, England) 2016. 111 (8) 1348-1359.

Rogeberg, Ole. A meta-analysis of the crash risk of cannabis-positive drivers in culpability studies-Avoiding interpretational bias. Accident; analysis and prevention 2019. 123, 69-78.

Rossi, Giordano Novak, Osorio, Flavia L., Morgan, Celia J. A., Crippa, Jose Alexandre S., Bouso, Jose Carlos, Rocha, Juliana Mendes, Zuardi, Antonio W., Hallak, Jaime E. C., Santos, Rafael G. Dos. The effects of Cannabidiol (CBD) and Delta-9-Tetrahydrocannabinol (THC) on the recognition of emotions in facial expressions: A systematic review of randomized controlled trials. Neuroscience and biobehavioral reviews 2020. 118, 236-246.

Rothbart, Rachel and Stein, Dan J. Pharmacotherapy of trichotillomania (hair pulling disorder): an updated systematic review. Expert Opinion on Pharmacotherapy 2014. 15 (18) 2709-2719.

Ruiz-Veguilla, Miguel, Callado, Luis F., and Ferrin, Maite. Neurological soft signs in patients with psychosis and cannabis abuse: a systematic review and meta-analysis of paradox. Current Pharmaceutical Design 2012. 18 (32) 5156-5164.

Sabe, Michel, Zhao, Nan, Kaiser, Stefan. Cannabis, nicotine and the negative symptoms of schizophrenia: Systematic review and meta-analysis of observational studies. Neuroscience and biobehavioral reviews 2020. 116, 415-425.

Sami, Musa Basseer and Bhattacharyya, Sagnik. Are cannabis-using and non-using patients different groups? Towards understanding the neurobiology of cannabis use in psychotic disorders. Journal of psychopharmacology (Oxford, England) 2018. 32 (8) 825-849.

Sami, Musa Basser, Rabiner, Eugenii A., and Bhattacharyya, Sagnik. Does cannabis affect dopaminergic signaling in the human brain? A systematic review of evidence to date. European neuropsychopharmacology : the journal of the European College of Neuropsychopharmacology 2015. 25 (8) 1201-1224.

Sanadgol, Nima, Zahedani, Shahram Shahraki, Sharifzadeh, Mohammad, Khalseh, Roghayeh, Barbari, Gullam Reza, and Abdollahi, Mohammad. Recent Updates in Imperative Natural Compounds for Healthy Brain and Nerve Function: A Systematic Review of Implications for Multiple Sclerosis. Current Drug Targets 2017. 18 (13) 1499-1517.

Sanchez-Gutierrez, Teresa, Fernandez-Castilla, Belen, Barbeito, Sara, Gonzalez-Pinto, Ana, Becerra-Garcia, Juan Antonio, Calvo, Ana. Cannabis use and nonuse in patients with first-episode psychosis: A systematic review and meta-analysis of studies comparing neurocognitive functioning. European psychiatry : the journal of the Association of European Psychiatrists 2020. 63 (1) e6.

Sandfort, Theodorus G. M., Knox, Justin R., Alcala, Carolina, El-Bassel, Nabila, Kuo, Irene, and Smith, Laramie R. Substance Use and HIV Risk Among Men Who Have Sex With Men in Africa: A Systematic Review. Journal of acquired immune deficiency syndromes (1999) 2017. 76 (2) e34-e46.

Santana, Thaiana Aragao, Trufelli, Damila Cristina, Matos, Leandro Luongo de, Cruz, Felipe Melo, and Del Giglio, Auro. Meta-analysis of adjunctive non-NK1 receptor antagonist medications for the control of acute and delayed chemotherapy-induced nausea and vomiting. Supportive care in cancer : official journal of the Multinational Association of Supportive Care in Cancer 2015. 23 (1) 213-222.

Sarris, Jerome, Sinclair, Justin, Karamacoska, Diana, Davidson, Maggie, Firth, Joseph. Medicinal cannabis for psychiatric disorders: a clinically-focused systematic review. BMC psychiatry 2020. 20 (1) 24.

Schauer, Gillian L., Rosenberry, Zachary R., and Peters, Erica N. Marijuana and tobacco co-administration in blunts, spliffs, and mulled cigarettes: A systematic literature review. Addictive Behaviors 2017. 64, 200-211.

Schlossarek, Samantha, Kempkensteffen, Jurgen, Reimer, Jens, and Verthein, Uwe. Psychosocial Determinants of Cannabis Dependence: A Systematic Review of the Literature. European Addiction Research 2016. 22 (3) 131-144.

Schoeler, T., Kambeitz, J., Behlke, I., Murray, R., and Bhattacharyya, S. The effects of cannabis on memory function in users with and without a psychotic disorder: findings from a combined meta-analysis. Psychological Medicine 2016. 46 (1) 177-188.

Schoeler, Tabea, Monk, Anna, Sami, Musa B., Klamerus, Ewa, Foglia, Enrico, Brown, Ruth, Camuri, Giulia, Altamura, A. Carlo, Murray, Robin, and Bhattacharyya, Sagnik. Continued versus discontinued cannabis use in patients with psychosis: a systematic review and meta-analysis. The lancet.Psychiatry 2016. 3 (3) 215-225.

Schreiner, Amy M. and Dunn, Michael E. Residual effects of cannabis use on neurocognitive performance after prolonged abstinence: a meta-analysis. Experimental and clinical psychopharmacology 2012. 20 (5) 420-429.

Schroder, Sven, Beckmann, Kathrin, Franconi, Giovanna, Meyer-Hamme, Gesa, Friedemann, Thomas, Greten, Henry Johannes, Rostock, Matthias, and Efferth, Thomas. Can medical herbs stimulate regeneration or neuroprotection and treat neuropathic pain in chemotherapy-induced peripheral neuropathy?. Evidence-based complementary and alternative medicine : eCAM 2013. 2013, 423713.

Schuckit, Marc A. Comorbidity of substance use disorders with psychiatric conditions. 2007., 133-155.

Schumacher, Amy, Marzell, Miesha, Toepp, Angela J., and Schweizer, Marin L. Association Between Marijuana Use and Condom Use: A Meta-Analysis of Between-Subject Event-Based Studies. Journal of studies on alcohol and drugs 2018. 79 (3) 361-369.

Schussel, Victor, Kenzo, Lucas, Santos, Andreia, Bueno, Julia, Yoshimura, Ellen, de Oliveira Cruz Latorraca, Carolina, Pachito, Daniela Vianna, and Riera, Rachel. Cannabinoids for nausea and vomiting related to chemotherapy: Overview of systematic reviews. Phytotherapy research : PTR 2018. 32 (4) 567-576.

Semple, D. M., McIntosh, A. M., and Lawrie, S. M. Cannabis as a risk factor for psychosis: Systematic review. Journal of Psychopharmacology 2005. 19 (2) 187-194.

Shilpa. Toxicological and medicolegal aspects of Vijaya (Cannabis sativa Linn.): A systematic review. International Journal of Research in Ayurveda and Pharmacy 2019. 10 (1) 7-10.

Shishko, Ilona, Oliveira, Rosana, Moore, Troy A., and Almeida, Kenneth. A review of medical marijuana for the treatment of posttraumatic stress disorder: Real symptom re-leaf or just high hopes?. The mental health clinician 2018. 8 (2) 86-94.

Sideli, Lucia, Quigley, Harriet, La Cascia, Caterina, Murray, Robin M. Cannabis Use and the Risk for Psychosis and Affective Disorders. Journal of dual diagnosis 2020. 16 (1) 22-42.

Singh, Shalini and Balhara, Yatan Pal Singh. A review of Indian research on co-occurring cannabis use disorders& psychiatric disorders. The Indian journal of medical research 2017. 146 (2) 186-195.

Skalski, Linda M., Towe, Sheri L., Sikkema, Kathleen J., and Meade, Christina S. The Impact of Marijuana Use on Memory in HIV-Infected Patients: A Comprehensive Review of the HIV and Marijuana Literatures. Current Drug Abuse Reviews 2016. 9 (2) 126-141.

Skelley, Jessica W., Deas, Crystal M., Curren, Zachary, Ennis, Jonathan. Use of cannabidiol in anxiety and anxiety-related disorders. Journal of the American Pharmacists Association : JAPhA 2020. 60 (1) 253-261.

Smith, Janette L., Mattick, Richard P., Jamadar, Sharna D., and Iredale, Jaimi M. Deficits in behavioural inhibition in substance abuse and addiction: a meta-analysis. Drug and Alcohol Dependence 2014. 145, 1-33.

Smith, Lesley A., Azariah, Fredric, Lavender, Verna T. C., Stoner, Nicola S., and Bettiol, Silvana. Cannabinoids for nausea and vomiting in adults with cancer receiving chemotherapy. The Cochrane database of systematic reviews 2015. (11) CD009464.

Snedecor, Sonya J., Sudharshan, Lavanya, Cappelleri, Joseph C., Sadosky, Alesia, Desai, Pooja, Jalundhwala, Yash J., and Botteman, Marc. Systematic review and comparison of pharmacologic therapies for neuropathic pain associated with spinal cord injury. Journal of Pain Research 2013. 6, 539-547.

Snedecor, Sonya J., Sudharshan, Lavanya, Cappelleri, Joseph C., Sadosky, Alesia, Mehta, Sonam, and Botteman, Marc. Systematic review and meta-analysis of pharmacological therapies for painful diabetic peripheral neuropathy. Pain practice : the official journal of World Institute of Pain 2014. 14 (2) 167-184.

Song, Ashley, Myung, No Kang, Bogumil, David, Ihenacho, Ugonna, Burg, Madeleine L., Cortessis, Victoria K. Incident testicular cancer in relation to using marijuana and smoking tobacco: A systematic review and meta-analysis of epidemiologic studies. Urologic oncology 2020. 38 (7) 642.e1-642.e9.

Sorensen, Cecilia J., DeSanto, Kristen, Borgelt, Laura, Phillips, Kristina T., and Monte, Andrew A. Cannabinoid Hyperemesis Syndrome: Diagnosis, Pathophysiology, and Treatment-a Systematic Review. Journal of medical toxicology : official journal of the American College of Medical Toxicology 2017. 13 (1) 71-87.

Stevens, A. J. and Higgins, M. D. A systematic review of the analgesic efficacy of cannabinoid medications in the management of acute pain. Acta anaesthesiologica Scandinavica 2017. 61 (3) 268-280.

Stockings, Emily, Bartlem, Kate, Hall, Alix, Hodder, Rebecca, Gilligan, Conor, Wiggers, John, Wolfenden, Luke, Sherker, Shauna. Whole-of-community interventions to reduce population-level harms arising from alcohol and other drug use: a systematic review and meta-analysis. Addiction (Abingdon, England) 2018. 113 (11) 1984-2018.

Stockings, Emily, Campbell, Gabrielle, Hall, Wayne D., Nielsen, Suzanne, Zagic, Dino, Rahman, Rakin, Murnion, Bridin, Farrell, Michael, Weier, Megan, and Degenhardt, Louisa. Cannabis and cannabinoids for the treatment of people with chronic noncancer pain conditions: a systematic review and meta-analysis of controlled and observational studies. Pain 2018. 159 (10) 1932-1954.

Stockings, Emily, Zagic, Dino, Campbell, Gabrielle, Weier, Megan, Hall, Wayne D., Nielsen, Suzanne, Herkes, Geoffrey K., Farrell, Michael, and Degenhardt, Louisa. Evidence for cannabis and cannabinoids for epilepsy: a systematic review of controlled and observational evidence. Journal of neurology, neurosurgery, and psychiatry 2018. 89 (7) 741-753.

Stout, Stephen M. and Cimino, Nina M. Exogenous cannabinoids as substrates, inhibitors, and inducers of human drug metabolizing enzymes: a systematic review. Drug metabolism reviews 2014. 46 (1) 86-95.

Szoke, Andrei, Galliot, Anne Marie, Richard, Jean Romain, Ferchiou, Aziz, Baudin, Gregoire, Leboyer, Marion, and Schurhoff, Franck. Association between cannabis use and schizotypal dimensions--a meta-analysis of cross-sectional studies. Psychiatry Research 2014. 219 (1) 58-66.

Tait, Robert J., Caldicott, David, Mountain, David, Hill, Simon L., and Lenton, Simon. A systematic review of adverse events arising from the use of synthetic cannabinoids and their associated treatment. Clinical toxicology (Philadelphia, Pa.) 2016. 54 (1) 1-13.

Tan, Christina San San, Lee, Shaun Wen Huey. Warfarin and food, herbal or dietary supplement interactions: A systematic review. British journal of clinical pharmacology 2021. 87 (2) 352-374.

Teasell, Robert W., Mehta, Swati, Aubut, Jo Anne, Foulon, Brianne, Wolfe, Dalton L., Hsieh, Jane T. C., Townson, Andrea F., Short, Christine, and Spinal Cord Injury Rehabilitation Evidence Research Team. A systematic review of pharmacologic treatments of pain after spinal cord injury. Archives of physical medicine and rehabilitation 2010. 91 (5) 816-831.

Tetrault, J. M. and Crothers, K. What are the pulmonary effects of smoking marijuana?. Journal of Respiratory Diseases 2007. 28 (6) 253-254.

Toledo-Fernandez, A., Brzezinski-Rittner, A., Roncero, C., Benjet, C., Salvador-Cruz, J., and Marin-Navarrete, R. Assessment of neurocognitive disorder in studies of cognitive impairment due to substance use disorder: A systematic review. Journal of Substance Use 2018. 23 (5) 535-550.

Torres-Moreno, Mari Carmen, Papaseit, Esther, Farre, Magi, Torrens, Marta. Assessment of Efficacy and Tolerability of Medicinal Cannabinoids in Patients With Multiple Sclerosis: A Systematic Review and Meta-analysis. JAMA network open 2018. 1 (6) e183485.

Torres-Moreno, Mari Carmen, Papaseit, Esther, Torrens, Marta, and Farre, Magi. Assessment of Efficacy and Tolerability of Medicinal Cannabinoids in Patients With Multiple Sclerosis: A Systematic Review and Meta-analysis. JAMA network open 2018. 1 (6) e183485.

Tournebize, Juliana, Gibaja, Valerie, and Kahn, Jean Pierre. Acute effects of synthetic cannabinoids: Update 2015. Substance abuse 2017. 38 (3) 344-366.

Tramer, M. R., Carroll, D., Campbell, F. A., Reynolds, D. J., Moore, R. A., and McQuay, H. J. Cannabinoids for control of chemotherapy induced nausea and vomiting: quantitative systematic review. BMJ (Clinical research ed.) 2001. 323 (7303) 16-21.

Turna, Jasmine, Syan, Sabrina K., Frey, Benicio N., Rush, Brian, Costello, Mary Jean, Weiss, Mark, and MacKillop, James. Cannabidiol as a Novel Candidate Alcohol Use Disorder Pharmacotherapy: A Systematic Review. Alcoholism, clinical and experimental research 2019. 43 (4) 550-563.

Twomey, Conal D. Association of cannabis use with the development of elevated anxiety symptoms in the general population: a meta-analysis. Journal of Epidemiology and Community Health 2017. 71 (8) 811-816.

Underner, M., Jaafari, N., Peiffer, G., Perriot, J. Asthma and cannabis, cocaine or heroin use. Revue des Maladies Respiratoires 2020. 37 (7) 572-589.

Underner, M., Urban, T., Perriot, J., Peiffer, G., Harika-Germaneau, G., and Jaafari, N. [Spontaneous pneumothorax and lung emphysema in cannabis users]. Revue de pneumologie clinique 2018. 74 (6) 400-415.

Vaessen, Thomas, Hernaus, Dennis, Myin-Germeys, Inez, and van Amelsvoort, Therese. The dopaminergic response to acute stress in health and psychopathology: A systematic review. Neuroscience and Biobehavioral Reviews 2015. 56, 241-251.

van der Meer, Floor J., Velthorst, Eva, Meijer, Carin J., Machielsen, Marise W. J., and de Haan, Lieuwe. Cannabis use in patients at clinical high risk of psychosis: impact on prodromal symptoms and transition to psychosis. Current Pharmaceutical Design 2012. 18 (32) 5036-5044.

Vaucher, J., Keating, B. J., Lasserre, A. M., Gan, W., Lyall, D. M., Ward, J., Smith, D. J., Pell, J. P., Sattar, N., Pare, G., and Holmes, M. V. Cannabis use and risk of schizophrenia: a Mendelian randomization study. Molecular psychiatry 2018. 23 (5) 1287-1292.

Vyas, Marianne Beare, LeBaron, Virginia T., and Gilson, Aaron M. The use of cannabis in response to the opioid crisis: A review of the literature. Nursing outlook 2018. 66 (1) 56-65.

Walitt, Brian, Klose, Petra, Fitzcharles, Mary Ann, Phillips, Tudor, and Hauser, Winfried. Cannabinoids for fibromyalgia. The Cochrane database of systematic reviews 2016. 7, CD011694.

Walsh, Zach, Gonzalez, Raul, Crosby, Kim, Thiessen, S., Carroll, Chris, and Bonn-Miller, Marcel O. Medical cannabis and mental health: A guided systematic review. Clinical Psychology Review 2017. 51, 15-29.

Wang, Tongtong, Collet, Jean Paul, Shapiro, Stan, and Ware, Mark A. Adverse effects of medical cannabinoids: a systematic review. CMAJ : Canadian Medical Association journal = journal de l'Association medicale canadienne 2008. 178 (13) 1669-1678.

Werneck, Maira Aguiar, Kortas, Guilherme Trevizan, de Andrade, Arthur Guerra, and Castaldelli-Maia, Joao Mauricio. A Systematic Review of the Efficacy of Cannabinoid Agonist Replacement Therapy for Cannabis Withdrawal Symptoms. CNS Drugs 2018. 32 (12) 1113-1129.

Wijarnpreecha, Karn, Panjawatanan, Panadeekarn, and Ungprasert, Patompong. Use of cannabis and risk of advanced liver fibrosis in patients with chronic hepatitis C virus infection: A systematic review and meta-analysis. Journal of evidence-based medicine 2018. 11 (4) 272-277.

Wijayendran, Surapi Bhairavi, O'Neill, Aisling, and Bhattacharyya, Sagnik. The effects of cannabis use on salience attribution: a systematic review. Acta neuropsychiatrica 2018. 30 (1) 43-57.

Wilson, Lorna, Szigeti, Attila, Kearney, Angela, and Clarke, Mary. Clinical characteristics of primary psychotic disorders with concurrent substance abuse and substance-induced psychotic disorders: A systematic review. Schizophrenia Research 2017.

Wisdom, Jennifer P. and Manuel, Jennifer I. Prevalence of Substance Use in People With First-Episode Psychosis. Journal of Dual Diagnosis 2011. 7 (1-2) 39-49.

Xue, Siqi, Husain, M. Ishrat, Zhao, Haoyu, Ravindran, Arun V. Cannabis Use and Prospective Long-Term Association with Anxiety: A Systematic Review and Meta-Analysis of Longitudinal Studies: Usage du cannabis et association prospective a long terme avec l'anxiete: une revue systematique et une meta-analyse d'etudes longitudinales. Canadian journal of psychiatry. Revue canadienne de psychiatrie 2021. 66 (2) 126-138.

Yanes, Julio A., McKinnell, Zach E., Busler, Jessica N., Robinson, Jennifer L., Reid, Meredith A., Michel, Jesse S., Pangelinan, Melissa M., Sutherland, Matthew T., Gonzalez, Raul, Younger, Jarred W. Effects of cannabinoid administration for pain: A meta-analysis and meta-regression. Experimental and Clinical Psychopharmacology 2019. 27 (4) 370-382.

Yanes, Julio A., Riedel, Michael C., Ray, Kimberly L., Kirkland, Anna E., Bird, Ryan T., Boeving, Emily R., Reid, Meredith A., Gonzalez, Raul, Robinson, Jennifer L., Laird, Angela R., and Sutherland, Matthew T. Neuroimaging meta-analysis of cannabis use studies reveals convergent functional alterations in brain regions supporting cognitive control and reward processing. Journal of psychopharmacology (Oxford, England) 2018. 32 (3) 283-295.

Yarnell, Stephanie. The Use of Medicinal Marijuana for Posttraumatic Stress Disorder: A Review of the Current Literature. The primary care companion for CNS disorders 2015. 17 (3).

Yucel, Murat, Bora, Emre, Lubman, Dan I., Solowij, Nadia, Brewer, Warrick J., Cotton, Sue M., Conus, Philippe, Takagi, Michael J., Fornito, Alex, Wood, Stephen J., McGorry, Patrick D., and Pantelis, Christos. The impact of cannabis use on cognitive functioning in patients with schizophrenia: a meta-analysis of existing findings and new data in a first-episode sample. Schizophrenia Bulletin 2012. 38 (2) 316-330.

Zammit, Stanley, Moore, Theresa H. M., Lingford-Hughes, Anne, Barnes, Thomas R. E., Jones, Peter B., Burke, Margaret, and Lewis, Glyn. Effects of cannabis use on outcomes of psychotic disorders: systematic review. The British journal of psychiatry : the journal of mental science 2008. 193 (5) 357-363.

Zantut, Paulo R. A., Veras, Mariana M., Yariwake, Victor Y., Takahashi, Walter Y., Saldiva, Paulo H., Young, Lucy H., Damico, Francisco Max, Fajersztajn, Lais. Effects of cannabis and its components on the retina: a systematic review. Cutaneous and ocular toxicology 2020. 39 (1) 1-9.

Zarei, Shabnam, Salimi, Yahya, Repo, Eveliina, Daglioglu, Nebile, Safaei, Zahra, Guzel, Evsen, Asadi, Anvar. A global systematic review and meta-analysis on illicit drug consumption rate through wastewater-based epidemiology. Environmental science and pollution research international 2020. 27 (29) 36037-36051.

Zhang, Li Rita, Morgenstern, Hal, Greenland, Sander, Chang, Shen Chih, Lazarus, Philip, Teare, M. Dawn, Woll, Penella J., Orlow, Irene, Cox, Brian, Cannabis and Respiratory Disease Research Group of New Zealand, Brhane, Yonathan, Liu, Geoffrey, and Hung, Rayjean J. Cannabis smoking and lung cancer risk: Pooled analysis in the International Lung Cancer Consortium. International journal of cancer 2015. 136 (4) 894-903.

Zhang, Melvyn W. B., Ying, Jiangbo, Wing, Tracey, Song, Guo, Fung, Daniel S. S., and Smith, Helen E. Cognitive Biases in Cannabis, Opioid, and Stimulant Disorders: A Systematic Review. Frontiers in Psychiatry 2018. 9, 376.

Zhang, Melvyn, Ying, Jiangbo, Wing, Tracey, Song, Guo, Fung, Daniel S. S., and Smith, Helen. A Systematic Review of Attention Biases in Opioid, Cannabis, Stimulant Use Disorders. International journal of environmental research and public health 2018. 15 (6).

Zhornitsky, Simon, Pelletier, Julie, Assaf, Roxane, Giroux, Sarah, Li, Chiang-Shan R., Potvin, Stephane. Acute effects of partial CB1 receptor agonists on cognition - A meta-analysis of human studies. Progress in neuro-psychopharmacology & biological psychiatry 2021. 104, 110063.

Zilverstand, Anna, Huang, Anna S., Alia-Klein, Nelly, and Goldstein, Rita Z. Neuroimaging Impaired Response Inhibition and Salience Attribution in Human Drug Addiction: A Systematic Review. Neuron 2018. 98 (5) 886-903.

No current cannabis use

Ghasemiesfe, Mehrnaz, Ravi, Divya, Vali, Marzieh, Korenstein, Deborah, Arjomandi, Mehrdad, Frank, James, Austin, Peter C., and Keyhani, Salomeh. Marijuana Use, Respiratory Symptoms, and Pulmonary Function: A Systematic Review and Meta-analysis. Annals of Internal Medicine 2018. 169 (2) 106-115.

Haddad, Rebecca, Denys, Pierre, Arlandis, Salvador, Giannantoni, Antonella, Del Popolo, Giulio, Panicker, Jalesh N., De Ridder, Dirk, Pauwaert, Kim, Van Kerrebroeck, Philipp E., Everaert, Karel. Nocturia and Nocturnal Polyuria in Neurological Patients: From Epidemiology to Treatment. A Systematic Review of the Literature. European urology focus 2020. 6 (5) 922-934.

Meehan-Atrash, Jiries, Korzun, Tetiana, Ziegler, Aaron. Cannabis Inhalation and Voice Disorders: A Systematic Review. JAMA otolaryngology-- head & neck surgery 2019.

Does not meet criteria of a systematic review

Akinyemi, Edward, Randhawa, Gavin, Longoria, Victor, Zeine, Rana. Medical Marijuana Effects in Movement Disorders, Focus on Huntington Disease; A Literature Review. Journal of pharmacy & pharmaceutical sciences : a publication of the Canadian Society for Pharmaceutical Sciences, Societe canadienne des sciences pharmaceutiques 2020. 23.

Andrzejewski, K., Barbano, R., and Mink, J. Cannabinoids in the treatment of movement disorders: A systematic review of case series and clinical trials. Basal Ganglia 2016. 6 (3) 173-181.

Bonaccorso, Stefania, Ricciardi, Angelo, Zangani, Caroline, Chiappini, Stefania, Schifano, Fabrizio. Cannabidiol (CBD) use in psychiatric disorders: A systematic review. Neurotoxicology 2019. 74, 282-298.

Bougea, Anastasia, Koros, Christos, Simitsi, Athina-Maria, Chrysovitsanou, Chrysa, Leonardos, Athanasios, Stefanis, Leonidas. Medical cannabis as an alternative therapeutics for Parkinsons' disease: Systematic review. Complementary therapies in clinical practice 2020. 39, 101154.

Bravo-Soto, Gonzalo A. and Juri, Carlos. Are cannabinoids effective for Parkinson's disease?. Medwave 2017. 17 (Suppl2) e6974.

Campbell, F. A., Tramer, M. R., Carroll, D., Reynolds, D. J., Moore, R. A., and McQuay, H. J. Are cannabinoids an effective and safe treatment option in the management of pain? A qualitative systematic review. BMJ (Clinical research ed.) 2001. 323 (7303) 13-16.

Darkovska-Serafimovska, Marija, Serafimovska, Tijana, Arsova-Sarafinovska, Zorica, Stefanoski, Sasho, Keskovski, Zlatko, and Balkanov, Trajan. Pharmacotherapeutic considerations for use of cannabinoids to relieve pain in patients with malignant diseases. Journal of Pain Research 2018. 11, 837-842.

Davis, Mellar P., Behm, Bertrand, Mehta, Zankhana, and Fernandez, Carlos. The Potential Benefits of Palmitoylethanolamide in Palliation: A Qualitative Systematic Review. The American journal of hospice & palliative care 2019., 1049909119850807.

Groh, Carla J. Medical Cannabis and Psychiatric Disorders: Implications for Psychiatric Nurses. Journal of the American Psychiatric Nurses Association 2020., 1078390320945791.

Hillen, Jodie Belinda, Soulsby, Natalie, Alderman, Chris, Caughey, Gillian E. Safety and effectiveness of cannabinoids for the treatment of neuropsychiatric symptoms in dementia: a systematic review. Therapeutic advances in drug safety 2019. 10, 2042098619846993.

Inglet, Shannon, Winter, Bradly, Yost, Sarah E., Entringer, Sophia, Lian, Anh, Biksacky, Meryl, Pitt, Renee D., Mortensen, Whitney. Clinical Data for the Use of Cannabis-Based Treatments: A Comprehensive Review of the Literature. Annals of Pharmacotherapy 2020. 54 (11) 1109-1143.

Katz, I., Katz, D., Shoenfeld, Y., and Porat-Katz, B. S. Clinical evidence for utilizing cannabinoids in the elderly. Israel Medical Association Journal 2017. 19 (2) 71-75.

Koppel, Barbara S., Brust, John C. M., Fife, Terry, Bronstein, Jeff, Youssof, Sarah, Gronseth, Gary, and Gloss, David. Systematic review: efficacy and safety of medical marijuana in selected neurologic disorders: report of the Guideline Development Subcommittee of the American Academy of Neurology. Neurology 2014. 82 (17) 1556-1563.

Krishnan, Sarada, Cairns, Ruth, and Howard, Robert. Cannabinoids for the treatment of dementia. The Cochrane database of systematic reviews 2009. (2) CD007204.

Lobos Urbina, Diego and Pena Duran, Jose. Are cannabinoids effective for treatment of pain in patients with active cancer?. Medwave 2016. 16 Suppl 3, e6539.

Paunescu, Horia, Dima, Lorena, Ghita, Isabel, Coman, Laurentiu, Ifteni, Petru Iulian, Fulga, Ion, Coman, Oana Andreia. A Systematic Review of Clinical Studies on the Effect of Psychoactive Cannabinoids in Psychiatric Conditions in Alzheimer Dementia. American journal of therapeutics 2020. 27 (3) e249-e269.

Pocuca, Nina, Walter, T. Jordan, Minassian, Arpi, Young, Jared W., Geyer, Mark A., Perry, William. The Effects of Cannabis Use on Cognitive Function in Healthy Aging: A Systematic Scoping Review. Archives of clinical neuropsychology : the official journal of the National Academy of Neuropsychologists 2020.

Rabgay, Karma, Waranuch, Neti, Chaiyakunapruk, Nathorn, Sawangjit, Ratree, Ingkaninan, Kornkanok, Dilokthornsakul, Piyameth. The effects of cannabis, cannabinoids, and their administration routes on pain control efficacy and safety: A systematic review and network meta-analysis. Journal of the American Pharmacists Association : JAPhA 2020. 60 (1) 225-234.e6.

Rodriguez-Almaraz, Jesus-Eduardo, Chang, Susan, Clarke, Jennifer, Oberheim-Bush, Nancy Ann, Taylor, Jennie, Buerki, Robin, Berger, Mitchel, Zablotska, Lydia, Lobach, Iryna, Butowski, Nicholas. A systematic review and meta-analysis examining the effects of cannabis and its derivatives in adults with malignant CNS tumors. Neuro-oncology practice 2020. 7 (4) 376-383.

Roser, Patrik and Haussleiter, Ida S. Antipsychotic-like effects of cannabidiol and rimonabant: systematic review of animal and human studies. Current Pharmaceutical Design 2012. 18 (32) 5141-5155.

Sawtelle, Lindsey, Holle, Lisa M. Use of Cannabis and Cannabinoids in Patients With Cancer. Annals of Pharmacotherapy 2020.

Scott, Emmi P., Brennan, Emily, Benitez, Andreana. A Systematic Review of the Neurocognitive Effects of Cannabis Use in Older Adults. Current addiction reports 2019. 6 (4) 443-455.

Shin, Sarah, Mitchell, Christine, Mannion, Kelly, Smolyn, Julianne, and Meghani, Salimah H. An Integrated Review of Cannabis and Cannabinoids in Adult Oncologic Pain Management. Pain management nursing : official journal of the American Society of Pain Management Nurses 2018.

Tateo, Sydney. State of the evidence: Cannabinoids and cancer pain-A systematic review. Journal of the American Association of Nurse Practitioners 2017. 29 (2) 94-103.

Wilkinson, Samuel T., Radhakrishnan, Rajiv, and D'Souza, Deepak Cyril. A Systematic Review of the Evidence for Medical Marijuana in Psychiatric Indications. The Journal of clinical psychiatry 2016. 77 (8) 1050-1064.

Zhornitsky, Simon and Potvin, Stephane. Cannabidiol in humans-the quest for therapeutic targets. Pharmaceuticals (Basel, Switzerland) 2012. 5 (5) 529-552.

Randomized controlled trials

Not published in English or French

Dumont, G. J. H. [Nabiximols as a substitute for cannabis]. Nabiximols als substituut voor cannabis? 2020. 164.

Ibera, C., Shalom, B., Saifi, F., Shruder, J., and Davidson, E. EFFECTS OF CANNABIS EXTRACT PREMEDICATION ON ANESTHETIC DEPTH. Harefuah 2018. 157 (3) 162‐166.

Seeling, W., Kneer, L., Buchele, B., Gschwend, J., Maier, L., Nett, C., Simmet, T., Steffen, P., Schneider, M., and Rockemann, M. DELTA9-tetrahydrocannabinol and the opioid receptor agonist piritramide do not act synergistically in postoperative pain. Der Anaesthesist 2006. 55 (4) 391-400.

Testing the administration of cannabinoids in neurological diseases. Ideggyogyaszati szemle 2005. 58 (5-6) 175-176.

Wissel, J., Entner, T., Muller, J., Brenneis, C., Berger, T., and Poewe, W. Nabilone reduces spasticity related pain: a double-blind placebo-controlled cross-over trial. Neurologie und rehabilitation 2004. 10 (4) 187‐216.

Full text not available

Cerny, T., Lueftner, D., Possinger, K., Ernst, G., Ruhstaller, T., Meissner, W., Ko, Y. D., Schnelle, M., Reif, M., and Strasser, F. Oral cannabis - extract (CE) versus delta-9-tetrahydrocannabinol (THC) for patients with cancer-related anorexia (CRA): a randomized, double-blind, placebo-controlled study. Proceedings of the american society of clinical oncology 2003., 730.

Fox, P. and Zajicek, J. A multicentre randomised controlled trial of cannabinoids in multiple sclerosis. JNS 2001. 187 (Suppl 1).

Fox, S. H., Kellett, M., Moore, P., Crossman, A. R., and Brotchie, J. M. A Randomised, Double-Blind, Placebo-Controlled Trial to Assess the Potential of Cannabinoid Receptor Stimulation in the Treatment of Dystonia. Neurology 2001. 56 (8 Suppl 3) A125.

Petro, D. An n-of one clinical trial design for the study of marijuana in treatment of spasticity in multiple sclerosis. JNS 2001. 187 (Suppl 1).

Tan, W., Bourbeau, J., Maltais, F., Hernandez, P., Marciniuk, D., O'Donnell, D., Walker, B., Chapman, K., Fitzgerald, M., and Road, J. Marijuana smoking in the middle-aged and elderly population is associated with rapid decline in FEV1. American journal of respiratory and critical care medicine 2018. 197 (MeetingAbstracts).

Ward, A. S., Haney, S. D., Comer, S. D., Hart, C. L., Foltin, R. W., and Fischman, M. W. Oral THC vs. smoked marijuana: a within subjects comparison of effects. NIDA research monograph 2000.

Conference abstract, letter, commentary, or other non-relevant design

[Author not listed]. Nausea and vomiting associated with HIV therapy are reduced with Marinol. The AIDS reader 2000. 10 (12) 701-702.

[Author not listed]. Cannabinoid agonist shows promise for treating cannabis dependence. Brown university psychopharmacology update 2019. 30 (11) 3‐4.

[Author not listed]. P.4.03 The acute effects of cannabidiol on reward processing in humans: an fMRI study. European Neuropsychopharmacology 2019. 29, S702‐S703.

[Author not listed]. Phase 2 trial shows encouraging target for treatment of cannabis use disorder. Brown university psychopharmacology update 2019. 30 (4) 1‐5.

Abdallah, S., Smith, B., Ware, M., Moore, M., Zhi Li, P., Bourbeau, J., and Jensen, D. Effect of inhaled vaporized cannabis on dynamic airway function, breathlessness and exercise intolerance in adults with advanced COPD: a randomized controlled trial. European Respiratory Journal 2018. 52.

Abrams, Donald I., Guzman, Manuel. Can Cannabis Cure Cancer?. JAMA Oncology 2020. 6 (3) 323-324.

Ackerman, William. Cannabinoids: the changing scientific evidence. The Journal of the Arkansas Medical Society 2003. 99 (9) 278-279.

Actrn. A 2-part, randomized, double-blind, placebo-controlled study of the safety, tolerability, pharmacokinetics and pharmacodynamics of single and multiple ascending doses of FTP 198 in healthy volunteers. http://www.who.int/trialsearch/Trial2.aspx?TrialID=ACTRN12620000181909 2020.

Actrn. A Phase 1 study to evaluate Safety, Tolerability and Pharmacokinetics of Ultramicronized-Palmitoylethanolamide (PEA) in Healthy Subjects. http://www.who.int/trialsearch/Trial2.aspx?TrialID=ACTRN12620000213943 2020.

Actrn. A phase I/II double-blind, randomised controlled trial assessing effect of medicinal cannabis on quality of life and symptom control in advanced cancer. http://www.who.int/trialsearch/Trial2.aspx?TrialID=ACTRN12619001534178 2019.

Actrn. A Randomised, Double-Blind, Vehicle-Controlled Study to Evaluate Safety, Tolerability, and Efficacy of Two Dosage Forms of BTX 1801 Applied Twice Daily for Five Days to the Anterior Nares of Healthy Adults Nasally Colonised with Staphylococcus aureus. http://www.who.int/trialsearch/Trial2.aspx?TrialID=ACTRN12620000456954 2020.

Actrn. A Randomised, Open-label, Cross-over Study to Evaluate the Pharmacokinetics of a Single Dose of two Formulations of Orally Administered Tetrahydrocannabinol/Cannabidiol Combination in Fed Healthy Volunteers. http://www.who.int/trialsearch/Trial2.aspx?TrialID=ACTRN12620000462987 2020.

Actrn. A Randomized, Double-Blind, Placebo-Controlled, Multiple-Dose Study to Assess the Safety and Pharmacokinetics of CHI-936 in Healthy Participants. http://www.who.int/trialsearch/Trial2.aspx?TrialID=ACTRN12619001450101 2019.

Actrn. A single-dose, double-blind, placebo-controlled, randomised, crossover study of an oral cannabis-based medicine (ETC120) on sleep, cognition, and next-day function in adults with primary insomnia disorder. http://www.who.int/trialsearch/Trial2.aspx?TrialID=ACTRN12619000714189 2019.

Actrn. A study of the safety and tolerability of single doses of wafers containing cannabidiol (CBD) that dissolve under the tongue compared to CBD oil that is swallowed in healthy volunteers. http://www.who.int/trialsearch/Trial2.aspx?TrialID=ACTRN12620000891921 2020.

Actrn. A study that evaluates the effectiveness of oral combined THC/CBD for people with advanced cancer experiencing a range of symptoms. http://www.who.int/trialsearch/Trial2.aspx?TrialID=ACTRN12619000037101 2019.

Actrn. An examination into the effects of an integrative treatment for depression and / or anxiety in adults. http://www.who.int/trialsearch/Trial2.aspx?TrialID=ACTRN12619000452190 2019.

Actrn. Cannabidiol (CBD) treatment for insomnia. http://www.who.int/trialsearch/Trial2.aspx?TrialID=ACTRN12620000070932 2020.

Actrn. Proof of concept study on the combined effect of dronabinol and acetazolamide on apnoea hypopnoea index (AHI) in adults with obstructive sleep apnoea. http://www.who.int/trialsearch/Trial2.aspx?TrialID=ACTRN12620000916943 2020.

Actrn. Randomised controlled trial to determine the effects of short-term oral cannabidiol (CBD) ingestion on inflammation, muscle damage and functional recovery following downhill running in healthy untrained individuals. http://www.who.int/trialsearch/Trial2.aspx?TrialID=ACTRN12619001120167 2019.

Actrn. Tetrahydrocannabinol for cancer-related anorexia. http://www.who.int/trialsearch/Trial2.aspx?TrialID=ACTRN12619000491167 2019.

Actrn. The comparison of a NanoCelleâ„¢ cannabis formulation (NanaBisâ„¢) and a cannabis oil formulation derived from the same raw cannabis oil material administered in equal concentration to the oral mucosa in healthy volunteers: a pharmacokinetic, open-label, randomised exploratory study. http://www.who.int/trialsearch/Trial2.aspx?TrialID=ACTRN12619001494123 2019.

Actrn. The effect of a cannabidiol (medicinal cannabis) toothpaste and mouthwash on gingivitis. http://www.who.int/trialsearch/Trial2.aspx?TrialID=ACTRN12619000882123 2019.

Actrn. The effect of cannabidiol (CBD) on exercise physiology and bioenergetics. http://www.who.int/trialsearch/Trial2.aspx?TrialID=ACTRN12620000941965 2020.

Actrn. The effect of cannabidiol (CBD) on simulated driving performance. http://www.who.int/trialsearch/Trial2.aspx?TrialID=ACTRN12619001552178 2019.

Actrn. The effect of medicinal cannabis oil on sleep in adults with insomnia: the Cannabis Sleep Study. http://www.who.int/trialsearch/Trial2.aspx?TrialID=ACTRN12620000220965 2020.

Actrn. The effects of medicinal cannabis on driving performance, sedation and mood. http://www.who.int/trialsearch/Trial2.aspx?TrialID=ACTRN12619000932167 2019.

Actrn. The effects of novel medicinal cannabis formulation IHL-42X on apnoea hypopnea index in adults with suspected or diagnosed mild to moderate obstructive sleep apnoea. http://www.who.int/trialsearch/Trial2.aspx?TrialID=ACTRN12619001103156 2019.

Actrn. Vehicle and Comparator-Controlled, Evaluator-blinded Trial to Evaluate the Safety and Anti-Psoriatic Efficacy of Topical Formulations of BTX 1308 in Subjects with Psoriasis Vulgaris in a Psoriasis Plaque Test. http://www.who.int/trialsearch/Trial2.aspx?TrialID=ACTRN12618001802291 2018.

Adekanmi, O., Freeman, R., Waterfield, M., Waterfield, A., Bishop, R., and Zajicek, J. The effect of cannabinoids on lower urinary tract symptoms in multiple sclerosis: a randomised placebo controlled trial (Cams-Luts study) (Abstract). Proceedings of the international continence society united kingdom 11th annual scientific meeting, bournemouth, united kingdom, 18-19 march 2004., 23.

Aggarwal, S. K. Use of cannabinoids in cancer care: Palliative care. Current Oncology 2016. 23 (Supplement 2) S33-S36.

Ahuja, Abhimanyu S. Cannabis-based treatments as an alternative remedy for epilepsy. Integrative Medicine Research 2019. 8 (3) 200-201.

Alcover, K. C., Oluwoye, O., Kriegel, L., McPherson, S., McDonell, M. G. Impact of first episode psychosis treatment on heavy cannabis use: secondary analysis on RAISE-ETP study. Schizophrenia research 2019. 211, 86‐87.

Alexander, K., Goldberg, J., and Korc-Grodzicki, B. Palliative Care and Symptom Management in Older Patients with Cancer. Clinics in Geriatric Medicine 2016. 32 (1) 45-62.

Anonymous. Erratum: Dose-ranging effect of adjunctive oral cannabidiol vs placebo on convulsive seizure frequency in dravet syndrome: A randomized clinical trial (JAMA Neurology (2020) DOI: 10.1001/jamaneurol.2020.0073). JAMA Neurology 2020. 77 (5) 655.

Astafieva, N., Kobzev, D., Gamova, I., Perfilova, I., Udovichenko, E., and Michailova, I. Allergic sensitization to Cannabis ruderalis: prevalence, clinical and immunologic characteristics, subcutaneous immunotherapy. Allergy 2016. 71, 173‐.

Babalonis, S., Lofwall, M. R., Nuzzo, P. A., Elayi, C., Malcolm, R. J., Haney, M., and Walsh, S. L. Examination of the behavioral effects of oral cannabidiol alone and in combination with smoked marijuana. Drug and Alcohol Dependence 2015. 156, e13.

Ben-Menachem, E., Gunning, B., Arenas Cabrera, C. M., Van Landingham, K., Crockett, J., Taylor, L., Critchley, D., Tayo, B., Morrison, G., and Toledo, M. A phase 2 trial to explore the potential for a pharmacokinetic drug-drug interaction with valproate when in combination with cannabidiol in adult epilepsy patients. Epilepsia 2018. 59, S51‐.

Bhattacharyya, S., Appiah-Kusi, E., Wilson, R., Hird, A., Bossong, M., Valmaggia, L., and McGuire, P. Effect of cannabidiol on paranoid ideation in a virtual reality environment in individuals at ultra high risk of psychosis. Early Intervention in Psychiatry 2016. 10, 74‐.

Birnbaum, Angela. How High Can Patients Get on CBD?. Epilepsy Currents 2019. 19 (6) 382-384.

Boggs, D. L., Gupta, A., D'Souza, D. C., Bielen, K., Thurnauer, H., Nhundu, V., Shearer, L., Rispoli, L., Huguenel, B., and Ranganathan, M. Cannabinoid receptor antagonist treatment of cognitive dysfunction in schizophrenia. Journal of Pharmacy Practice 2015. 28 (3) 321‐.

Boggs, D. L., Ranganathan, M., Luddy, C., Cahill, J., D'Souza, D. C., and Skosnik, P. D. Assessment of the dose-dependent motor effects of intravenous delta-9-tetrahydrocannabinol (THC). Journal of Pharmacy Practice 2016. 29 (3) 272‐.

Bossong, M. G., Schubart, C. D., Van Hell, H. H., Iseger, T. A., Van Saane, W., Jager, G., Van Osch, M. J. P., Jansma, J. M., Kahn, R. S., and Boks, M. P. Genetic modulation of acute effects of delta-9-tetrahydrocannabinol (THC) on resting state brain function. European neuropsychopharmacology. 2014. 24, S303‐S304.

Bossong, M. G., Van Hell, H. H., Jager, G., Brouwer, A., Kahn, R. S., and Ramsey, N. F. Involvement of the endocannabinoid system in associative memory: a pharmacological MRI study. European neuropsychopharmacology. 2009. 19, S308‐S309.

Bossong, M., Wilson, R., Appiah-Kusi, E., Linsen, F., Zelaya, F., Allen, P., McGuire, P., Bhattacharyya, S. O12.7. TREATMENT WITH CANNABIDIOL REDUCES RESTING STATE PERFUSION IN INDIVIDUALS AT CLINICAL HIGH RISK FOR PSYCHOSIS.2019 Congress of the Schizophrenia International Research Society, 10-14 April 2019, Orlando, Florida. Schizophrenia bulletin 2019. 45, S200.

Bouhlal, S., Mewmeyer, M., McDiarmid, G. R., Abulseoud, O. A., Huestis, M. A., and Leggio, L. Effects of smoked, vaporized, and oral cannabis administration on appetitive hormones and relationships with subjective effects: a clinical study. Neuropsychopharmacology 2017. 43, S454‐.

Carley, D. W., Prasad, B., Reid, K. J., Malkani, R., Attarian, H., Abbott, S. M., Vern, B., Xie, H., Yuan, C., and Zee, P. The pace (pharmacotherapy of apnea by cannabimimetic enhancement) clinical trial: characteristics of clinical responders to dronabinol treatment of obstructive sleep apnea. Sleep medicine 2017. 40, e45‐e46.

Carley, D. W., Prasad, B., Reid, K. J., Malkani, R., Attarian, H., Abbott, S., Vern, B., Xie, H., Yuan, C., and Zee, P. C. Dronabinol reduces AHI and daytime sleepiness in patients with moderate to severe obstructive sleep apnea syndrome. Sleep 2017. 40, A207‐A208.

Chagas, M. H. N., Tumas, V., Penna-Pereira, M., a Neto, M., Sobreira, E. T., Eckeli, A. L., Hallak, J. E. C., dos Santos, A. C., Crippa, J. A. S., and Zuardi, A. W. Cannabidiol add-on usual treatment improves the outcome of patients with Parkinson's disease. European neuropsychopharmacology. 2013. 23, S546‐S547.

Chmiel, J., Elborn, S., Constantine, S., and White, B. A double-blind, placebo-controlled phase 2 study in adults with cystic fibrosis of anabasum, a selective cannabinoid receptor type 2 agonist. Pediatric pulmonology 2017. 52, 317‐.

Choi, Sang-Ho, Mou, Yongshan, Silva, Afonso C. Cannabis and Cannabinoid Biology in Stroke. Stroke 2019. 50 (9) 2640-2645.

Colizzi, M., McGuire, P., Giampietro, V., Williams, S., Brammer, M., and Bhattacharyya, S. Delta-9-tetrahydrocannabinol challenge in cannabis users and nonusers differentially affects brain function and behavior: an fMRI study of development of tolerance. Schizophrenia Bulletin 2018. 44, S413‐.

Colizzi, M., McGuire, P., Giampietro, V., Williams, S., Brammer, M., and Bhattacharyya, S. Do cannabis users develop tolerance for the psychoactive effects of delta-9-tetrahydrocannabinol? An fMRI study. European Psychiatry 2018. 48, S333‐.

Cooper, R. E., Williams, E., Seegobin, S., Tye, C., Kuntsi, J., and Asherson, P. Cannabinoids in attention-deficit/hyperactivity disorder: a randomised-controlled trial. European Neuropsychopharmacology 2016. 26, S130‐.

Cortes-Briones, J. A., Cahill, J. D., Skosnik, P. D., Mathalon, D. H., Williams, A., Sewell, R. A., Roach, B. J., Ford, J. M., Ranganathan, M., and D'Souza, D. C. Increased neural noise is related to the psychosis-like effects of Thc. Schizophrenia bulletin. 2015. 41, S16.

Ctri. A randomized, double blind, placebo controlled dose-response relationship study in healthy, adult, human subjects. http://www.who.int/trialsearch/Trial2.aspx?TrialID=CTRI/2019/01/016937 2019.

Ctri. Additional benefit of adding Canabinoid or placebo tablet to antiemtic chemotherapeutic regimen to improvise the nausea vomiting control. http://www.who.int/trialsearch/Trial2.aspx?TrialID=CTRI/2020/03/024106 2020.

Ctri. BA/BE study on Aspart mix 30/70 and NOVOMIX 30. http://www.who.int/trialsearch/Trial2.aspx?TrialID=CTRI/2019/06/019644 2019.

Ctri. Effectiveness of the screening and brief intervention for hazardous and harmful cannabis use among patients on opioid agonist treatment (OAT)coming at the Drug Deaddiction and Treatment Center at PGIMER, Chandigarh in the year 20-21. http://www.who.int/trialsearch/Trial2.aspx?TrialID=CTRI/2020/09/028072 2020.

Ctri. MAGNETIC THERAPY FOR CANNABIS USE. http://www.who.int/trialsearch/Trial2.aspx?TrialID=CTRI/2019/09/021037 2019.

Dahlgren, M. Kathryn, Sagar, Kelly A., Lambros, Ashley M., Smith, Rosemary T., Gruber, Staci A. Urinary Tetrahydrocannabinol After 4 Weeks of a Full-Spectrum, High-Cannabidiol Treatment in an Open-label Clinical Trial. JAMA psychiatry 2021. 78 (3) 335-337.

Davis, M. P. Cannabinoids for chronic pain. Journal of Pain and Palliative Care Pharmacotherapy 2008. 22 (4) 315-316.

Doss, Manoj K., Weafer, Jessica, Gallo, David A., and de Wit, Harriet. DELTA9-Tetrahydrocannabinol at Retrieval Drives False Recollection of Neutral and Emotional Memories. Biological Psychiatry 2018. 84 (10) 743-750.

Drks. Acute and chronic influences of cannabidiol on the ability to regenerate after strength and endurance training. http://www.who.int/trialsearch/Trial2.aspx?TrialID=DRKS00018770 2019.

Drks. Prospective, placebo-controlled, double-blind, randomized, cross-over study for the assessment of the blood concentration-response relationship of cannabidiol (CBD) and tetrahydrocannabinol (THC) after single and repetitive vapor inhalation of CBD-rich legal cannabis products and its potential implication on the driving ability. http://www.who.int/trialsearch/Trial2.aspx?TrialID=DRKS00018836 2019.

D'Souza, D. C., Wray, Y., MacDougall, L., Wilson, A., Genovese, and Krystal, J. H. Cannabinoid-dopamine interactions and psychosis: effects of haloperidol in a cannabinoid (tm)modelº psychosis. Schizophrenia research (abstracts of the VIII international congress on schizophrenia research; 2001 april 28-may 2; british columbia, canada) 2001. 49 (1‐2 Suppl) 252‐253.

Dumont, G., Kramers, C., Sweep, E., Touw, D., Van Hasselt, J., de Kam, M., van Gerven, J., Buitelaar, J., and Verkes, R. J. Cannabis co-administration potentiates MDMA effects on temperature and heart rate. European neuropsychopharmacology. 2009. 19, S188.

Euctr, A. T. A study to evaluate efficay and safety of G-Pen compared to GLUCAGENÂ® HYPOKITÂ®. http://www.who.int/trialsearch/Trial2.aspx?TrialID=EUCTR2018-002661-19-AT 2018.

Euctr, A. T. Cannabidiol in Treatment of Pain due to Attrition of the Knee Joint. http://www.who.int/trialsearch/Trial2.aspx?TrialID=EUCTR2019-003591-40-AT 2020.

Euctr, A. T. The effect of Tetrahydrocannabinol on ocular hemodynamics in patients with primary open angle glaucoma- A Phase II Study. http://www.who.int/trialsearch/Trial2.aspx?TrialID=EUCTR2019-003089-42-AT 2019.

Euctr, C. Z. A study to evaluate the efficacy and safety of nabiximols oromucosal spray as add-on therapy in patients with muscle stiffness due to multiple sclerosis. http://www.who.int/trialsearch/Trial2.aspx?TrialID=EUCTR2019-002623-14-CZ 2019.

Euctr, D. E. A Phase IIa Study to Determine the Safety, Tolerability and Efficacy of a daily oral dose of THX-110 in Adult Patients with Tourette Syndrome (TS). http://www.who.int/trialsearch/Trial2.aspx?TrialID=EUCTR2018-000014-38-DE 2018.

Euctr, D. K. Can-Art Effect and safety of using Canabis derivatives for the treatment of pain in patients with inflammatory Arthritis, such as reumatoid arthritis and ankylosing spondylitis, the latter being a type of arthritis that causes a long term inflammation of the joints of the spine. A randomized, double blinded, placebo controlled trial, i.e. in this drug trial, a control group is given a placebo while another group is given the Cannabis derivative being studied. http://www.who.int/trialsearch/Trial2.aspx?TrialID=EUCTR2017-004226-15-DK 2018.

Euctr, D. K. Cannabis based medication for the treatment of pain generated in the nervous system. http://www.who.int/trialsearch/Trial2.aspx?TrialID=EUCTR2017-005198-38-DK 2018.

Euctr, D. K. The effect of cannabis products on nerve pain and muscle stiffness in patients with multiple sclerosis and in patients with spinal cord injury. http://www.who.int/trialsearch/Trial2.aspx?TrialID=EUCTR2018-002315-98-DK 2018.

Euctr, E. S. A randomized trial to evaluate the safety, tolerability, and pharmacokinetics of GWP42003-P in conjunction with therapeutic hypothermia in neonates with moderate or severe birth asphyxia. http://www.who.int/trialsearch/Trial2.aspx?TrialID=EUCTR2016-000936-17-ES 2019.

Euctr, E. S. An irregular trial in which the identity of those receiving the intervention in twin groups is concealed from both the administrators and subject until the test is completed. The trial is to check the safety and efficacy of GWP42003-P versus Placebo as a joining therapy in Participants with Schizophrenia Experiencing Inadequate Response to Ongoing Antipsychotic Treatment. http://www.who.int/trialsearch/Trial2.aspx?TrialID=EUCTR2019-003369-16-ES 2020.

Euctr, E. S. Clinical study to investigate the efficacy and safety of the test substance BX-1 (dronabinol) for the symptomatic relief of spasticity in patients with multiple sclerosis. http://www.who.int/trialsearch/Trial2.aspx?TrialID=EUCTR2018-000001-23-ES 2018.

Euctr, G. B. A randomised feasibility trial investigating Sativex® for the treatment of the Agitation & Aggression (A/A) in Alzheimer’s Dementia. http://www.who.int/trialsearch/Trial2.aspx?TrialID=EUCTR2020-001056-17-GB 2020.

Euctr, G. B. A trial to test how efficient Nabiximols is for treatment of spacticity in patients with Multiple Sclerosis. http://www.who.int/trialsearch/Trial2.aspx?TrialID=EUCTR2019-002625-29-GB 2020.

Euctr, G. B. CANnabidiol for Behavioural Symptoms in Alzheimerâ€™s Disease. http://www.who.int/trialsearch/Trial2.aspx?TrialID=EUCTR2019-002106-52-GB 2020.

Euctr, G. B. CANnabidiol for Parkinsonâ€™s Disease Psychosis. http://www.who.int/trialsearch/Trial2.aspx?TrialID=EUCTR2019-003623-37-GB 2020.

Euctr, N. L. Early-life stress, the endocannabinoid system, and fear memory extinction. http://www.who.int/trialsearch/Trial2.aspx?TrialID=EUCTR2017-004823-66-NL 2019.

Euctr, N. L. Interaction between opioids and cannabinoids in the treatment of fibromyalgia pain. http://www.who.int/trialsearch/Trial2.aspx?TrialID=EUCTR2019-001861-33-NL 2019.

Euctr, N. L. Vaporised cannabis, driving and cognition. http://www.who.int/trialsearch/Trial2.aspx?TrialID=EUCTR2018-003945-40-NL 2018.

f8mtn, R. B. R. Ingestion of Cannabis oil in Fibromyalgic people. http://www.who.int/trialsearch/Trial2.aspx?TrialID=RBR-2f8mtn 2020.

Fitzcharles, M. A., Shir, Y., Joseph, L., and Ware, M. A. The effects of nabilone on insomnia in fibromyalgia: results of a randomized controlled trial. Arthritis and rheumatism 2009. 60, 1429‐.

Flachenecker, P., Zettl, U., and Henze, T. THC: CBD oromucosal spray (nabiximols) in the long term treatment of multiple sclerosis spasticity. The MOVE 2 long-term study. Multiple sclerosis. 2013. 19 (11 SUPPL. 1) 527.

Foltin, R. W., Lewis, S., Haney, M., Hart, C. L., Ward, A., and Fischman, M. W. Effects ofsmoked marijuana in African-American and non-hispanic caucasian males. Drug and Alcohol Dependence 2001. 63 Suppl 1, 48.

Freeman, R., Adekanmi, O., Waterfield, M., Waterfield, A., Bishop, R., and Zajicek, J. The effect of cannabinoids on lower urinary tract symptoms in multiple sclerosis: a randomised placebo controlled trial (CAMS-LUTS study) (Abstract). Neurourology and Urodynamics 2004. 23 (5/6) 607.

French, J., Thiele, E., Mazurkiewicz-Beldzinska, M., Benbadis, S., Marsh, E., Joshi, C., Roberts, C., Taylor, A., and Sommerville, K. Cannabidiol (CBD) significantly reduces drop seizure frequency in Lennox-Gastaut syndrome (LGS): results of a multi-center, randomized, double-blind, placebo controlled trial (GWPCARE4). Neurology 2017. 88 (16).

fw8kg, R. B. R. Initial study of the effects of cannabidiol in patients with generalized anxiety compared to the effects of an anxiolytic (clonazepan). http://www.who.int/trialsearch/Trial2.aspx?TrialID=RBR-2fw8kg 2020.

Garrido, M., Charlottel, L., Riba, J., Puntes, M., Antonijoan, R. M., Martinez, D., Claramunt, J., Gonzalez, M., Martinez, M., and Martinez, M. I. Low abuse potential and overall subjective effects after the sublingual administration of therapeutic single doses of tetrahydrocannabinol, cannabidiol or the combination of both drugs. Basic and clinical pharmacology and toxicology. 2013. 113, 12‐13.

Green, S., Hindocha, C., Yamamori, Y., Yim, J. L. L., Jones, A. P. M., Walker, H. R., Lewis, G., Howes, O. D., Curran, H. V., and Freeman, T. P. P.3.19 The effects of cannabidiol on cerebral blood flow and its relationship to memory: an arterial spin labelling study. European Neuropsychopharmacology 2019. 29, S693‐.

Grotenhermen, F. Cannabinoids do not reduce objective measurements in muscle spasticity, but people with multiple sclerosis perceive some benefit. Evidence-Based Healthcare 2004. 8 (3) 159-161.

Haney, M., Bedi, G., and Cooper, Z. Chronic naltrexone modulates marijuana's reinforcing subjective and cardiovascular effects. Neuropsychopharmacology 2012. 38, S346.

Harden-Harrison, M. M., Munsell, M. F., Fisch, M. J., Ule, U. J., Saccaro, S., Onitilo, A., Metzner-Sadurski, J. K., Giguere, J. K., and Grunberg, S. M. Dronabinol for the prevention of nausea from cyclophosphamide and/or adriamycin. Supportive Care in Cancer 2012. 20, S209‐S210.

Hartley, S., Vaugier, I., Laraibi, A., Alvarez, J. C., and Quera-Salva, M. A. The effects of cannabis on vigilance and simulated driving. Sleep medicine 2017. 40, e127‐.

Hinchcliff, M. Lenabasum for skin disease in patients with diffuse cutaneous systemic sclerosis. Arthritis & rheumatology (hoboken, N.J.) 2020.

Hindocha, C., Freeman, T. P., Schafer, G., Gardener, C., Morgan, C. J. A., and Curran, H. V. Individual and combined effects of delta-9-tetrahydrocannabinol and cannabidiol on model psychosis and memory function. European neuropsychopharmacology. 2014. 24, S526.

Hjorthoj, C., Fohlmann, A., Mette Larsen, A., Gluud, C., Arendt, M., and Nordentoft, M. CapOpus-intervention study for cannabis use disorder in psychosis. Early Intervention in Psychiatry 2012. 6, 28.

Hobart, J. C. and Zajicek, J. P. Cannabis as a symptomatic treatment for MS: clinically meaningful MUSEC to the stiffness and walking problems of people with MS. Multiple sclerosis. 2012. 18 (4 SUPPL. 1) 247.

Hunault, C. C., Van Der Burgt, E. P. M., Bocker, K. B. E., Kenemans, J. L., de Vries, I., and Meulenbelt, J. A non-linear relationship between THC serum concentration and effects after recreational smoking of cannabis cigarettes containing up to 23% THC. Toxicology letters. 2015. 238 (2 SUPPL. 1) S158.

Hunter, D., Oldfield, G., Tich, N., and Messenheimer, J. Synthetic transdermal cannabidiol for the treatment of knee pain due to osteoarthritis. Osteoarthritis and cartilage 2018. 26, S26‐.

Hurd, Yasmin L. Erratum: Cannabidiol for the reduction of cue-induced craving and anxiety in drug-abstinent individuals with heroin use disorder: A double-blind randomized placebo-controlled trial (American Journal of Psychiatry DOI: 10.1176/appi.ajp.2019.18101191). American Journal of Psychiatry 2020. 177 (7) 641.

Hurd, Yasmin L. Leading the Next CBD Wave-Safety and Efficacy. JAMA psychiatry 2020. 77 (4) 341-342.

Irct20200408046986N. Safety and effectiveness of Satinex buccal spray. http://www.who.int/trialsearch/Trial2.aspx?TrialID=IRCT20200408046986N1 2020.

Irving, P. M., Iqbal, T., Nwokolo, C., Subramanian, S., Bloom, S. L., Prasad, N., Hart, A., Murray, C., Lindsay, J. O., and Taylor, A. A randomised, double-blind, placebo-controlled, parallel group, multi-centred pilot study to assess the symptomatic treatment of ulcerative colitis with cannabidiol. Gastroenterology. 2015. 148 (4 SUPPL. 1) S275.

Irving, P., Iqbal, T., Nwokolo, C., Subramanian, S., Bloom, S., Prasad, N., Hart, A., Murray, C., Lindsay, J., and Taylor, A. Cannabidiol for symptomatic treatment of ulcerative colitis: results from a randomised, double-blind, placebo-controlled, parallel group, multi-centred pilot study. Journal of crohn's and colitis. 2015. 9, S287.

Irving, P., Iqbal, T., Nwokolo, C., Subramanian, S., Bloom, S., Prasad, N., Hart, A., Murray, C., Lindsay, J., and Taylor, A. Trial to assess cannabidiol in the symptomatic treatment of ulcerative colitis. Gut. 2015. 64, A430.

Isrctn. Multi-centre trial of cannabidiol (CBD) for the treatment of Parkinson's disease psychosis. http://www.who.int/trialsearch/Trial2.aspx?TrialID=ISRCTN87895237 2020.

Isrctn. The effect of ALFALIFEâ„¢ in reducing low-grade inflammation. http://www.who.int/trialsearch/Trial2.aspx?TrialID=ISRCTN12319920 2020.

Iversen, L. Cannabinoids in pain management. Few well controlled trials of cannabis exist for systemic review. BMJ (Clinical research ed.) 2001. 323 (7323).

Jadoon, K. A., Ratcliffe, S., O'Sullivan, S. E., and Tan, G. D. Tetrahydrocannabivarin (THCV) improves glycaemic control in Type 2 diabetes. Diabetic medicine. 2014. 31, 67.

Jager, G., Bossong, M. G., Van Hell, H. H., and Ramsey, N. F. Modulatory effects of cannabinoids on memory-related brain activity: a pharmacological MRI study. European neuropsychopharmacology. 2009. 19 (var.pagings) S213.

James, J. S. Marijuana safety study completed: weight gain, no safety problems. AIDS treatment news 2000. (348) 3-4.

Joshi, C., Thiele, E., Marsh, E., French, J., Mazurkiewicz-Beldzinska, M., Benbadis, S., Roberts, C., Taylor, A., and Sommerville, K. Treatment with cannabidiol (CBD) significantly reduces drop and total seizure frequency in lennox-gastaut syndrome (LGS): results of a Multicenter, Randomized, Double-blind, Placebo Controlled Trial (GWPCARE4). Annals of Neurology 2017. 82, S293‐.

Katafygiotis, S., Kavia, R., Gonzales, G., Dimitriadis, F., Malousi, A., Ioannidis, E., Fowler, C. J., Lambropoulos, A., and Apostolidis, A. Do cannabinoid agonists used for the treatment of refractory lower urinary tract symptoms in patients with neurogenic detrusor overactivity have an effect on bladder afferent pathways?. Neurourology and Urodynamics 2011. 30 (6) 846‐847.

Kavia, R., De Ridder, D., Sarantis, N., Constantinescu, C., and Fowler, C. J. Randomised controlled trial of cannabis based medicine (CBM, SATIVEX trademark) to treat detrusor overactivity in multiple sclerosis (Abstract number 94). Neurourology and Urodynamics 2006. 25 (6) 622‐623.

Kayser, R. R., Raskin, M., Snorrason, I., Hezel, D. M., Haney, M., Simpson, H. B. Cannabinoid Augmentation of Exposure-Based Psychotherapy for Obsessive-Compulsive Disorder. Journal of clinical psychopharmacology 2020. 40 (2) 207‐210.

Killestein, J., Hoogervorst, E. L. J., Kalkers, N. F., van Winsen, L. M. L., Uitdehagg, B. M. J., Linssen-Schuurmans, C. D., Staats, P. G. M., Zaadstra, B. M., Gorter, R. W., and Polman, C. H. The effects of orally administred cannabinoids in multiple sclerosis patients: a pilot study. Multiple sclerosis (Houndmills, Basingstoke, England) 2000. 6 (Suppl 1) S28.

Killestein, J., Hoogervorst, E. L. J., Reif, M., Kalkers, N. F., Van Loenen, A. C., Staats, P. G. M., Gorter, R. W., Uitdehaag, B. M. J., and Polman, C. H. Safety, tolerability, and efficacy of orally administered cannabinoids in MS. Neurology 2002. 58 (9) 1404-1407.

Koch, C. D., Xu, L., Curtis, S. A., Roberts, J. D., Bunch, D. R., El-Khoury, J. M. Urinary cannabinoid mass spectrometry profiles differentiate dronabinol from cannabis use. Clinica chimica acta 2020. 510, 515‐521.

Lanctot, K. L., Ruthirakuhan, M., Gallagher, D., Sherman, C., Abraham, E. H., Verhoeff, N. P. L. G., Iaboni, A., Black, S. E., Andreazza, A. C., and Kiss, A. NABILONE SIGNIFICANTLY IMPROVES AGITATION/AGGRESSION IN PATIENTS WITH MODERATE-TO-SEVERE AD: PRELIMINARY RESULTS OF A PLACEBO-CONTROLLED, DOUBLE-BLIND, CROSS-OVER TRIAL. Alzheimer's & dementia 2018. 14 (7) 1385‐.

Lane, S. D., Cherek, D. R., Pietras, C. J., and Steinberg, J. L. Marijuana effects on humans sensitivity to consequences. Drug and Alcohol Dependence 2001. 63 Suppl 1, 87.

Lazaridis, Dovena, Eraikhuemen, Nathaniel, Williams, Kia, Lovince, Judith. Treatment of seizures associated with lennox-gastaut and dravet syndromes: A focus on cannabidiol oral solution. P and T 2019. 44 (5) 255-266.

Leocani, L., Nuara, A., Houdayer, E., Del Carro, U., Straffi, L., Martinelli, V., Rossi, P., Schiavetti, I., Amadio, S., and Sormani, M. P. Effect of THC-CBD oromucosal spray (Sativex) on measures of spasticity in multiple sclerosis: a doubleblind, placebo-controlled, crossover study. Multiple sclerosis (Houndmills, Basingstoke, England) 2014. 20 (1 SUPPL. 1) 498.

Leone, C., Di Stefano, G., Biasiotta, A., La Cesa, S., Piroso, S., Pepe, A., Tartaglia, G., Gori, M. C., Onesti, M., and Inghilleri, M. Dronabinol inhibits nociceptive transmission in humans. A double blind randomized controlled study. Clinical Neurophysiology 2016. 127 (4) e145‐.

Levin, D. N., Dulberg, Z., Chan, A., Hare, G., Mazer, C., and Hong, A. A randomized controlled trial of nabilone for the prevention of postoperative nausea and vomiting in elective surgery. Anesthesia and analgesia 2016. 122 (5) S463‐.

Levy-Cooperman, N., Harrison, S. J., Chen, N. L., and Chakraborty, B. Differences in cannabinoid-induced changes in cognitive performance in light stimulant and cannabis users versus heavy cannabis users. European Neuropsychopharmacology 2012. 22, S403‐S404.

Leweke, F. M., Gerth, C. W., Nolden, B. M., Schreiber, D., Schultze-Lutter, F., and Hellmich, M. Cannabidiol as antipsychotic. European Neuropsychopharmacology 2008. 18, S171.

Leweke, F. M., Hellmich, M., Kranaster, L., and Koethe, D. Cannabidiol as a new type of an antipsychotic: results from a placebo-controlled clinical trial. Biological psychiatry. 2012. 71 (8 SUPPL. 1) 63S.

Leweke, F. M., Rohleder, C., Enning, F., Pahlisch, F., Schaefer, C., Mueller, J. K., Hellmich, M., Koethe, D., and Bumb, J. M. A homeostatic role for the endocannabinoid system in schizophrenia. International journal of neuropsychopharmacology. 2014. 17, 14.

Leweke, M. The endocannabinoid system in schizophrenia-a mechanistically new approach to its pathophysiology and treatment. Schizophrenia bulletin. 2013. 39, S341.

Linares, I., Guimaraes, F. S., Eckeli, A., Zuardi, A. W., Souza, J. D. S., Hallak, J. E. C., and Crippa, J. A. S. Lack of cannabidiol effects on sleep-wake cycle in healthy patients. European neuropsychopharmacology.Conference: 29th european college of neuropsychopharmacology congress, ECNP 2016.Austria.Conference start: 20160917.Conference end: 20160920 2016. 26, S369.

Lus, G., Cantello, R., Danni, M. C., Brini, A., Sarchielli, P., Tassinari, T., and Signoriello, E. “Taste”, a pilot study: palatability and oral cavity tolerability of Sativex and possible improvement measures in multiple sclerosis patients with resistant spasticity. Multiple Sclerosis Journal 2017. 23 (3) 996‐997.

Mazurkiewicz-Beldzinska, M., Thiele, E. A., Benbadis, S., Marsh, E. D., Joshi, C., French, J. A., Roberts, C., Taylor, A., and Sommerville, K. Treatment with cannabidiol (CBD) significantly reduces drop seizure frequency in lennox-gastaut syndrome (LGS): results of a multi-centre, randomised, double-blind, placebocontrolled trial (GWPCARE4). Epilepsia 2017. 58, S55‐.

McGuire, P., Robson, P., Cubala, W., Vasile, D., Morrison, P., Barron, R., Taylor, A., and Wright, S. A randomized controlled trial of cannabidiol in schizophrenia. Schizophrenia Bulletin 2018. 44, S27‐.

Meng, H., Dai, T., Hanlon, J. G., Downar, J., Alibhai, S. M. H., Clarke, H. Cannabis and cannabinoids in cancer pain management. Current opinion in supportive and palliative care 2020. 14 (2) 87‐93.

Mersiades, A., Stockler, M., Simes, J., Kirby, A., Morton, R. L., Wong, N., Bhardwaj, A., Tran, A. D., Tognela, A., Haber, P. S., Lintzeris, N., McGregor, I., Allsop, D. J., Olver, I., Gedye, C., Briscoe, K., Fox, P., Aghmesheh, M., Hahn, C., Grimison, P. Pilot and definitive randomised double-blind placebo-controlled trials evaluating an oral cannabinoid-rich THC/CBD cannabis extract for secondary prevention of chemotherapy-induced nausea and vomiting (CINV). Annals of oncology : official journal of the European Society for Medical Oncology 2018. 29 (Supplement 8).

Messenheimer, J. A., O'Brien, T., Berkovic, S., French, J., Bonn-Miller, M., and Gutterman, D. Transdermal cannabidiol (CBD) gel for the treatment of focal epilepsy in adults. Neurology 2018. 90 (24) e2188‐.

Michaud, J., Lefebvre, M., Mathieu, S., Papageorgiou, A., Wood, G., Holmes, I., Sicard, E., and Chamberland, G. A first-in-human trial comparing pharmacokinetics, pharmacodynamics and safety of cannabis following multiple, ascending doses of dried tabletsdelivered by smoking inhalation. Clinical pharmacology in drug development 2018. 7, 36‐37.

Montebello, M., Allsop, D., Copeland, J., Lintzeris, N., Dunlop, A., Sadler, C., Holland, G., Muhleisen, P., Norberg, M., and McGregor, I. Cannabinoid replacement therapy for management of cannabis withdrawal: a randomized controlled trial of nabiximols (Sativex®). Australian and New Zealand Journal of Psychiatry 2014. 48, 79‐.

Mueller, J. K., Reuter, A. R., Lange, B., Schaefer, A., Hanke, F., Pahlisch, F., Schaefer, C., Schmidt, A. M., Woelfl, T., and Enning, F. Effects and interaction of delta-9-tetrahydrocannabidiol and cannabidiol on psychopathology, neurocognition, and endocannabinoids in serum of healthy volunteers: influence on psychopathology. Neuropsychopharmacology 2016. 41, S589‐.

Muller-Vahl, Kirsten R. Cannabinoids reduce symptoms of Tourette's syndrome. Expert Opinion on Pharmacotherapy 2003. 4 (10) 1717-1725.

Naftali, T., Bar Lev Schlieder, L., Sklerovsky Benjaminov, F., Lish, I., Hirsch, J., and Konikoff, F. M. Cannabis induces clinical and endoscopic improvement in moderately active ulcerative colitis (UC). Journal of Crohn's & colitis 2018. 12, S306‐.

Naftali, T., Bar-Lev Schlieder, L., Konikoff, F., Benjaminov, F., Lish, I., Sergeev, I., and Ringel, Y. Cannabis induces clinical response but no endoscopic response in Crohn's disease patients. United european gastroenterology journal 2018. 6 (8) A75‐.

Naftali, T., Bar-Lev, L., Gabay, G., Chowers, Y., Dotan, I., Bronshtein, M., Stein, A., and Konikoff, F. M. Tetrahydrocannabinol (THC) rich medical cannabis induces clinical and biochemical improvement with a steroid sparing effect in active crohn's disease. Gastroenterology. 2012. 142 (5 SUPPL. 1) S780.

Naftali, T., Barlev, L., Gabay, G., Chowers, Y., Dotan, I., Stein, A., Bronstein, M., and Konikoff, F. M. Tetrahydrocannabinol (THC) induces clinical and biochemical improvement with a steroid sparing effect in active inflammatory bowel disease. Journal of Crohn's & colitis 2013. 7, S153.

Naftali, T., Mechoulam, R., Gabay, G., Stein, A., Bronshtein, M., Mari, A., and Konikoff, F. M. Cannabidiol treatment does not effect active crohn's disease. Gastroenterology. 2013. 144 (5 SUPPL. 1) S180.

Nct. A Clinical Trial of a Hemp-Derived Cannabidiol Product for Anxiety. https://clinicaltrials.gov/show/NCT04286594 2020.

Nct. A Phase 2a Study to Evaluate the Safety, Tolerability and Efficacy of Cannabidiol as a Steroid-sparing Therapy in Steroid-dependent Crohn's Disease Patients. https://clinicaltrials.gov/show/NCT04056442 2019.

Nct. A Prospective, Randomized, Double Blind Trial Comparing Dronabinol to a Placebo in the Management of Post-operative Pain in Total Joint Arthroplasty. https://clinicaltrials.gov/show/NCT04298528 2020.

Nct. A Study Investigating the Bioavailability of CBD and THC in an Emulsion Product in a Healthy Population. https://clinicaltrials.gov/show/NCT04601207 2020.

Nct. A Study of Sativex® for Pain Relief of Peripheral Neuropathic Pain, Associated With Allodynia. https://clinicaltrials.gov/show/NCT00710554 2008.

Nct. A Study to Assess the Effect of Cannabidiol Oil on Pain After Ureteroscopy for Kidney Stones. https://clinicaltrials.gov/show/NCT04387617 2020.

Nct. A Study to Investigate the Efficacy and Safety of Cannabidiol (GWP42003-P; CBD) as Adjunctive Treatment for Seizures Associated With Lennox-Gastaut Syndrome in Children and Adults. https://clinicaltrials.gov/show/NCT02224690 2014.

Nct. A Study to Test the Pharmacodynamic, Pharmacokinetic, Safety, and Tolerability of Padsevonil in Healthy Study Participants Receiving Either Ethanol or Cannabidiol. https://clinicaltrials.gov/show/NCT04039919 2019.

Nct. Achieving Cannabis Cessation-Evaluating N-Acetylcysteine Treatment. https://clinicaltrials.gov/show/NCT01675661 2012.

Nct. Adapted Cognitive/Affective Remediation for Cannabis Misuse in Schizophrenia. https://clinicaltrials.gov/show/NCT01292577 2011.

Nct. Age Differences in the Effects of Cannabis on Simulated Driving. https://clinicaltrials.gov/show/NCT04325958 2020.

Nct. Age-Related Effects of THC. https://clinicaltrials.gov/show/NCT04294966 2020.

Nct. Analgesic Effects of Cannabidiol for Simple Tooth Extractions in Dental Patients. https://clinicaltrials.gov/show/NCT04271917 2020.

Nct. Behavioral Pharmacology of Cannabis and Nicotine. https://clinicaltrials.gov/show/NCT04124432 2019.

Nct. Behavioral Pharmacology of THC and Alpha-pinene. https://clinicaltrials.gov/show/NCT04130633 2019.

Nct. Bioequivalence Assessment of Cannabidiol (CBD) Administrated in Oral Formulations. https://clinicaltrials.gov/show/NCT03877991 2019.

Nct. Burnout and Distress preventiOn With caNnabidiol in Front-line Health Care workerS deAling wIth COVID-19. https://clinicaltrials.gov/show/NCT04504877 2020.

Nct. CAN BREATHE in COPD Trial. https://clinicaltrials.gov/show/NCT03060993 2017.

Nct. CANDIS - Targeted Treatment for Cannabis Disorders. https://clinicaltrials.gov/show/NCT00252980 2005.

Nct. Cannabidiol (CBD) for the Treatment of Alcohol Withdrawal. https://clinicaltrials.gov/show/NCT04205682 2019.

Nct. Cannabidiol and CES1 Interactions in Healthy Subjects. https://clinicaltrials.gov/show/NCT04603391 2020.

Nct. Cannabidiol and Management of Endometriosis Pain. https://clinicaltrials.gov/show/NCT04527003 2020.

Nct. Cannabidiol and Oral Contraceptive Pills: exploring a Drug-Drug Interaction. https://clinicaltrials.gov/show/NCT04396730 2020.

Nct. CANnabiDiol for CoviD-19 pATiEnts With Mild to Moderate Symptoms. https://clinicaltrials.gov/show/NCT04467918 2020.

Nct. Cannabidiol for Inflammatory Bowel Disease. https://clinicaltrials.gov/show/NCT01037322 2009.

Nct. Cannabidiol for PTSD and TBI. https://clinicaltrials.gov/show/NCT04550377 2020.

Nct. Cannabidiol for Treatment of Recent-onset Psychosis With Comorbid Cannabis Use. https://clinicaltrials.gov/show/NCT04105231 2019.

Nct. Cannabidiol in Patients With COVID-19 and Cardiovascular Disease or Risk Factors. https://clinicaltrials.gov/show/NCT04615949 2020.

Nct. Cannabidiol Treatment of Cognitive Dysfunction in Schizophrenia. https://clinicaltrials.gov/show/NCT00588731 2007.

Nct. Cannabidiol, Morphine, Pain. https://clinicaltrials.gov/show/NCT04030442 2019.

Nct. Cannabinoid Supplementation on Vascular and Cognitive Function. https://clinicaltrials.gov/show/NCT03295903 2017.

Nct. Cannabinoids and an Anti-inflammatory Diet for the Treatment of Neuropathic Pain After Spinal Cord Injury. https://clinicaltrials.gov/show/NCT04057456 2019.

Nct. Cannabinoids and Cerebellar-Motor Functioning. https://clinicaltrials.gov/show/NCT01853020 2013.

Nct. Cannabinoids for Taxane Induced Peripheral Neuropathy. https://clinicaltrials.gov/show/NCT03782402 2018.

Nct. Cannabinoids for the Treatment of Anxiety Disorders: an 8-Week Pilot Study. https://clinicaltrials.gov/show/NCT04569760 2020.

Nct. Cannabinoids in PLWHIV on Effective ART. https://clinicaltrials.gov/show/NCT03550352 2018.

Nct. Cannabinoids, Neural Synchrony, and Information Processing. https://clinicaltrials.gov/show/NCT00708994 2008.

Nct. Cannabis Effects as a Function of Sex (CanSex). https://clinicaltrials.gov/show/NCT04385082 2020.

Nct. Cannabis Effects on Electroencephalography. https://clinicaltrials.gov/show/NCT04316598 2020.

Nct. Cannabis for Acute Migraine: a Randomized, Double-blind, Placebo-controlled, Crossover Trial. https://clinicaltrials.gov/show/NCT04360044 2020.

Nct. Cannabis For Cancer-Related Symptoms. https://clinicaltrials.gov/show/NCT03948074 2019.

Nct. Cannabis for Inflammatory Bowel Disease. https://clinicaltrials.gov/show/NCT01040910 2009.

Nct. Cannabis for the Prophylactic Treatment of Migraine. https://clinicaltrials.gov/show/NCT03972124 2019.

Nct. Cannabis Impairment Detection Application (CIDA). https://clinicaltrials.gov/show/NCT04230460 2020.

Nct. Cannabis Use Disorder Treatment Study. https://clinicaltrials.gov/show/NCT04567394 2020.

Nct. CBD in Opioid Use and Chronic Pain. https://clinicaltrials.gov/show/NCT04587791 2020.

Nct. CBD Oil for Reducing Emotional Impact of COVID-19. https://clinicaltrials.gov/show/NCT04603781 2020.

Nct. CBD-Microglia PET Study. https://clinicaltrials.gov/show/NCT04398719 2020.

Nct. Characterization of the Pharmacodynamic Response to Vaped THC. https://clinicaltrials.gov/show/NCT04340700 2020.

Nct. CHI-902 for Treatment of Social Anxiety Disorder. https://clinicaltrials.gov/show/NCT04086342 2019.

Nct. CHI-907 CBD Extract and Experiences of Test Anxiety. https://clinicaltrials.gov/show/NCT04269252 2020.

Nct. COGNITIVE BEHAVIORAL THERAPY PROGRAM TO FIRST-EPISODE PSYCHOSIS PATIENTS AND CANNABIS ABUSE. https://clinicaltrials.gov/show/NCT02319746 2014.

Nct. Combination of an Investigational Cannabinoid and Methadone for HIV-associated Neuropathy. https://ClinicalTrials.gov/show/NCT00723918 2009.

Nct. Combined Pharmacotherapy for Cannabis Dependency. https://clinicaltrials.gov/show/NCT01020019 2009.

Nct. Comparative Analysis of the Effectiveness of the Use of Nimesulide and CBD Oil in Patients With Pain in the Preauricular Region Due to the Pain-dysfunctional Syndrome of the Temporomandibular Joint. https://clinicaltrials.gov/show/NCT04609748 2020.

Nct. Comparison of Cannabinoids to Placebo in Management of TMJ Pain and Myofascial Pain in the TMJ Region. https://clinicaltrials.gov/show/NCT04298554 2020.

Nct. Developing a Mobile Method to Measure THC-induced Impairment. https://clinicaltrials.gov/show/NCT03804840 2019.

Nct. Dexanabinol in Severe Traumatic Brain Injury. https://clinicaltrials.gov/show/NCT00129857 2005.

Nct. Do Discounted Vouchers for Medical Cannabis Reduce Opioid Use in Adults With Pain. https://clinicaltrials.gov/show/NCT04495725 2020.

Nct. Dronabinol for Pain and Inflammation in Adults Living With Sickle Cell Disease. https://clinicaltrials.gov/show/NCT03978156 2019.

Nct. Dronabinol for Post-operative Pain After Lumbar Fusion. https://clinicaltrials.gov/show/NCT04346407 2020.

Nct. Effect of an Emollient Cream Containing 0.5% Cannabidiol and 1% Hemp Oil in the Hydration and Erythema of the Skin. https://clinicaltrials.gov/show/NCT04045314 2019.

Nct. Effect of Cannabinoid Agonist on Gastrointestinal and Colonic Motor Functions in Patients With Irritable Bowel Syndrome (IBS). https://clinicaltrials.gov/show/NCT01253408 2010.

Nct. Effect of Cannabis and Endocannabinoids on HIV Neuropathic Pain. https://ClinicalTrials.gov/show/NCT03099005 2018.

Nct. Effect of Hemp-CBD on Patients With CIPN. https://clinicaltrials.gov/show/NCT04398446 2020.

Nct. Effect of Non-psychoactive Cannabidiol as an Adjunct to Botulinum Toxin in Blepharospasm. https://clinicaltrials.gov/show/NCT04423341 2020.

Nct. Effect of Topical CBD Cream for Degenerative Hallux Disorders. https://clinicaltrials.gov/show/NCT04103814 2019.

Nct. Effectiveness Study of Dronabinol and BRENDA for the Treatment of Cannabis Withdrawal. https://clinicaltrials.gov/show/NCT00480441 2007.

Nct. Effects of Cannabidiol (CBD) Versus Placebo as an Adjunct to Treatment in Early Psychosis. https://clinicaltrials.gov/show/NCT04411225 2020.

Nct. Effects of Cannabis Administration Routes on Human Performance and Pharmacokinetics. https://clinicaltrials.gov/show/NCT02177513 2014.

Nct. Effects of Cannabis on Cognition and Endocannabinoid Levels in Bipolar Disorder Patients and Healthy Volunteers. https://clinicaltrials.gov/show/NCT04231643 2020.

Nct. Effects of CHI-554 CBD on Fear. https://clinicaltrials.gov/show/NCT04577612 2020.

Nct. Effects of Citicoline on Brain Function and Behavior in Marijuana-Dependent Individuals. https://clinicaltrials.gov/show/NCT00158249 2005.

Nct. Effects of Delta9-tetrahydrocannabinol (THC) on Retention of Memory for Fear Extinction Learning in PTSD: R33 Study. https://clinicaltrials.gov/show/NCT04080427 2019.

Nct. Effects of Dronabinol in Opioid Maintained Patients. https://clinicaltrials.gov/show/NCT04025359 2019.

Nct. Effects of Hemp Oil on Markers of Optimal Wellness, Stress Resilience and Recovery in Healthy Subjects. https://clinicaltrials.gov/show/NCT04294706 2020.

Nct. Effects of THC on Emotional Memory Retrieval. https://clinicaltrials.gov/show/NCT03471585 2018.

Nct. Effects of THC-Free CBD Oil on Agitation in Patients With Alzheimer's Disease. https://clinicaltrials.gov/show/NCT04436081 2020.

Nct. Efficacy and Safety of Dronabinol in the Improvement of Chemotherapy-induced and Tumor-related Symptoms in Advanced Pancreatic Cancer. https://clinicaltrials.gov/show/NCT03984214 2019.

Nct. Efficacy and Safety of GWP42003-P for Seizures Associated With Lennox-Gastaut Syndrome in Children and Adults. https://clinicaltrials.gov/show/NCT02224560 2014.

Nct. Efficacy and Safety of Surinabant Treatment as an Aid to Smoking Cessation (SURSMOKE). https://clinicaltrials.gov/show/NCT00432575 2007.

Nct. Efficacy of a Controlled Short-term Trial of Cannabidiol (CBD) Ingestion on Reducing Symptomatic Response and Facilitating Recovery After Induced Muscle Injury. https://clinicaltrials.gov/show/NCT04586712 2020.

Nct. Efficacy of Cannabidiol in Knee Osteoarthritis. https://clinicaltrials.gov/show/NCT04607603 2020.

Nct. Evaluation of the Effect of tDCS on Cannabis Craving. https://clinicaltrials.gov/show/NCT04389528 2020.

Nct. Eye Tracking as a Biomarker of Cannabis Effects. https://clinicaltrials.gov/show/NCT04100590 2019.

Nct. FALCON: a Multicenter Randomized Controlled Trial. https://clinicaltrials.gov/show/NCT02558556 2015.

Nct. Impact of Cannabis on Pain and Inflammation Among Patients With Rheumatoid or Psoriatic Arthritis. https://clinicaltrials.gov/show/NCT04269993 2020.

Nct. Impact of Reduced Cannabis Use on Functional Outcomes (R33 Phase). https://clinicaltrials.gov/show/NCT04567238 2020.

Nct. Influence of a Medicinal Cannabinoid Agonist on Responses to Food Images and Food Intake. https://clinicaltrials.gov/show/NCT02310347 2014.

Nct. Interactions Between Cannabinoids and Cytochrome P450-Metabolized Drugs. https://clinicaltrials.gov/show/NCT04201197 2019.

Nct. Investigating the Acute Effects of THC on Functional Brain Systems. https://clinicaltrials.gov/show/NCT00628706 2008.

Nct. Investigating the Effect of Dronabinol on Post-surgical Pain. https://clinicaltrials.gov/show/NCT04099355 2019.

Nct. Marijuana for HIV-Related Peripheral Neuropathy. https://ClinicalTrials.gov/show/NCT00046722 2003.

Nct. Mechanisms of CBD for Chronic Pain. https://clinicaltrials.gov/show/NCT04044729 2019.

Nct. Medicinal Cannabis for Painful HIV Neuropathy. https://ClinicalTrials.gov/show/NCT00255580 2001.

Nct. MUltiple Sclerosis and Extract of Cannabis (MUSEC) Study. https://clinicaltrials.gov/show/NCT00552604 2007.

Nct. Myorelaxant Effect of Cannabis Cream Topical Skin Application in Patients With TMD. https://clinicaltrials.gov/show/NCT03994640 2019.

Nct. Nabilone Effect on the Attenuation of Anorexia, Nutritional Status and Quality of Life in Lung Cancer Patients. https://clinicaltrials.gov/show/NCT02802540 2016.

Nct. Nabilone for Agitation Blinded Intervention Trial. https://clinicaltrials.gov/show/NCT04516057 2020.

Nct. N-Acetylcysteine for Smoking Cessation in Tobacco and Cannabis Co-Use. https://clinicaltrials.gov/show/NCT04627922 2020.

Nct. Neuroimaging and CBD for Heroin Use Disorder. https://clinicaltrials.gov/show/NCT04567784 2020.

Nct. Neuroprotection by Cannabinoids in Huntington's Disease. https://clinicaltrials.gov/show/NCT01502046 2011.

Nct. Oral Cannabidiol for Opioid Withdrawal. https://clinicaltrials.gov/show/NCT04238754 2020.

Nct. Orexigenic Therapy With Delta-9-tetrahydrocannabinol in Advanced Cancer Patients With Chemosensory Abnormalities - a Pilot Study. https://clinicaltrials.gov/show/NCT00316563 2006.

Nct. Pain Response to Cannabidiol in Induced Acute Nociceptive Pain, Allodynia and Hyperalgesia By Using a Model Mimicking Acute Pain in Healthy Adults. https://clinicaltrials.gov/show/NCT03985995 2019.

Nct. Pain Response to Cannabidiol in Opioid-induced Hyperalgesia, Acute Nociceptive Pain and Allodynia By Using a Model Mimicking Acute Pain in Healthy Adults. https://clinicaltrials.gov/show/NCT04059978 2019.

Nct. Pharmacokinetic and Pharmacodynamic Effects of Smoked and Vaporized Cannabis. https://clinicaltrials.gov/show/NCT03676166 2018.

Nct. Pharmacokinetics of CBD From a Hennep Extract. https://clinicaltrials.gov/show/NCT04589455 2020.

Nct. Phase II Clinical Trial, Use of KL16-012 in Women With Fibromyalgia Refractary to Conventional Treatment. https://clinicaltrials.gov/show/NCT04239469 2020.

Nct. Processing and Effects of Cannabis. https://clinicaltrials.gov/show/NCT00225407 2005.

Nct. Prospective Blinded Randomized Controlled Trial Evaluating the Outcomes of Cannabinoid (CBD) Roll-on Topical Stick in Primary Total Knee Arthroplasty. https://clinicaltrials.gov/show/NCT04585230 2020.

Nct. RCT of CBD for Anxiety in Advanced Breast Cancer. https://clinicaltrials.gov/show/NCT04482244 2020.

Nct. Reducing Cannabis Use for Sleep Among Adults Using Medical Cannabis. https://clinicaltrials.gov/show/NCT03964974 2019.

Nct. Safety and Effectiveness of Nabiximols Oromucosal Spray as Add-on Therapy in Participants With Spasticity Due to Multiple Sclerosis. https://clinicaltrials.gov/show/NCT04203498 2019.

Nct. Safety and Effects on Responses to Stress and Pain of Natural Medical Marijuana Products. https://clinicaltrials.gov/show/NCT04226690 2020.

Nct. Safety and Efficacy of a FAAH-Inhibitor to Treat Cannabis Withdrawal. https://clinicaltrials.gov/show/NCT01618656 2012.

Nct. Safety and Efficacy of Inhaled Cannabis (Synthetic THC/CBD) for Improving Quality of Life in Advanced Cancer Patients. https://clinicaltrials.gov/show/NCT04001010 2019.

Nct. Safety and Efficacy of Inhaled Cannabis For the Uncontrolled Pain Releif in Patients With Advanced Cancer. https://clinicaltrials.gov/show/NCT04042545 2019.

Nct. Safety and Efficacy of Nabilone in Alzheimer's Disease. https://clinicaltrials.gov/show/NCT02351882 2015.

Nct. Safety and Efficacy on Spasticity Symptoms of a Cannabis Sativa Extract in Motor Neuron Disease. https://clinicaltrials.gov/show/NCT01776970 2013.

Nct. Safety and Efficacy Study of Dronabinol to Treat Obstructive Sleep Apnea. https://clinicaltrials.gov/show/NCT01755091 2012.

Nct. Safety and Pharmacokinetics of Smoked Cannabis in Healthy Patients. https://clinicaltrials.gov/show/NCT04314804 2020.

Nct. Study of Prevention of Postoperative Nausea and Vomiting Using Cesamet. https://clinicaltrials.gov/show/NCT02115529 2014.

Nct. Study of the Safety and Efficacy of Nabiximols Oromucosal Spray Versus Placebo in Patients With Post-traumatic Stress Disorder. https://clinicaltrials.gov/show/NCT04592159 2020.

Nct. Study to Assess Food Effect on Sativex Bioavailability. https://clinicaltrials.gov/show/NCT01322464 2011.

Nct. Sublingual Cannabidiol for Chronic Pain. https://clinicaltrials.gov/show/NCT03984565 2019.

Nct. THC and Ketamine Effects in Humans: relation to Neural Oscillations and Psychosis. https://clinicaltrials.gov/show/NCT04199468 2019.

Nct. THC Crossover Study. https://clinicaltrials.gov/show/NCT04429568 2020.

Nct. The Analgesic Efficacy of Δ9-THC (Namisol®) in Patients With Persistent Postsurgical Abdominal Pain. https://clinicaltrials.gov/show/NCT01562483 2012.

Nct. The CBD-IC Randomized Controlled Trial: evaluation of Hemp Cannabidiol SuppositoRies for Pain and Urinary SymptOms in INterstitial Cystitis (CHRONIC). https://clinicaltrials.gov/show/NCT04349930 2020.

Nct. The Effect of Dronabinol on Ocular Hemodynamics in Patients With Primary Open Angle Glaucoma. https://clinicaltrials.gov/show/NCT04596826 2020.

Nct. The Effect of Marijuana and Prescription Medications in Mood, Performance and Sleep. https://clinicaltrials.gov/show/NCT00893269 2009.

Nct. The Effects of Cannabidiol on the Driving Ability of Healthy Adults. https://clinicaltrials.gov/show/NCT04590495 2020.

Nct. The Effects of Cannabinoid on Patients With Non-GERD Related Non Cardiac Chest Pain. https://clinicaltrials.gov/show/NCT01598207 2012.

Nct. The Efficacy and Safety of 3% Cannabidiol (CBD) Cream in Patients With Epidermolysis Bullosa: a Phase II/III Trial. https://clinicaltrials.gov/show/NCT04613102 2020.

Nct. The Pharmacokinetic and Pharmacodynamic Effects of Oral Cannabidiol (CBD) Under Acute and Chronic Exposure Conditions. https://clinicaltrials.gov/show/NCT04283019 2020.

Nct. The Use of Cannabinoid Patch for Knee Osteoarthritis. https://clinicaltrials.gov/show/NCT04412837 2020.

Nct. Topical CBD in Joint Arthritis. https://clinicaltrials.gov/show/NCT04611347 2020.

Nct. Treating Nightmares in Posttraumatic Stress Disorder With Dronabinol. https://clinicaltrials.gov/show/NCT04448808 2020.

Nct. Trial to Investigate the Safety and Efficacy of GWP42003-P Versus Placebo as Adjunctive Therapy in Participants With Schizophrenia Experiencing Inadequate Response to Ongoing Antipsychotic Treatment. https://clinicaltrials.gov/show/NCT04421456 2020.

Nct. Use of CBD Oil in the Treatment of Posttraumatic Stress Disorder. https://clinicaltrials.gov/show/NCT04197102 2019.

Nct. Varenicline for Co-occurring Cannabis and Tobacco Use. https://clinicaltrials.gov/show/NCT04595318 2020.

Nugent, Shannon M., Meghani, Salimah H., Rogal, Shari S., Merlin, Jessica S. Medical cannabis use among individuals with cancer: An unresolved and timely issue. Cancer 2020. 126 (9) 1832-1836.

Nurmikko, T. J., Serpell, M. G., Hoggart, B., Toomey, P. J., and Morlion, B. J. A multi-centre, double-blind, randomized, controlled trial of oro-mucosal cannabis based medicine in the treatment of neuropathic pain characterized by allodynia. Neurology 2005. 64 (Suppl 1) A374, Abstract.

Nuzzo, P. A., Lofwall, M. R., Elayi, S. C., Gill, K., Babalonis, S., and Walsh, S. L. Physiologic effects of dronabinol during opioid withdrawal in humans. Drug and alcohol dependence. 2015. 156, e165‐e166.

O’Neill, A., Annibale, L., Blest-Hopley, G., Wilson, R., Bhattacharyya, S. CBD MODULATION OF HIPPOCAMPAL GLUTAMATE IN PSYCHOSIS.Schizophrenia International Research Society (SIRS) 2020 Congress. Schizophrenia bulletin 2020. 46, S11.

O'Donnell, Brian, Meissner, Hannah, Gupta, Vikas. Dronabinol. 2020.

O'Neill, A., Wilson, R., Appiah-Kusi, E., Bossong, M., McGuire, P., and Bhattacharyya, S. Effects of cannabidiol on mediotemporal and dorsostriatal activity during encoding and recall, in the at-risk mental state for psychosis. Schizophrenia Bulletin 2017. 43, S187‐.

Patel, A., Devinsky, O., Cross, J. H., Villanueva, V., Wirrell, E., VanLandingham, K., Roberts, C., Checketts, D., and Zuberi, S. Cannabidiol (CBD) significantly reduces drop seizure frequency in Lennox-Gastaut syndrome (LGS): results of a dose-ranging, multi-center, randomized, double-blind, placebo-controlled trial (GWPCARE3). Neurology 2017. 89 (8) e100‐.

Pearlson, G., Anderson, B., Khadka, S., Dager, A., and Stevens, M. Effects of inhaled, vaporized cannabis on functional MRI signal and behavior in a simulated driving program. Neuropsychopharmacology.Conference: 55th annual meeting of the american college of neuropsychopharmacology, ACNP 2016.United states.Conference start: 20161204.Conference end: 20161208 2016. 41, S201.

Peball, Marina, Werkmann, Mario, Ellmerer, Philipp, Stolz, Raphaela, Valent, Dora, Knaus, Hans Gunther, Ulmer, Hanno, Djamshidian, Atbin, Poewe, Werner, and Seppi, Klaus. Nabilone for non-motor symptoms of Parkinson's disease: a randomized placebo-controlled, double-blind, parallel-group, enriched enrolment randomized withdrawal study (The NMS-Nab Study). Journal of neural transmission (Vienna, Austria : 1996) 2019.

Perry, D., Ton, J., and Allan, G. M. Evidence for THC versus CBD in cannabinoids. Canadian Family Physician 2018. 64 (7) 519.

Perry, M. S. Don’t Fear the Reefer—Evidence Mounts for Plant-Based Cannabidiol as Treatment for Epilepsy. Epilepsy currents 2019. 19 (2) 93‐95.

Perry, M. Scott. Don't Fear the Reefer-Evidence Mounts for Plant-Based Cannabidiol as Treatment for Epilepsy. Epilepsy currents 2019. 19 (2) 93-95.

Pierre, Joseph M. Cannabidiol (CBD) for schizophrenia: Promise or pipe dream?. Current Psychiatry 2019. 18 (5) 13-20.

Pinsger, M. Benefit of an Add-On-Treatment with a synthetic cannabinomimeticum on patients with chronic back pain-a randomized controlled trial. European spine journal 2012. 21 (11) 2366‐.

Pittler, M. H. No effect of cannabis on induced inflammatory pain. Focus on Alternative and Complementary Therapies 2009. 14 (1) 19-20.

Prasad, B., Radulovacki, M. G., and Carley, D. W. Randomized placebo controlled trial of dronabinol in obstructive sleep apnea. American journal of respiratory and critical care medicine 2011. 183 (1 MeetingAbstracts).

Rabinak, C. A., Mori, S., Lyons, M., Angstadt, M., and Phan, K. L. Cannabinoid effects on prefrontal activation during regulation of negative affect. Biological psychiatry. 2014. 75 (9 SUPPL. 1) 227S.

Rabinak, C., Peters, C., Elrahal, F., Milad, M., Rauch, S., Phan, K. L., and Greenwald, M. Cannabinoid facilitation of fear extinction in posttraumatic stress disorder. Biological Psychiatry 2018. 83 (9) S21‐.

Rabinak, C., Peters, C., Silverstein, B., Marusak, H., Gorka, S., and Phan, K. L. Effects of acute A9-tetrahydrocannabinol on resting-state functional connectivity in fear-related neural circuitry. Biological Psychiatry 2017. 81 (10) S327‐.

Radhakrishnan, R., Cahill, J., Cortes-Briones, J., Shosnik, P., Sewell, R. A., and Bois, F. Effects of low-dose THC and pharmacologically-induced GABA deficit on cognition and information processing. Biological psychiatry. 2015. 77 (9 SUPPL. 1) 274S.

Raymundi, Ana Maria, da Silva, Thiago R., Sohn, Jeferson M. B., Bertoglio, Leandro J., Stern, Cristina A. Effects of 9-tetrahydrocannabinol on aversive memories and anxiety: a review from human studies. BMC psychiatry 2020. 20 (1) 420.

Rekand, Tiina. THC:CBD spray and MS spasticity symptoms: data from latest studies. European Neurology 2014. 71 Suppl 1, 4-9.

Riva, N., Mora, G., Soraru, G., Lunetta, C., Clerici, M., and Falzone, Y. The CANALS study: a randomized, double-blind, placebo-controlled, multicentre study to assess the safety and efficacy on spasticity symptoms of a Cannabis Sativa extract in motor neuron disease patients. European journal of neurology 2016. 23 (Suppl S2) 46, Abstract.

Riva, N., Mora, G., Soraru, G., Lunetta, C., Falzone, Y., Marinou, K., Maestri, E., Fazio, R., Comola, M., and Comi, G. The canals study: a randomized, double-blind, placebo-controlled, multicentre study to assess the safety and efficacyon spasticity symptoms of a cannabis sativa extract in motor neuron disease patients. Amyotrophic lateral sclerosis and frontotemporal degeneration 2016. 17, 44‐.

Rohleder, C., Pahlisch, F., Schaefer, C., Hellmich, M., Enning, F., Van Der List, T. F., Mueller, J. K., Bumb, J. M., Koethe, D., and Leweke, F. M. The endocannabinoid system as a pharmacological target for antipsychotic treatment and more?. Early Intervention in Psychiatry 2012. 6, 7.

Rubin, Rita. Cannabidiol Products Are Everywhere, but Should People Be Using Them?. JAMA - Journal of the American Medical Association 2019. 322 (22) 2156-2158.

Ruthirakuhan, M., Herrmann, N., Abraham, E. H., Sherman, C., Verhoeff, N. P. L. G., Kiss, A., Black, S. E., Andreazza, A. C., and Lanctot, K. L. A randomized, placebo-controlled, cross-over trial investigating nabilone as a treatment for agitation in patients with moderate-to-severe Alzheimer's disease: blinded, interim safety results. Alzheimer's & dementia 2017. 13 (7) 1254‐.

Ruthirakuhan, M., Herrmann, N., Gallagher, D., Verhoeff, N. P. L. G., Black, S. E., and Lanctot, K. L. CEREBROCHOLESTEROL, A MARKER OF AGITATION SEVERITY IN PATIENTS WITH MODERATE-TO-SEVERE ALZHEIMER'S DISEASE. Alzheimer's & dementia 2018. 14 (7) 1552‐P1553.

Sahlem, G., Baker, N., Malcolm, R. J., and McRae-Clark, A. High impulsivity correlates with cannabis cue-induced craving in a non-treatment seeking cohort of heavy cannabis users. Drug and Alcohol Dependence 2017. 171, e180‐.

Salloum, I. M., Cornelius, J. R., Douaihy, A., Caceda, R., Miao, F., and Kirisci, L. Differential impact of cannabis abuse on depression remission in comorbid depression and alcoholism. Alcoholism, clinical and experimental research 2012. 36, 299A.

Salloum, I. M., Cornelius, J. R., Douaihy, A., Caceda, R., Miao, F., Levent, K., and Michael, T. E. Longstanding cannabis abuse is associated with decreased likelihood of remission from major depression. Neuropsychopharmacology. 2011. 36, S300‐S301.

Sathyapalan, T., Dakroury, Y., Ahmed, L., Elshewehy, A. M., Kilpatrick, E. S., Coady, A. M., and Atkin, S. L. Endocannabinoid receptor blockade increases hepatocyte growth factor and reduces insulin levels in obese women with polycystic ovary syndrome. Clinical endocrinology 2016. 85 (4) 671‐673.

Schoedel, K., Etges, T., Levy-Cooperman, N., Mills, C., Sellers, E., Setnik, B., Szeto, I., and Sommerville, K. A randomized, double-blind, placebo-controlled, crossover study to evaluate the abuse potential of purified cannabidiol (CBD) in subjects with a history of recreational polydrug use. Neurology 2018. 90 (15).

Short, S. C. and Little, C. A 2-part safety and exploratory efficacy randomised double-blind, placebo-controlled study of a 1: 1 ratio of cannabidiol and delta-9-tetrahydrocannabinol (CBD: tHC) plus doseintense temozolomide in patients with recurrent glioblastoma multiforme (GBM). Neuro-oncology 2017. 19, vi13‐.

Solowij, N., Broyd, S., Van Hell, H., Greenwood, L. M., Michie, P., Todd, J., Jones, A., Zuardi, A., Leweke, F. M., and Murray, R. Opposite effects of THC and CBD on auditory mismatch negativity: a randomised controlled trial of acute cannabinoid administration. European Neuropsychopharmacology 2014. 24, S215‐.

Spiera, R. F., Hummers, L. K., Chung, L., Frech, T. M., Domsic, R. T., Hsu, V., Furst, D. E., Gordon, J. K., Mayes, M. D., and Simms, R. W. A phase 2 study of safety and efficacy of anabasum (JBT-101), a cannabinoid receptor type 2 agonist, in diffuse cutaneous systemic sclerosis. Arthritis & rheumatology 2017. 69.

Stevens, M., Pancholi, K., Hawkins, K., Potenza, M., Pearlson, G., and Anderson, B. The effect of cannabis use on cognitive set-shifting functional connectivity. Neuropsychopharmacology 2016. 41, S283‐S284.

stgs, R. B. R. Effect of cannabidiol in the treatment of crack dependence. http://www.who.int/trialsearch/Trial2.aspx?TrialID=RBR-4stgs8 2019.

Stone, J. M., Morrison, P. D., Nottage, J., Bhattacharyya, S., Feilding, A., and McGuire, P. K. Delta-9-tetrahydrocannabinol disruption of time perception and of self-timed actions. Pharmacopsychiatry 2010. 43 (6) 236-237.

Subbaraman, M. S., Patterson, D., Metrik, J., and Swift, R. Cannabis use during treatment for alcohol use disorders is associated with more frequent alcohol consumption. Alcoholism: clinical and experimental research.Conference: 39th annual scientific meeting of the research society on alcoholism.New orleans, LA united states.Conference start: 20160625.Conference end: 20160629.Conference publication: (var.pagings) 2016. 40, 102A.

Tctr. comparison efficacy of ACT and DBT in Cannabis use disorder. http://www.who.int/trialsearch/Trial2.aspx?TrialID=TCTR20200319007 2020.

Tctr. Effectiveness of Cannabis-based medicines for treatment of neuropathic pain in patients with traumatic brachial plexus injury: a triple blind, cross over, randomized control trial. http://www.who.int/trialsearch/Trial2.aspx?TrialID=TCTR20191106004 2019.

Theunissen, E., Kauert, G., Toennes, S., Moeller, M., Sambeth, A., Blanchard, M., and Ramaekers, J. Event related potentials in occasional and heavy cannabis users while under the influence of cannabis. International Journal of Neuropsychopharmacology 2012. 15, 227.

Thiele, E. A., Mazurkiewicz-Beldzinska, M., Benbadis, S., Marsh, E. D., Joshi, C., French, J. A., Roberts, C., Taylor, A., and Sommerville, K. Treatment with cannabidiol (CBD) significantly reduces drop seizure frequency in Lennox Gastaut Syndrome (LGS): results of a multi - Center, randomized, double-blind, Placebo-controlled trial (GWPCARE4). Neurotherapeutics 2017. 14 (3) 824‐825.

Thompson, J. P., Lam, E., Thomas, A. M., Harry, F., Hutchings, A. D., Marshall, R. W., and Routledge, P. A. The pharmacokinetics and CNS effects of 5 and 10 mg of oral DELTA-9-tetrahydrocannabinol in man. British Journal of Clinical Pharmacology 2000. 50 (4) 385-386.

Turcotte, D. A., Gomori, A. J., Esfahani, F. E., and Namaka, M. P. Randomized, double-blinded, placebo-controlled study evaluating the efficacy and safety of nabilone adjunctive to gabapentin in managing multiple sclerosis-induced neuropathic pain. European journal of pain supplements. 2011. 5 (1) 240‐241.

Turcotte, D., Chateau, D., Doupe, M., Esfahani, F., Ethans, K., Gomori, A., Prout, M., and Namaka, M. A randomised, double-blinded, placebo-controlled study evaluating the efficacy and safety of nabilone as an adjunctive to gabapentin in managing multiple sclerosisinduced neuropathic pain. Multiple sclerosis. 2011. 17 (10 SUPPL. 1) S475‐S476.

Turcotte, D., Doupe, M., Torabi, M., Ethans, K., Esfahani, F., Gomori, A., and Namaka, M. A randomized, double-blinded, placebo-controlled study evaluating efficacy and tolerability of nabilone as an adjunctive to gabapentin in the management of multiple sclerosis-induced neuropathic pain. Multiple sclerosis. 2013. 19 (11 SUPPL. 1) 112.

Twelves, C., Short, S., and Wright, S. A two-part safety and exploratory efficacy randomized double-blind, placebo-controlled study of a 1: 1 ratio of the cannabinoids cannabidiol and delta-9-tetrahydrocannabinol (CBD: tHC) plus dose-intense temozolomide in patients with recurrent glioblastoma multiforme (GBM). Journal of Clinical Oncology 2017. 35 (15).

Valle, M., Martial, L. C., Garrido, M., Rodriguez-Fornells, A., Puntes, M., Antonijoan, R. M., Claramunt, J., Martinez, D., Gonzalez, M., and Martinez, M. Cannabidiol attenuates the increased susceptibility to false memories produced by tetrahydrocannabinol. Drug and alcohol dependence. 2015. 156, e228.

van Amerongen, G., Beumer, T., Killestein, J., and Groeneveld, G. J. Individualized dosing of a novel oral DELTA9-THC formulation improves subjective spasticity and pain in patients with progressive multiple sclerosis. Multiple sclerosis (Houndmills, Basingstoke, England) 2014. 20 (1 SUPPL. 1) 478‐479.

van den Elsen, G. A. H., Ahmed, A. I. A., Jan Verkes, R., Kramers, K., Feuth, T., Olde Rikkert, M. G. M., and van der Marck, M. A. Efficacy and safety of delta-9-tetrahydrocannabinol in behavioral disturbances in dementia: a randomized controlled trial. Alzheimer's & dementia 2015. 11 (7) 469‐P470.

Van Hell, H. H., Bossong, M. G., Jager, G., Saliasi, E., Kahn, R. S., and Ramsey, N. F. The acute effects of delta-9-tetrahydrocannabinol (THC) on brain activity during working memory. European neuropsychopharmacology. 2009. 19 (var.pagings) S308.

Van Hell, H. H., Broyd, S., Greenwood, L., Croft, R., and Solowij, N. Delta9-tetrahydrocannabinol (THC) and cannabidiol (CBD) alone and in combination affect brain activity during rest. European neuropsychopharmacology. 2015. 25, S621‐S622.

Wallace, M., Atkinson, J., Gouaux, B., Marcotte, T., and Umlauf, A. Effect of smoked cannabis on painful diabetic peripheral neuropathy. Journal of pain. 2013. 14 (4 SUPPL. 1) S62.

Wallace, M. Correlation of tetrahydrocannabinol plasma levels with pain reduction in diabetic peripheral neuropathy. Pain Medicine (United States) 2017. 18 (3) e4‐.

Walter, C., Oertel, B. G., Felden, L., Noth, U., Deichmann, R., and Lotsch, J. The effects of delta-9-tetrahydrocannabinol on nasal chemosensitivity: a pharmacological fMRI study in healthy volunteers. Naunyn-Schmiedeberg's Archives of Pharmacology 2011. 383, 75.

Walther, Sebastian, Schupbach, Brigitte, Seifritz, Erich, Homan, Philipp, and Strik, Werner. Randomized, controlled crossover trial of dronabinol, 2.5 mg, for agitation in 2 patients with dementia. Journal of Clinical Psychopharmacology 2011. 31 (2) 256-258.

Werth, V. P., Hejazi, E., Pena, S. M., Haber, J. S., Okawa, J., Feng, R., Gabre, K., Concha, J., Constantine, S., and White, B. Comparison of patients with dermatomyositis in a specialty clinic versus clinical trial with anabasum (JBT-101), a cannabinoid receptor type 2 agonist. Arthritis & rheumatology 2017. 69.

Wirrell, E., Devinsky, O., Patel, A., Zuberi, S., Cross, J., Villanueva, V., Roberts, C., Checketts, D., and VanLandingham, K. Cannabidiol (CBD) Significantly Reduces Drop and Total Seizure Frequency in Lennox Gastaut Syndrome (LGS): results of a Dose Ranging, Multicenter, Randomized, Double Blind, Placebo Controlled Trial (GWPCARE3). Annals of Neurology 2017. 82 (S21) S279‐S280, Abstract.

Wright, S., Vachova, M. M., and Novakova, I. The effect of long-term treatment with a prescription cannabisbased THC: CBD oromucosal spray on cognitive function and mood: a 12 month double blind placebo-controlled study in people with spasticity due to multiple sclerosis. Multiple sclerosis. 2013. 19 (11 SUPPL. 1) 572‐573.

Zajicek, John. Cannabinoids on trial for multiple sclerosis. The Lancet.Neurology 2002. 1 (3) 147.

Zalai, D., Chung, S. A., Hussain, N., and Shapiro, C. M. Does cannabinoid really improve sleep? Testing the sleep effects of nabilone in chronic pain patients: a placebo-controlled, randomized, pilot study. Psychotherapy and psychosomatics. 2015. 84, 81.

Zuberi, S., Devinsky, O., Patel, A., Cross, J. H., Villanueva, V., Wirrell, E. C., Roberts, C., Checketts, D., and Van Landingham, K. Cannabidiol (CBD) significantly reduces drop and total seizure frequency in lennox-gastaut syndrome (LGS): results of a dose-ranging, multi-centre, randomised, double-blind, placebo-controlled trial (GWPCARE3). Epilepsia 2017. 58, S13‐S14.

Zuberi, S., Patel, A. D., Wilfong, A., Joshi, C., Gil-Nagel, A., Roberts, C., Vanlandingham, K. Cannabidiol (CBD) significantly reduces drop-seizure frequency in Lennox-Gastaut syndrome (LGS): pooled efficacy and safety results from two randomized controlled trials. Developmental medicine and child neurology 2017. 59 (Suppl 4) 18‐19.

Participants not randomized and not a potentially relevant NRS

[Author not listed]. Pharmacokinetic investigation of synthetic cannabidiol oral formulations in healthy volunteers. European journal of pharmaceutics and biopharmaceutics 2020. 154, 108‐115.

[Author not listed]. Effects of exercise on experimentally manipulated craving for cannabis: a preliminary study. Experimental and clinical psychopharmacology 2018. 26 (5) 456‐466.

Androvicova, R., Horacek, J., Tintera, J., Hlinka, J., Rydlo, J., Jezova, D., Balikova, M., Hlozek, T., Miksatkova, P., Kuchar, M., Roman, M., Tomicek, P., Tyls, F., Viktorinova, M., and Palenicek, T. Individual prolactin reactivity modulates response of nucleus accumbens to erotic stimuli during acute cannabis intoxication: an fMRI pilot study. Psychopharmacology 2017. 234 (13) 1933-1943.

Bedi, Gillinder, Foltin, Richard W., Gunderson, Erik W., Rabkin, Judith, Hart, Carl L., Comer, Sandra D., Vosburg, Suzanne K., and Haney, Margaret. Efficacy and tolerability of high-dose dronabinol maintenance in HIV-positive marijuana smokers: a controlled laboratory study. Psychopharmacology 2010. 212 (4) 675-686.

Bhattacharyya, Sagnik, Atakan, Z., Martin-Santos, R., Crippa, J. A., Kambeitz, J., Malhi, S., Giampietro, V., Williams, S., Brammer, M., Rubia, K., Collier, D. A., and McGuire, P. K. Impairment of inhibitory control processing related to acute psychotomimetic effects of cannabis. European neuropsychopharmacology : the journal of the European College of Neuropsychopharmacology 2015. 25 (1) 26-37.

Bhattacharyya, Sagnik, Egerton, Alice, Kim, Euitae, Rosso, Lula, Riano Barros, Daniela, Hammers, Alexander, Brammer, Michael, Turkheimer, Federico E., Howes, Oliver D., and McGuire, Philip. Acute induction of anxiety in humans by delta-9-tetrahydrocannabinol related to amygdalar cannabinoid-1 (CB1) receptors. Scientific reports 2017. 7 (1) 15025.

Ghahari, Shahrbanoo, Zandnia, Fatemeh, Mazloumirad, Mohammad, Ghayoomi, Raoofeh, Gheitarani, Bahram. The effectiveness of chair work intervention on anxiety and depression in divorced women using Cannabis. Asian journal of psychiatry 2019. 44, 161-162.

Good, Phillip D., Greer, Ristan M., Huggett, Georgina E., Hardy, Janet R. An Open-Label Pilot Study Testing the Feasibility of Assessing Total Symptom Burden in Trials of Cannabinoid Medications in Palliative Care. Journal of palliative medicine 2020. 23 (5) 650-655.

Goodman, Samantha, Leos-Toro, Cesar, Hammond, David. The impact of plain packaging and health warnings on consumer appeal of cannabis products. Drug and alcohol dependence 2019. 205, 107633.

Gupta, Swapnil, De Aquino, Joao P., D'Souza, Deepak C., and Ranganathan, Mohini. Effects of haloperidol on the delta-9-tetrahydrocannabinol response in humans: a responder analysis. Psychopharmacology 2019.

Hall, Sharon M., Humfleet, Gary L., Gorecki, Julie A., Munoz, Ricardo F., Reus, Victor I., and Prochaska, Judith J. Older versus younger treatment-seeking smokers: differences in smoking behavior, drug and alcohol use, and psychosocial and physical functioning. Nicotine & tobacco research : official journal of the Society for Research on Nicotine and Tobacco 2008. 10 (3) 463-470.

Hindocha, C., Freeman, T. P., Grabski, M., Crudgington, H., Davies, A. C., Stroud, J. B., Das, R. K., Lawn, W., Morgan, C. J. A., and Curran, H. V. The effects of cannabidiol on impulsivity and memory during abstinence in cigarette dependent smokers. Scientific reports 2018. 8 (1) 7568.

Lammert, S., Harrison, K., Tosun, N., Allen, S. Menstrual Cycle in Women Who Co-use Marijuana and Tobacco. Journal of addiction medicine 2018. 12 (3) 207‐211.

Lee, D. C., Walker, D. D., Hughes, J. R., Brunette, M. F., Scherer, E., Stanger, C., Etter, J. F., Auty, S., Budney, A. J. Sequential and simultaneous treatment approaches to cannabis use disorder and tobacco use. Journal of substance abuse treatment 2019. 98, 39‐46.

Lus, Giacomo, Cantello, Roberto, Danni, Maura Chiara, Rini, Agusto, Sarchielli, Paola, Tassinari, Tiziana, and Signoriello, Elisabetta. Palatability and oral cavity tolerability of THC:CBD oromucosal spray and possible improvement measures in multiple sclerosis patients with resistant spasticity: a pilot study. Neurodegenerative disease management 2018. 8 (2) 105-113.

Morales-Munoz, I., Jurado-Barba, R., Caballero, M., Rodriguez-Jimenez, R., Jimenez-Arriero, M. A., and Fernandez-Guinea, S. Cannabis abuse effects on prepulse inhibition in patients with first episode psychosis in schizophrenia. Journal of neuropsychiatry and clinical neurosciences 2015. 27 (1) 48‐53.

Morrison, Gilmour, Crockett, Julie, Blakey, Graham, and Sommerville, Kenneth. A Phase 1, Open-Label, Pharmacokinetic Trial to Investigate Possible Drug-Drug Interactions Between Clobazam, Stiripentol, or Valproate and Cannabidiol in Healthy Subjects. Clinical pharmacology in drug development 2019.

Onesti, E., Frasca, V., Ceccanti, M., Tartaglia, G., Gori, M. C., Cambieri, C., Libonati, L., Palma, E., Inghilleri, M. Short-Term Ultramicronized Palmitoylethanolamide Therapy in Patients with Myasthenia Gravis: a Pilot Study to Possible Future Implications of Treatment. CNS & neurological disorders drug targets 2019. 18 (3) 232‐238.

Orri, M., Seguin, J. R., Castellanos-Ryan, N., Tremblay, R. E., Cote, S. M., Turecki, G., Geoffroy, M. C. A genetically informed study on the association of cannabis, alcohol, and tobacco smoking with suicide attempt. Molecular psychiatry 2020.

Ottaviani, Giulia, Rupel, Katia, Gobbo, Margherita, Poropat, Augusto, Zoi, Valentina, Di Lenarda, Roberto, Biasotto, Matteo, Faraon, Michela. Efficacy of ultramicronized palmitoylethanolamide in burning mouth syndrome-affected patients: a preliminary randomized double-blind controlled trial. Clinical oral investigations 2019. 23 (6) 2743-2750.

Rogers, Andrew H., Shepherd, Justin M., Buckner, Julia D., Garey, Lorra, Manning, Kara, Orr, Michael F., Schmidt, Norman B., Zvolensky, Michael J. Current cannabis use and smoking cessation among treatment seeking combustible smokers. Drug and alcohol dependence 2020. 209, 107928.

Rossi, Gemma Caterina Maria, Lumini, Chiara, Bettio, Federica, Picasso, Erica, Ruberto, Giulio, Briola, Aba, Mirabile, Alessandra, Paviglianiti, Alessia, Bianchi, Paolo Emilio, Scudeller, Luigia, Pasinetti, Gian Maria. Effect of palmitoylethanolamide on inner retinal function in glaucoma: a randomized, single blind, crossover, clinical trial by pattern-electroretinogram. Scientific reports 2020. 10 (1) 10468.

Rubenstein, Dana, Aston, Elizabeth R., Nollen, Nicole L., Mayo, Matthew S., Brown, Alexandra R., Ahluwalia, Jasjit S. Factors Associated With Cannabis Use Among African American Nondaily Smokers. Journal of addiction medicine 2020. 14 (5) e170-e174.

Schoedel, Kerri A., Addy, Carol, Chakraborty, Bijan, Rosko, Kim, Dunbar, Stephanie, Maes, Andrea, Chen, Nancy, Stoch, Selwyn Aubrey, Wagner, John, Chodakewitz, Jeff, and Sellers, Edward M. Human abuse potential and cognitive effects of taranabant, a cannabinoid 1 receptor inverse agonist: a randomized, double-blind, placebo- and active-controlled, crossover study in recreational polydrug users. Journal of Clinical Psychopharmacology 2012. 32 (4) 492-502.

Staquet, M., Gantt, C., Machin, D. Effect of a nitrogen analog of tetrahydrocannabinol on cancer pain. Clin Pharmacol Ther 1978. 23 (4) 397-401.

Tarique, Abdullah A., Evron, Tama, Zhang, George, Tepper, Mark A., Morshed, Mohammed M., Andersen, Isabella S. G., Begum, Nelufa, Sly, Peter D., Fantino, Emmanuelle. Anti-inflammatory effects of lenabasum, a cannabinoid receptor type 2 agonist, on macrophages from cystic fibrosis. Journal of cystic fibrosis : official journal of the European Cystic Fibrosis Society 2020. 19 (5) 823-829.

Theunissen, E. L., Heckman, P., de Sousa Fernandes Perna, E. B., Kuypers, K. P. C., Sambeth, A., Blokland, A., Prickaerts, J., Toennes, S. W., and Ramaekers, J. G. Rivastigmine but not vardenafil reverses cannabis-induced impairment of verbal memory in healthy humans. Psychopharmacology 2015. 232 (2) 343-353.

Verweij, K. J. H., Treur, J. L., Vink, J. M. Investigating causal associations between use of nicotine, alcohol, caffeine and cannabis: a two-sample bidirectional Mendelian randomization study. Addiction (Abingdon, England) 2018. 113 (7) 1333‐1338.

Woolard, Robert, Baird, Janette, Mello, Michael J., Lee, Christina, Harington, Magda, Nirenberg, Ted, Becker, Bruce, Stein, Lynn, and Longabaugh, Richard. Injuries, negative consequences, and risk behaviors among both injured and uninjured emergency department patients who report using alcohol and marijuana. Journal of emergencies, trauma, and shock 2009. 2 (1) 23-28.

Zhang, Xuan, de Leon, Jose, Crespo-Facorro, Benedicto, Diaz, Francisco J. Measuring individual benefits of psychiatric treatment using longitudinal binary outcomes: Application to antipsychotic benefits in non-cannabis and cannabis users. Journal of biopharmaceutical statistics 2020. 30 (5) 916-940.

Potentially relevant NRS added to the appropriate database for screening

Prashad, Shikha, Dedrick, Elizabeth S., To, Wing Ting, Vanneste, Sven, Filbey, Francesca M. Testing the role of the posterior cingulate cortex in processing salient stimuli in cannabis users: an rTMS study. European Journal of Neuroscience 2019. 50 (3) 2357-2369.

Taylor, L., Crockett, J., Tayo, B., Morrison, G. A Phase 1, Open-Label, Parallel-Group, Single-Dose Trial of the Pharmacokinetics and Safety of Cannabidiol (CBD) in Subjects With Mild to Severe Hepatic Impairment. Journal of clinical pharmacology 2019. 59 (8) 1110‐1119.

Did not meet age criteria

Adam, K. C. S., Doss, M. K., Pabon, E., Vogel, E. K., de Wit, H. Î”9-Tetrahydrocannabinol (THC) impairs visual working memory performance: a randomized crossover trial. Neuropsychopharmacology 2020.

Adam, Kirsten C. S., Doss, Manoj K., Pabon, Elisa, Vogel, Edward K., de Wit, Harriet. DELTA9-Tetrahydrocannabinol (THC) impairs visual working memory performance: a randomized crossover trial. Neuropsychopharmacology : official publication of the American College of Neuropsychopharmacology 2020. 45 (11) 1807-1816.

Andries, Alin, Frystyk, Jan, Flyvbjerg, Allan, and Stoving, Rene Klinkby. Changes in IGF-I, urinary free cortisol and adipokines during dronabinol therapy in anorexia nervosa: Results from a randomised, controlled trial. Growth hormone & IGF research : official journal of the Growth Hormone Research Society and the International IGF Research Society 2015. 25 (5) 247-252.

Arkell, Thomas R., Lintzeris, Nicholas, Kevin, Richard C., Ramaekers, Johannes G., Vandrey, Ryan, Irwin, Christopher, Haber, Paul S., and McGregor, Iain S. Cannabidiol (CBD) content in vaporized cannabis does not prevent tetrahydrocannabinol (THC)-induced impairment of driving and cognition. Psychopharmacology 2019.

Arndt, David L. and de Wit, Harriet. Cannabidiol Does Not Dampen Responses to Emotional Stimuli in Healthy Adults. Cannabis and Cannabinoid Research 2017. 2 (1) 105-113.

Babalonis, Shanna, Haney, Margaret, Malcolm, Robert J., Lofwall, Michelle R., Votaw, Victoria R., Sparenborg, Steven, and Walsh, Sharon L. Oral cannabidiol does not produce a signal for abuse liability in frequent marijuana smokers. Drug and Alcohol Dependence 2017. 172, 9-13.

Babalonis, Shanna, Lofwall, Michelle R., Sloan, Paul A., Nuzzo, Paul A., Fanucchi, Laura C., Walsh, Sharon L. Cannabinoid modulation of opioid analgesia and subjective drug effects in healthy humans. Psychopharmacology 2019. 236 (11) 3341-3352.

Ballard, Michael E. and de Wit, Harriet. Combined effects of acute, very-low-dose ethanol and delta(9)-tetrahydrocannabinol in healthy human volunteers. Pharmacology, biochemistry, and behavior 2011. 97 (4) 627-631.

Bedi, Gillinder, Cooper, Ziva D., and Haney, Margaret. Subjective, cognitive and cardiovascular dose-effect profile of nabilone and dronabinol in marijuana smokers. Addiction Biology 2013. 18 (5) 872-881.

Bergamaschi, Mateus M., Queiroz, Regina Helena Costa, Chagas, Marcos Hortes Nisihara, de Oliveira, Danielle Chaves Gomes, De Martinis, Bruno Spinosa, Kapczinski, Flavio, Quevedo, Joao, Roesler, Rafael, Schroder, Nadja, Nardi, Antonio E., Martin-Santos, Rocio, Hallak, Jaime Eduardo Cecilio, Zuardi, Antonio Waldo, and Crippa, Jose Alexandre. Cannabidiol reduces the anxiety induced by simulated public speaking in treatment-naive social phobia patients. Neuropsychopharmacology : official publication of the American College of Neuropsychopharmacology 2011. 36 (6) 1219-1226.

Bocker, K. B. E., Gerritsen, J., Hunault, C. C., Kruidenier, M., Mensinga, Tj T., and Kenemans, J. L. Cannabis with high delta9-THC contents affects perception and visual selective attention acutely: an event-related potential study. Pharmacology, biochemistry, and behavior 2010. 96 (1) 67-74.

Bossong, Matthijs G., Jansma, J. Martijn, van Hell, Hendrika H., Jager, Gerry, Oudman, Erik, Saliasi, Emi, Kahn, Rene S., and Ramsey, Nick F. Effects of delta9-tetrahydrocannabinol on human working memory function. Biological Psychiatry 2012. 71 (8) 693-699.

Cooper, Ziva D., Bedi, Gillinder, Ramesh, Divya, Balter, Rebecca, Comer, Sandra D., and Haney, Margaret. Impact of co-administration of oxycodone and smoked cannabis on analgesia and abuse liability. Neuropsychopharmacology : official publication of the American College of Neuropsychopharmacology 2018. 43 (10) 2046-2055.

Cooper, Ziva D., Comer, Sandra D., and Haney, Margaret. Comparison of the analgesic effects of dronabinol and smoked marijuana in daily marijuana smokers. Neuropsychopharmacology : official publication of the American College of Neuropsychopharmacology 2013. 38 (10) 1984-1992.

Cortes-Briones, Jose, Skosnik, Patrick D., Mathalon, Daniel, Cahill, John, Pittman, Brian, Williams, Ashley, Sewell, R. Andrew, Ranganathan, Mohini, Roach, Brian, Ford, Judith, and D'Souza, Deepak Cyril. DELTA9-THC Disrupts Gamma (gamma)-Band Neural Oscillations in Humans. Neuropsychopharmacology : official publication of the American College of Neuropsychopharmacology 2015. 40 (9) 2124-2134.

Davies, Cathy, Wilson, Robin, Appiah-Kusi, Elizabeth, Blest-Hopley, Grace, Brammer, Michael, Perez, Jesus, Murray, Robin M., Allen, Paul, Bossong, Matthijs G., McGuire, Philip, Bhattacharyya, Sagnik. A single dose of cannabidiol modulates medial temporal and striatal function during fear processing in people at clinical high risk for psychosis. Translational psychiatry 2020. 10 (1) 311.

Doss, M. K., Weafer, J., Gallo, D. A., de Wit, H. Δ9-Tetrahydrocannabinol at Retrieval Drives False Recollection of Neutral and Emotional Memories. Biological psychiatry 2018. 84 (10) 743‐750.

Englund, Amir, Atakan, Zerrin, Kralj, Aleksandra, Tunstall, Nigel, Murray, Robin, and Morrison, Paul. The effect of five day dosing with THCV on THC-induced cognitive, psychological and physiological effects in healthy male human volunteers: A placebo-controlled, double-blind, crossover pilot trial. Journal of psychopharmacology (Oxford, England) 2016. 30 (2) 140-151.

Freeman, Daniel, Dunn, Graham, Murray, Robin M., Evans, Nicole, Lister, Rachel, Antley, Angus, Slater, Mel, Godlewska, Beata, Cornish, Robert, Williams, Jonathan, Di Simplicio, Martina, Igoumenou, Artemis, Brenneisen, Rudolf, Tunbridge, Elizabeth M., Harrison, Paul J., Harmer, Catherine J., Cowen, Philip, and Morrison, Paul D. How cannabis causes paranoia: using the intravenous administration of 9-tetrahydrocannabinol (THC) to identify key cognitive mechanisms leading to paranoia. Schizophrenia Bulletin 2015. 41 (2) 391-399.

Fusar-Poli, Paolo, Crippa, Jose A., Bhattacharyya, Sagnik, Borgwardt, Stefan J., Allen, Paul, Martin-Santos, Rocio, Seal, Marc, Surguladze, Simon A., O'Carrol, Colin, Atakan, Zerrin, Zuardi, Antonio W., and McGuire, Philip K. Distinct effects of {delta}9-tetrahydrocannabinol and cannabidiol on neural activation during emotional processing. Archives of general psychiatry 2009. 66 (1) 95-105.

Gorka, Stephanie M., Phan, K. Luan, Lyons, Maryssa, Mori, Shoko, Angstadt, Mike, and Rabinak, Christine A. Cannabinoid Modulation of Frontolimbic Activation and Connectivity During Volitional Regulation of Negative Affect. Neuropsychopharmacology : official publication of the American College of Neuropsychopharmacology 2016. 41 (7) 1888-1896.

Greenwald, M. K. and Stitzer, M. L. Antinociceptive, subjective and behavioral effects of smoked marijuana in humans. Drug and Alcohol Dependence 2000. 59 (3) 261-275.

Guy, G. W. and Robson, P. J. A phase I, double blind, three-way crossover study to assess the pharmacokinetic profile of Cannabis Based Medicine Extract (CBME) administered sublingually in variant cannabinoid ratios in normal healthy male volunteers (GWPK0215). Journal of Cannabis Therapeutics 2003. 3 (4) 121-152.

Haney, Margaret, Malcolm, Robert J., Babalonis, Shanna, Nuzzo, Paul A., Cooper, Ziva D., Bedi, Gillinder, Gray, Kevin M., McRae-Clark, Aimee, Lofwall, Michelle R., Sparenborg, Steven, and Walsh, Sharon L. Oral Cannabidiol does not Alter the Subjective, Reinforcing or Cardiovascular Effects of Smoked Cannabis. Neuropsychopharmacology : official publication of the American College of Neuropsychopharmacology 2016. 41 (8) 1974-1982.

Haney, Margaret, Rabkin, Judith, Gunderson, Erik, and Foltin, Richard W. Dronabinol and marijuana in HIV(+) marijuana smokers: acute effects on caloric intake and mood. Psychopharmacology 2005. 181 (1) 170-178.

Herrmann, Evan S., Cooper, Ziva D., Bedi, Gillinder, Ramesh, Divya, Reed, Stephanie Collins, Comer, Sandra D., Foltin, Richard W., and Haney, Margaret. Varenicline and nabilone in tobacco and cannabis co-users: effects on tobacco abstinence, withdrawal and a laboratory model of cannabis relapse. Addiction Biology 2019. 24 (4) 765-776.

Hill, Kevin P., Palastro, Matthew D., Gruber, Staci A., Fitzmaurice, Garrett M., Greenfield, Shelly F., Lukas, Scott E., and Weiss, Roger D. Nabilone pharmacotherapy for cannabis dependence: A randomized, controlled pilot study. The American journal on addictions 2017. 26 (8) 795-801.

Hindocha, Chandni, Freeman, Tom P., Grabski, Meryem, Stroud, Jack B., Crudgington, Holly, Davies, Alan C., Das, Ravi K., Lawn, William, Morgan, Celia J. A., and Curran, H. Valerie. Cannabidiol reverses attentional bias to cigarette cues in a human experimental model of tobacco withdrawal. Addiction (Abingdon, England) 2018.

Hindocha, Chandni, Freeman, Tom P., Schafer, Grainne, Gardner, Chelsea, Bloomfield, Michael A. P., Bramon, Elvira, Morgan, Celia J. A., and Curran, H. Valerie. Acute effects of cannabinoids on addiction endophenotypes are moderated by genes encoding the CB1 receptor and FAAH enzyme. Addiction Biology 2019., e12762.

Hommer, N., Kallab, M., Szegedi, S., Puchner, S., Stjepanek, K., Bauer, M., Werkmeister, R. M., Schmetterer, L., Abensperg-Traun, M., Garhöfer, G., et al. The Effect of Orally Administered Dronabinol on Optic Nerve Head Blood Flow in Healthy Subjects-A Randomized Clinical Trial. Clinical pharmacology and therapeutics 2020. 108 (1) 155‐161.

Hommer, Nikolaus, Kallab, Martin, Szegedi, Stephan, Puchner, Stefan, Stjepanek, Kristina, Bauer, Martin, Werkmeister, Rene M., Schmetterer, Leopold, Abensperg-Traun, Marihan, Garhofer, Gerhard, Schmidl, Doreen. The Effect of Orally Administered Dronabinol on Optic Nerve Head Blood Flow in Healthy Subjects-A Randomized Clinical Trial. Clinical pharmacology and therapeutics 2020. 108 (1) 155-161.

Hosseini, Adele, McLachlan, Andrew J., Lickliter, Jason D. A phase I trial of the safety, tolerability and pharmacokinetics of cannabidiol administered as single-dose oil solution and single and multiple doses of a sublingual wafer in healthy volunteers. British journal of clinical pharmacology 2021. 87 (4) 2070-2077.

Huestis, Marilyn A., Blount, Benjamin C., Milan, Daniel F., Newmeyer, Matthew N., Schroeder, Jennifer, and Smith, Michael L. Correlation of creatinine- and specific gravity-normalized free and glucuronidated urine cannabinoid concentrations following smoked, vaporized, and oral cannabis in frequent and occasional cannabis users. Drug Testing and Analysis 2019.

Huestis, Marilyn A., Sempio, Cristina, Newmeyer, Matthew N., Andersson, Maria, Barnes, Allan J., Abulseoud, Osama A., Blount, Benjamin C., Schroeder, Jennifer, Smith, Michael L. Free and Glucuronide Urine Cannabinoids after Controlled Smoked, Vaporized and Oral Cannabis Administration in Frequent and Occasional Cannabis Users. Journal of analytical toxicology 2020. 44 (7) 651-660.

Hunault, Claudine C., Bocker, Koen B. E., Stellato, R. K., Kenemans, J. Leon, de Vries, Irma, and Meulenbelt, Jan. Acute subjective effects after smoking joints containing up to 69 mg DELTA9-tetrahydrocannabinol in recreational users: a randomized, crossover clinical trial. Psychopharmacology 2014. 231 (24) 4723-4733.

Jicha, Crystal J., Lofwall, Michelle R., Nuzzo, Paul A., Babalonis, Shanna, Elayi, Samy Claude, and Walsh, Sharon L. Safety of oral dronabinol during opioid withdrawal in humans. Drug and Alcohol Dependence 2015. 157, 179-183.

Kalliomäki, J., Philipp, A., Baxendale, J., Annas, P., Karlsten, R., and Segerdahl, M. Lack of effect of central nervous system-active doses of nabilone on capsaicin-induced pain and hyperalgesia. Clinical and experimental pharmacology & physiology 2012. 39 (4) 336‐342.

Kalliomaki, Jarkko, Philipp, Andrew, Baxendale, Jane, Annas, Peter, Karlsten, Rolf, and Segerdahl, Marta. Lack of effect of central nervous system-active doses of nabilone on capsaicin-induced pain and hyperalgesia. Clinical and experimental pharmacology & physiology 2012. 39 (4) 336-342.

Kalliomaki, Jarkko, Segerdahl, Marta, Webster, Lynn, Reimfelt, Annika, Huizar, Karin, Annas, Peter, Karlsten, Rolf, and Quiding, Hans. Evaluation of the analgesic efficacy of AZD1940, a novel cannabinoid agonist, on post-operative pain after lower third molar surgical removal. Scandinavian journal of pain 2013. 4 (1) 17-22.

Karschner, E. L., Darwin, W. D., McMahon, R. P., Liu, F., Wright, S., Goodwin, R. S., and Huestis, M. A. Subjective and physiological effects after controlled Sativex and oral THC administration. Clinical pharmacology and therapeutics 2011. 89 (3) 400-407.

Karschner, Erin L., Darwin, W. David, Goodwin, Robert S., Wright, Stephen, and Huestis, Marilyn A. Plasma cannabinoid pharmacokinetics following controlled oral delta9-tetrahydrocannabinol and oromucosal cannabis extract administration. Clinical chemistry 2011. 57 (1) 66-75.

Kaufmann, R. M., Kraft, B., Frey, R., Winkler, D., Weiszenbichler, S., Backer, C., Kasper, S., and Kress, H. G. Acute psychotropic effects of oral cannabis extract with a defined content of Delta9-tetrahydrocannabinol (THC) in healthy volunteers. Pharmacopsychiatry 2010. 43 (1) 24-32.

Kayser, Reilly R., Haney, Margaret, Raskin, Marissa, Arout, Caroline, Simpson, Helen Blair. Acute effects of cannabinoids on symptoms of obsessive-compulsive disorder: A human laboratory study. Depression and anxiety 2020. 37 (8) 801-811.

Kim, H. K., Pears, K. C., Leve, L. D., Chamberlain, P., Smith, D. K. Intervention Effects on Health-Risking Sexual Behavior Among Girls in Foster Care: the Role of Placement Disruption and Tobacco and Marijuana Use. Journal of child & adolescent substance abuse 2013. 22 (5) 370‐387.

Klumpers, Linda E., Cole, David M., Khalili-Mahani, Najmeh, Soeter, Roelof P., Te Beek, Erik T., Rombouts, Serge A. R. B., and van Gerven, Joop M. A. Manipulating brain connectivity with delta9-tetrahydrocannabinol: a pharmacological resting state FMRI study. NeuroImage 2012. 63 (3) 1701-1711.

Knaub, K., Sartorius, T., Dharsono, T., Wacker, R., Wilhelm, M., Schön, C. A Novel Self-Emulsifying Drug Delivery System (SEDDS) Based on VESIsorb® Formulation Technology Improving the Oral Bioavailability of Cannabidiol in Healthy Subjects. Molecules (Basel, Switzerland) 2019. 24 (16).

Knaub, Katharina, Sartorius, Tina, Dharsono, Tanita, Wacker, Roland, Wilhelm, Manfred, Schon, Christiane. A Novel Self-Emulsifying Drug Delivery System (SEDDS) Based on VESIsorb R Formulation Technology Improving the Oral Bioavailability of Cannabidiol in Healthy Subjects. Molecules (Basel, Switzerland) 2019. 24 (16).

Leweke, F. M., Piomelli, D., Pahlisch, F., Muhl, D., Gerth, C. W., Hoyer, C., Klosterkotter, J., Hellmich, M., and Koethe, D. Cannabidiol enhances anandamide signaling and alleviates psychotic symptoms of schizophrenia. Translational psychiatry 2012. 2, e94.

Lofwall, Michelle R., Babalonis, Shanna, Nuzzo, Paul A., Elayi, Samy Claude, and Walsh, Sharon L. Opioid withdrawal suppression efficacy of oral dronabinol in opioid dependent humans. Drug and Alcohol Dependence 2016. 164, 143-150.

Martin-Santos, R., Crippa, J. A., Batalla, A., Bhattacharyya, S., Atakan, Z., Borgwardt, S., Allen, P., Seal, M., Langohr, K., Farre, M., Zuardi, A. W., and McGuire, P. K. Acute effects of a single, oral dose of d9-tetrahydrocannabinol (THC) and cannabidiol (CBD) administration in healthy volunteers. Current Pharmaceutical Design 2012. 18 (32) 4966-4979.

Mathew, Roy J., Wilson, William H., and Davis, Robin. Postural syncope after marijuana: a transcranial Doppler study of the hemodynamics. Pharmacology, biochemistry, and behavior 2003. 75 (2) 309-318.

Miller, Ryan E., Brown, Timothy L., Lee, Stella, Tibrewal, Ishaan, Gaffney, Gary G., Milavetz, Gary, Hartman, Rebecca L., Gorelick, David A., Compton, Richard, Huestis, Marilyn A. Impact of cannabis and low alcohol concentration on divided attention tasks during driving. Traffic injury prevention 2020., 1-7.

Morrison, P. D., Zois, V., McKeown, D. A., Lee, T. D., Holt, D. W., Powell, J. F., Kapur, S., and Murray, R. M. The acute effects of synthetic intravenous Delta9-tetrahydrocannabinol on psychosis, mood and cognitive functioning. Psychological Medicine 2009. 39 (10) 1607-1616.

NCT00079560. Comparing the Effects of Smoked and Oral Marijuana in Individuals With HIV/AIDS. Https://clinicaltrials.gov/show/nct00079560 2004.

NCT00241592. Vaporization as a Smokeless Cannabis Delivery System. Https://clinicaltrials.gov/show/nct00241592 2005.

NCT00373503. Effect of Lofexidine and Oral THC on Marijuana Withdrawal and Relapse. Https://clinicaltrials.gov/show/nct00373503 2006.

NCT00490269. Ph1 Marinol Interaction Study - Part 2 - 1. Https://clinicaltrials.gov/show/nct00490269 2007.

NCT00624715. Cannabinoid Receptor Function & Alcoholism. Https://clinicaltrials.gov/show/nct00624715 2008.

NCT00946348. Cannabis and Schizophrenia: self-Medication and Agonist Treatment. Https://clinicaltrials.gov/show/nct00946348 2009.

NCT01322139. Sativex Thorough QT/QTc Study. Https://clinicaltrials.gov/show/nct01322139 2011.

NCT01323465. Study to Evaluate the Effect of Rifampicin, Ketoconazole, and Omeprazole on the Pharmacokinetics of Sativex. Https://clinicaltrials.gov/show/nct01323465 2011.

NCT01323569. Abuse Potential of Sativex. Https://clinicaltrials.gov/show/nct01323569 2011.

NCT01347762. Nabilone for Cannabis Dependence: a Pilot Study. Https://clinicaltrials.gov/show/nct01347762 2011.

NCT01748799. Fixed or Self-Titrated Dosages of Sativex on Cannabis Users. Https://clinicaltrials.gov/show/nct01748799 2012.

NCT01844687. Laboratory Study of Cannabidiol on the Effects of Smoked Marijuana. Https://clinicaltrials.gov/show/nct01844687 2013.

NCT01853020. Cannabinoids and Cerebellar-Motor Functioning. Https://clinicaltrials.gov/show/nct01853020 2013.

NCT02051387. Cannabidiol as a Different Type of an Antipsychotic: drug Delivery and Interaction Study. Https://clinicaltrials.gov/show/nct02051387 2013.

NCT02061020. Study of the Relationship Between Dose-concentration-effect of Delta-9-tetrahydrocannabinol (THC) and the Ability to Drive in Chronic or Occasional Cannabis Users. Https://clinicaltrials.gov/show/nct02061020 2013.

NCT02240160. A Randomised, Open-label, Three-way Crossover Study to Assess the Pharmacokinetics and Safety of Single Doses of Four Sprays of Sativex® in a Range of Oral pH Environments in Healthy Subjects. Https://clinicaltrials.gov/show/nct02240160 2014.

NCT02291562. Effects of THC and CBD on Human Episodic Memory Function. Https://clinicaltrials.gov/show/nct02291562 2014.

NCT02318537. Cannabidiol Oral Solution as an Adjunctive Therapy for Treatment of Participants With Inadequately Controlled Lennox-Gastaut Syndrome. Https://clinicaltrials.gov/show/nct02318537 2014.

NCT02318563. Cannabidiol Oral Solution as an Adjunctive Therapy for Treatment of Participants With Inadequately Controlled Dravet Syndrome. Https://clinicaltrials.gov/show/nct02318563 2014.

NCT02325011. An Study to Evaluate the Effects of Fluconazole on the Pharmacokinetics (PK) of Sativex® in Healthy Subjects With Cannabis Experience. Https://clinicaltrials.gov/show/nct02325011 2014.

NCT02472847. Cannabinoid Control of Fear Extinction Neural Circuits in Humans. Https://clinicaltrials.gov/show/nct02472847 2015.

NCT02751359. Characterization of the Analgesic Effect of CBD in Healthy, Normal Volunteers. Https://clinicaltrials.gov/show/nct02751359 2016.

NCT02783092. A Double-Blind Trial to Evaluate Efficacy and Safety of Cannabidiol as an add-on Therapy for Treatment in Refractory Epilepsy. Https://clinicaltrials.gov/show/nct02783092 2016.

NCT02844933. Cannabidiol Oral Solution for the Treatment of Subjects With Prader-Willi Syndrome. Https://clinicaltrials.gov/show/nct02844933 2016.

NCT02902081. Cannabidiol and Emotional Stimuli. Https://clinicaltrials.gov/show/nct02902081 2016.

NCT02983773. Marijuana's Impact on Alcohol Motivation and Consumption. Https://clinicaltrials.gov/show/nct02983773 2016.

NCT03008005. Effects of Delta-9 Tetrahydrocannabinol (THC) on Retention of Memory for Fear Extinction Learning in PTSD: r61 Study. Https://clinicaltrials.gov/show/nct03008005 2016.

NCT03164512. The Pharmacokinetics and Pharmacodynamics of Oral and Vaporized Cannabidiol. Https://clinicaltrials.gov/show/nct03164512 2017.

NCT03191084. Examine the Feasibility of a Standardized Field Test for Marijuana Impairment: laboratory Evaluations. Https://clinicaltrials.gov/show/nct03191084 2017.

NCT03201835. 5-Way Crossover Study to Compare the Safety, Tolerability and Pharmacokinetics of New Oral Cannabinoid Formulations Administered as Single Doses, With Buccal Sativex®, in Healthy Volunteers. Https://clinicaltrials.gov/show/nct03201835 2017.

NCT03215940. Treatment of Chronic Pain With Cannabidiol (CBD) and Delta-9-tetrahydrocannabinol (THC). Https://clinicaltrials.gov/show/nct03215940 2017.

NCT03251326. Nabilone in Cannabis Users With PTSD. Https://clinicaltrials.gov/show/nct03251326 2017.

NCT03627117. Influence of CBD on Episodic Memory in Healthy Subjects. Https://clinicaltrials.gov/show/nct03627117 2018.

NCT03656029. Dose-response of Cannabis and Driving. Https://clinicaltrials.gov/show/nct03656029 2018.

NCT03705559. Opioid and Cannabinoid Interactions. Https://clinicaltrials.gov/show/nct03705559 2018.

NCT03809546. Individual Differences in Drug Response. Https://clinicaltrials.gov/show/nct03809546 2019.

NCT03832816. Behavioral and Physiological Effects of THC and CBD. Https://clinicaltrials.gov/show/nct03832816 2019.

NCT03904849. Cannabidiol for Alcohol Use Disorder. Https://clinicaltrials.gov/show/nct03904849 2019.

NCT03907163. The Effect of Tetrahydrocannabinol on Ocular Hemodynamics in Healthy Subjects. Https://clinicaltrials.gov/show/nct03907163 2019.

NCT03936907. Comparison of Safety, Tolerability and Pharmacokinetics of Medical Grade Cannabis (MGC) Orally Disintegrating Tablets With Buccal Sativex®, in Healthy Adult Volunteers. Https://clinicaltrials.gov/show/nct03936907 2019.

Norberg, M. M., Barnier, E., Weidemann, G., Chakerian, K., Cornish, J. L., Rapee, R. M. Randomised pilot study of cannabis cue exposure: reducing cue reactivity while building tolerance. Clinical psychologist 2018. 22 (2) 126‐136.

NTR1840. Alcohol and cannabis. Http://www.who.int/trialsearch/trial2.aspx? Trialid=ntr1840 2009.

Pezdek, K., Abed, E., Reisberg, D. Marijuana impairs the accuracy of eyewitness memory and the confidence-accuracy relationship too. Journal of applied research in memory and cognition 2020., No Pagination Specified.

Rabinak, Christine A., Blanchette, Ashley, Zabik, Nicole L., Peters, Craig, Marusak, Hilary A., Iadipaolo, Allesandra, Elrahal, Farrah. Cannabinoid modulation of corticolimbic activation to threat in trauma-exposed adults: a preliminary study. Psychopharmacology 2020. 237 (6) 1813-1826.

Ranganathan, Mohini, Sewell, R. Andrew, Carbuto, Michelle, Elander, Jacqueline, Schnakenberg, Ashley, Radhakrishnan, Rajiv, Pittman, Brian, and D'Souza, Deepak Cyril. Effects of DELTA9-tetrahydrocannabinol in individuals with a familial vulnerability to alcoholism. Psychopharmacology 2014. 231 (12) 2385-2393.

Redmond, William John, Goffaux, Philippe, Potvin, Stephane, and Marchand, Serge. Analgesic and antihyperalgesic effects of nabilone on experimental heat pain. Current medical research and opinion 2008. 24 (4) 1017-1024.

Sellers, Edward M., Schoedel, Kerri, Bartlett, Cindy, Romach, Myroslava, Russo, Ethan B., Stott, Colin G., Wright, Stephen, White, Linda, Duncombe, Paul, and Chen, Chien Feng. A Multiple-Dose, Randomized, Double-Blind, Placebo-Controlled, Parallel-Group QT/QTc Study to Evaluate the Electrophysiologic Effects of THC/CBD Spray. Clinical pharmacology in drug development 2013. 2 (3) 285-294.

Sewell, R. Andrew, Schnakenberg, Ashley, Elander, Jacqueline, Radhakrishnan, Rajiv, Williams, Ashley, Skosnik, Patrick D., Pittman, Brian, Ranganathan, Mohini, and D'Souza, D. Cyril. Acute effects of THC on time perception in frequent and infrequent cannabis users. Psychopharmacology 2013. 226 (2) 401-413.

Skalski, Linda M., Gunn, Rachel L., Caswell, Amy, Maisto, Stephen, and Metrik, Jane. Sex-related marijuana expectancies as predictors of sexual risk behavior following smoked marijuana challenge. Experimental and clinical psychopharmacology 2017. 25 (5) 402-411.

Spronk, Desiree B., de Bruijn, Ellen R. A., Van Wel, Janelle H. P., Ramaekers, Johannes G., and Verkes, Robbert J. Acute effects of cocaine and cannabis on response inhibition in humans: an ERP investigation. Addiction Biology 2016. 21 (6) 1186-1198.

Spronk, Desiree B., Verkes, Robbert J., Cools, Roshan, Franke, Barbara, Van Wel, Janelle H. P., Ramaekers, Johannes G., and de Bruijn, Ellen R. A. Opposite effects of cannabis and cocaine on performance monitoring. European neuropsychopharmacology : the journal of the European College of Neuropsychopharmacology 2016. 26 (7) 1127-1139.

Spronk, Desiree, Dumont, Glenn J. H., Verkes, Robbert J., and de Bruijn, Ellen R. A. Acute effects of delta-9-tetrahydrocannabinol on performance monitoring in healthy volunteers. Frontiers in behavioral neuroscience 2011. 5, 59.

Taylor, Lesley, Crockett, Julie, Tayo, Bola, Checketts, Daniel, Sommerville, Kenneth. Abrupt withdrawal of cannabidiol (CBD): A randomized trial. Epilepsy & behavior : E&B 2020. 104 (Pt A) 106938.

Taylor, Lesley, Gidal, Barry, Blakey, Graham, Tayo, Bola, and Morrison, Gilmour. A Phase I, Randomized, Double-Blind, Placebo-Controlled, Single Ascending Dose, Multiple Dose, and Food Effect Trial of the Safety, Tolerability and Pharmacokinetics of Highly Purified Cannabidiol in Healthy Subjects. CNS Drugs 2018. 32 (11) 1053-1067.

van Amerongen, G., Siebenga, P., de Kam, M. L., Hay, J. L., and Groeneveld, G. J. Effect profile of paracetamol, DELTA9-THC and promethazine using an evoked pain test battery in healthy subjects. European journal of pain (London, England) 2018. 22 (7) 1331-1342.

Weizman, Libat, Dayan, Lior, Brill, Silviu, Nahman-Averbuch, Hadas, Hendler, Talma, Jacob, Giris, and Sharon, Haggai. Cannabis analgesia in chronic neuropathic pain is associated with altered brain connectivity. Neurology 2018. 91 (14) e1285-e1294.

Wilson, Robin, Bossong, Matthijs G., Appiah-Kusi, Elizabeth, Petros, Natalia, Brammer, Michael, Perez, Jesus, Allen, Paul, McGuire, Philip, Bhattacharyya, Sagnik. Cannabidiol attenuates insular dysfunction during motivational salience processing in subjects at clinical high risk for psychosis. Translational psychiatry 2019. 9 (1) 203.

A mix of older and younger adults, but no cannabis-related findings for older adults

[Author not listed]. Cannabidiol presents an inverted U-shaped dose-response curve in a simulated public speaking test. Revista brasileira de psiquiatria (sao paulo, brazil : 1999) 2019. 41 (1) 9‐14.

Abrams, D. I., Jay, C. A., Shade, S. B., Vizoso, H., Reda, H., Press, S., Kelly, M. E., Rowbotham, M. C., and Petersen, K. L. Cannabis in painful HIV-associated sensory neuropathy: a randomized placebo-controlled trial. Neurology 2007. 68 (7) 515-521.

Abrams, Donald I., Couey, Paul, Dixit, Niharika, Sagi, Varun, Hagar, Ward, Vichinsky, Elliott, Kelly, Mary Ellen, Connett, John E., Gupta, Kalpna. Effect of Inhaled Cannabis for Pain in Adults With Sickle Cell Disease: A Randomized Clinical Trial. JAMA network open 2020. 3 (7) e2010874.

Abrams, Donald I., Hilton, Joan F., Leiser, Roslyn J., Shade, Starley B., Elbeik, Tarek A., Aweeka, Francesca T., Benowitz, Neal L., Bredt, Barry M., Kosel, Bradley, Aberg, Judith A., Deeks, Steven G., Mitchell, Thomas F., Mulligan, Kathleen, Bacchetti, Peter, McCune, Joseph M., and Schambelan, Morris. Short-term effects of cannabinoids in patients with HIV-1 infection: a randomized, placebo-controlled clinical trial. Annals of Internal Medicine 2003. 139 (4) 258-266.

ACTRN12615000414516. An evaluative study on the safety and efficacy of standard anti-emetics compared to standard anti-emetics plus natural cannabinoids extract for the treatment of chemotherapy induced nausea and vomiting. Http://www.who.int/trialsearch/trial2.aspx? Trialid=actrn12615000414516 2015.

ACTRN12616001036404. CannabisCINV: a placebo-controlled trial evaluating an oral THC/CBD cannabis extract for secondary prevention of chemotherapy-induced nausea and vomiting in patients of any known malignancy receiving chemotherapy. Http://www.who.int/trialsearch/trial2.aspx? Trialid=actrn12616001036404 2016.

ACTRN12617001287325. A Phase 2 Randomised, Double Blind Clinical Trial assessing the Tolerability of Two different Ratios of Medicinal Cannabis in patients with Glioblastoma multiforme (GBM). Http://www.who.int/trialsearch/trial2.aspx? Trialid=actrn12617001287325 2017.

ACTRN12618000487213. The CANBACK trial, to determine the efficacy of oral cannabidiol, when compared to placebo, as an adjunct for the treatment of acute non-traumatic low back pain. Http://www.who.int/trialsearch/trial2.aspx? Trialid=actrn12618000487213 2018.

ACTRN12618000545268. A Randomised, Double Blind, Placebo-Controlled Trial of Medicinal Cannabis in Adults with Tourette's Syndrome. Http://www.who.int/trialsearch/trial2.aspx? Trialid=actrn12618000545268 2018.

Almog, Shlomo, Aharon-Peretz, Judith, Vulfsons, Simon, Ogintz, Miri, Abalia, Hadas, Lupo, Tal, Hayon, Yael, Eisenberg, Elon. The pharmacokinetics, efficacy, and safety of a novel selective-dose cannabis inhaler in patients with chronic pain: A randomized, double-blinded, placebo-controlled trial. European journal of pain (London, England) 2020. 24 (8) 1505-1516.

Andries, A., Frystyk, J., Flyvbjerg, A., Støving, R. K. Dronabinol in severe, enduring anorexia nervosa: a randomized controlled trial. International journal of eating disorders 2014. 47 (1) 18‐23.

Andries, Alin, Frystyk, Jan, Flyvbjerg, Allan, and Stoving, Rene Klinkby. Dronabinol in severe, enduring anorexia nervosa: a randomized controlled trial. The International journal of eating disorders 2014. 47 (1) 18-23.

Andries, Alin, Gram, Bibi, and Stoving, Rene Klinkby. Effect of dronabinol therapy on physical activity in anorexia nervosa: a randomised, controlled trial. Eating and weight disorders : EWD 2015. 20 (1) 13-21.

Aragona, Massimiliano, Onesti, Emanuela, Tomassini, Valentina, Conte, Antonella, Gupta, Shiva, Gilio, Francesca, Pantano, Patrizia, Pozzilli, Carlo, and Inghilleri, Maurizio. Psychopathological and cognitive effects of therapeutic cannabinoids in multiple sclerosis: a double-blind, placebo controlled, crossover study. Clinical Neuropharmacology 2009. 32 (1) 41-47.

Ball, Susan, Vickery, Jane, Hobart, Jeremy, Wright, Dave, Green, Colin, Shearer, James, Nunn, Andrew, Cano, Mayam Gomez, MacManus, David, Miller, David, Mallik, Shahrukh, and Zajicek, John. The Cannabinoid Use in Progressive Inflammatory brain Disease (CUPID) trial: a randomised double-blind placebo-controlled parallel-group multicentre trial and economic evaluation of cannabinoids to slow progression in multiple sclerosis. Health technology assessment (Winchester, England) 2015. 19 (12) vii-187.

Ben-Menachem, Elinor, Gunning, Boudewijn, Arenas Cabrera, Carmen Maria, VanLandingham, Kevan, Crockett, Julie, Critchley, David, Wray, Louise, Tayo, Bola, Morrison, Gilmour, Toledo, Manuel. A Phase II Randomized Trial to Explore the Potential for Pharmacokinetic Drug-Drug Interactions with Stiripentol or Valproate when Combined with Cannabidiol in Patients with Epilepsy. CNS drugs 2020. 34 (6) 661-672.

Berman, Jonathan S., Symonds, Catherine, and Birch, Rolfe. Efficacy of two cannabis based medicinal extracts for relief of central neuropathic pain from brachial plexus avulsion: results of a randomised controlled trial. Pain 2004. 112 (3) 299-306.

Bhattacharyya, Sagnik, Sainsbury, Thomas, Nosarti, Chiara, Atakan, Zerrin, McGuire, P. K., Allen, Paul, Giampietro, Vincent, Brammer, Michael. Increased hippocampal engagement during learning as a marker of sensitivity to psychotomimetic effects of delta-9-THC. Psychological medicine 2018. 48 (16) 2748-2756.

Bhattacharyya, Sagnik, Wilson, Robin, Appiah-Kusi, Elizabeth, O'Neill, Aisling, Brammer, Michael, Perez, Jesus, Murray, Robin, Allen, Paul, Bossong, Matthijs G., and McGuire, Philip. Effect of Cannabidiol on Medial Temporal, Midbrain, and Striatal Dysfunction in People at Clinical High Risk of Psychosis: A Randomized Clinical Trial. JAMA Psychiatry 2018. 75 (11) 1107-1117.

Bisaga, Adam, Sullivan, Maria A., Glass, Andrew, Mishlen, Kaitlyn, Pavlicova, Martina, Haney, Margaret, Raby, Wilfrid N., Levin, Frances R., Carpenter, Kenneth M., Mariani, John J., and Nunes, Edward V. The effects of dronabinol during detoxification and the initiation of treatment with extended release naltrexone. Drug and Alcohol Dependence 2015. 154, 38-45.

Bloomfield, Michael A. P., Green, Sebastian F., Hindocha, Chandni, Yamamori, Yumeya, Yim, Jocelyn Lok Ling, Jones, Augustus P. M., Walker, Hannah R., Tokarczuk, Pawel, Statton, Ben, Howes, Oliver D., Curran, H. Valerie, Freeman, Tom P. The effects of acute cannabidiol on cerebral blood flow and its relationship to memory: An arterial spin labelling magnetic resonance imaging study. Journal of psychopharmacology (Oxford, England) 2020. 34 (9) 981-989.

Boggs, Douglas L., Cortes-Briones, Jose A., Surti, Toral, Luddy, Christina, Ranganathan, Mohini, Cahill, John D., Sewell, Andrew R., D'Souza, Deepak C., and Skosnik, Patrick D. The dose-dependent psychomotor effects of intravenous delta-9-tetrahydrocannabinol (DELTA9-THC) in humans. Journal of psychopharmacology (Oxford, England) 2018. 32 (12) 1308-1318.

Boggs, Douglas L., Surti, Toral, Gupta, Aarti, Gupta, Swapnil, Niciu, Mark, Pittman, Brian, Schnakenberg Martin, Ashley M., Thurnauer, Halle, Davies, Andrew, D'Souza, Deepak C., and Ranganathan, Mohini. The effects of cannabidiol (CBD) on cognition and symptoms in outpatients with chronic schizophrenia a randomized placebo controlled trial. Psychopharmacology 2018. 235 (7) 1923-1932.

Bredt, Barry M., Higuera-Alhino, Dana, Shade, Starley B., Hebert, Samuel J., McCune, Joseph M., and Abrams, Donald I. Short-term effects of cannabinoids on immune phenotype and function in HIV-1-infected patients. Journal of Clinical Pharmacology 2002. 42 (S1) 82S-89S.

Brezing, C. A., Choi, C. J., Pavlicova, M., Brooks, D., Mahony, A. L., Mariani, J. J., Levin, F. R. Abstinence and reduced frequency of use are associated with improvements in quality of life among treatment-seekers with cannabis use disorder. The american journal on addictions 2018. 27 (2) 101‐107.

Brown, Timothy L., Richard, Christian, Meghdadi, Amir, Poole, Jared, Fink, Abigail, Stevanovic Karic, Marija, McConnell, Marissa, Rupp, Greg, Schmitt, Rose, Gaffney, Gary G., Milavetz, Gary, Berka, Chris. EEG biomarkers acquired during a short, straight-line simulated drive to predict impairment from cannabis intoxication. Traffic injury prevention 2020., 1-5.

Buggy, Donal J., Toogood, Lynn, Maric, Shelagh, Sharpe, Paul, Lambert, David G., and Rowbotham, David J. Lack of analgesic efficacy of oral delta-9-tetrahydrocannabinol in postoperative pain. Pain 2003. 106 (1-2) 169-172.

Carley, David W., Prasad, Bharati, Reid, Kathryn J., Malkani, Roneil, Attarian, Hryar, Abbott, Sabra M., Vern, Boris, Xie, Hui, Yuan, Chengbo, and Zee, Phyllis C. Pharmacotherapy of Apnea by Cannabimimetic Enhancement, the PACE Clinical Trial: Effects of Dronabinol in Obstructive Sleep Apnea. Sleep 2018. 41 (1).

Chaves, Carolina, Bittencourt, Paulo Cesar T., Pelegrini, Andreia. Ingestion of a THC-Rich Cannabis Oil in People with Fibromyalgia: A Randomized, Double-Blind, Placebo-Controlled Clinical Trial. Pain medicine (Malden, Mass.) 2020. 21 (10) 2212-2218.

Chmiel, James F., Flume, Patrick, Downey, Damian G., Dozor, Allen J., Colombo, Carla, Mazurek, Henryk, Sapiejka, Ewa, Rachel, Marta, Constantine, Scott, Conley, Brian, Dgetluck, Nancy, Dinh, Quinn, White, Barbara, Elborn, J. Stuart, Lenabasum, J. B. T. C. F. Study Group. Safety and efficacy of lenabasum in a phase 2 randomized, placebo-controlled trial in adults with cystic fibrosis. Journal of cystic fibrosis : official journal of the European Cystic Fibrosis Society 2021. 20 (1) 78-85.

Colizzi, Marco, McGuire, Philip, Giampietro, Vincent, Williams, Steve, Brammer, Mick, and Bhattacharyya, Sagnik. Modulation of acute effects of delta-9-tetrahydrocannabinol on psychotomimetic effects, cognition and brain function by previous cannabis exposure. European neuropsychopharmacology : the journal of the European College of Neuropsychopharmacology 2018. 28 (7) 850-862.

Colizzi, Marco, McGuire, Philip, Giampietro, Vincent, Williams, Steve, Brammer, Mick, and Bhattacharyya, Sagnik. Previous cannabis exposure modulates the acute effects of delta-9-tetrahydrocannabinol on attentional salience and fear processing. Experimental and clinical psychopharmacology 2018. 26 (6) 582-598.

Colizzi, Marco, Weltens, Nathalie, Lythgoe, David J., Williams, Steve Cr, Van Oudenhove, Lukas, Bhattacharyya, Sagnik. Differential sensitivity to the acute psychotomimetic effects of delta-9-tetrahydrocannabinol associated with its differential acute effects on glial function and cortisol. Psychological medicine 2020., 1-8.

Colizzi, Marco, Weltens, Nathalie, McGuire, Philip, Lythgoe, David, Williams, Steve, Van Oudenhove, Lukas, and Bhattacharyya, Sagnik. Delta-9-tetrahydrocannabinol increases striatal glutamate levels in healthy individuals: implications for psychosis. Molecular psychiatry 2019.

Colizzi, Marco, Weltens, Nathalie, McGuire, Philip, Van Oudenhove, Lukas, and Bhattacharyya, Sagnik. Descriptive Psychopathology of the Acute Effects of Intravenous Delta-9-Tetrahydrocannabinol Administration in Humans. Brain sciences 2019. 9 (4).

Collin, C., Davies, P., Mutiboko, I. K., Ratcliffe, S., and Sativex Spasticity in MS Study Group. Randomized controlled trial of cannabis-based medicine in spasticity caused by multiple sclerosis. European journal of neurology 2007. 14 (3) 290-296.

Collin, C., Ehler, E., Waberzinek, G., Alsindi, Z., Davies, P., Powell, K., Notcutt, W., O'Leary, C., Ratcliffe, S., Novakova, I., Zapletalova, O., Pikova, J., and Ambler, Z. A double-blind, randomized, placebo-controlled, parallel-group study of Sativex, in subjects with symptoms of spasticity due to multiple sclerosis. Neurological research 2010. 32 (5) 451-459.

Cooper, Ruth E., Williams, Emma, Seegobin, Seth, Tye, Charlotte, Kuntsi, Jonna, and Asherson, Philip. Cannabinoids in attention-deficit/hyperactivity disorder: A randomised-controlled trial. European neuropsychopharmacology : the journal of the European College of Neuropsychopharmacology 2017. 27 (8) 795-808.

Corey-Bloom, Jody, Wolfson, Tanya, Gamst, Anthony, Jin, Shelia, Marcotte, Thomas D., Bentley, Heather, and Gouaux, Ben. Smoked cannabis for spasticity in multiple sclerosis: a randomized, placebo-controlled trial. CMAJ : Canadian Medical Association journal = journal de l'Association medicale canadienne 2012. 184 (10) 1143-1150.

Curtis, Adrienne, Mitchell, Ian, Patel, Smitaa, Ives, Natalie, and Rickards, Hugh. A pilot study using nabilone for symptomatic treatment in Huntington's disease. Movement disorders : official journal of the Movement Disorder Society 2009. 24 (15) 2254-2259.

de Bruijn, Suzanne E. M., de Graaf, Cees, Witkamp, Renger F., and Jager, Gerry. Explorative Placebo-Controlled Double-Blind Intervention Study with Low Doses of Inhaled DELTA9-Tetrahydrocannabinol and Cannabidiol Reveals No Effect on Sweet Taste Intensity Perception and Liking in Humans. Cannabis and Cannabinoid Research 2017. 2 (1) 114-122.

de la Salle, Sara, Inyang, Lawrence, Impey, Danielle, Smith, Dylan, Choueiry, Joelle, Nelson, Renee, Heera, Jasmit, Baddeley, Ashley, Ilivitsky, Vadim, Knott, Verner. Acute separate and combined effects of cannabinoid and nicotinic receptor agonists on MMN-indexed auditory deviance detection in healthy humans. Pharmacology, biochemistry, and behavior 2019. 184, 172739.

de Vries, Marjan, van Rijckevorsel, Dagmar C. M., Vissers, Kris C. P., Wilder-Smith, Oliver H. G., and van Goor, Harry. Single dose delta-9-tetrahydrocannabinol in chronic pancreatitis patients: analgesic efficacy, pharmacokinetics and tolerability. British Journal of Clinical Pharmacology 2016. 81 (3) 525-537.

de Vries, Marjan, van Rijckevorsel, Dagmar C. M., Vissers, Kris C. P., Wilder-Smith, Oliver H. G., van Goor, Harry, and Pain and Nociception Neuroscience Research Group. Tetrahydrocannabinol Does Not Reduce Pain in Patients With Chronic Abdominal Pain in a Phase 2 Placebo-controlled Study. Clinical gastroenterology and hepatology : the official clinical practice journal of the American Gastroenterological Association 2017. 15 (7) 1079-1086.

Devinsky, Orrin, Patel, Anup D., Cross, J. Helen, Villanueva, Vicente, Wirrell, Elaine C., Privitera, Michael, Greenwood, Sam M., Roberts, Claire, Checketts, Daniel, VanLandingham, Kevan E., Zuberi, Sameer M., and Study Group. Effect of Cannabidiol on Drop Seizures in the Lennox-Gastaut Syndrome. The New England journal of medicine 2018. 378 (20) 1888-1897.

DRKS00004526. Longterm effects of cannabis and amphetamin use and social-emotional brainfunctions. Http://www.who.int/trialsearch/trial2.aspx? Trialid=drks00004526 2013.

D'Souza, Deepak Cyril, Abi-Saab, Walid Michel, Madonick, Steven, Forselius-Bielen, Kimberlee, Doersch, Anne, Braley, Gabriel, Gueorguieva, Ralitza, Cooper, Thomas B., and Krystal, John Harrison. Delta-9-tetrahydrocannabinol effects in schizophrenia: implications for cognition, psychosis, and addiction. Biological Psychiatry 2005. 57 (6) 594-608.

Duran, Marta, Perez, Eulalia, Abanades, Sergio, Vidal, Xavier, Saura, Cristina, Majem, Margarita, Arriola, Edurne, Rabanal, Manel, Pastor, Antoni, Farre, Magi, Rams, Neus, Laporte, Joan Ramon, and Capella, Dolors. Preliminary efficacy and safety of an oromucosal standardized cannabis extract in chemotherapy-induced nausea and vomiting. British Journal of Clinical Pharmacology 2010. 70 (5) 656-663.

Eibach, Luca, Scheffel, Simone, Cardebring, Madeleine, Lettau, Marie, Ozgur Celik, M., Morguet, Andreas, Roehle, Robert, Stein, Christoph. Cannabidivarin for HIV-Associated Neuropathic Pain: A Randomized, Blinded, Controlled Clinical Trial. Clinical pharmacology and therapeutics 2021. 109 (4) 1055-1062.

Ellis, Ronald J., Toperoff, Will, Vaida, Florin, van den Brande, Geoffrey, Gonzales, James, Gouaux, Ben, Bentley, Heather, and Atkinson, J. Hampton. Smoked medicinal cannabis for neuropathic pain in HIV: a randomized, crossover clinical trial. Neuropsychopharmacology : official publication of the American College of Neuropsychopharmacology 2009. 34 (3) 672-680.

Esfandyari, Tuba, Camilleri, Michael, Busciglio, Irene, Burton, Duane, Baxter, Kari, and Zinsmeister, Alan R. Effects of a cannabinoid receptor agonist on colonic motor and sensory functions in humans: a randomized, placebo-controlled study. American journal of physiology.Gastrointestinal and liver physiology 2007. 293 (1) G137-G145.

EUCTR2004-002509-63-GB. A double blind, randomised, placebo controlled, parallel group study of Sativex, in subjects with symptoms of spasticity due to multiple sclerosis - N/A. Http://www.who.int/trialsearch/trial2.aspx? Trialid=euctr2004-002509-63-gb 2005.

EUCTR2005-000393-47-IT. Effect of Cannabis Based Medicine Extract Sativex on brain function as assessed by fMRI and neurophysiologic evaluation in patients with Multiple Sclerosis a double blind, randomised, placebo-controlled, crossover study. Http://www.who.int/trialsearch/trial2.aspx? Trialid=euctr2005-000393-47-it 2007.

EUCTR2006-005910-11-DE. A two-phase Phase 3 study of the safety and efficacy of Sativex®, in the symptomatic relief of spasticity in subjects with spasticity due to multiple sclerosis: phase A – single blind response assessment; Phase B - double blind, randomised, placebo controlled, parallel group study. Http://www.who.int/trialsearch/trial2.aspx? Trialid=euctr2006-005910-11-de 2008.

EUCTR2007-000444-27-AT. Treatment of Patients Suffering from Nausea/Vomiting with Dronabinol (tetrahydrocannabinol) Comparsion of three dosages versus standard therapy in a cross-over design. Http://www.who.int/trialsearch/trial2.aspx? Trialid=euctr2007-000444-27-at 2007.

EUCTR2007-002138-13-GB. A placebo controlled, parallel group, randomised withdrawal study of subjects with symptoms of spasticity due to multiple sclerosis who are receiving long-term GW-1000-02 (Sativex®). Http://www.who.int/trialsearch/trial2.aspx? Trialid=euctr2007-002138-13-gb 2007.

EUCTR2009-017080-41-GB. A study to asses the effects of cannabidiol (CBD) on liver fat levels with people who have fatty liver disease. Http://www.who.int/trialsearch/trial2.aspx? Trialid=euctr2009-017080-41-gb 2010.

EUCTR2010-022808-40-IT. A fase II, randomized, Double-Blind, Placebo-Controlled, Multicentre Study for the Safety and Efficacy on Spasticity Symptoms of a Cannabis Sativa Extract in Motor Neuron Disease Patients - ND. Http://www.who.int/trialsearch/trial2.aspx? Trialid=euctr2010-022808-40-it 2011.

EUCTR2011-000180-28-GB. GWMD09126 - GWP42003: gWP42004 antipsychotic(s) weight gain treatment. Http://www.who.int/trialsearch/trial2.aspx? Trialid=euctr2011-000180-28-gb 2011.

EUCTR2011-002258-30-IT. CLINICAL STUDY TO EVALUATE THE EFFECTIVENESS OF Sativex in relieving pain PEOPLE AFFECTED BY MULTIPLE SCLEROSIS. Http://www.who.int/trialsearch/trial2.aspx? Trialid=euctr2011-002258-30-it 2012.

EUCTR2012-000730-19-NL. ?9-THC (Namisol®) in Chronic Pancreatitis Patients Suffering From Persistent Abdominal Pain. Http://www.who.int/trialsearch/trial2.aspx? Trialid=euctr2012-000730-19-nl 2012.

EUCTR2012-000812-27-NL. ?9-THC (Namisol®) in persistent postsurgical pain. Http://www.who.int/trialsearch/trial2.aspx? Trialid=euctr2012-000812-27-nl 2012.

EUCTR2013-000212-22-RO. GWP42003 as adjunctive therapy to first line antipsychotics in schizophrenia and related psychotic disorders. Http://www.who.int/trialsearch/trial2.aspx? Trialid=euctr2013-000212-22-ro 2014.

EUCTR2013-001140-61-GB. GWP42004 as add on to metformin in the treatment of participants with Type 2 diabetes. Http://www.who.int/trialsearch/trial2.aspx? Trialid=euctr2013-001140-61-gb 2013.

EUCTR2014-002594-11-ES. A study of GWP42006 in people with focal seizures. Http://www.who.int/trialsearch/trial2.aspx? Trialid=euctr2014-002594-11-es 2015.

EUCTR2014-004094-17-NL. Does adding cannabidiol (a component of cannabis) enhance the treatment of phobias where patients are confronted with their fears (exposure treatment). Http://www.who.int/trialsearch/trial2.aspx? Trialid=euctr2014-004094-17-nl 2015.

EUCTR2015-004451-40-AT. SATIVEX® AS ADD-ON THERAPY VS. FURTHER OPTIMIZED FIRST-LINE ANTISPASTICS. Http://www.who.int/trialsearch/trial2.aspx? Trialid=euctr2015-004451-40-at 2016.

EUCTR2017-003574-13-DK. Medical cannabis for the treatment of pain in patients with hand osteoarthritis and psoriatic arthritis. Http://www.who.int/trialsearch/trial2.aspx? Trialid=euctr2017-003574-13-dk 2018.

Farokhnia, Mehdi, McDiarmid, Gray R., Newmeyer, Matthew N., Munjal, Vikas, Abulseoud, Osama A., Huestis, Marilyn A., Leggio, Lorenzo. Effects of oral, smoked, and vaporized cannabis on endocrine pathways related to appetite and metabolism: a randomized, double-blind, placebo-controlled, human laboratory study. Translational psychiatry 2020. 10 (1) 71.

Feinstein, Anthony, Meza, Cecilia, Stefan, Cristiana, Staines, W. Richard. Discontinuing cannabis improves depression in people with multiple sclerosis: A short report. Multiple sclerosis (Houndmills, Basingstoke, England) 2021. 27 (4) 636-639.

Fox, P., Bain, P. G., Glickman, S., Carroll, C., and Zajicek, J. The effect of cannabis on tremor in patients with multiple sclerosis. Neurology 2004. 62 (7) 1105-1109.

Fox, Susan H., Kellett, Mark, Moore, A. Peter, Crossman, Alan R., and Brotchie, Jonathan M. Randomised, double-blind, placebo-controlled trial to assess the potential of cannabinoid receptor stimulation in the treatment of dystonia. Movement disorders : official journal of the Movement Disorder Society 2002. 17 (1) 145-149.

Frank, B., Serpell, M. G., Hughes, J., Matthews, J. N. S., and Kapur, D. Comparison of analgesic effects and patient tolerability of nabilone and dihydrocodeine for chronic neuropathic pain: randomised, crossover, double blind study. BMJ (Clinical research ed.) 2008. 336 (7637) 199-201.

Freeman, R. M., Adekanmi, O., Waterfield, M. R., Waterfield, A. E., Wright, D., and Zajicek, J. The effect of cannabis on urge incontinence in patients with multiple sclerosis: a multicentre, randomised placebo-controlled trial (CAMS-LUTS). International urogynecology journal and pelvic floor dysfunction 2006. 17 (6) 636-641.

Freeman, Tom P., Hindocha, Chandni, Baio, Gianluca, Shaban, Natacha D. C., Thomas, Emily M., Astbury, Danica, Freeman, Abigail M., Lees, Rachel, Craft, Sam, Morrison, Paul D., Bloomfield, Michael A. P., O'Ryan, Dominic, Kinghorn, Jane, Morgan, Celia J. A., Mofeez, Ali, Curran, H. Valerie. Cannabidiol for the treatment of cannabis use disorder: a phase 2a, double-blind, placebo-controlled, randomised, adaptive Bayesian trial. The lancet. Psychiatry 2020. 7 (10) 865-874.

Gilman, J. M., Yücel, M. A., Pachas, G. N., Potter, K., Levar, N., Broos, H., Manghis, E. M., Schuster, R. M., Evins, A. E. Delta-9-tetrahydrocannabinol intoxication is associated with increased prefrontal activation as assessed with functional near-infrared spectroscopy: a report of a potential biomarker of intoxication. NeuroImage 2019. 197, 575‐585.

Gilman, Jodi M., Yucel, Meryem A., Pachas, Gladys N., Potter, Kevin, Levar, Nina, Broos, Hannah, Manghis, Eve M., Schuster, Randi M., and Evins, A. Eden. Delta-9-tetrahydrocannabinol intoxication is associated with increased prefrontal activation as assessed with functional near-infrared spectroscopy: A report of a potential biomarker of intoxication. NeuroImage 2019. 197, 575-585.

Goodwin, R. S., Gustafson, R. A., Barnes, A., Nebro, W., Moolchan, E. T., and Huestis, M. A. DELTA9-tetrahydrocannabinol, 11-hydroxy-DELTA9-tetrahydrocannabinol and 11-nor-9-carboxy-DELTA9-tetrahydrocannabinol in human plasma after controlled oral administration of cannabinoids. Therapeutic drug monitoring 2006. 28 (4) 545-551.

Grimison, P., Mersiades, A., Kirby, A., Lintzeris, N., Morton, R., Haber, P., Olver, I., Walsh, A., McGregor, I., Cheung, Y., Tognela, A., Hahn, C., Briscoe, K., Aghmesheh, M., Fox, P., Abdi, E., Clarke, S., Della-Fiorentina, S., Shannon, J., Gedye, C., Begbie, S., Simes, J., Stockler, M. Oral THC:CBD cannabis extract for refractory chemotherapy-induced nausea and vomiting: a randomised, placebo-controlled, phase II crossover trial. Annals of oncology : official journal of the European Society for Medical Oncology 2020. 31 (11) 1553-1560.

Hammoud, M. Z., Peters, C., Hatfield, J. R. B., Gorka, S. M., Phan, K. L., Milad, M. R., Rabinak, C. A. Influence of Δ9-tetrahydrocannabinol on long-term neural correlates of threat extinction memory retention in humans. Neuropsychopharmacology 2019. 44 (10) 1769‐1777.

Hammoud, Mira Z., Peters, Craig, Hatfield, Joshua R. B., Gorka, Stephanie M., Phan, K. Luan, Milad, Mohammed R., and Rabinak, Christine A. Influence of DELTA9-tetrahydrocannabinol on long-term neural correlates of threat extinction memory retention in humans. Neuropsychopharmacology : official publication of the American College of Neuropsychopharmacology 2019.

Hefner, K. R., Starr, M. J., and Curtin, J. J. Heavy marijuana use but not deprivation is associated with increased stressor reactivity. Journal of Abnormal Psychology 2018. 127 (4) 348-358.

Hindocha, C., Freeman, T. P., Xia, J. X., Shaban, N. D. C., and Curran, H. V. Acute memory and psychotomimetic effects of cannabis and tobacco both 'joint' and individually: a placebo-controlled trial. Psychological Medicine 2017. 47 (15) 2708-2719.

Hindocha, Chandni, Freeman, Tom P., Schafer, Grainne, Gardener, Chelsea, Das, Ravi K., Morgan, Celia J. A., and Curran, H. Valerie. Acute effects of delta-9-tetrahydrocannabinol, cannabidiol and their combination on facial emotion recognition: a randomised, double-blind, placebo-controlled study in cannabis users. European neuropsychopharmacology : the journal of the European College of Neuropsychopharmacology 2015. 25 (3) 325-334.

Hobbs, Jack M., Vazquez, Allegra R., Remijan, Nicholas D., Trotter, Roxanne E., McMillan, Thomas V., Freedman, Kimberly E., Wei, Yuren, Woelfel, Keith A., Arnold, Olivia R., Wolfe, Lisa M., Johnson, Sarah A., Weir, Tiffany L. Evaluation of pharmacokinetics and acute anti-inflammatory potential of two oral cannabidiol preparations in healthy adults. Phytotherapy research : PTR 2020. 34 (7) 1696-1703.

Hundal, Harneet, Lister, Rachel, Evans, Nicole, Antley, Angus, Englund, Amir, Murray, Robin M., Freeman, Daniel, and Morrison, Paul D. The effects of cannabidiol on persecutory ideation and anxiety in a high trait paranoid group. Journal of psychopharmacology (Oxford, England) 2018. 32 (3) 276-282.

Hurd, Y. L., Spriggs, S., Alishayev, J., Winkel, G., Gurgov, K., Kudrich, C., Oprescu, A. M., Salsitz, E. Cannabidiol for the Reduction of Cue-Induced Craving and Anxiety in Drug-Abstinent Individuals With Heroin Use Disorder: a Double-Blind Randomized Placebo-Controlled Trial. American journal of psychiatry 2019. 176 (11) 911‐922.

Hurd, Yasmin L., Spriggs, Sharron, Alishayev, Julia, Winkel, Gary, Gurgov, Kristina, Kudrich, Chris, Oprescu, Anna M., and Salsitz, Edwin. Cannabidiol for the Reduction of Cue-Induced Craving and Anxiety in Drug-Abstinent Individuals With Heroin Use Disorder: A Double-Blind Randomized Placebo-Controlled Trial. The American journal of psychiatry 2019., appiajp201918101191.

Irving, Peter M., Iqbal, Tariq, Nwokolo, Chuka, Subramanian, Sreedhar, Bloom, Stuart, Prasad, Neeraj, Hart, Ailsa, Murray, Charles, Lindsay, James O., Taylor, Adam, Barron, Rachel, and Wright, Stephen. A Randomized, Double-blind, Placebo-controlled, Parallel-group, Pilot Study of Cannabidiol-rich Botanical Extract in the Symptomatic Treatment of Ulcerative Colitis. Inflammatory Bowel Diseases 2018. 24 (4) 714-724.

ISRCTN05531690. Post-operative analgesic effects of an oral cannabinoid. Http://www.who.int/trialsearch/trial2.aspx? Trialid=isrctn05531690 2006.

ISRCTN16782845. A randomised, double-blind, placebo-controlled, cross-over pilot study using nabilone for symptomatic relief in patients with Huntington's disease. Http://www.who.int/trialsearch/trial2.aspx? Trialid=isrctn16782845 2005.

ISRCTN29199098. Efficacy of a topical cannabinoid preparation in decreasing symptoms of rheumatoid arthritis. Http://www.who.int/trialsearch/trial2.aspx? Trialid=isrctn29199098 2014.

ISRCTN58754671. A functional magnetic imaging (fMRI) study of the effects of cannabis-based medicines (CMEs) on the neutral activity associated with noxious thermal stimulation in healthy human volunteers (CRI.FM024). Protocol V2. FINAL. Http://www.who.int/trialsearch/trial2.aspx? Trialid=isrctn58754671 2004.

ISRCTN89498802. Does cannabidiol treatment lead to recovery of brain structure and function in cannabis users?. Http://www.who.int/trialsearch/trial2.aspx? Trialid=isrctn89498802 2017.

Issa, Mohammed A., Narang, Sanjeet, Jamison, Robert N., Michna, Edward, Edwards, Robert R., Penetar, David M., and Wasan, Ajay D. The subjective psychoactive effects of oral dronabinol studied in a randomized, controlled crossover clinical trial for pain. The Clinical journal of pain 2014. 30 (6) 472-478.

Jadoon, Khalid A., Tan, Garry D., and O'Sullivan, Saoirse E. A single dose of cannabidiol reduces blood pressure in healthy volunteers in a randomized crossover study. JCI insight 2017. 2 (12).

Jochimsen, P. R., Lawton, R. L., VerSteeg, K., Noyes, R., Jr. Effect of benzopyranoperidine, a delta-9-THC congener, on pain. Clin Pharmacol Ther 1978. 24 (2) 223-7.

Karst, Matthias, Salim, Kahlid, Burstein, Sumner, Conrad, Ingomar, Hoy, Ludwig, and Schneider, Udo. Analgesic effect of the synthetic cannabinoid CT-3 on chronic neuropathic pain: a randomized controlled trial. JAMA - Journal of the American Medical Association 2003. 290 (13) 1757-1762.

Kavia, R. B. C., De Ridder, D., Constantinescu, C. S., Stott, C. G., and Fowler, C. J. Randomized controlled trial of Sativex to treat detrusor overactivity in multiple sclerosis. Multiple sclerosis (Houndmills, Basingstoke, England) 2010. 16 (11) 1349-1359.

Kleine-Brueggeney, Maren, Greif, Robert, Brenneisen, Rudolf, Urwyler, Natalie, Stueber, Frank, and Theiler, Lorenz G. Intravenous Delta-9-Tetrahydrocannabinol to Prevent Postoperative Nausea and Vomiting: A Randomized Controlled Trial. Anesthesia and analgesia 2015. 121 (5) 1157-1164.

Kloft, Lilian, Otgaar, Henry, Blokland, Arjan, Monds, Lauren A., Toennes, Stefan W., Loftus, Elizabeth F., Ramaekers, Johannes G. Cannabis increases susceptibility to false memory. Proceedings of the National Academy of Sciences of the United States of America 2020. 117 (9) 4585-4589.

Klooker, T. K., Leliefeld, K. E. M., Van Den Wijngaard, R. M., and Boeckxstaens, G. E. E. The cannabinoid receptor agonist delta-9-tetrahydrocannabinol does not affect visceral sensitivity to rectal distension in healthy volunteers and IBS patients. Neurogastroenterology and motility : the official journal of the European Gastrointestinal Motility Society 2011. 23 (1) 30-e2.

Klumpers, Linda E., Beumer, Tim L., van Hasselt, Johan G. C., Lipplaa, Astrid, Karger, Lennard B., Kleinloog, H. Daniel, Freijer, Jan I., de Kam, Marieke L., and van Gerven, Joop M. A. Novel DELTA(9) -tetrahydrocannabinol formulation Namisol has beneficial pharmacokinetics and promising pharmacodynamic effects. British Journal of Clinical Pharmacology 2012. 74 (1) 42-53.

Knoller, Nachshon, Levi, Leon, Shoshan, Igal, Reichenthal, Eli, Razon, Nissim, Rappaport, Zvi H., and Biegon, Anat. Dexanabinol (HU-211) in the treatment of severe closed head injury: a randomized, placebo-controlled, phase II clinical trial. Critical care medicine 2002. 30 (3) 548-554.

Kosel, Bradley W., Aweeka, Francesca T., Benowitz, Neal L., Shade, Starley B., Hilton, Joan F., Lizak, Patricia S., and Abrams, Donald I. The effects of cannabinoids on the pharmacokinetics of indinavir and nelfinavir. AIDS (London, England) 2002. 16 (4) 543-550.

Kowal, Mikael A., Hazekamp, Arno, Colzato, Lorenza S., van Steenbergen, Henk, van der Wee, Nic J. A., Durieux, Jeffrey, Manai, Meriem, and Hommel, Bernhard. Cannabis and creativity: highly potent cannabis impairs divergent thinking in regular cannabis users. Psychopharmacology 2015. 232 (6) 1123-1134.

Kowal, Mikael A., van Steenbergen, Henk, Colzato, Lorenza S., Hazekamp, Arno, van der Wee, Nic J. A., Manai, Meriem, Durieux, Jeffrey, and Hommel, Bernhard. Dose-dependent effects of cannabis on the neural correlates of error monitoring in frequent cannabis users. European neuropsychopharmacology : the journal of the European College of Neuropsychopharmacology 2015. 25 (11) 1943-1953.

Langford, R. M., Mares, J., Novotna, A., Vachova, M., Novakova, I., Notcutt, W., and Ratcliffe, S. A double-blind, randomized, placebo-controlled, parallel-group study of THC/CBD oromucosal spray in combination with the existing treatment regimen, in the relief of central neuropathic pain in patients with multiple sclerosis. Journal of Neurology 2013. 260 (4) 984-997.

Lawn, Will, Freeman, Tom P., Pope, Rebecca A., Joye, Alyssa, Harvey, Lisa, Hindocha, Chandni, Mokrysz, Claire, Moss, Abigail, Wall, Matthew B., Bloomfield, Michael Ap, Das, Ravi K., Morgan, Celia Ja, Nutt, David J., and Curran, H. Valerie. Acute and chronic effects of cannabinoids on effort-related decision-making and reward learning: an evaluation of the cannabis 'amotivational' hypotheses. Psychopharmacology 2016. 233 (19-20) 3537-3552.

Leocani, L., Nuara, A., Houdayer, E., Schiavetti, I., Del, Carro U., Amadio, S., Straffi, L., Rossi, P., Martinelli, V., Vila, C., Sormani, M. P., and Comi, G. Sativex and clinical-neurophysiological measures of spasticity in progressive multiple sclerosis. Journal of Neurology 2015. 262 (11) 2520-2527.

Leocani, Letizia, Nuara, Arturo, Houdayer, Elise, Schiavetti, Irene, Del Carro, Ubaldo, Amadio, Stefano, Straffi, Laura, Rossi, Paolo, Martinelli, Vittorio, Vila, Carlos, Sormani, Maria Pia, and Comi, Giancarlo. Sativex() and clinical-neurophysiological measures of spasticity in progressive multiple sclerosis. Journal of Neurology 2015. 262 (11) 2520-2527.

Levin, David Neville, Dulberg, Zachary, Chan, An Wen, Hare, Gregory M. T., Mazer, C. David, and Hong, Aaron. A randomized-controlled trial of nabilone for the prevention of acute postoperative nausea and vomiting in elective surgery. Canadian journal of anaesthesia = Journal canadien d'anesthesie 2017. 64 (4) 385-395.

Lichtman, Aron H., Lux, Eberhard Albert, McQuade, Robert, Rossetti, Sandro, Sanchez, Raymond, Sun, Wei, Wright, Stephen, Kornyeyeva, Elena, and Fallon, Marie T. Results of a Double-Blind, Randomized, Placebo-Controlled Study of Nabiximols Oromucosal Spray as an Adjunctive Therapy in Advanced Cancer Patients with Chronic Uncontrolled Pain. Journal of Pain and Symptom Management 2018. 55 (2) 179-188.

Linares, Ila M. P., Guimaraes, Francisco S., Eckeli, Alan, Crippa, Ana C. S., Zuardi, Antonio W., Souza, Jose D. S., Hallak, Jaime E., and Crippa, Jose A. S. No Acute Effects of Cannabidiol on the Sleep-Wake Cycle of Healthy Subjects: A Randomized, Double-Blind, Placebo-Controlled, Crossover Study. Frontiers in Pharmacology 2018. 9, 315.

Lintzeris, Nicholas, Bhardwaj, Anjali, Mills, Llewellyn, Dunlop, Adrian, Copeland, Jan, McGregor, Iain, Bruno, Raimondo, Gugusheff, Jessica, Phung, Nghi, Montebello, Mark, Chan, Therese, Kirby, Adrienne, Hall, Michelle, Jefferies, Meryem, Luksza, Jennifer, Shanahan, Marian, Kevin, Richard, Allsop, David, Agonist Replacement for Cannabis Dependence study, group. Nabiximols for the Treatment of Cannabis Dependence: A Randomized Clinical Trial. JAMA internal medicine 2019.

Lintzeris, Nicholas, Mills, Llewellyn, Dunlop, Adrian, Copeland, Jan, McGregor, Iain, Bruno, Raimondo, Kirby, Adrienne, Montebello, Mark, Hall, Michelle, Jefferies, Meryem, Kevin, Richard, Bhardwaj, Anjali, Agonist Replacement For Cannabis Dependence Study, Group. Cannabis use in patients 3 months after ceasing nabiximols for the treatment of cannabis dependence: Results from a placebo-controlled randomised trial. Drug and alcohol dependence 2020. 215, 108220.

Liu, Z., Galettis, P., Broyd, S. J., van Hell, H., Greenwood, L. M., de Krey, P., Steigler, A., Zhu, X., Schneider, J., Solowij, N., et al. Model-based analysis on systemic availability of coadministered cannabinoids after controlled vaporised administration. Internal medicine journal 2019.

Liu, Zheng, Galettis, Peter, Broyd, Samantha J., van Hell, Hendrika, Greenwood, Lisa-Marie, de Krey, Peter, Steigler, Amy, Zhu, Xiao, Schneider, Jennifer, Solowij, Nadia, Martin, Jennifer H. Model-based analysis on systemic availability of co-administered cannabinoids after controlled vaporised administration. Internal medicine journal 2020. 50 (7) 846-853.

Lopez, Hector L., Cesareo, Kyle R., Raub, Betsy, Kedia, A. William, Sandrock, Jennifer E., Kerksick, Chad M., Ziegenfuss, Tim N. Effects of Hemp Extract on Markers of Wellness, Stress Resilience, Recovery and Clinical Biomarkers of Safety in Overweight, But Otherwise Healthy Subjects. Journal of dietary supplements 2020. 17 (5) 561-586.

López-Sendón Moreno, J. L., García Caldentey, J., Trigo Cubillo, P., Ruiz Romero, C., García Ribas, G., Alonso Arias, M. A., García de Yébenes, M. J., Tolón, R. M., Galve-Roperh, I., and Sagredo, O. A double-blind, randomized, cross-over, placebo-controlled, pilot trial with Sativex in Huntington's disease. Journal of Neurology 2016. 263 (7) 1390‐1400.

Lopez-Sendon Moreno, Jose Luis, Garcia Caldentey, Juan, Trigo Cubillo, Patricia, Ruiz Romero, Carolina, Garcia Ribas, Guillermo, Alonso Arias, M. A. A., Garcia de Yebenes, Maria Jesus, Tolon, Rosa Maria, Galve-Roperh, Ismael, Sagredo, Onintza, Valdeolivas, Sara, Resel, Eva, Ortega-Gutierrez, Silvia, Garcia-Bermejo, Maria Laura, Fernandez Ruiz, Javier, Guzman, Manuel, and Garcia de Yebenes Prous, Justo. A double-blind, randomized, cross-over, placebo-controlled, pilot trial with Sativex in Huntington's disease. Journal of Neurology 2016. 263 (7) 1390-1400.

Lu, S., Cameron, K., Ganesan, S., Feldman, B., and McKenna, M. A double-blind placebo control pilot study on the safety and tolerability of Nabilone in marijuana users. Mental Health and Substance Use: Dual Diagnosis 2013. 6 (2) 133-139.

Lunn, S., Diaz, P., O'Hearn, S., Cahill, S. P., Blake, A., Narine, K., Dyck, J. R. B. Human Pharmacokinetic Parameters of Orally Administered Î”9-Tetrahydrocannabinol Capsules Are Altered by Fed Versus Fasted Conditions and Sex Differences. Cannabis and cannabinoid research 2019. 4 (4) 255‐264.

Lunn, Stephanie, Diaz, Patrick, O'Hearn, Shannon, Cahill, Shaina P., Blake, Alexia, Narine, Kelly, Dyck, Jason R. B. Human Pharmacokinetic Parameters of Orally Administered DELTA9-Tetrahydrocannabinol Capsules Are Altered by Fed Versus Fasted Conditions and Sex Differences. Cannabis and cannabinoid research 2019. 4 (4) 255-264.

Lynch, Mary E., Cesar-Rittenberg, Paula, and Hohmann, Andrea G. A double-blind, placebo-controlled, crossover pilot trial with extension using an oral mucosal cannabinoid extract for treatment of chemotherapy-induced neuropathic pain. Journal of Pain and Symptom Management 2014. 47 (1) 166-173.

Maas, Andrew I. R., Murray, Gordon, Henney, Herbert, Kassem, Nadim, Legrand, Valerie, Mangelus, Miriam, Muizelaar, Jan Paul, Stocchetti, Nino, Knoller, Nachshon, and Pharmos, TBI investigators. Efficacy and safety of dexanabinol in severe traumatic brain injury: results of a phase III randomised, placebo-controlled, clinical trial. The Lancet.Neurology 2006. 5 (1) 38-45.

Mahlberg, R. and Walther, S. Actigraphy in agitated patients with dementia. Monitoring treatment outcomes. Zeitschrift fur Gerontologie und Geriatrie 2007. 40 (3) 178-184.

Malik, Z., Bayman, L., Valestin, J., Rizvi-Toner, A., Hashmi, S., and Schey, R. Dronabinol increases pain threshold in patients with functional chest pain: a pilot double-blind placebo-controlled trial. Diseases of the esophagus : official journal of the International Society for Diseases of the Esophagus 2017. 30 (2) 1-8.

Manini, Alex F., Yiannoulos, Georgia, Bergamaschi, Mateus M., Hernandez, Stephanie, Olmedo, Ruben, Barnes, Allan J., Winkel, Gary, Sinha, Rajita, Jutras-Aswad, Didier, Huestis, Marilyn A., and Hurd, Yasmin L. Safety and pharmacokinetics of oral cannabidiol when administered concomitantly with intravenous fentanyl in humans. Journal of addiction medicine 2015. 9 (3) 204-210.

Markovà, J., Essner, U., Akmaz, B., Marinelli, M., Trompke, C., Lentschat, A., and Vila, C. Sativex® as add-on therapy vs. further optimized first-line ANTispastics (SAVANT) in resistant multiple sclerosis spasticity: a double-blind, placebo-controlled randomised clinical trial. International Journal of Neuroscience 2019. 129 (2) 119‐128.

Markova, J. Sativex® as Add-on therapy Vs. further optimized first-line ANTispastics (SAVANT) in resistant multiple sclerosis spasticity double blind randomized clinical trial. Multiple Sclerosis Journal 2017. 23 (3) 990‐.

Markova, Jolana, Essner, Ute, Akmaz, Bulent, Marinelli, Marcella, Trompke, Christiane, Lentschat, Arnd, and Vila, Carlos. Sativex as add-on therapy vs. further optimized first-line ANTispastics (SAVANT) in resistant multiple sclerosis spasticity: a double-blind, placebo-controlled randomised clinical trial. The International journal of neuroscience 2019. 129 (2) 119-128.

Mason, Natasha L., Theunissen, Eef L., Hutten, Nadia R. P. W., Tse, Desmond H. Y., Toennes, Stefan W., Jansen, Jacobus F. A., Stiers, Peter, Ramaekers, Johannes G. Reduced responsiveness of the reward system is associated with tolerance to cannabis impairment in chronic users. Addiction biology 2021. 26 (1) e12870.

Mason, Natasha L., Theunissen, Eef L., Hutten, Nadia R. P. W., Tse, Desmond H. Y., Toennes, Stefan W., Stiers, Peter, and Ramaekers, Johannes G. Cannabis induced increase in striatal glutamate associated with loss of functional corticostriatal connectivity. European neuropsychopharmacology : the journal of the European College of Neuropsychopharmacology 2019. 29 (2) 247-256.

McGuire, Philip, Robson, Philip, Cubala, Wieslaw Jerzy, Vasile, Daniel, Morrison, Paul Dugald, Barron, Rachel, Taylor, Adam, and Wright, Stephen. Cannabidiol (CBD) as an Adjunctive Therapy in Schizophrenia: A Multicenter Randomized Controlled Trial. The American journal of psychiatry 2018. 175 (3) 225-231.

Meiri, Eyal, Jhangiani, Haresh, Vredenburgh, James J., Barbato, Luigi M., Carter, Frederick J., Yang, Hwa Ming, and Baranowski, Vickie. Efficacy of dronabinol alone and in combination with ondansetron versus ondansetron alone for delayed chemotherapy-induced nausea and vomiting. Current medical research and opinion 2007. 23 (3) 533-543.

Meneses-Gaya, Carolina de, Crippa, Jose A., Hallak, Jaime E., Miguel, Andre Q., Laranjeira, Ronaldo, Bressan, Rodrigo A., Zuardi, Antonio W., Lacerda, Acioly L. Cannabidiol for the treatment of crack-cocaine craving: an exploratory double-blind study. Revista brasileira de psiquiatria (Sao Paulo, Brazil : 1999) 2020.

Meuth, Sven G., Henze, Thomas, Essner, Ute, Trompke, Christiane, Vila Silvan, Carlos. Tetrahydrocannabinol and cannabidiol oromucosal spray in resistant multiple sclerosis spasticity: consistency of response across subgroups from the SAVANT randomized clinical trial. The International journal of neuroscience 2020. 130 (12) 1199-1205.

Morgan, Celia J. A., Freeman, Tom P., Hindocha, Chandni, Schafer, Grainne, Gardner, Chelsea, and Curran, H. Valerie. Individual and combined effects of acute delta-9-tetrahydrocannabinol and cannabidiol on psychotomimetic symptoms and memory function. Translational psychiatry 2018. 8 (1) 181.

Morrison, P. D. and Stone, J. M. Synthetic delta-9-tetrahydrocannabinol elicits schizophrenia-like negative symptoms which are distinct from sedation. Human psychopharmacology 2011. 26 (1) 77-80.

Muller-Vahl, K. R., Koblenz, A., Jobges, M., Kolbe, H., Emrich, H. M., and Schneider, U. Influence of treatment of Tourette syndrome with delta9-tetrahydrocannabinol (delta9-THC) on neuropsychological performance. Pharmacopsychiatry 2001. 34 (1) 19-24.

Muller-Vahl, K. R., Schneider, U., Koblenz, A., Jobges, M., Kolbe, H., Daldrup, T., and Emrich, H. M. Treatment of Tourette's syndrome with Delta 9-tetrahydrocannabinol (THC): a randomized crossover trial. Pharmacopsychiatry 2002. 35 (2) 57-61.

Muller-Vahl, K. R., Schneider, U., Prevedel, H., Theloe, K., Kolbe, H., Daldrup, T., and Emrich, H. M. DELTA9-tetrahydrocannabinol (THC) is effective in the treatment of tics in Tourette syndrome: A 6-week randomized trial. Journal of Clinical Psychiatry 2003. 64 (4) 459-465.

Muller-Vahl, Kirsten R., Prevedel, Heidrun, Theloe, Karen, Kolbe, Hans, Emrich, Hinderk M., and Schneider, Udo. Treatment of Tourette syndrome with delta-9-tetrahydrocannabinol (delta 9-THC): no influence on neuropsychological performance. Neuropsychopharmacology : official publication of the American College of Neuropsychopharmacology 2003. 28 (2) 384-388.

Muller-Vahl, Kirsten R., Schneider, Udo, Prevedel, Heidrun, Theloe, Karen, Kolbe, Hans, Daldrup, Thomas, and Emrich, Hinderk M. Delta 9-tetrahydrocannabinol (THC) is effective in the treatment of tics in Tourette syndrome: a 6-week randomized trial. The Journal of clinical psychiatry 2003. 64 (4) 459-465.

Naef, Myrtha, Curatolo, Michele, Petersen-Felix, Steen, Arendt-Nielsen, Lars, Zbinden, Alex, and Brenneisen, Rudolf. The analgesic effect of oral delta-9-tetrahydrocannabinol (THC), morphine, and a THC-morphine combination in healthy subjects under experimental pain conditions. Pain 2003. 105 (1-2) 79-88.

Naftali, Timna, Mechulam, Refael, Marii, Amir, Gabay, Gila, Stein, Asaf, Bronshtain, Miriam, Laish, Ido, Benjaminov, Fabiana, and Konikoff, Fred M. Low-Dose Cannabidiol Is Safe but Not Effective in the Treatment for Crohn's Disease, a Randomized Controlled Trial. Digestive diseases and sciences 2017. 62 (6) 1615-1620.

Narang, Sanjeet, Gibson, Daniel, Wasan, Ajay D., Ross, Edgar L., Michna, Edward, Nedeljkovic, Srdjan S., and Jamison, Robert N. Efficacy of dronabinol as an adjuvant treatment for chronic pain patients on opioid therapy. The journal of pain : official journal of the American Pain Society 2008. 9 (3) 254-264.

NCT, 01606202. A study of cannabis based medicine extracts and placebo in patients with pain due to spinal cord injury. Clinicaltrials.gov/ct2/results? Term= NCT 01606202&search=search 2012.

NCT00046722. Marijuana for HIV-Related Peripheral Neuropathy. Https://clinicaltrials.gov/show/nct00046722 2002.

NCT00123201. Study to Evaluate the Efficacy and Safety of Dronabinol Metered Dose Inhaler (MDI) in Acute Treatment of Migraine Headache. Https://clinicaltrials.gov/show/nct00123201 2005.

NCT00176163. Supporting Effect of Dronabinol on Behavioral Therapy in Fibromyalgia and Chronic Back Pain. Https://clinicaltrials.gov/show/nct00176163 2005.

NCT00202423. fMRI and Neurophysiological Study Protocol on Cannabinoids in Multiple Sclerosis. Clinicaltrials gov, national institutes of health [http://www clinicaltrials gov] 2005.

NCT00217971. Dronabinol Treatment for Marijuana Addiction. Https://clinicaltrials.gov/show/nct00217971 2005.

NCT00241579. Analgesic Efficacy of Smoked Cannabis. Https://clinicaltrials.gov/show/nct00241579 2005.

NCT00248378. Short-Term Effects of Medicinal Cannabis Therapy on Spasticity in Multiple Sclerosis. Clinicaltrials gov, national institutes of health [http://www clinicaltrials gov] 2001.

NCT00254761. Effects of Smoked Marijuana on Neuropathic Pain. Https://clinicaltrials.gov/show/nct00254761 2005.

NCT00255580. Medicinal Cannabis for Painful HIV Neuropathy. Https://clinicaltrials.gov/show/nct00255580 2005.

NCT00260741. Cannabis for Spasticity in Multiple Sclerosis. Https://clinicaltrials.gov/show/nct00260741 2005.

NCT00272207. A Trial Assessing the Effect of Nabilone on Pain and Quality of Life in Patients With Fibromyalgia. Https://clinicaltrials.gov/show/nct00272207 2006.

NCT00285051. Comparison of Delta-8-THC to Ondansetron in the Prevention of Acute Nausea From Moderately Emetogenic Chemotherapy. Https://clinicaltrials.gov/show/nct00285051 2006.

NCT00309413. A Clinical Trial on the Antipsychotic Properties of Cannabidiol. Https://clinicaltrials.gov/show/nct00309413 2006.

NCT00377468. Effect of Delta-9-Tetrahydrocannabinol on the Prevention of Chronic Pain in Patients With Acute CRPS (ETIC-Study). Https://clinicaltrials.gov/show/nct00377468 2006.

NCT00381199. Nabilone Versus Amitriptyline in Improving Quality of Sleep in Patients With Fibromyalgia. Https://clinicaltrials.gov/show/nct00381199 2006.

NCT00384410. Use of the Cannabinoid Nabilone for the Promotion of Sleep in Chronic, Non-Malignant Pain Patients. Https://clinicaltrials.gov/show/nct00384410 2006.

NCT00391079. Sativex Versus Placebo When Added to Existing Treatment for Central Neuropathic Pain in MS. Clinicaltrials gov, national institutes of health [http://www clinicaltrials gov] 2009.

NCT00397605. Cannabinoids in Bipolar Affective Disorder. Https://clinicaltrials.gov/show/nct00397605 2006.

NCT00418925. Efficacy of Dronabinol for the Treatment of Cervical Dystonia. Https://clinicaltrials.gov/show/nct00418925 2007.

NCT00480181. Efficacy and Safety Evaluation of Nabilone as Adjunctive Therapy to Gabapentin for the Management of Neuropathic Pain in Multiple Sclerosis. Clinicaltrials gov, national institutes of health [http://www clinicaltrials gov] 2007.

NCT00530764. A Study of Sativex® for Pain Relief in Patients With Advanced Malignancy. Https://clinicaltrials.gov/show/nct00530764 2007.

NCT00552604. MUltiple Sclerosis and Extract of Cannabis (MUSEC) Study. Clinicaltrials gov, national institutes of health [http://www clinicaltrials gov] 2010.

NCT00553059. Palonosetron and Dexamethasone With or Without Dronabinol in Preventing Nausea and Vomiting in Patients Receiving Chemotherapy For Cancer. Https://clinicaltrials.gov/show/nct00553059 2007.

NCT00588731. Cannabidiol Treatment of Cognitive Dysfunction in Schizophrenia. Https://clinicaltrials.gov/show/nct00588731 2007.

NCT00623376. Randomized Double Blind Cross Over Study for Nabilone in Spasticity in Spinal Cord Injury Persons. Https://clinicaltrials.gov/show/nct00623376 2008.

NCT00628290. Evaluation of the Antipsychotic Efficacy of Cannabidiol in Acute Schizophrenic Psychosis. Https://clinicaltrials.gov/show/nct00628290 2008.

NCT00642499. Dronabinol Versus Placebo in Treatment and Prevention of Highly Active Anti-Retroviral Therapy (HAART)-Related Nausea and Vomiting. Https://clinicaltrials.gov/show/nct00642499 2008.

NCT00674609. A Study of Sativex® for Pain Relief in Patients With Advanced Malignancy. Https://clinicaltrials.gov/show/nct00674609 2008.

NCT00678795. A Parallel Group Study to Compare SativexÂ® With Placebo in the Treatment of Detrusor Overactivity in Patients With MS. Clinicaltrials gov, national institutes of health [http://www clinicaltrials gov] 2008.

NCT00681538. A Study of the Safety and Effectiveness of SativexÂ®, for the Relief of Symptoms of Spasticity in Subjects With Multiple Sclerosis (MS). Clinicaltrials gov, national institutes of health [http://www clinicaltrials gov] 2010.

NCT00682929. Cannabis for Spasticity in Multiple Sclerosis: a Placebo-Controlled Study. Clinicaltrials gov, national institutes of health [http://www clinicaltrials gov] 2003.

NCT00697710. A Phase Ib/IIa, Double-Blind, Randomized Study to Assess the Safety, Tolerability, Pharmacokinetics, and Pharmacodynamics of S-777469 in Subjects With Atopic Dermatitis. Https://clinicaltrials.gov/show/nct00697710 2008.

NCT00699634. Nabilone for the Treatment of Phantom Limb Pain. Https://clinicaltrials.gov/show/nct00699634 2008.

NCT00710424. A Study of Sativex® for Pain Relief Due to Diabetic Neuropathy. Https://clinicaltrials.gov/show/nct00710424 2008.

NCT00711646. A Study of SativexÂ® for Relief of Spasticity in Subjects With Multiple Sclerosis. Clinicaltrials gov, national institutes of health [http://www clinicaltrials gov] 2008.

NCT00711880. A Study of Sativex® for Relief of Peripheral Neuropathic Pain Associated With Allodynia. Https://clinicaltrials.gov/show/nct00711880 2008.

NCT00713323. A Study to Compare the Safety and Tolerability of Sativex® in Patients With Neuropathic Pain. Https://clinicaltrials.gov/show/nct00713323 2008.

NCT00713817. A Study to Determine the Maintenance of Effect After Long-term Treatment of Sativex® in Subjects With Neuropathic Pain. Https://clinicaltrials.gov/show/nct00713817 2008.

NCT00723918. Combination of an Investigational Cannabinoid and Methadone for HIV-associated Neuropathy. Https://clinicaltrials.gov/show/nct00723918 2008.

NCT00781001. Efficacy of Inhaled Cannabis in Diabetic Painful Peripheral Neuropathy. Https://clinicaltrials.gov/show/nct00781001 2008.

NCT00842985. Dronabinol Interactions With Cognitive Enhancing Drug in Humans. Https://clinicaltrials.gov/show/nct00842985 2009.

NCT00959218. Efficacy and Safety of the Pain Relieving Effect of Dronabinol in Central Neuropathic Pain Related to Multiple Sclerosis. Clinicaltrials gov, national institutes of health [http://www clinicaltrials gov] 2009.

NCT00965809. Add on Study on Δ9-THC Treatment for Posttraumatic Stress Disorders (PTSD). Https://clinicaltrials.gov/show/nct00965809 2009.

NCT00982982. Effects of Delta-9-THC and Iomazenil in Healthy Humans. Https://clinicaltrials.gov/show/nct00982982 2009.

NCT01024335. Dronabinol Naltrexone Treatment for Opioid Dependence. Https://clinicaltrials.gov/show/nct01024335 2009.

NCT01025700. Nabilone & Marijuana Addiction. Https://clinicaltrials.gov/show/nct01025700 2009.

NCT01035281. Efficacy Study of Nabilone in the Treatment of Diabetic Peripheral Neuropathic Pain. Https://clinicaltrials.gov/show/nct01035281 2009.

NCT01037088. CCRC: the Analgesic Effect of Vaporized Cannabis on Neuropathic Pain. Clinicaltrials gov, national institutes of health [http://www clinicaltrials gov] 2009.

NCT01149018. Efficacy Trial of Oral Tetrahydrocannabinol in Patients With Fibromyalgia. Https://clinicaltrials.gov/show/nct01149018 2010.

NCT01180374. The Effects of Cannabidiol and ∆-9-THC in Humans. Https://clinicaltrials.gov/show/nct01180374 2010.

NCT01222468. Effect of Cannabinoids on Spasticity and Neuropathic Pain in Spinal Cord Injured Persons. Https://clinicaltrials.gov/show/nct01222468 2010.

NCT01262651. Sativex® for Relieving Persistent Pain in Participants With Advanced Cancer. Https://clinicaltrials.gov/show/nct01262651 2010.

NCT01318369. Efficacy Study of Δ9-THC to Treat Chronic Abdominal Pain. Https://clinicaltrials.gov/show/nct01318369 2011.

NCT01380457. Bioavailability Study of Dronabinol Capsules 10 mg Under Fasting Conditions. Https://clinicaltrials.gov/show/nct01380457 2011.

NCT01394185. Effects of Dronabinol (Oral THC) on Cannabis Use. Https://clinicaltrials.gov/show/nct01394185 2011.

NCT01517100. The Role of Endocannabinoids in Insulin Production and Action. Https://clinicaltrials.gov/show/nct01517100 2012.

NCT01538225. Neurophysiological Study of Sativex in Multiple Sclerosis (MS) Spasticity. Https://clinicaltrials.gov/show/nct01538225 2012.

NCT01551511. Δ9-THC (Namisol®) in Chronic Pancreatitis Patients Suffering From Persistent Abdominal Pain. Https://clinicaltrials.gov/show/nct01551511 2012.

NCT01555983. Vaporized Cannabis and Spinal Cord Injury Pain. Https://clinicaltrials.gov/show/nct01555983 2012.

NCT01591629. The Effects of ∆-9-THC and Naloxone in Humans. Https://clinicaltrials.gov/show/nct01591629 2012.

NCT01599234. A Study to Evaluate the Efficacy of Sativex in Relieving Symptoms of Spasticity Due to Multiple Sclerosis. Https://clinicaltrials.gov/show/nct01599234 2012.

NCT01604265. A Study of Sativex in the Treatment of Central Neuropathic Pain Due to Multiple Sclerosis. Https://clinicaltrials.gov/show/nct01604265 2012.

NCT01606176. A Study to Evaluate the Effects of Cannabis Based Medicine in Patients With Pain of Neurological Origin. Clinicaltrials.gov/ct2/results? Cond=&term=nct01606176&cntry1=&state1=&recrs= 2012.

NCT01606189. A Study to Compare Sublingual Cannabis Based Medicine Extracts With Placebo to Treat Brachial Plexus Injury Pain. Https://clinicaltrials.gov/show/nct01606189 2012.

NCT01606202. A Study of Cannabis Based Medicine Extracts and Placebo in Patients With Pain Due to Spinal Cord Injury. Https://clinicaltrials.gov/show/nct01606202 2012.

NCT01610687. A Long-term Safety Extension Study of Delta-9-tetrahydrocannabinol (THC) and Cannabidiol (CBD) in Multiple Sclerosis. Https://clinicaltrials.gov/show/nct01610687 2012.

NCT01610700 and Wade, D. Double Blind, Randomised, Parallel Group, Placebo Controlled Trial of a Combination of THC and CBD in Patients With Multiple Sclerosis, Followed by an Open Label Assessment and Study Extension. Http://clinicaltrials.gov/show/nct01610700 2001.

NCT01610713. An Study to Investigate the Efficacy of Delta-9-tetrahydrocannabinol (THC) and Cannabidiol (CBD) in Multiple Sclerosis. Https://clinicaltrials.gov/show/nct01610713 2012.

NCT01665573. Cannabinoid Augmentation of Fear Response in Humans. Https://clinicaltrials.gov/show/nct01665573 2012.

NCT01747850. Sativex and Behavioral-relapse Prevention Strategy in Cannabis Dependence. Https://clinicaltrials.gov/show/nct01747850 2012.

NCT01755091. Safety and Efficacy Study of Dronabinol to Treat Obstructive Sleep Apnea. Https://clinicaltrials.gov/show/nct01755091 2012.

NCT01771731. Vaporized Cannabis for Chronic Pain Associated With Sickle Cell Disease. Https://clinicaltrials.gov/show/nct01771731 2013.

NCT01790555. Perioperative Δ9-THC for Postsurgical Pain. Https://clinicaltrials.gov/show/nct01790555 2013.

NCT01812616. A Safety Study of Sativex Compared With Placebo (Both With Dose-intense Temozolomide) in Recurrent Glioblastoma Patients. Https://clinicaltrials.gov/show/nct01812616 2013.

NCT01826188. Combined THC and CBD Drops for Treatment of Crohn's Disease. Https://clinicaltrials.gov/show/nct01826188 2013.

NCT01868048. Phase 3, 28-week, Randomized, Double-blind, Placebo-controlled Safety and Efficacy Study of Nabiximols as an add-on Therapy in Subjects With Spasticity Due to Multiple Sclerosis. Https://clinicaltrials.gov/show/nct01868048 2013.

NCT01964547. A Randomized Study of Sativex on Cognitive Function and Mood: multiple Sclerosis Patients. Https://clinicaltrials.gov/show/nct01964547 2013.

NCT01969474. Phase 1 Study to Study the Efficacy and Safety of Cannabis in the Treatment of Tinnitus. Clinicaltrials.gov [www.clinicaltrials.gov] 2013.

NCT02088060. A Four-week Clinical Trial Investigating Efficacy and Safety of Cannabidiol as a Treatment for Acutely Ill Schizophrenic Patients. Https://clinicaltrials.gov/show/nct02088060 2014.

NCT02249299. Experimental Medicine in ADHD - Cannabinoids. Https://clinicaltrials.gov/show/nct02249299 2014.

NCT02283281. Anesthetic Premedication With a Cannabis Extract (Cannapremed). Https://clinicaltrials.gov/show/nct02283281 2014.

NCT02291536. Processing of Salient Emotional Stimuli as a Function of Tetrahydrocannabinol (THC) and Cannabidiol (CBD). Https://clinicaltrials.gov/show/nct02291536 2014.

NCT02400203. FREE Living Hulled HEMP Seed and Oil Trial. Https://clinicaltrials.gov/show/nct02400203 2015.

NCT02460692. Trial of Dronabinol and Vaporized Cannabis in Neuropathic Low Back Pain. Https://clinicaltrials.gov/show/nct02460692 2015.

NCT02504151. Cannabidiol Treatment in Patients With Early Psychosis. Https://clinicaltrials.gov/show/nct02504151 2015.

NCT02539823. Acute and Short-term Effects of CBD on Cue-induced Craving in Drug-abstinent Heroin-dependent Humans. Https://clinicaltrials.gov/show/nct02539823 2015.

NCT02544750. An Open-label Extension Trial of Cannabidiol (GWP42003-P, CBD) for Seizures in Tuberous Sclerosis Complex (GWPCARE6). Https://clinicaltrials.gov/show/nct02544750 2015.

NCT02544763. A Randomized Controlled Trial of Cannabidiol (GWP42003-P, CBD) for Seizures in Tuberous Sclerosis Complex (GWPCARE6). Https://clinicaltrials.gov/show/nct02544763 2015.

NCT02559167. Cannabidiol and Cocaine Craving/Dependence. Https://clinicaltrials.gov/show/nct02559167 2015.

NCT02560545. Cannabinoids Effects on the Pain Modulation System. Https://clinicaltrials.gov/show/nct02560545 2015.

NCT02564952. An Open-label Extension Trial to Investigate Possible Drug-drug Interactions Between Clobazam and Cannabidiol. Https://clinicaltrials.gov/show/nct02564952 2015.

NCT02565108. A Randomized Controlled Trial to Investigate Possible Drug-drug Interactions Between Clobazam and Cannabidiol. Https://clinicaltrials.gov/show/nct02565108 2015.

NCT02607891. A Study to Investigate Possible Drug-drug Interactions Between Stiripentol or Valproate and Cannabidiol in Patients With Epilepsy. Https://clinicaltrials.gov/show/nct02607891 2015.

NCT02607904. An Open-label Extension Trial to Investigate Possible Drug-drug Interactions Between Stiripentol or Valproate and Cannabidiol in Patients With Epilepsy. Https://clinicaltrials.gov/show/nct02607904 2015.

NCT02608931. The Safety, Tolerability and Efficacy of Dronabinol, for the Treatment of Nausea and Vomiting in Familial Dysautonomia. Https://clinicaltrials.gov/show/nct02608931 2015.

NCT02675842. Investigation of Cannabis for Pain and Inflammation in Lung Cancer. Https://clinicaltrials.gov/show/nct02675842 2016.

NCT02683018. Investigation of Cannabis for Chronic Pain and Palliative Care. Https://clinicaltrials.gov/show/nct02683018 2016.

NCT02728687. Topical Menthol +/- Mannitol for Painful Diabetic Peripheral Neuropathy. Https://clinicaltrials.gov/show/nct02728687 2016.

NCT02729623. The Pharmacokinetics, Safety, and Ease of Use of a Portable Metered-Dose Cannabis Inhaler. Https://clinicaltrials.gov/show/nct02729623 2016.

NCT02802540. Nabilone Effect on the Attenuation of Anorexia, Nutritional Status and Quality of Life in Lung Cancer Patients. Https://clinicaltrials.gov/show/nct02802540 2016.

NCT02811510. Gender Related Differences in the Acute Effects of Delta-9-Tetrahydrocannabinol in Healthy Humans: sub-Study I. Https://clinicaltrials.gov/show/nct02811510 2016.

NCT02849587. Using a Field Performance Test on an iPad to Evaluate Driving Under the Influence of Cannabis. Https://clinicaltrials.gov/show/nct02849587 2016.

NCT02911324. Cannabinoid Medication for Adults With OCD. Https://clinicaltrials.gov/show/nct02911324 2016.

NCT02926859. Enhancing Recovery in Early Schizophrenia. Https://clinicaltrials.gov/show/nct02926859 2016.

NCT02961309. A Laboratory Model of Increasing Delta-9-tetrahydrocannabinol (THC) Potency on Cigarette Smoking. Https://clinicaltrials.gov/show/nct02961309 2016.

NCT02976779. A Phase I, Double Blind, Randomized, Placebo Controlled, Maximal Dose Study to Determine the Safety, Tolerability of Topical Cream Containing MGC (Medical Grade Cannabis) in Healthy Volunteers. Https://clinicaltrials.gov/show/nct02976779 2016.

NCT03003260. The Effect of CanChew® Cannabidiol (CBD) Containing Chewing Gum on Irritable Bowel Syndrome. Https://clinicaltrials.gov/show/nct03003260 2016.

NCT03005119. Evaluation of the Safety, Tolerability, and Efficacy of Orally Administered PTL201 in MS Patients With Spasticity-related Symptoms. Https://clinicaltrials.gov/show/nct03005119 2016.

NCT03060993. CAN BREATHE in COPD Trial. Https://clinicaltrials.gov/show/nct03060993 2017.

NCT03087201. CANNAbinoids in the Treatment of TICS (CANNA-TICS). Https://clinicaltrials.gov/show/nct03087201 2017.

NCT03098940. A Bioavailability Study on Dronabinol. Https://clinicaltrials.gov/show/nct03098940 2017.

NCT03099005. Effect of Cannabis and Endocannabinoids on HIV Neuropathic Pain. Https://clinicaltrials.gov/show/nct03099005 2017.

NCT03102918. Cannabidiol Pharmacotherapy for Adults With Cannabis Use Disorder. Https://clinicaltrials.gov/show/nct03102918 2017.

NCT03172741. The Effects of Different Medical Marijuana Strains on Motor and Cognitive Function in People With Multiple Sclerosis. Https://clinicaltrials.gov/show/nct03172741 2017.

NCT03206463. Cognitive and Psychophysiological Effects of Delta-9-Tetrahydrocannabinol in Bipolar Disorder. Https://clinicaltrials.gov/show/nct03206463 2017.

NCT03245658. The Effect of Cannabis in Pancreatic Cancer. Https://clinicaltrials.gov/show/nct03245658 2017.

NCT03247244. Safety and Efficacy of Cannabis in Tourette Syndrome. Https://clinicaltrials.gov/show/nct03247244 2017.

NCT03310593. Cannabidiol as an Adjunctive Treatment for Bipolar Depression. Https://clinicaltrials.gov/show/nct03310593 2017.

NCT03337503. Safety and Efficacy of Medical Cannabis Oil in the Treatment of Patients With Chronic Pain. Https://clinicaltrials.gov/show/nct03337503 2017.

NCT03339622. Safety and Efficacy of Smoked Cannabis for Improving Quality of Life in Advanced Cancer Patients. Https://clinicaltrials.gov/show/nct03339622 2017.

NCT03398083. Evaluation of the Effects of Cannabidiol (CBD) Compared to Delta-9-Tetrahydrocannabinol (THC) and Alprazolam. Https://clinicaltrials.gov/show/nct03398083 2018.

NCT03422861. Nabilone Use For Acute Pain in Inflammatory Bowel Disease Patients. Https://clinicaltrials.gov/show/nct03422861 2018.

NCT03467620. Cannabidiol Usage as an Adjunct Therapy for Crohn's Disease. Https://clinicaltrials.gov/show/nct03467620 2018.

NCT03508895. Hemp Seed Protein Consumption for Hypertension. Https://clinicaltrials.gov/show/nct03508895 2018.

NCT03518801. Cannabidiol and Prolonged Exposure. Https://clinicaltrials.gov/show/nct03518801 2018.

NCT03530800. Dronabinol in Trichotillomania. Https://clinicaltrials.gov/show/nct03530800 2018.

NCT03549819. Cannabidiol for the Treatment of Anxiety Disorders: an 8-Week Pilot Study. Https://clinicaltrials.gov/show/nct03549819 2018.

NCT03550352. Cannabinoids in PLWHIV on Effective ART. Https://clinicaltrials.gov/show/nct03550352 2018.

NCT03564548. Inhaled Cannabis Versus Fentanyl Buccal Tablets for Management of Breakthrough Pain in Cancer Patients. Https://clinicaltrials.gov/show/nct03564548 2018.

NCT03609853. Behavioral Pharmacology of THC and D-limonene. Https://clinicaltrials.gov/show/nct03609853 2018.

NCT03635593. Cannabis Oil for Chronic Non-Cancer Pain Treatment. Https://clinicaltrials.gov/show/nct03635593 2018.

NCT03651726. A Study to Examine the Efficacy of a Therapeutic THX-110 for Tourette Syndrome. Https://clinicaltrials.gov/show/nct03651726 2018.

NCT03664141. Impact of Cannabis Oil on Nutrition in Hemodialysis Patients Study (ICON-HP Study). Https://clinicaltrials.gov/show/nct03664141 2018.

NCT03675971. COPE: cannabinoids to Obviate Pain Experiment After Knee Replacement. Https://clinicaltrials.gov/show/nct03675971 2018.

NCT03690791. Efficacy of Cannabinoids in Amyotrophic Lateral Sclerosis or Motor Neurone Disease. Https://clinicaltrials.gov/show/nct03690791 2018.

NCT03693833. CBS Treatment in Hand Osteoarthritis and Psoriatic Arthritis. Https://clinicaltrials.gov/show/nct03693833 2018.

NCT03744091. Evaluation of the Pharmacokinetics of Prana P1 Capsules. Https://clinicaltrials.gov/show/nct03744091 2018.

NCT03756974. BX-1 in Spasticity Due to Multiple Sclerosis. Https://clinicaltrials.gov/show/nct03756974 2018.

NCT03763851. Cannabis Oil and Radiation Therapy for the Management of Pain. Https://clinicaltrials.gov/show/nct03763851 2018.

NCT03769896. Nabilone for Non-motor Symptoms in Parkinson's Disease. Https://clinicaltrials.gov/show/nct03769896 2018.

NCT03805750. Trial of Cannabis for Essential Tremor. Https://clinicaltrials.gov/show/nct03805750 2019.

NCT03813095. Exploratory Dose Ranging Study Assessing APH-1501 for the Treatment of Opioid Addiction. Https://clinicaltrials.gov/show/nct03813095 2019.

NCT03825965. Cannabinoids vs. Placebo on Persistent Post-surgical Pain Following TKA: a Pilot RCT. Https://clinicaltrials.gov/show/nct03825965 2019.

NCT03928015. Evaluation of Dronabinol For Acute Pain Following Traumatic Injury. Https://clinicaltrials.gov/show/nct03928015 2019.

NCT03929835. Study to Investigate the Efficacy and Safety of Cannabis Oil for the Treatment of Subjects With Hidradenitis Suppurativa. Https://clinicaltrials.gov/show/nct03929835 2019.

NCT03941288. Efficacy and Safety of Cannabidiol for Gastroparesis and Functional Dyspepsia. Https://clinicaltrials.gov/show/nct03941288 2019.

Nitecka-Buchta, Aleksandra, Nowak-Wachol, Anna, Wachol, Kacper, Walczynska-Dragon, Karolina, Olczyk, Pawel, Batoryna, Olgierd, Kempa, Wojciech, Baron, Stefan. Myorelaxant Effect of Transdermal Cannabidiol Application in Patients with TMD: A Randomized, Double-Blind Trial. Journal of clinical medicine 2019. 8 (11).

Novotna, A., Mares, J., Ratcliffe, S., Novakova, I., Vachova, M., Zapletalova, O., Gasperini, C., Pozzilli, C., Cefaro, L., Comi, G., Rossi, P., Ambler, Z., Stelmasiak, Z., Erdmann, A., Montalban, X., Klimek, A., Davies, P., and Sativex Spasticity Study Group. A randomized, double-blind, placebo-controlled, parallel-group, enriched-design study of nabiximols* (Sativex() ), as add-on therapy, in subjects with refractory spasticity caused by multiple sclerosis. European journal of neurology 2011. 18 (9) 1122-1131.

Noyes, R., Jr., Brunk, S. F., Baram, D. A., and Canter, A. Analgesic effect of delta-9-tetrahydrocannabinol. J Clin Pharmacol 1975. 15 (2-3) 139-143.

NTR1787. The Role of the Endocannabinoid System in Psychiatric Disorders and Symptoms: a Pharmacological fMRI study. Http://www.who.int/trialsearch/trial2.aspx? Trialid=ntr1787 2009.

NTR52. The effect of Marinol (tetra-9-hydrocannabinol) on the frequency of TLESRs. Http://www.who.int/trialsearch/trial2.aspx? Trialid=ntr52 2005.

Nurmikko, Turo J., Serpell, Mick G., Hoggart, Barbara, Toomey, Peter J., Morlion, Bart J., and Haines, Derek. Sativex successfully treats neuropathic pain characterised by allodynia: a randomised, double-blind, placebo-controlled clinical trial. Pain 2007. 133 (1-3) 210-220.

O'Neill, Aisling, Wilson, Robin, Blest-Hopley, Grace, Annibale, Luciano, Colizzi, Marco, Brammer, Mick, Giampietro, Vincent, Bhattacharyya, Sagnik. Normalization of mediotemporal and prefrontal activity, and mediotemporal-striatal connectivity, may underlie antipsychotic effects of cannabidiol in psychosis. Psychological medicine 2021. 51 (4) 596-606.

Parikh, Neha, Kramer, William G., Khurana, Varun, Cognata Smith, Christina, and Vetticaden, Santosh. Bioavailability study of dronabinol oral solution versus dronabinol capsules in healthy volunteers. Clinical pharmacology : advances and applications 2016. 8, 155-162.

Peters, Erica N., Herrmann, Evan S., Smith, Carson, Wilhelm, Jess Alan, Koszowski, Bartosz, Halquist, Matthew, Kosmider, Leon, Poklis, Justin, Roth, Sage, Bart, Stephan, Pickworth, Wallace B. Impact of smoked cannabis on tobacco cigarette smoking intensity and subjective effects: A placebo-controlled, double-blind, within-subjects human laboratory study. Experimental and clinical psychopharmacology 2020.

Pini, Luigi Alberto, Guerzoni, Simona, Cainazzo, Maria Michela, Ferrari, Anna, Sarchielli, Paola, Tiraferri, Ilaria, Ciccarese, Michela, and Zappaterra, Maurizio. Nabilone for the treatment of medication overuse headache: results of a preliminary double-blind, active-controlled, randomized trial. The journal of headache and pain 2012. 13 (8) 677-684.

Pooyania, Sepideh, Ethans, Karen, Szturm, Tony, Casey, Alan, and Perry, Daryl. A randomized, double-blinded, crossover pilot study assessing the effect of nabilone on spasticity in persons with spinal cord injury. Archives of physical medicine and rehabilitation 2010. 91 (5) 703-707.

Portenoy, Russell K., Ganae-Motan, Elena Doina, Allende, Silvia, Yanagihara, Ronald, Shaiova, Lauren, Weinstein, Sharon, McQuade, Robert, Wright, Stephen, and Fallon, Marie T. Nabiximols for opioid-treated cancer patients with poorly-controlled chronic pain: a randomized, placebo-controlled, graded-dose trial. The journal of pain : official journal of the American Pain Society 2012. 13 (5) 438-449.

Pretzsch, C. M., Freyberg, J., Voinescu, B., Lythgoe, D., Horder, J., Mendez, M. A., Wichers, R., Ajram, L., Ivin, G., Heasman, M., et al. Effects of cannabidiol on brain excitation and inhibition systems; a randomised placebo-controlled single dose trial during magnetic resonance spectroscopy in adults with and without autism spectrum disorder. Neuropsychopharmacology 2019. 44 (8) 1398‐1405.

Pretzsch, Charlotte M., Voinescu, Bogdan, Lythgoe, David, Horder, Jamie, Mendez, Maria Andreina, Wichers, Robert, Ajram, Laura, Ivin, Glynis, Heasman, Martin, Edden, Richard A. E., Williams, Steven, Murphy, Declan G. M., Daly, Eileen, McAlonan, Grainne M. Effects of cannabidivarin (CBDV) on brain excitation and inhibition systems in adults with and without Autism Spectrum Disorder (ASD): a single dose trial during magnetic resonance spectroscopy. Translational psychiatry 2019. 9 (1) 313.

Pretzsch, Charlotte Marie, Freyberg, Jan, Voinescu, Bogdan, Lythgoe, David, Horder, Jamie, Mendez, Maria Andreina, Wichers, Robert, Ajram, Laura, Ivin, Glynis, Heasman, Martin, Edden, Richard A. E., Williams, Steven, Murphy, Declan G. M., Daly, Eileen, and McAlonan, Grainne M. Effects of cannabidiol on brain excitation and inhibition systems; a randomised placebo-controlled single dose trial during magnetic resonance spectroscopy in adults with and without autism spectrum disorder. Neuropsychopharmacology : official publication of the American College of Neuropsychopharmacology 2019.

R Noyes, SF Brunk, DA Avery, AC Canter. The analgesic properties of delta-9-tetrahydrocannabinol and codeine. Clinical pharmacology and therapeutics 1975. 18 (1).

Rabinak, Christine A., Peters, Craig, Marusak, Hilary A., Ghosh, Samiran, and Phan, K. Luan. Effects of acute DELTA9-tetrahydrocannabinol on next-day extinction recall is mediated by post-extinction resting-state brain dynamics. Neuropharmacology 2018. 143, 289-298.

Ramaekers, J. G., Kauert, G., Theunissen, E. L., Toennes, S. W., and Moeller, M. R. Neurocognitive performance during acute THC intoxication in heavy and occasional cannabis users. Journal of psychopharmacology (Oxford, England) 2009. 23 (3) 266-277.

Ranganathan, Mohini, De Aquino, Joao P., Cortes-Briones, Jose A., Radhakrishnan, Rajiv, Pittman, Brian, Bhakta, Savita, D'Souza, Deepak C. Highs and lows of cannabinoid-dopamine interactions: effects of genetic variability and pharmacological modulation of catechol-O-methyl transferase on the acute response to delta-9-tetrahydrocannabinol in humans. Psychopharmacology 2019. 236 (11) 3209-3219.

Ranganathan, Mohini, Radhakrishnan, Rajiv, Addy, Peter H., Schnakenberg-Martin, Ashley M., Williams, Ashley H., Carbuto, Michelle, Elander, Jacqueline, Pittman, Brian, Andrew Sewell, R., Skosnik, Patrick D., and D'Souza, Deepak Cyril. Tetrahydrocannabinol (THC) impairs encoding but not retrieval of verbal information. Progress in neuro-psychopharmacology & biological psychiatry 2017. 79 (Pt B) 176-183.

Reichenbach, Zachary Wilmer, Sloan, Joshua, Rizvi-Toner, Amna, Bayman, Levent, Valestin, Jessica, and Schey, Ron. A 4-week pilot study with the cannabinoid receptor agonist dronabinol and its effect on metabolic parameters in a randomized trial. Clinical Therapeutics 2015. 37 (10) 2267-2274.

Riggs, Patricia K., Vaida, Florin, Rossi, Steven S., Sorkin, Linda S., Gouaux, Ben, Grant, Igor, and Ellis, Ronald J. A pilot study of the effects of cannabis on appetite hormones in HIV-infected adult men. Brain research 2012. 1431, 46-52.

Riva, Nilo, Mora, Gabriele, Soraru, Gianni, Lunetta, Christian, Ferraro, Ottavia E., Falzone, Yuri, Leocani, Letizia, Fazio, Raffaella, Comola, Mauro, Comi, Giancarlo, and CANALS Study Group. Safety and efficacy of nabiximols on spasticity symptoms in patients with motor neuron disease (CANALS): a multicentre, double-blind, randomised, placebo-controlled, phase 2 trial. The Lancet.Neurology 2019. 18 (2) 155-164.

Rog, David J., Nurmikko, Turo J., Friede, Tim, and Young, Carolyn A. Randomized, controlled trial of cannabis-based medicine in central pain in multiple sclerosis. Neurology 2005. 65 (6) 812-819.

Schimrigk, Sebastian, Marziniak, Martin, Neubauer, Christine, Kugler, Eva Maria, Werner, Gudrun, and Abramov-Sommariva, Dimitri. Dronabinol Is a Safe Long-Term Treatment Option for Neuropathic Pain Patients. European Neurology 2017. 78 (5-6) 320-329.

Schindler, Emmanuelle A. D., Schnakenberg Martin, Ashley M., Sewell, R. Andrew, Ranganathan, Mohini, DeForest, Anna, Pittman, Brian P., Perrino, Albert, Jr., D'Souza, Deepak C. In an exploratory randomized, double-blind, placebo-controlled, cross-over study, psychoactive doses of intravenous delta-9-tetrahydrocannabinol fail to produce antinociceptive effects in healthy human volunteers. Psychopharmacology 2020. 237 (10) 3097-3107.

Schoedel, Kerri A., Szeto, Isabella, Setnik, Beatrice, Sellers, Edward M., Levy-Cooperman, Naama, Mills, Catherine, Etges, Tilden, and Sommerville, Kenneth. Abuse potential assessment of cannabidiol (CBD) in recreational polydrug users: A randomized, double-blind, controlled trial. Epilepsy & behavior : E&B 2018. 88, 162-171.

Serpell, M., Ratcliffe, S., Hovorka, J., Schofield, M., Taylor, L., Lauder, H., and Ehler, E. A double-blind, randomized, placebo-controlled, parallel group study of THC/CBD spray in peripheral neuropathic pain treatment. European journal of pain (London, England) 2014. 18 (7) 999-1012.

Sexton, B. F., Tunbridge, R. J., Brook Carter, N., Jackson, P. G., Wright, K., Stark, M. M., and Englehart, K. The influence of cannabis on driving. TRL report 2000.

Skrabek, Ryan Quinlan, Galimova, Lena, Ethans, Karen, and Perry, Daryl. Nabilone for the treatment of pain in fibromyalgia. The journal of pain : official journal of the American Pain Society 2008. 9 (2) 164-173.

Solowij, Nadia, Broyd, Samantha, Greenwood, Lisa Marie, van Hell, Hendrika, Martelozzo, Dave, Rueb, Kuna, Todd, Juanita, Liu, Zheng, Galettis, Peter, Martin, Jennifer, Murray, Robin, Jones, Alison, Michie, Patricia T., and Croft, Rodney. A randomised controlled trial of vaporised DELTA9-tetrahydrocannabinol and cannabidiol alone and in combination in frequent and infrequent cannabis users: acute intoxication effects. European Archives of Psychiatry and Clinical Neuroscience 2019. 269 (1) 17-35.

Spiera, Robert, Hummers, Laura, Chung, Lorinda, Frech, Tracy M., Domsic, Robyn, Hsu, Vivien, Furst, Daniel E., Gordon, Jessica, Mayes, Maureen, Simms, Robert, Lafyatis, Robert, Martyanov, Viktor, Wood, Tammara, Whitfield, Michael L., Constantine, Scott, Lee, Elizabeth, Dgetluck, Nancy, White, Barbara. Safety and Efficacy of Lenabasum in a Phase II, Randomized, Placebo-Controlled Trial in Adults With Systemic Sclerosis. Arthritis & rheumatology (Hoboken, N.J.) 2020. 72 (8) 1350-1360.

Spindle, Tory R., Cone, Edward J., Schlienz, Nicolas J., Bigelow, George E., Vandrey, Ryan, Mitchell, John M., Flegel, Ronald, Hayes, Eugene. Acute Effects of Smoked and Vaporized Cannabis in Healthy Adults Who Infrequently Use Cannabis: A Crossover Trial. JAMA network open 2018. 1 (7) e184841.

Sugarman, Dawn E., Poling, James, and Sofuoglu, Mehmet. The safety of modafinil in combination with oral 9-tetrahydrocannabinol in humans. Pharmacology, biochemistry, and behavior 2011. 98 (1) 94-100.

Sultan, Salahaden R., O'Sullivan, Saoirse E., England, Timothy J. The effects of acute and sustained cannabidiol dosing for seven days on the haemodynamics in healthy men: A randomised controlled trial. British journal of clinical pharmacology 2020. 86 (6) 1125-1138.

Svendsen, Kristina B., Jensen, Troels S., and Bach, Flemming W. Does the cannabinoid dronabinol reduce central pain in multiple sclerosis? Randomised double blind placebo controlled crossover trial. BMJ (Clinical research ed.) 2004. 329 (7460) 253.

Thiele, Elizabeth A., Marsh, Eric D., French, Jacqueline A., Mazurkiewicz-Beldzinska, Maria, Benbadis, Selim R., Joshi, Charuta, Lyons, Paul D., Taylor, Adam, Roberts, Claire, Sommerville, Kenneth, and Study Group. Cannabidiol in patients with seizures associated with Lennox-Gastaut syndrome (GWPCARE4): a randomised, double-blind, placebo-controlled phase 3 trial. Lancet (London, England) 2018. 391 (10125) 1085-1096.

Toth, Cory, Mawani, Shefina, Brady, Shauna, Chan, Cynthia, Liu, CaiXia, Mehina, Essie, Garven, Alexandra, Bestard, Jennifer, and Korngut, Lawrence. An enriched-enrolment, randomized withdrawal, flexible-dose, double-blind, placebo-controlled, parallel assignment efficacy study of nabilone as adjuvant in the treatment of diabetic peripheral neuropathic pain. Pain 2012. 153 (10) 2073-2082.

Turcotte, Dana, Doupe, Malcolm, Torabi, Mahmoud, Gomori, Andrew, Ethans, Karen, Esfahani, Farid, Galloway, Katie, and Namaka, Mike. Nabilone as an adjunctive to gabapentin for multiple sclerosis-induced neuropathic pain: a randomized controlled trial. Pain medicine (Malden, Mass.) 2015. 16 (1) 149-159.

van Amerongen, G., Kanhai, K., Baakman, A. C., Heuberger, J., Klaassen, E., Beumer, T. L., Strijers, R. L. M., Killestein, J., van Gerven, J., Cohen, A., et al. Effects on Spasticity and Neuropathic Pain of an Oral Formulation of Δ9-tetrahydrocannabinol in Patients WithProgressive Multiple Sclerosis. Clinical therapeutics 2018. 40 (9) 1467‐1482.

van Amerongen, Guido, Kanhai, Kawita, Baakman, Anne Catrien, Heuberger, Jules, Klaassen, Erica, Beumer, Tim L., Strijers, Rob L. M., Killestein, Joep, van Gerven, Joop, Cohen, Adam, and Groeneveld, Geert Jan. Effects on Spasticity and Neuropathic Pain of an Oral Formulation of DELTA9-tetrahydrocannabinol in Patients WithProgressive Multiple Sclerosis. Clinical Therapeutics 2018. 40 (9) 1467-1482.

van de Donk, Tine, Niesters, Marieke, Kowal, Mikael A., Olofsen, Erik, Dahan, Albert, and van Velzen, Monique. An experimental randomized study on the analgesic effects of pharmaceutical-grade cannabis in chronic pain patients with fibromyalgia. Pain 2019. 160 (4) 860-869.

van, Amerongen G., Kanhai, K., Baakman, A. C., Heuberger, J., Klaassen, E., Beumer, T. L., Strijers, R. L. M., Killestein, J., van, Gerven J., Cohen, A., and Groeneveld, G. J. Effects on Spasticity and Neuropathic Pain of an Oral Formulation of DELTA9-tetrahydrocannabinol in Patients With Progressive Multiple Sclerosis. Clinical Therapeutics 2018. 40 (9) 1467-1482.

Vaney, C., Heinzel-Gutenbrunner, M., Jobin, P., Tschopp, F., Gattlen, B., Hagen, U., Schnelle, M., and Reif, M. Efficacy, safety and tolerability of an orally administered cannabis extract in the treatment of spasticity in patients with multiple sclerosis: a randomized, double-blind, placebo-controlled, crossover study. Multiple sclerosis (Houndmills, Basingstoke, England) 2004. 10 (4) 417-424.

Wade, Derick T., Makela, Petra, Robson, Philip, House, Heather, and Bateman, Cynthia. Do cannabis-based medicinal extracts have general or specific effects on symptoms in multiple sclerosis? A double-blind, randomized, placebo-controlled study on 160 patients. Multiple sclerosis (Houndmills, Basingstoke, England) 2004. 10 (4) 434-441.

Wade, Derick T., Robson, Philip, House, Heather, Makela, Petra, and Aram, Julia. A preliminary controlled study to determine whether whole-plant cannabis extracts can improve intractable neurogenic symptoms. Clinical rehabilitation 2003. 17 (1) 21-29.

Wall, Matthew B., Pope, Rebecca, Freeman, Tom P., Kowalczyk, Oliwia S., Demetriou, Lysia, Mokrysz, Claire, Hindocha, Chandni, Lawn, Will, Bloomfield, Michael Ap, Freeman, Abigail M., Feilding, Amanda, Nutt, David J., and Curran, H. Valerie. Dissociable effects of cannabis with and without cannabidiol on the human brain's resting-state functional connectivity. Journal of psychopharmacology (Oxford, England) 2019., 269881119841568.

Wallace, Mark, Schulteis, Gery, Atkinson, J. Hampton, Wolfson, Tanya, Lazzaretto, Deborah, Bentley, Heather, Gouaux, Ben, and Abramson, Ian. Dose-dependent effects of smoked cannabis on capsaicin-induced pain and hyperalgesia in healthy volunteers. Anesthesiology 2007. 107 (5) 785-796.

Walter, C., Oertel, B., Felden, L., Nöth, U., Vermehren, J., Deichmann, R., Lötsch, J. Effects of oral Δ-tetrahydrocannabinol on the cerebral processing of olfactory input in healthy non-addicted subjects. European journal of clinical pharmacology 2017. 73 (12) 1579‐1587.

Walter, Carmen, Oertel, Bruno G., Felden, Lisa, Noth, Ulrike, Vermehren, Johannes, Deichmann, Ralf, and Lotsch, Jorn. Effects of oral DELTA9-tetrahydrocannabinol on the cerebral processing of olfactory input in healthy non-addicted subjects. European journal of clinical pharmacology 2017. 73 (12) 1579-1587.

Walter, Carmen, Oertel, Bruno G., Ludyga, Dagmar, Ultsch, Alfred, Hummel, Thomas, and Lotsch, Jorn. Effects of 20 mg oral DELTA(9) -tetrahydrocannabinol on the olfactory function of healthy volunteers. British Journal of Clinical Pharmacology 2014. 78 (5) 961-969.

Ware, Mark A., Fitzcharles, Mary Ann, Joseph, Lawrence, and Shir, Yoram. The effects of nabilone on sleep in fibromyalgia: results of a randomized controlled trial. Anesthesia and analgesia 2010. 110 (2) 604-610.

Ware, Mark A., Wang, Tongtong, Shapiro, Stan, Robinson, Ann, Ducruet, Thierry, Huynh, Thao, Gamsa, Ann, Bennett, Gary J., and Collet, Jean Paul. Smoked cannabis for chronic neuropathic pain: a randomized controlled trial. CMAJ : Canadian Medical Association journal = journal de l'Association medicale canadienne 2010. 182 (14) E694-E701.

Weber, M., Goldman, B., and Truniger, S. Tetrahydrocannabinol (THC) for cramps in amyotrophic lateral sclerosis: a randomised, double-blind crossover trial. Journal of neurology, neurosurgery, and psychiatry 2010. 81 (10) 1135-1140.

Weltens, Nathalie, Depoortere, Inge, Tack, Jan, and Van Oudenhove, Lukas. Effect of acute DELTA9-tetrahydrocannabinol administration on subjective and metabolic hormone responses to food stimuli and food intake in healthy humans: a randomized, placebo-controlled study. The American journal of clinical nutrition 2019. 109 (4) 1051-1063.

Weltens, Nathalie, Van Oudenhove, Lukas, Depoortere, Inge, Tack, Jan. Effect of acute delta9-tetrahydrocannabinol administration on subjective and metabolic hormone responses to food stimuli and food intake in healthy humans: A randomized, placebo-controlled study. American Journal of Clinical Nutrition 2019. 109 (4) 1051-1063.

Wesnes, K. A., Annas, P., Edgar, C. J., Deeprose, C., Karlsten, R., Philipp, A., Kalliomaki, J., and Segerdahl, M. Nabilone produces marked impairments to cognitive function and changes in subjective state in healthy volunteers. Journal of psychopharmacology (Oxford, England) 2010. 24 (11) 1659-1669.

Wilsey, Barth L., Deutsch, Reena, Samara, Emil, Marcotte, Thomas D., Barnes, Allan J., Huestis, Marilyn A., and Le, Danny. A preliminary evaluation of the relationship of cannabinoid blood concentrations with the analgesic response to vaporized cannabis. Journal of Pain Research 2016. 9, 587-598.

Wilsey, Barth, Marcotte, Thomas D., Deutsch, Reena, Zhao, Holly, Prasad, Hannah, and Phan, Amy. An Exploratory Human Laboratory Experiment Evaluating Vaporized Cannabis in the Treatment of Neuropathic Pain From Spinal Cord Injury and Disease. The journal of pain : official journal of the American Pain Society 2016. 17 (9) 982-1000.

Wilsey, Barth, Marcotte, Thomas, Deutsch, Reena, Gouaux, Ben, Sakai, Staci, and Donaghe, Haylee. Low-dose vaporized cannabis significantly improves neuropathic pain. The journal of pain : official journal of the American Pain Society 2013. 14 (2) 136-148.

Wilsey, Barth, Marcotte, Thomas, Tsodikov, Alexander, Millman, Jeanna, Bentley, Heather, Gouaux, Ben, and Fishman, Scott. A randomized, placebo-controlled, crossover trial of cannabis cigarettes in neuropathic pain. The journal of pain : official journal of the American Pain Society 2008. 9 (6) 506-521.

Wissel, Jorg, Haydn, Tanja, Muller, Jorg, Brenneis, Christian, Berger, Thomas, Poewe, Werner, and Schelosky, Ludwig D. Low dose treatment with the synthetic cannabinoid Nabilone significantly reduces spasticity-related pain : a double-blind placebo-controlled cross-over trial. Journal of Neurology 2006. 253 (10) 1337-1341.

Wong, B. S., Camilleri, M., Eckert, D., Carlson, P., Ryks, M., Burton, D., and Zinsmeister, A. R. Randomized pharmacodynamic and pharmacogenetic trial of dronabinol effects on colon transit in irritable bowel syndrome-diarrhea. Neurogastroenterology and motility : the official journal of the European Gastrointestinal Motility Society 2012. 24 (4) 358-e169.

Wong, Banny S., Camilleri, Michael, Busciglio, Irene, Carlson, Paula, Szarka, Lawrence A., Burton, Duane, and Zinsmeister, Alan R. Pharmacogenetic trial of a cannabinoid agonist shows reduced fasting colonic motility in patients with nonconstipated irritable bowel syndrome. Gastroenterology 2011. 141 (5) 1638-7.

Zajicek, J. P., Sanders, H. P., Wright, D. E., Vickery, P. J., Ingram, W. M., Reilly, S. M., Nunn, A. J., Teare, L. J., Fox, P. J., and Thompson, A. J. Cannabinoids in multiple sclerosis (CAMS) study: safety and efficacy data for 12 months follow up. Journal of neurology, neurosurgery, and psychiatry 2005. 76 (12) 1664-1669.

Zajicek, John Peter, Hobart, Jeremy C., Slade, Anita, Barnes, David, Mattison, Paul G., and MUSEC Research Group. Multiple sclerosis and extract of cannabis: results of the MUSEC trial. Journal of neurology, neurosurgery, and psychiatry 2012. 83 (11) 1125-1132.

Zajicek, John, Ball, Susan, Wright, David, Vickery, Jane, Nunn, Andrew, Miller, David, Cano, Mayam Gomez, McManus, David, Mallik, Sharukh, Hobart, Jeremy, and CUPID investigator group. Effect of dronabinol on progression in progressive multiple sclerosis (CUPID): a randomised, placebo-controlled trial. The Lancet.Neurology 2013. 12 (9) 857-865.

Zajicek, John, Fox, Patrick, Sanders, Hilary, Wright, David, Vickery, Jane, Nunn, Andrew, Thompson, Alan, and UK MS Research Group. Cannabinoids for treatment of spasticity and other symptoms related to multiple sclerosis (CAMS study): multicentre randomised placebo-controlled trial. Lancet (London, England) 2003. 362 (9395) 1517-1526.

Zaytseva, Y., Horáček, J., Hlinka, J., Fajnerová, I., Androvičová, R., Tintěra, J., Salvi, V., Balíková, M., Hložek, T., Španiel, F., et al. Cannabis-induced altered states of consciousness are associated with specific dynamic brain connectivity states. Journal of psychopharmacology (Oxford, England) 2019. 33 (7) 811‐821.

Zaytseva, Yuliya, Horacek, Jiri, Hlinka, Jaroslav, Fajnerova, Iveta, Androvicova, Renata, Tintera, Jaroslav, Salvi, Virginio, Balikova, Marie, Hlozek, Tomas, Spaniel, Filip, and Palenicek, Tomas. Cannabis-induced altered states of consciousness are associated with specific dynamic brain connectivity states. Journal of psychopharmacology (Oxford, England) 2019., 269881119849814.

No outcome of interest

Wallace, Mark S., Marcotte, Thomas D., Atkinson, J. H., Padovano, Hayley Treloar, Bonn-Miller, Marcel. A Secondary Analysis from a Randomized Trial on the Effect of Plasma Tetrahydrocannabinol Levels on Pain Reduction in Painful Diabetic Peripheral Neuropathy. The journal of pain 2020. 21 (11-12) 1175-1186.

Non-randomized studies

Not published in English or French

[Author not listed]. An impact of psychoactive substance abuse on a course and functional abilities in patients with schizophrenia. Ceska a slovenska psychiatrie 2019. 115 (1) 14‐19.

Altintas, Merih, Inanc, Leman, Hunca, Ayse Nazli, Ektiricioglu, Cihan, Yilmaz, Nihan, Tuna, Zehra Olcay, Uney, Ridvan. Theory of mind, aggression and impulsivity in patients with synthetic cannabinoid use disorders: A case-control study. Anadolu Psikiyatri Dergisi 2019. 20 (1) 5-12.

Bartolucci, Jorge, Nazzal, N., Verdugo, Fernando J., Prieto, Juan Carlos, Sepulveda, Pablo, and Corbalan, Ramon. [Characteristics, management, and outcomes of illicit drug consumers with acute myocardial infarction]. Revista medica de Chile 2016. 144 (1) 39-46.

Cesarini, Martin, Gonzalez Rojas, Natalia, Etcheverry, Jose Luis, Calderon, Jelipe F. Peralta, Diaz, Diego, Da Prat de Magalhaes, Gustavo A., Rojas, Galeno J., Parisi, Virginia L., Persi, Gabriel G., Gatto, Emilia M. Clinical registry of patients with movement disorders, reporting self-prescription of non standarized formulation of cannabis. Experience from a movement disorder center in Buenos Aires. Neurologia Argentina 2020.

Donath, Carolin, Pendergrass, Anna, Carolin, M. Sack, Weiss, Maren, Baier, Dirk. Prevalence and predictors for heavy episodic drinking-A cross-sectional representative study in Lower Saxony. Sucht 2019. 65 (5) 297-311.

Goldberg, E., Lakamper, S. Cannabidiol in the context of first-time road traffic medical fitness testing in Switzerland: Retrospective study on the significance and influence on the assessment outcome. Rechtsmedizin 2020.

Iliev, Y. and Akabaliev, V. Acute poisoning with dependency causing psychoactive substances: Gender differences in some socio-demographic and disease characteristics. General Medicine 2002. 4 (2) 32-36.

Iliev, Y. and Akabaliev, V. Acute poisonings with psychoactive substances - Sociodemographic characteristics and trends. Bulgarian Medicine 2002. 10 (4) 22-26.

Jakab, Laszlo, Szanto, Zalan, Benko, Istvan, Szalai, Zsuzsanna, Poto, Laszlo, and Molnar, F. Tamas. [Ethiopathogenesis of spontaneous primary pneumothorax. Marijuana: cause or a blame?]. Magyar sebeszet 2012. 65 (6) 421-425.

Jonsson, Arnar Jan, Birgisdottir, Hera, and Sigurdsson, Engilbert. [Does the use of cannabis increase the risk for psychosis and the development of schizophrenia?]. Laeknabladid 2014. 100 (9) 443-451.

Konstantinova-Larsen, S. V., Normann, P. T., Arnestad, M., Karinen, R., Christophersen, A. S., and Morland, J. Surveillance of abused drugs in forensic autopsy cases in Norway. Norsk Epidemiologi 2011. 21 (1) 43-48.

Lorente Fernandez, L., Monte Boquet, E., Perez-Miralles, F., Gil Gomez, I., Escutia Roig, M., Bosca Blasco, I., Poveda Andres, J. L., and Casanova-Estruch, B. Clinical experiences with cannabinoids in spasticity management in multiple sclerosis. Neurologia (Barcelona, Spain) 2014. 29 (5) 257-260.

Makkos, Zoltan, Fejes, Lilla, Inczedy-Farkas, Gabriella, Kassai-Farkas, Akos, Faludi, Gabor, and Lazary, Judit. [Clinical characteristics of cannabis-induced schizophrenia spectrum disorder]. Neuropsychopharmacologia Hungarica : a Magyar Pszichofarmakologiai Egyesulet lapja = official journal of the Hungarian Association of Psychopharmacology 2011. 13 (3) 127-138.

Medjedovic, A., Lebbink, R. W., Ramcharan, A. The effects of medicinal cannabis oil on migraine attacks. Pharmaceutisch Weekblad 2020. 155 (39) 23-31.

Moll Tuduri, Concepcion, Salgado Garcia, Emilio Jose, Santana Moreno, Daniel, Escoda Turon, Ona, Escoda Turon, Rosa, Nogue Xarau, Santiago. Emergency department visits for transient global amnesia alone or in association with substance abuse. Amnesia global transitoria y consumo de toxicos que motivan la consulta al servicio de urgencias. 2019. 31 (5) 341-345.

Muller, Kai W., Koch, Andreas, Beutel, Manfred E., Dickenhorst, Ulrike, Medenwaldt, Jens, and Wolfling, Klaus. [Internet addiction as a co-morbid disorder among patients of german addiction rehabilitation facilities: an exploratory investigation of clinical prevalence]. Psychiatrische Praxis 2012. 39 (6) 286-292.

Rentero Martin, David, Arias, Francisco, Sanchez-Romero, Sergio, Rubio, Gabriel, Rodriguez-Jimenez, Roberto. Cannabis-induced psychosis: clinical characteristics and its differentiation from schizophrenia with and without cannabis use. Adicciones 2020., 1251.

Simsek, Hamdi, Altintoprak, Ayse Ender. Research on the facial emotion identification-discrimination and communication skills in synthetic cannabinoid users. Anadolu Psikiyatri Dergisi 2019. 21 (Special Issue 1) 42-45.

Tomassini, Annarita, Roncone, Rita, Verni, Laura, Ortenzi, Roberta, Di Melchiorre, Giuditta, Tosone, Adele, Casacchia, Massimo, and Pollice, Rocco. [Use of cannabis and psychopathological risk in onset psychosis]. Rivista di psichiatria 2012. 47 (2) 170-177.

Turon, Rosa Escoda, Tuduri, Concepcion Moll, Garcia, Emilio Jose Salgado, Turon, Ona Escoda, Xarau, Santiago Nogue, Moreno, Daniel Santana. Emergency department visits for transient global amnesia alone or in association with substance abuse. Emergencias 2019. 31 (5) 341-345.

van der Meer, Sanchez Z. and Nappo, S. A. Progression on drug use and its intervening factors among crack users. Revista de saude publica 2002. 36 (4) 420-430.

Vermeulen, Jentien, Van De Kraats, Gerben. THC content in cannabis and the risk of psychosis: A patient control study. Tijdschrift voor Psychiatrie 2019. 61 (10) 733.

Weeda, M. R., Peters, B. D., de Haan, L., and Linszen, D. H. [Residual neuropsychological, structural and functional brain abnormalities after long-term cannabis use]. Tijdschrift voor psychiatrie 2006. 48 (3) 185-193.

Wendelmuth, Christoph, Wirz, Stefan, Torontali, Misel, Gastmeier, Anne, Gastmeier, Knud. [Dronabinol in geriatric pain and palliative care patients : A retrospective evaluation of statutory-health-insurance-covered outpatient medical treatment]. Dronabinol bei geriatrischen Schmerz- und Palliativpatienten : Eine retrospektive Auswertung der ambulanten kassenarztlichen Therapie. 2019. 33 (5) 384-391.

Wendelmuth, Christoph, Wirz, Stefan, Torontali, Misel, Gastmeier, Anne, Gastmeier, Knud. [Erratum to: Dronabinol in geriatric pain and palliative care patients : A retrospective evaluation of statutory-health-insurance-covered outpatient medical treatment]. Erratum zu: Dronabinol bei geriatrischen Schmerz- und Palliativpatienten : Eine retrospektive Auswertung der ambulanten kassenarztlichen Therapie. 2019. 33 (6) 562.

Conference abstract, letter, commentary, or other non-relevant design

[Author not listed]. Cannabis herb may have advantages over THC in epilepsy. Pharmaceutical Journal 2003. 271 (7258) 76.

[Author not listed]. Cannabis use can cause lasting memory problems. Pharmaceutical Journal 2002. 268 (7190) 391.

[Author not listed]. Dronabinol can't replace medical marijuana. Managed care (Langhorne, Pa.) 2005. 14 (8) 58.

[Author not listed]. Drug-induced male infertility. Prescrire International 2007. 16 (87) 22.

[Author not listed]. Marijuana use possibly linked to perio disease. Journal of the California Dental Association 2008. 36 (10) 725-729.

[Author not listed]. Marijuana use up. Medicine Today 2001. 2 (8) 7.

[Author not listed]. More on cannabis and psychosis. Medicine Today 2005. 6 (1) 10-11.

[Author not listed]. NICE to look at evidence for using cannabis for multiple sclerosis. Pharmaceutical Journal 2002. 268 (7199) 708.

Abi-Jaoude, Elia, Chen, Lei, Cheung, Patrina, Bhikram, Tracy, and Sandor, Paul. Preliminary Evidence on Cannabis Effectiveness and Tolerability for Adults With Tourette Syndrome. The Journal of neuropsychiatry and clinical neurosciences 2017. 29 (4) 391-400.

Actrn. Medicinal cannabis use among dementia patients. http://www.who.int/trialsearch/Trial2.aspx?TrialID=ACTRN12619000474156 2019.

Actrn. Pilot Study: effect of electrical brain stimulation on mental skills of individuals with long-term cannabis use. http://www.who.int/trialsearch/Trial2.aspx?TrialID=ACTRN12619000462189 2019.

Ali, Atif, Bin Asad, Muhammad Hassham Hassan, Akhtar, Naveed, Khan, Hira, Ahmad, Zia. The improvement on the skin surface by a new type of dermocosmetic loaded plant extract: A split face skin topographic study. Pakistan Journal of Pharmaceutical Sciences 2020. 33 (2) 531-535.

Andonian, D. O., Seaman, S. R., and Josephson, E. B. Profound hypotension and bradycardia in the setting of synthetic cannabinoid intoxication - A case series. American Journal of Emergency Medicine 2017. 35 (6) 940.

Anonymous. Association between cannabis use and complications related to ulcerative colitis in hospitalized patients: A propensity matched retrospective cohort study: Erratum. Medicine 2019. 98 (35) e17046.

Arfken, Cynthia L., Owens, Darlene, Madeja, Cheryl, and DeAngelis, Christina. Exploratory comparative study on the diffusion of synthetic cannabinoids and synthetic cathinones. Journal of Psychoactive Drugs 2014. 46 (5) 362-368.

Backberg, M., Tworek, L., Beck, O., and Helander, A. Analytically Confirmed Intoxications Involving MDMB-CHMICA from the STRIDA Project. Journal of Medical Toxicology 2017. 13 (1) 52-60.

Barnes, J. New findings in pharmacognosy: Myrrh, cannabis and feverfew. Pharmaceutical Journal 2000. 265 (7114) 427-428.

Barnett, James R., Grinspoon, Reid A., Harisinghani, Mukesh, Caruso, Paul A., Thiele, Elizabeth A. The efficacy of cannabidiol on renal angiomyolipoma and subependymal giant cell tumor volume in tuberous sclerosis complex. Journal of clinical neuroscience : official journal of the Neurosurgical Society of Australasia 2020. 77, 85-88.

Bateman, C. Get pragmatic about pot. South African Medical Journal 2000. 90 (8) 752-754.

Battah, A. Q. H., Al-Hadidi, M. S., Abdullat, E. M., and Hadidi, K. A. Alcohol and psychoactive drugs in road traffic fatalities within northern district of Amman. Jordan Medical Journal 2013. 47 (1) 66-72.

Berkowitz, Eugene A., Henry, Travis S., Veeraraghavan, Srihari, Staton, Gerald W. J., and Gal, Anthony A. Pulmonary effects of synthetic marijuana: chest radiography and CT findings. AJR.American journal of roentgenology 2015. 204 (4) 750-757.

Bhattacharyya, S., Atakan, Z., Martin-Santos, R., Crippa, J. A., Kambeitz, J., Prata, D., Williams, S., Brammer, M., Collier, D. A., and McGuire, P. K. Genetic moderators of sensitivity to the symptomatic and neural effects of Cannabis on psychosis. Schizophrenia bulletin. 2013. 39, S148‐S149.

Bichu, Shrirang, Tilve, Parag, Dhakate, Tushar, Kakde, Pranit, Bhasin, Nikhil, Jawandhiya, Pankaj, Dixit, Abhishek, Jain, Pranesh, Billa, Viswanath, Kirpalani, Ashok, Amin, Pravin. Catheter Related Right Atrial Thrombus in Patients on Maintenance Hemodialysis: Results of a Single Centre Retrospective Study from a Tertiary Care Hospital. The Journal of the Association of Physicians of India 2018. 66 (12) 31-34.

Blazejewski, S., Girodet, P.-O., Orriols, L., Capelli, A., and Moore, N. Factors associated with serious traffic crashes: A prospective study in Southwest France. Archives of internal medicine 2012. 172 (13) 1039-1041.

Bobitt, J., Qualls, S. H., Schuchman, M., Wickersham, R., Lum, H. D., Arora, K., Milavetz, G., and Kaskie, B. Qualitative Analysis of Cannabis Use Among Older Adults in Colorado. Drugs and Aging 2019.

Boyd, J., Reyes, M., and Clark, A. J. Use of a synthetic cannabinoid in the in-patient with chronic non-cancer pain. European journal of pain (London, England) 2009. 13, S124‐.

Brakatselos, Charalampos, Delis, Foteini, Asprogerakas, Michail-Zois, Lekkas, Panagiotis, Tseti, Ioulia, Tzimas, Petros S., Petrakis, Eleftherios A., Halabalaki, Maria, Skaltsounis, Leandros A., Antoniou, Katerina. Cannabidiol Modulates the Motor Profile and NMDA Receptor-related Alterations Induced by Ketamine. Neuroscience 2021. 454, 105-115.

Brooks, D. J., Aharonovich, E., and Levin, F. R. Cognitive impairment and treatment retention among cannabis dependent patients. Proceedings of the 70th annual scientific meeting of the college on problems of drug dependence; 2008 june 14-19; san juan, puerto rico, USA 2008., 23.

Cadman, P. E. Hypophosphatemia in Users of Cannabis. American Journal of Kidney Diseases 2017. 69 (1) 152-155.

Carr, Connie, Vertelney, Haley, Fronk, Joshua, and Trieu, Sandy. Dronabinol for the Treatment of Paraneoplastic Night Sweats in Cancer Patients: A Report of Five Cases. Journal of palliative medicine 2019.

Chagas, M. H., Eckeli, A. L., Zuardi, A. W., Pena-Pereira, M. A., Sobreira-Neto, M. A., Sobreira, E. T., Camilo, M. R., Bergamaschi, M. M., Schenck, C. H., Hallak, J. E., Tumas, V., Crippa, J. A. Cannabidiol can improve complex sleep-related behaviours associated with rapid eye movement sleep behaviour disorder in Parkinson's disease patients: a case series. J Clin Pharm Ther 2014. 39 (5) 564-6.

Chen, Yushiuan, Askenazi, Michele, Albanese, Bernadette A., Marx, Grace E. Syndromic Surveillance of Emergency Department Visits for Acute Adverse Effects of Marijuana, Tri-County Health Department, Colorado, 2016-2017. Public Health Reports 2019. 134 (2) 132-140.

Chocron, Yaniv, Vaucher, Julien, Zuber, Jean-Philippe. Cannabinoid hyperemesis syndrome. The BMJ 2019. 366, l4336.

Chung, Matthew, Kim, Hee Kee, Abdi, Salahadin. Update on cannabis and cannabinoids for cancer pain. Current opinion in anaesthesiology 2020. 33 (6) 825-831.

Cruccu, Giorgio, Di Stefano, Giulia, Truini, Andrea, Marchettini, Paolo. Micronized palmitoylethanolamide: A post hoc analysis of a controlled study in patients with low back pain - Sciatica. CNS and Neurological Disorders - Drug Targets 2019. 18 (6) 491-495.

Cunetti, L., Manzo, L., Peyraube, R., Arnaiz, J., Curi, L., and Orihuela, S. Chronic Pain Treatment With Cannabidiol in Kidney Transplant Patients in Uruguay. Transplantation proceedings 2018. 50 (2) 461-464.

Curtis, Nickel J. Medical marijuana for urologic chronic pelvic pain. Canadian Urological Association Journal 2018. 12 (6S3 Supplement 3) S181-S183.

Curtis, Susanna A., Lew, Dana, Spodick, Jonathan, Hendrickson, Jeanne E., Minniti, Caterina P., Roberts, John D. Medical marijuana certification for patients with sickle cell disease: a report of a single center experience. Blood advances 2020. 4 (16) 3814-3821.

Degenhardt, Louisa, Hall, Wayne, and Lynskey, Michael. Testing hypotheses about the relationship between cannabis use and psychosis. Drug and Alcohol Dependence 2003. 71 (1) 37-48.

Dervaux, Alain, Goldberger, Celine, Gourion, David, Bourdel, Marie Chantal, Laqueille, Xavier, Loo, Henri, Olie, Jean Pierre, and Krebs, Marie Odile. Impulsivity and sensation seeking in cannabis abusing patients with schizophrenia. Schizophrenia Research 2010. 123 (2-3) 278-280.

Desbois, Anne Claire and Cacoub, Patrice. Cannabis-associated arterial disease. Annals of Vascular Surgery 2013. 27 (7) 996-1005.

Dos Santos, R. G., Guimaraes, F. S., Crippa, J. A. S., Hallak, J. E. C., Rossi, G. N., Rocha, J. M., Zuardi, A. W. Serious adverse effects of cannabidiol (CBD): a review of randomized controlled trials. Expert opinion on drug metabolism & toxicology 2020.

Doss, Manoj K., Weafer, Jessica, Gallo, David A., de Wit, Harriet. DELTA9-Tetrahydrocannabinol During Encoding Impairs Perceptual Details yet Spares Context Effects on Episodic Memory. Biological psychiatry. Cognitive neuroscience and neuroimaging 2020. 5 (1) 110-118.

Duong, Heather M. Assessing the bidirectional relationship of posttraumatic stress disorder and substance use disorders. Dissertation Abstracts International: Section B: The Sciences and Engineering 2020. 81 (2-B) No-Specified.

Eisenberg, Elon, Ogintz, Miri, and Almog, Shlomo. The pharmacokinetics, efficacy, safety, and ease of use of a novel portable metered-dose cannabis inhaler in patients with chronic neuropathic pain: a phase 1a study. Journal of pain & palliative care pharmacotherapy 2014. 28 (3) 216-225.

Elliott, Luther, Golub, Andrew, Bennett, Alexander, and Guarino, Honoria. PTSD and cannabis-related coping among recent veterans in New York City. Contemporary Drug Problems: An Interdisciplinary Quarterly 2015. 42 (1) 60-76.

Falkowski, Carol. Why we need to be cautious about medical marijuana. Reefer sadness. Minnesota medicine 2014. 97 (4) 39-41.

Frankel, J. P., Hughes, A., Lees, A. J., and Stern, G. M. Marijuana for parkinsonian tremor. Journal of neurology, neurosurgery, and psychiatry 1990. 53 (5) 436.

Garakani, A., Welch, A. K., Jaffe, R. J., Protin, C. A., and McDowell, D. M. Psychosis and Low Cyanocobalamin in a Patient Abusing Nitrous Oxide and Cannabis. Psychosomatics 2014. 55 (6) 715-719.

Ghelani, Amar. Motives for Recreational Cannabis Use among Mental Health Professionals. Journal of Substance Use 2020.

Gonzalez-Wilhelm, Leonardo. Prevalence of alcohol and illicit drugs in blood specimens from drivers involved in traffic law offenses. Systematic review of cross-sectional studies. Traffic Injury Prevention 2007. 8 (2) 189-198.

Goyal, Sharad, Kubendran, Sindhu, Kogan, Mikhail, Rao, Yuan J. High expectations: The landscape of clinical trials of medical marijuana in oncology. Complementary therapies in medicine 2020. 49, 102336.

Gupta, Jeetendra Kumar. Bipolar disorder: A major setback in substance abuse. Indian Journal of Forensic Medicine and Toxicology 2020. 14 (3) 844-848.

Gupta, N., McDonald, M. A., Ross, H. J., Kobashigawa, J. A. Cannabis Use and Heart Transplantation: A Canadian Perspective. The Journal of heart and lung transplantation : the official publication of the International Society for Heart Transplantation 2020. 39 (4) S263-S264.

Hagerty, Sarah L., YorkWilliams, Sophie L., Sabbineni, Amithrupa, Bryan, Angela D., Hutchison, Kent E., Bidwell, L. Cinnamon, Weiland, Barbara J., Blaine, Sara K. DRD2 methylation is associated with executive control network connectivity and severity of alcohol problems among a sample of polysubstance users. Addiction Biology 2020. 25 (1) e12684.

Haleem, Reham, Wright, Robert. A Scoping Review on Clinical Trials of Pain Reduction With Cannabis Administration in Adults. Journal of clinical medicine research 2020. 12 (6) 344-351.

Hall, W. Dissecting the causal anatomy of the link between cannabis and other illicit drugs. Addiction 2006. 101 (4) 472-473.

Hall, W. Is cannabis use psychotogenic?. The Lancet 2006. 367 (9506) 193-195.

Hallstone, Michael. An exploratory investigation of marijuana and other drug careers. Journal of Psychoactive Drugs 2006. 38 (1) 65-75.

Harvey, David G., II. Epidemiology of marijuana use and mental health in the context of changing policies. Dissertation Abstracts International: Section B: The Sciences and Engineering 2020. 81 (12-B) No-Specified.

Huckabay, Loucine, Rannalli, Debby, Fisher, Dennis G., Reynolds, Grace L., Erlyana, Erlyana. Gender differences in risk taking behaviors for Chlamydia trachomatis. Health care for women international 2020. 41 (10) 1147-1165.

Iszaj, Fruzsina, Ehmann, Bea, Griffiths, Mark D., and Demetrovics, Zsolt. A Qualitative Study on the Effects of Psychoactive Substance use upon Artistic Creativity. Substance use & misuse 2018. 53 (8) 1275-1280.

Iversen, Leslie. Long-term effects of exposure to cannabis. Current Opinion in Pharmacology 2005. 5 (1) 69-72.

Jay, Therese M., Krebs, Marie-Odile, Kebir, Oussama. Exposure to cannabinoids can lead to persistent cognitive and psychiatric disorders. European Journal of Pain (United Kingdom) 2019. 23 (7) 1225-1233.

Jones, Jefferson M., Shults, Ruth A., Robinson, Byron, Komatsu, Kenneth K., Sauber-Schatz, Erin K. Marijuana and alcohol use among injured drivers evaluated at level I trauma centers in Arizona, 2008-2014. Drug and alcohol dependence 2019. 204, 107539.

Karila, L. and Benyamina, A. Psychiatric and addictologic complications of new synthetic products. La Revue du praticien 2018. 68 (6) 676-679.

Kepple, Nancy J. and Freisthler, Bridget. Exploring the ecological association between crime and medical marijuana dispensaries. Journal of studies on alcohol and drugs 2012. 73 (4) 523-530.

Khadanga, S. and Ades, P. A. What do we tell patients with coronary artery disease about marijuana use?. Coronary Artery Disease 2018. 29 (1) 1-3.

Kicman, Aleksandra, Toczek, Marek. The effects of cannabidiol, a non-intoxicating compound of cannabis, on the cardiovascular system in health and disease. International Journal of Molecular Sciences 2020. 21 (18) 1-45.

Klipsch, E. C., Hathaway, T., Mangus, R. S. Effect of Donor Death by Drug Intoxication Combined with Chronic Drug Abuse on Lung Transplantation: A Single Center Study. The Journal of heart and lung transplantation : the official publication of the International Society for Heart Transplantation 2020. 39 (4) S142.

Kolla, B. P., Mansukhani, M. P., Olson, E. J., St.Louis, E. K., Silber, M. H., and Morgenthaler, T. I. Medical Cannabis for Obstructive Sleep Apnea: Premature and Potentially Harmful. Mayo Clinic proceedings 2018. 93 (6) 689-692.

Krediet, Erwin, Janssen, Debbie G. A., Egberts, Toine C. G., Heerdink, Eibert R., Vermetten, Eric. Experiences with medical cannabis in the treatment of veterans with PTSD: Results from a focus group discussion. European Neuropsychopharmacology 2020. 36, 244-254.

Kuchenbuch, Mathieu, D'Onofrio, Gianluca, Chemaly, Nicole, Barcia, Giulia, Teng, Theo, Nabbout, Rima. Add-on cannabidiol significantly decreases seizures in 3 patients with SYNGAP1 developmental and epileptic encephalopathy. Epilepsia open 2020. 5 (3) 496-500.

Labay, L. M., Caruso, J. L., Gilson, T. P., Phipps, R. J., Knight, L. D., Lemos, N. P., McIntyre, I. M., Stoppacher, R., Tormos, L. M., Weins, A. L., Williams, E., and Logan, B. K. Synthetic cannabinoid drug use as a cause or contributory cause of death. Forensic Science International 2016. 260, 31-39.

Lafferty, Christopher K., Britt, Jonathan P. Cannabis Exposure Enhances Subcortical Control of Nucleus Accumbens Activity. Biological Psychiatry 2020. 87 (7) 592-594.

Lamy, Francois R., Daniulaityte, Raminta, Nahhas, Ramzi W., Barratt, Monica J., Smith, Alan G., Sheth, Amit, Martins, Silvia S., Boyer, Edward W., and Carlson, Robert G. Increases in synthetic cannabinoids-related harms: Results from a longitudinal web-based content analysis. The International journal on drug policy 2017. 44, 121-129.

Laqueille, Xavier, Ghodhbane, Souheil, Nacef, Fethi, Choubani, Zied, Nehdi, Mohamed, Douki, Saida, and Dervaux, Alain. Tobacco, alcohol and cannabis use in Tunisian patients with schizophrenia. Schizophrenia Research 2008. 98 (1-3) 327-328.

Lawn, Will, Hill, James, Hindocha, Chandni, Yim, Jocelyn, Yamamori, Yumeya, Jones, Gus, Walker, Hannah, Green, Sebastian F., Wall, Matthew B., Howes, Oliver D., Curran, H. Valerie, Freeman, Tom P., Bloomfield, Michael Ap. The acute effects of cannabidiol on the neural correlates of reward anticipation and feedback in healthy volunteers. Journal of psychopharmacology (Oxford, England) 2020. 34 (9) 969-980.

Lee, C. and Moll, S. Migratory superficial thrombophlebitis in a cannabis smoker. Circulation 2014. 130 (2) 214-215.

Leehey, M., Liu, Y., Epstein, C., Hart, F., Bainbridge, J., Cook, M., Sillau, S., Baud, Z., and Newman, H. Open label study of cannabidiol in Parkinson's disease. Movement Disorders 2017. 32, 913‐.

Lemelin, Annie, Godbout, Ariane, Pare, Guy, Bernard, Sophie. Improved Glycemic Control Through the Use of a Telehomecare Program in Patients with Diabetes Treated with Insulin. Diabetes technology & therapeutics 2020. 22 (4) 243-248.

Lemos, Nikolas P., San Nicolas, Alexander C., Volk, Justin A., Ingle, Eric A., and Williams, Chinyere M. Driving Under the Influence of Marijuana Versus Driving and Dying Under the Influence of Marijuana: A Comparison of Blood Concentrations of DELTA9-Tetrahydrocannabinol, 11-Hydroxy-DELTA9-Tetrahydrocannabinol, 11-Nor-9-Carboxy-DELTA9-Tetrahydrocannabinol and Other Cannabinoids in Arrested Drivers Versus Deceased Drivers. Journal of analytical toxicology 2015. 39 (8) 588-601.

Levinsohn, Erik A., Hill, Kevin P. Clinical uses of cannabis and cannabinoids in the United States. Journal of the Neurological Sciences 2020. 411, 116717.

Likar, Rudolf, Koestenberger, Markus, Stultschnig, Martin, Nahler, Gerhard. Concomitant treatment of malignant brain tumours with CBD - A case series and review of the literature. Anticancer Research 2019. 39 (10) 5797-5801.

Lissoni, P., Porro, G., Messina, G., Porta, E., Rovelli, F., Roselli, M. G., and Brivio, F. Morphine, melatonin, Marijuana, Magnolia and MYRRH as the "five m" schedule in the treatment of cancer pain and the possible dose-dependency of the antitumor and analgesic effects of the pineal hormone melatonin. Anticancer research 2014. 34 (10) 6033-6034.

Lopez-Valero, I., Torres, S., Salazar-Roa, M., Garcia-Taboada, E., Hernandez-Tiedra, S., Guzman, M., Sepulveda, J. M., Velasco, G., and Lorente, M. Optimization of a preclinical therapy of cannabinoids in combination with temozolomide against glioma. Biochemical pharmacology 2018. 157, 275-284.

Lotsch, J., Walter, C., Noth, U., Deichmann, R., and Oertel, B. G. Delta-9-tetrahydrocannabinol impedes pain memory. A pharmacological fMRI study in humans. Naunyn-schmiedeberg's archives of pharmacology. 2013. 386, S48.

Maharajan, Mari Kannan, Yong, Yu Jing, Yip, Hong Yang, Woon, Sze Shee, Yeap, Kar Mon, Yap, Khai Yeng, Yip, Shuen Chi, Yap, Kai Xian. Medical cannabis for chronic pain: can it make a difference in pain management?. Journal of Anesthesia 2019.

Maida, Vincent, Shi, Runjie B., Fazzari, Francesco G. T., Zomparelli, Lydia. Topical cannabis-based medicines - A novel paradigm and treatment for non-uremic calciphylaxis leg ulcers: An open label trial. International wound journal 2020. 17 (5) 1508-1516.

Malbergier, Andre, Amaral, Ricardo, de Andrade, Arthur Guerra, de Oliveira, Hercilio Pereira, Ometto, Mariella, Nicastri, Sergio, Cunha, Paulo Jannuzzi, Dib Goncalves, Priscila, dos Santos, Bernardo. Erratum to "Distinct effects of cocaine and cocaine + cannabis on neurocognitive functioning and abstinence: A six-month follow-up study" [Drug Alcohol Depend. 205 (2019) 107642](S0376871619304193)(10.1016/j.drugalcdep.2019.107642). Drug and Alcohol Dependence 2020. 208, 107860.

Marcellin, Fabienne, Miailhes, Patrick, Santos, Melina, Mercie, Patrick, Di Beo, Vincent, Salmon-Ceron, Dominique, Barre, Tangui, Wittkop, Linda, Protopopescu, Camelia, Zucman, David, Sogni, Philippe, Carrieri, Patrizia, Group, Anrs Co Hepavih Study. Cannabis Use and Plasma Human Immunodeficiency Virus (HIV) RNA Levels in Patients Coinfected With HIV and Hepatitis C Virus Receiving Antiretroviral Therapy: Data From the ANRS CO13 HEPAVIH Cohort. Clinical infectious diseases : an official publication of the Infectious Diseases Society of America 2020. 71 (9) 2536-2538.

Marcellin, Fabienne, Santos, Melina, Di Beo, Vincent, Barre, Tangui, Protopopescu, Camelia, Carrieri, Patrizia, Miailhes, Patrick, Mercie, Patrick, Salmon-Ceron, Dominique, Wittkop, Linda, Zucman, David, Sogni, Philippe. Cannabis use and plasma HIV RNA levels in HIV-HCV co-infected patients receiving antiretroviral therapy: data from the ANRS CO13 HEPAVIH cohort. Clinical infectious diseases : an official publication of the Infectious Diseases Society of America 2020.

Martin, H., Bursztejn, A. C., Albuisson, E., Leguern, A., Mahe, E., Villemur, B., Blaise, S., Perceau, G., Goujon, E., Lok, C., Modiano, P., Debure, C., Guillot, B., Maillard, H., Say, M., Carvalho-Lallement, P., Dompmartin, A., Journet-Tollhupp, J., Schmutz, J. L., Senet, P., Schoeffler, A., au nom du groupe d'Angiodermatologie de la, S. F. D. [Characteristics of chronic wounds in substance abuse: A retrospective study of 58 patients]. Caracteristiques des plaies chroniques chez les toxicomanes : etude retrospective de 58 patients. 2019. 146 (12) 793-800.

Mason, N. L., Theunissen, E. L., Hutten, Nrpw, Tse, D. H. Y., Toennes, S. W., Jansen, J. F. A., Stiers, P., Ramaekers, J. G. Reduced responsiveness of the reward system is associated with tolerance to cannabis impairment in chronic users. Addiction biology 2019.

McCallum, R. W. Medical options for the treatment of gastroparesis-related GERD. Gastroenterology and Hepatology 2009. 5 (10) 9-11.

Mikuriya, T. H. Cannabis as a substitute for alcohol: A harm-reduction approach. Journal of Cannabis Therapeutics 2004. 4 (1) 79-93.

Montagnese, Federica, Stahl, Kristina, Wenninger, Stephan, Schoser, Benedikt. A role for cannabinoids in the treatment of myotonia? Report of compassionate use in a small cohort of patients. Journal of neurology 2020. 267 (2) 415-421.

Moons, Philip. Propensity weighting: how to minimise comparative bias in non-randomised studies?. European journal of cardiovascular nursing : journal of the Working Group on Cardiovascular Nursing of the European Society of Cardiology 2020. 19 (1) 83-88.

Moore, Y. and Robinson, R. Cannabidiol reduced frequency of convulsive seizures in drug resistant Dravet syndrome. Archives of Disease in Childhood: Education and Practice Edition 2018. 103 (5) 278-279.

Moores, G., Lockey, A., and Attar, A. High times” Prevalence and perceptions of marijuana use among patients with epilepsy. Neurology 2018. 90 (15).

Morland, J., Bramness, J. G. DELTA9-tetrahydrocannabinol (THC) is present in the body between smoking sessions in occasional non-daily cannabis users. Forensic science international 2020. 309, 110188.

Nau, J.-Y. Some truths concerning the consumption of cannabis (2). Medecine et Hygiene 2002. 60 (2383) 578.

Nau, J.-Y. Some truths concerning the consumption of cannabis (3). Medecine et Hygiene 2002. 60 (2384) 601.

Nct. Assessing the Effect of Flavor on ENDS Users' Experiences and Exposures. https://clinicaltrials.gov/show/NCT04192032 2019.

Nct. Responses to Marijuana-Related Cues Versus Neutral Cues in Adults Taking Tetrahydrocannabinol (THC) - 2. https://clinicaltrials.gov/show/NCT00218504 2005.

Nordentoft, M. and Hjorthoj, C. Cannabis use and risk of psychosis in later life. The Lancet 2007. 370 (9584) 293-294.

Olesen, J. A., Posselt, C. M., Poulsen, C. H., Nordentoft, M., and Hjorthoj, C. Cannabis use disorders may protect against certain disorders of the digestive organs in people with schizophrenia but not in healthy controls. Psychological Medicine 2019., 1-8.

Osborne, Amy J., Pearson, John F., Noble, Alexandra J., Gemmell, Neil J., Horwood, L. John, Boden, Joseph M., Benton, Miles C., Macartney-Coxson, Donia P., Kennedy, Martin A. Genome-wide DNA methylation analysis of heavy cannabis exposure in a New Zealand longitudinal cohort. Translational psychiatry 2020. 10 (1) 114.

Page, Stacey A. and Verhoef, Marja J. Medicinal marijuana use: experiences of people with multiple sclerosis. Canadian family physician Medecin de famille canadien 2006. 52, 64-65.

Palamar, J. J., Acosta, P., Ompad, D. C., and Friedman, S. R. A Qualitative Investigation Comparing Psychosocial and Physical Sexual Experiences Related to Alcohol and Marijuana Use among Adults. Archives of sexual behavior 2018. 47 (3) 757-770.

Pane, Chiara, Sacca, Francesco. The use of medical grade cannabis in Italy for drug-resistant epilepsy: a case series. Neurological Sciences 2020. 41 (3) 695-698.

Patten, Scott B. Cannabis and non-psychotic mental disorders. Current opinion in psychology 2020. 38, 61-66.

Pedrozo-Pupo, John Carlos, Castaneda, Neddy Pamela, Campo-Arias, Adalberto. COPD secondary or associated with cannabis dependence. Respiratory Medicine Case Reports 2019. 28, 100902.

Pereira, P. F. and Simon, C. Substance psychoactive use in prison: A retrospective qualitative study. Psychotropes (Belgium) 2017. 23 (2) 35-56.

Pettit, C., Massick, S., and Bechtel, M. Cannabidiol-Induced Acute Generalized Exanthematous Pustulosis. Dermatitis 2018. 29 (6) 345-346.

Plange, Niklas, Arend, Kay O., Kaup, Marion, Doehmen, Benjamin, Adams, Herbert, Hendricks, Stefan, Cordes, Andreas, Huth, Julia, Sponsel, William E., and Remky, Andreas. Dronabinol and retinal hemodynamics in humans. American journal of ophthalmology 2007. 143 (1) 173-174.

Pope, Rebecca, Mokrysz, Claire, Hindocha, Chandni, Lawn, Will, Freeman, Abigail M., Curran, H. Valerie, Wall, Matthew B., Demetriou, Lysia, Nutt, David, Freeman, Tom P., Kowalczyk, Oliwia S., Bloomfield, Michael A. P., Feilding, Amanda. Dissociable effects of cannabis with and without cannabidiol on the human brain's resting-state functional connectivity. Journal of Psychopharmacology 2019. 33 (7) 822-830.

Pretzsch, Charlotte M., Voinescu, Bogdan, Mendez, Maria A., Wichers, Robert, Ajram, Laura, Ivin, Glynis, Heasman, Martin, Williams, Steven, Murphy, Declan Gm, Daly, Eileen, McAlonan, Grainne M. The effect of cannabidiol (CBD) on low-frequency activity and functional connectivity in the brain of adults with and without autism spectrum disorder (ASD). Journal of psychopharmacology (Oxford, England) 2019. 33 (9) 1141-1148.

Raes, E. and Verstraete, A.-G. Cannabis and driving: The situation in Europe. Annales pharmaceutiques francaises 2006. 64 (3) 197-203.

Reece, Albert Stuart, Hulse, Gary Kenneth. Canadian Cannabis Consumption and Patterns of Congenital Anomalies: An Ecological Geospatial Analysis. Journal of addiction medicine 2020.

Rehm, Jurgen, Manthey, Jakob. Cannabis and public health: a global experiment without control. World Psychiatry 2020. 19 (2) 192-194.

Reyes, M. Use of a synthetic cannabinoid in the in-patient with chronic non-cancer pain. Journal of Pain 2009. 10 (4) S37‐.

Richardson, S. J., Widmer, M., Zajicek, J., and Rule, S. A. Physiological doses of cannabinoids do not adversely affect MCL viability. Leukemia and Lymphoma 2007. 48 (9) 1855-1857.

Robert, Th, Kawkabani Marchini, A., Oumarou, G., and Uske, A. Reversible cerebral vasoconstriction syndrome identification of prognostic factors. Clinical neurology and neurosurgery 2013. 115 (11) 2351-2357.

Rose, D. Z., Guerrero, W. R., Mokin, M. V., Gooch, C. L., Bozeman, A. C., Pearson, J. M., and Scott, Burgin W. Hemorrhagic stroke following use of the synthetic marijuana spice. Neurology 2015. 85 (13) 1177-1179.

Ruppert, A. M., Amrioui, F., Giol, M., Assouad, J., Cadranel, J., Gounant, V. Prevalence of tobacco and cannabis use in a prospective cohort of spontaneous pneumothorax and cessation rate at 6 months. Respiratory medicine and research 2020. 78, 100793.

Salam, A. P. and Pozniak, A. L. Disseminated aspergillosis in an HIV-positive cannabis user taking steroid treatment. The Lancet Infectious Diseases 2017. 17 (8) 882.

Scaturro, Dalila, Asaro, Chiara, Lauricella, Lorenza, Letizia Mauro, Giulia, Tomasello, Sofia, Varrassi, Giustino. Combination of Rehabilitative Therapy with Ultramicronized Palmitoylethanolamide for Chronic Low Back Pain: An Observational Study. Pain and Therapy 2020. 9 (1) 319-326.

Schicho, R. and Storr, M. IBD: Patients with IBD find symptom relief in the Cannabis field. Nature Reviews Gastroenterology and Hepatology 2014. 11 (3) 142-143.

Schierenbeck, Thomas, Riemann, Dieter, Berger, Mathias, and Hornyak, Magdolna. Effect of illicit recreational drugs upon sleep: cocaine, ecstasy and marijuana. Sleep Medicine Reviews 2008. 12 (5) 381-389.

Schwarcz, Glenn, Karajgi, Basawaraj, and McCarthy, Richard. Synthetic delta-9-tetrahydrocannabinol (dronabinol) can improve the symptoms of schizophrenia. Journal of Clinical Psychopharmacology 2009. 29 (3) 255-258.

Scott-Thomas, Suzanne. Patterns of medical cannabis use differ between cancer and non-cancer patients. Clinical Pharmacist 2019. 11 (6).

Scully, C. Cannabis; adverse effects from an oromucosal spray. British dental journal 2007. 203 (6) E12-E17.

Solanki, Ram Kumar, Singh, Paramjeet, Gupta, Suresh, and Swami, Mukesh Kumar. Psychiatric morbidity associated with cannabis use: Varying presentation. Mental Health and Substance Use 2011. 4 (4) 348-350.

Storholm, Erik David, Silverberg, Michael J., and Satre, Derek D. Racial and Ethnic Differences in Substance Use Diagnoses, Comorbid Psychiatric Disorders, and Treatment Initiation among HIV-Positive and HIV-Negative Women in an Integrated Health Plan. Journal of Psychoactive Drugs 2016. 48 (5) 377-383.

Strouse, Thomas B. Clinical trials of cannabinoids in palliative medicine. Journal of Palliative Medicine 2020. 23 (5) 596-597.

Sulak, Dustin, Saneto, Russell, and Goldstein, Bonni. The current status of artisanal cannabis for the treatment of epilepsy in the United States. Epilepsy & behavior : E&B 2017. 70 (Pt B) 328-333.

Tan, W. C. and Sin, D. D. What are the long-term effects of smoked marijuana on lung health?. CMAJ 2018. 190 (42) E1243-E1244.

Touil, Nassim, Lavand'homme, Patricia. Cannabis hyperalgesia: A phenomenon underestimated in the peri-operative period?. European Journal of Anaesthesiology 2019. 36 (9) 623-624.

Uddin, Md Jamal, Nordentoft, Merete, Hjorthoj, Carsten, Dalsgaard, Soren, Wimberley, Theresa, Hougaard, David M., Borglum, Anders, Werge, Thomas. No evidence of associations between genetic liability for schizophrenia and development of cannabis use disorder. Psychological medicine 2019., 1-6.

Valdovinos, Erica M., Frazee, Bradley W., Hailozian, Christian, Haro, Daniel A., Herring, Andrew A. A Nonopioid, Nonbenzodiazepine Treatment Approach for Intractable Nausea and Vomiting in the Emergency Department. Journal of clinical gastroenterology 2020. 54 (4) 327-332.

van Esbroeck, Annelot C. M., van Rooden, Eva J., van der Stelt, Mario, Pacher, Pal, Varga, Zoltan V., Toth, Viktoria E., Onodi, Zsofia, Di, Xinyu, Hankemeier, Thomas, Kusmierczyk, Mariusz, Leszek, Przemyslaw, Ferdinandy, Peter. Activity-based protein profiling of the human failing ischemic heart reveals alterations in hydrolase activities involving the endocannabinoid system. Pharmacological Research 2020. 151, 104578.

van Ours, Jan C. Is cannabis a stepping-stone for cocaine?. Journal of health economics 2003. 22 (4) 539-554.

VanLandingham, Kevan E., Crockett, Julie, Taylor, Lesley, Morrison, Gilmour. A Phase 2, Double-Blind, Placebo-Controlled Trial to Investigate Potential Drug-Drug Interactions Between Cannabidiol and Clobazam. Journal of clinical pharmacology 2020. 60 (10) 1304-1313.

Walter, Carmen, Oertel, Bruno G., Felden, Lisa, Noth, Ulrike, Deichmann, Ralf, Lotsch, Jorn. Delta-9-tetrahydrocannabinol reduces the performance in sensory delayed discrimination tasks. A pharmacological-fMRI study in healthy volunteers. IBRO reports 2019. 7, 117-128.

Wenger, David S., Crothers, Kristina. Marijuana Smoking in Men with HIV Infection: A Cause for Concern. EClinicalMedicine 2019. 7, 5-6.

Wijnen, B., Armstrong, N., Ramaekers, B., Witlox, W., Westwood, M., Fayter, D., Ryder, S., Buksnys, T., Worthy, G., Misso, K., et al. Cannabidiol for Adjuvant Treatment of Seizures Associated with Lennox–Gastaut Syndrome and Dravet Syndrome: an Evidence Review Group Perspective of a NICE Single Technology Appraisal. PharmacoEconomics 2020.

Williams, C., Thompstone, J., and Wilkinson, M. Work-related contact urticaria to Cannabis sativa. Contact Dermatitis 2008. 58 (1) 62-63.

Wolff, V., Armspach, J.-P., Lauer, V., Rouyer, O., Bataillard, M., Marescaux, C., and Geny, B. Cannabis-related stroke: Myth or reality?. Stroke 2013. 44 (2) 558-563.

Wolff, Valerie and Jouanjus, Emilie. Strokes are possible complications of cannabinoids use. Epilepsy & behavior : E&B 2017. 70 (Pt B) 355-363.

Yankey, B. A., Rothenberg, R., Strasser, S., Ramsey-White, K., and Okosun, I. S. Effect of marijuana use on cardiovascular and cerebrovascular mortality: A study using the National Health and Nutrition Examination Survey linked mortality file. European Journal of Preventive Cardiology 2017. 24 (17) 1833-1840.

Yap, S. and Drummer, O. H. Prevalence of new psychoactive substances in Victorian fatally-injured drivers. Australian Journal of Forensic Sciences 2016. 48 (2) 230-243.

Yazici, A. B., Yazici, E., and Erol, A. Delirium and High Creatine Kinase and Myoglobin Levels Related to Synthetic Cannabinoid Withdrawal. Case Reports in Medicine 2017. 2017, 3894749.

Young, R. and Molina, A. More than bargained for: Pneumomediastinum and subcutaneous emphysema associated with synthetic cannabinoid use. BMJ Case Reports 2018. 11 (1) e228066.

Zobor, D., Strasser, T., Zobor, G., Schober, F., Messias, A., Strauss, O., Batra, A., and Zrenner, E. Ophthalmological assessment of cannabis-induced persisting perception disorder: Is there a direct retinal effect?. Documenta Ophthalmologica 2015. 130 (2) 121-130.

Zollner, J. P., Noda, A. H., Rosenow, F., and Strzelczyk, A. Improving post-hypoxic myoclonus using cannabidiol. Seizure 2019. 67, 38-39.

Zuardi, A. W., Hallak, J. E. C., Dursun, S. M., Morais, S. L., Sanches, R. F., Musty, R. E., and Crippa, J. A. S. Cannabidiol monotherapy for treatment-resistant schizophrenia. Journal of Psychopharmacology 2006. 20 (5) 683-686.

Full text not available

Aggarwal, Sunil K., Carter, Gregory T., Sullivan, Mark D., Zumbrunnen, Craig, Morrill, Richard, and Mayer, Jonathan D. Characteristics of patients with chronic pain accessing treatment with medical cannabis in Washington State. Journal of opioid management 2009. 5 (5) 257-286.

Calvino, B. Cannabinoids and pain. Douleurs 2007. 8 (4) 218-226.

Dickson, C., Bruno, R., and Brown, J. Investigating the role of serotonin in visual orientation processing using an 'ecstasy' (MDMA)-based research model. Neuropsychobiology 2009. 60 (3-4) 204-212.

Dissiz, Melike. The effect of heroin, cannabis and cocaine addiction on sexual functions and depression of women: A comparative cross-sectional study. Heroin Addiction and Related Clinical Problems 2019. 21 (4) 15-22.

Estler, C.-J. Causes and classification of adverse drug reactions. Chirurgische Praxis 2002. 60 (2) 321-330.

Flachenecker, Peter, Henze, Thomas, and Zettl, Uwe K. Nabiximols (THC/CBD oromucosal spray, Sativex) in clinical practice-Results of a multicenter, non-interventional study (MOVE 2) in patients with multiple sclerosis spasticity. European Neurology 2014. 71 (5-6) 271-279.

Jalal, M. Smoking Hashish (chars) a partial blessing in disguise. Medical Forum Monthly 2015. 26 (4) 14-16.

Jan, Sameer Ul Khaliq, Ali, Ahmad, Asad, Amir Zada. Assessing the medical and psychological problems faced by 'majun-e-falak sair' addicts. Heroin Addiction and Related Clinical Problems 2020. 22 (2) 29-34.

Kintz, P., Cirimele, V., Mairot, F., Muhlmann, M., and Ludes, B. [Drug tests on 198 drivers involved in an accident]. Presse medicale (Paris, France : 1983) 2000. 29 (23) 1275-1278.

Martin-Soelch, C., Kobel, M., Stoecklin, M., Michael, T., Weber, S., Krebs, B., and Opwis, K. Reduced response to reward in smokers and cannabis users. Neuropsychobiology 2009. 60 (2) 94-103.

Matthews, A. J. and Bruno, R. An investigation of factors associated with depressive symptoms among a sample of regular ecstasy consumers. Neuropsychobiology 2010. 61 (4) 215-222.

Renard, O., Chvetzoff, G., Corbin, S., Drouet, Y., Lasset, C. Cannabis and analgesic management: What are the consequences for the prescription of strong opioids? Observational study at the Leon-Berard center in a lung cancer patients cohort. Douleur et Analgesie 2019. 32 (2) 82-86.

Potentially relevant RCT or SR and added to the appropriate database for screening

Ruthirakuhan, Myuri, Herrmann, Nathan, Andreazza, Ana C., Verhoeff, Nicolaas Paul L. G., Gallagher, Damien, Black, Sandra E., Kiss, Alex, Lanctot, Krista L. Agitation, Oxidative Stress, and Cytokines in Alzheimer Disease: Biomarker Analyses From a Clinical Trial With Nabilone for Agitation. Journal of geriatric psychiatry and neurology 2020. 33 (4) 175-184.

Did not meet age criteria

Abouchedid, Rachelle, Hudson, Simon, Thurtle, Natalie, Yamamoto, Takahiro, Ho, James H., Bailey, George, Wood, Michelle, Sadones, Nele, Stove, Christophe P., Dines, Alison, Archer, John R. H., Wood, David M., and Dargan, Paul I. Analytical confirmation of synthetic cannabinoids in a cohort of 179 presentations with acute recreational drug toxicity to an Emergency Department in London, UK in the first half of 2015. Clinical toxicology (Philadelphia, Pa.) 2017. 55 (5) 338-345.

Agrawal, Arpana, Tillman, Rebecca, Grucza, Richard A., Nelson, Elliot C., McCutcheon, Vivia V., Few, Lauren, Conner, Kenneth R., Lynskey, Michael T., Dick, Danielle M., Edenberg, Howard J., Hesselbrock, Victor M., Kramer, John R., Kuperman, Samuel, Nurnberger, John I. J., Schuckit, Marc A., Porjesz, Bernice, and Bucholz, Kathleen K. Reciprocal relationships between substance use and disorders and suicidal ideation and suicide attempts in the Collaborative Study of the Genetics of Alcoholism. Journal of Affective Disorders 2017. 213, 96-104.

Alameda, Luis, Golay, Philippe, Baumann, Philipp, Morandi, Stephane, Ferrari, Carina, Conus, Philippe, and Bonsack, Charles. Assertive outreach for "difficult to engage" patients: A useful tool for a subgroup of patients in specialized early psychosis intervention programs. Psychiatry Research 2016. 239, 212-219.

Alibrahim, Osama A., Misau, Yusuf A., Mohammed, Alkali, Faruk, Mohammad Bashir, and Ss, Izzeldin. Prevalence of hepatitis C viral infection among injecting drug users in a Saudi Arabian hospital: A point cross sectional survey. Journal of public health in Africa 2018. 9 (1) 726.

Allsop, D. J. and Copeland, J. Age at first cannabis use moderates EEG markers of recovery from cannabis. Journal of Substance Use 2016. 21 (4) 400-406.

Ames, S. L., Grenard, J. L., Stacy, W., Xiao, L., He, Q., Wong, S. W., Xue, G., Wiers, W., and Bechara, A. Functional imaging of implicit marijuana associations during performance on an Implicit Association Test (IAT). Behavioural Brain Research 2013. 256, 494-502.

Anderson, Beth M., Rizzo, Matthew, Block, Robert I., Pearlson, Godfrey D., and O'Leary, Daniel S. Sex differences in the effects of marijuana on simulated driving performance. Journal of Psychoactive Drugs 2010. 42 (1) 19-30.

Angelucci, Francesco, Ricci, Valerio, Spalletta, Gianfranco, Pomponi, Massimiliano, Tonioni, Federico, Caltagirone, Carlo, and Bria, Pietro. Reduced serum concentrations of nerve growth factor, but not brain-derived neurotrophic factor, in chronic cannabis abusers. European neuropsychopharmacology : the journal of the European College of Neuropsychopharmacology 2008. 18 (12) 882-887.

Arendt, M., Rosenberg, R., Fjordback, L., Brandholdt, J., Foldager, L., Sher, L., and Munk-Jorgensen, P. Testing the self-medication hypothesis of depression and aggression in cannabis-dependent subjects. Psychological Medicine 2007. 37 (7) 935-945.

Arendt, Mikkel, Rosenberg, Raben, Foldager, Leslie, Sher, Leo, and Munk-Jorgensen, Povl. Withdrawal symptoms do not predict relapse among subjects treated for cannabis dependence. The American journal on addictions 2007. 16 (6) 461-467.

Arranz, Sara, Monferrer, Nuria, Jose Algora, M., Cabezas, Angel, Sole, Montse, Vilella, E., Labad, J., and Sanchez-Gistau, Vanessa. The relationship between the level of exposure to stress factors and cannabis in recent onset psychosis. Schizophrenia Research 2018. 201, 352-359.

Ashley, David L., De Jesus, Victor R., Blount, Benjamin C., Abulseoud, Osama A., Huestis, Marilyn A., Milan, Daniel F. Urinary acrylonitrile metabolite concentrations before and after smoked, vaporized, and oral cannabis in frequent and occasional cannabis users. International Journal of Environmental Research and Public Health 2020. 17 (18) 1-11.

Asmaro, Deyar, Carolan, Patrick L., and Liotti, Mario. Electrophysiological evidence of early attentional bias to drug-related pictures in chronic cannabis users. Addictive Behaviors 2014. 39 (1) 114-121.

Atsmon, J., Cherniakov, I., Izgelov, D., Hoffman, A., Domb, A. J., Deutsch, L., Deutsch, F., Heffetz, D., and Sacks, H. PTL401, a New Formulation Based on Pro-Nano Dispersion Technology, Improves Oral Cannabinoids Bioavailability in Healthy Volunteers. Journal of pharmaceutical sciences 2018. 107 (5) 1423-1429.

Austin, Amy E., van den Heuvel, Corinna, and Byard, Roger W. Causes of community suicides among indigenous South Australians. Journal of forensic and legal medicine 2011. 18 (7) 299-301.

Auther, A. M., Cadenhead, K. S., Carrion, R. E., Addington, J., Bearden, C. E., Cannon, T. D., McGlashan, T. H., Perkins, D. O., Seidman, L., Tsuang, M., Walker, E. F., Woods, S. W., and Cornblatt, B. A. Alcohol confounds relationship between cannabis misuse and psychosis conversion in a high-risk sample. Acta Psychiatrica Scandinavica 2015. 132 (1) 60-68.

Aversa, A., Rossi, F., Francomano, D., Bruzziches, R., Bertone, C., Santiemma, V., and Spera, G. Early endothelial dysfunction as a marker of vasculogenic erectile dysfunction in young habitual cannabis users. International journal of impotence research 2008. 20 (6) 566-573.

Badiani, Aldo, Boden, Joseph M., De Pirro, Silvana, Fergusson, David M., Horwood, L. John, and Harold, Gordon T. Tobacco smoking and cannabis use in a longitudinal birth cohort: evidence of reciprocal causal relationships. Drug and Alcohol Dependence 2015. 150, 69-76.

Bailey, E. L. and Swallow, B. L. The relationship between cannabis use and schizotypal symptoms. European Psychiatry 2004. 19 (2) 113-114.

Balan Moshe, Livia, Weizman, Abraham, Ben Dor, David H., Konas, Shai, Fischel, Zvi, Aizenberg, Dov, Gothelf, Doron, and Valevski, Avi. Differences in demographic and clinical characteristics between cannabis users and non-drug users: A retrospective study of patients at first hospitalization due to psychotic symptoms. Psychiatry Research 2018. 268, 454-459.

Bani-Ahmad, M. A., Mustafa, A. G., Ahmad, Aab, and Rahim, Ama. Assessment of oxidative stress of platelets among chronic heroin and hashish addicts. Human & experimental toxicology 2018. 37 (10) 1017-1024.

Barkus, E. J., Stirling, J., Hopkins, R. S., and Lewis, S. Cannabis-induced psychosis-like experiences are associated with high schizotypy. Psychopathology 2006. 39 (4) 175-178.

Barnes, Thomas R. E., Mutsatsa, Stanley H., Hutton, Sam B., Watt, Hilary C., and Joyce, Eileen M. Comorbid substance use and age at onset of schizophrenia. The British journal of psychiatry : the journal of mental science 2006. 188, 237-242.

Barnett, Jennifer H., Werners, Ursula, Secher, Sandra M., Hill, Katherine E., Brazil, Rossa, Masson, Kim, Pernet, David E., Kirkbride, James B., Murray, Graham K., Bullmore, Ed T., and Jones, Peter B. Substance use in a population-based clinic sample of people with first-episode psychosis. The British journal of psychiatry : the journal of mental science 2007. 190, 515-520.

Barrowclough, C., Gregg, L., Lobban, F., Bucci, S., and Emsley, R. The Impact of Cannabis Use on Clinical Outcomes in Recent Onset Psychosis. Schizophrenia Bulletin 2015. 41 (2) 382-390.

Battistella, Giovanni, Fornari, Eleonora, Annoni, Jean Marie, Chtioui, Haithem, Dao, Kim, Fabritius, Marie, Favrat, Bernard, Mall, Jean Frederic, Maeder, Philippe, and Giroud, Christian. Long-term effects of cannabis on brain structure. Neuropsychopharmacology : official publication of the American College of Neuropsychopharmacology 2014. 39 (9) 2041-2048.

Bayazit, H., Selek, S., Karababa, I. F., Cicek, E., and Aksoy, N. Evaluation of oxidant/antioxidant status and cytokine levels in patients with cannabis use disorder. Clinical Psychopharmacology and Neuroscience 2017. 15 (3) 237-242.

Bayazit, Huseyin, Cicek, Erdinc, Selek, Salih, Aksoy, Nurten, Karababa, I. Fatih, Kandemir, Hasan, and Basmaci Kandemir, Sultan. Increased S100B Levels in Cannabis Use Disorder. European Addiction Research 2016. 22 (4) 177-180.

Beaudoin, Melissa, Potvin, Stephane, Dellazizzo, Laura, Luigi, Mimosa, Giguere, Charles Edouard, and Dumais, Alexandre. Trajectories of Dynamic Risk Factors as Predictors of Violence and Criminality in Patients Discharged From Mental Health Services: A Longitudinal Study Using Growth Mixture Modeling. Frontiers in Psychiatry 2019. 10, 301.

Beraha, E. M., Cousijn, J., Hermanides, E., Goudriaan, A. E., and Wiers, R. W. Implicit associations and explicit expectancies toward cannabis in heavy cannabis users and controls. Frontiers in Psychiatry 2013. 4 (JUN) Article.

Berard, Annie M., Bedel, Aurelie, Le Trequesser, Remi, Freyburger, Genevieve, Nurden, Alan, Colomer, Sylvie, Guerin, Viviane, Vergnes, Marie Christine, Becker, Francois, Camelot, Gabriel, Bressolette, Luc, Lacroix, Philippe, Cambou, Jean Pierre, Bura-Riviere, Alessandra, Emmerich, Joseph, Darmon, Michel, Deletraz, Anne Marie, Mesli, Samir, Colombies, Brigitte, Vanbrugghe, Virginie, Conri, Claude, and Constans, Joel. Novel risk factors for premature peripheral arterial occlusive disease in non-diabetic patients: a case-control study. PLoS ONE 2013. 8 (3) e37882.

Berge, Daniel, Mane, Anna, Salgado, Purificacion, Cortizo, Romina, Garnier, Carolina, Gomez, Laura, Diez-Aja, Cristobal, Bulbena, Antoni, and Perez, Victor. Predictors of Relapse and Functioning in First-Episode Psychosis: A Two-Year Follow-Up Study. Psychiatric services (Washington, D.C.) 2016. 67 (2) 227-233.

Bersani, G., Bersani, F. S., Caroti, E., Russo, P., Albano, G., Valeriani, G., Imperatori, C., Minichino, A., Manuali, G., and Corazza, O. Negative symptoms as key features of depression among cannabis users: A preliminary report. European review for medical and pharmacological sciences 2016. 20 (3) 547-552.

Blagrove, M., Seddon, J., George, S., Parrott, A. C., Stickgold, R., Walker, M. P., Jones, K. A., and Morgan, M. J. Procedural and declarative memory task performance, and the memory consolidation function of sleep, in recent and abstinent ecstasy/MDMA users. Journal of Psychopharmacology 2011. 25 (4) 465-477.

Blevins, C. E., Anderson, B. J., Caviness, C. M., Herman, D. S., and Stein, M. D. Emerging Adults' Discussion of Substance Use and Sexual Behavior with Providers. Journal of health communication 2019. 24 (2) 121-128.

Boks, Marco P., van Hell, Hendrika H., Ramsey, Nick F., Bossong, Matthijs G., van Saane, Wesley, Schubart, Chris D., Iseger, Tabitha A., Jager, Gerry, van Osch, Matthias J. P., Jansma, J. Martijn, Kahn, Rene S. Acute effects of 9-tetrahydrocannabinol (THC) on resting state brain function and their modulation by COMT genotype. European Neuropsychopharmacology 2019. 29 (6) 766-776.

Bolla, Karen I., Lesage, Suzanne R., Gamaldo, Charlene E., Neubauer, David N., Funderburk, Frank R., Cadet, Jean Lud, David, Paula M., Verdejo-Garcia, Antonio, and Benbrook, Amy R. Sleep disturbance in heavy marijuana users. Sleep 2008. 31 (6) 901-908.

Bordnick, P. S., Copp, H. L., Traylor, A., Graap, K. M., Carter, B. L., Walton, A., and Ferrer, M. Reactivity to cannabis cues in virtual reality environments. Journal of Psychoactive Drugs 2009. 41 (2) 105-112.

Bossong, Matthijs G., Jansma, J. Martijn, van Hell, Hendrika H., Jager, Gerry, Kahn, Rene S., and Ramsey, Nick F. Default mode network in the effects of DELTA9-Tetrahydrocannabinol (THC) on human executive function. PLoS ONE 2013. 8 (7) e70074.

Bossong, Matthijs G., van Hell, Hendrika H., Jager, Gerry, Kahn, Rene S., Ramsey, Nick F., and Jansma, J. Martijn. The endocannabinoid system and emotional processing: a pharmacological fMRI study with 9-tetrahydrocannabinol. European neuropsychopharmacology : the journal of the European College of Neuropsychopharmacology 2013. 23 (12) 1687-1697.

Brandehoff, N., Adams, A., McDaniel, K., Banister, S. D., Gerona, R., and Monte, A. A. Synthetic cannabinoid "Black Mamba" infidelity in patients presenting for emergency stabilization in Colorado: a P SCAN Cohort. Clinical Toxicology 2018. 56 (3) 193-198.

Brook, Judith S., Lee, Jung Yeon, Finch, Stephen J., and Brook, David W. Developmental trajectories of marijuana use from adolescence to adulthood: relationship with using weapons including guns. Aggressive behavior 2014. 40 (3) 229-237.

Brucato, G., Masucci, M. D., Arndt, L. Y., Ben-David, S., Colibazzi, T., Corcoran, C. M., Crumbley, A. H., Crump, F. M., Gill, K. E., Kimhy, D., Lister, A., Schobel, S. A., Yang, L. H., Lieberman, J. A., and Girgis, R. R. Baseline demographics, clinical features and predictors of conversion among 200 individuals in a longitudinal prospective psychosis-risk cohort. Psychological Medicine 2017. 47 (11) 1923-1935.

Brunette, M. F., Mueser, K. T., Babbin, S., Meyer-Kalos, P., Rosenheck, R., Correll, C. U., Cather, C., Robinson, D. G., Schooler, N. R., Penn, D. L., Addington, J., Estroff, S. E., Gottlieb, J., Glynn, S. M., Marcy, P., Robinson, J., and Kane, J. M. Demographic and clinical correlates of substance use disorders in first episode psychosis. Schizophrenia Research 2018. 194, 4-12.

Buchanan, Tom, Heffernan, Thomas M., Parrott, Andrew C., Ling, Jonathan, Rodgers, Jacqui, and Scholey, Andrew B. A short self-report measure of problems with executive function suitable for administration via the Internet. Behavior research methods 2010. 42 (3) 709-714.

Buchy, L., Cadenhead, K. S., Cannon, T. D., Cornblatt, B. A., McGlashan, T. H., Perkins, D. O., Seidman, L. J., Tsuang, M. T., Walker, E. F., Woods, S. W., Heinssen, R., Bearden, C. E., Mathalon, D., and Addington, J. Substance use in individuals at clinical high risk of psychosis. Psychological Medicine 2015. 45 (11) 2275-2284.

Buchy, L., Seidman, L. J., Cadenhead, K. S., Cannon, T. D., Cornblatt, B. A., McGlashan, T. H., Perkins, D. O., Stone, W., Tsuang, M. T., Walker, E. F., Woods, S. W., Bearden, C. E., Mathalon, D. H., and Addington, J. Evaluating the relationship between cannabis use and IQ in youth and young adults at clinical high risk of psychosis. Psychiatry Research 2015. 230 (3) 878-884.

Buckner, J. D. and Schmidt, N. B. Social anxiety disorder and marijuana use problems: The mediating role of marijuana effect expectancies. Depression and Anxiety 2009. 26 (9) 864-870.

Buckner, J. D., Mallott, M. A., Schmidt, N. B., and Taylor, J. Peer influence and gender differences in problematic cannabis use among individuals with social anxiety. Journal of anxiety disorders 2006. 20 (8) 1087-1102.

Buckner, J. D., Zvolensky, M. J., Crosby, R. D., Wonderlich, S. A., Ecker, A. H., and Richter, A. Antecedents and consequences of cannabis use among racially diverse cannabis users: An analysis from ecological momentary assessment. Drug and Alcohol Dependence 2015. 147, 20-25.

Buhler, Babette, Hambrecht, Martin, Loffler, Walter, an der Heiden, Wolfram, and Hafner, Heinz. Precipitation and determination of the onset and course of schizophrenia by substance abuse--a retrospective and prospective study of 232 population-based first illness episodes. Schizophrenia Research 2002. 54 (3) 243-251.

Burns, J., Jhazbhay, K., and Emsley, R. Cannabis use predicts shorter duration of untreated psychosis and lower levels of negative symptoms in first-episode psychosis: A South African study. African Journal of Psychiatry (South Africa) 2010. 13 (5) 395-399.

Carey, Susan E., Nestor, Liam, Jones, Jennifer, Garavan, Hugh, and Hester, Robert. Impaired learning from errors in cannabis users: Dorsal anterior cingulate cortex and hippocampus hypoactivity. Drug and Alcohol Dependence 2015. 155, 175-182.

Cengel, Hanife Yilmaz, Bozkurt, Muge, Evren, Cuneyt, Umut, Gokhan, Keskinkilic, Cahit, and Agachanli, Ruken. Evaluation of cognitive functions in individuals with synthetic cannabinoid use disorder and comparison to individuals with cannabis use disorder. Psychiatry Research 2018. 262, 46-54.

Chao, T., Radoncic, V., Hien, D., Bedi, G., and Haney, M. Stress responding in cannabis smokers as a function of trauma exposure, sex, and relapse in the human laboratory. Drug and Alcohol Dependence 2018. 185, 23-32.

Chaudhary, Shraddha. Event related potentials in subjects of cannabis induced psychosis 'schizophrenia like' and cannabis dependent subjects without psychosis: A comparative study. Dissertation Abstracts International: Section B: The Sciences and Engineering 2020. 81 (9-B) No-Specified.

Chawla, Devika, Yang, Yang C., Desrosiers, Tania A., Westreich, Daniel J., Olshan, Andrew F., and Daniels, Julie L. Past-month cannabis use among U.S. individuals from 2002-2015: An age-period-cohort analysis. Drug and Alcohol Dependence 2018. 193, 177-182.

Clough, A. R., D'Abbs, P., Cairney, S., Gray, D., Maruff, P., Parker, R., and O'Reilly, B. Adverse mental health effects of cannabis use in two indigenous communities in Arnhem Land, Northern Territory, Australia: Exploratory study. Australian and New Zealand Journal of Psychiatry 2005. 39 (7) 612-620.

Colder, Craig R., Lee, Yong Hee, Frndak, Seth, Read, Jennifer P., Wieczorek, William F. Internalizing symptoms and cannabis and alcohol use: Between- and within-person risk pathways with coping motives. Journal of consulting and clinical psychology 2019. 87 (7) 629-644.

Colizzi, Marco, Burnett, Natoy, Costa, Rosalia, De Agostini, Mattia, Griffin, James, and Bhattacharyya, Sagnik. Longitudinal assessment of the effect of cannabis use on hospital readmission rates in early psychosis: A 6-year follow-up in an inpatient cohort. Psychiatry Research 2018. 268, 381-387.

Compton, M. T., Furman, A. C., and Kaslow, N. J. Lower negative symptom scores among cannabis-dependent patients with schizophrenia-spectrum disorders: Preliminary evidence from an African American first-episode sample. Schizophrenia Research 2004. 71 (1) 61-64.

Compton, Michael T., Kelley, Mary E., Ramsay, Claire E., Pringle, Makenya, Goulding, Sandra M., Esterberg, Michelle L., Stewart, Tarianna, and Walker, Elaine F. Association of pre-onset cannabis, alcohol, and tobacco use with age at onset of prodrome and age at onset of psychosis in first-episode patients. The American journal of psychiatry 2009. 166 (11) 1251-1257.

Cookey, Jacob, Crocker, Candice E., Bernier, Denise, Newman, Aaron J., Stewart, Sherry, McAllindon, David, and Tibbo, Philip G. Microstructural Findings in White Matter Associated with Cannabis and Alcohol Use in Early-Phase Psychosis: A Diffusion Tensor Imaging and Relaxometry Study. Brain connectivity 2018. 8 (9) 567-576.

Cookey, Jacob, McGavin, Jacob, Crocker, Candice E., Matheson, Kara, Stewart, Sherry H., Tibbo, Philip G. A Retrospective Study of the Clinical Characteristics Associated with Alcohol and Cannabis use in Early Phase Psychosis. Canadian journal of psychiatry. Revue canadienne de psychiatrie 2020. 65 (6) 426-435.

Cooper, Ziva D. and Haney, Margaret. Sex-dependent effects of cannabis-induced analgesia. Drug and Alcohol Dependence 2016. 167, 112-120.

Corcoran, Cheryl M., Kimhy, David, Stanford, Arielle, Khan, Shamir, Walsh, Julie, Thompson, Judy, Schobel, Scott, Harkavy-Friedman, Jill, Goetz, Ray, Colibazzi, Tiziano, Cressman, Victoria, and Malaspina, Dolores. Temporal association of cannabis use with symptoms in individuals at clinical high risk for psychosis. Schizophrenia Research 2008. 106 (2-3) 286-293.

Corley, Robin P., Winiger, Evan A., Morrison, Claire L., Hewitt, John K., Ellingson, Jarrod M., Hopfer, Christian J., Pasman, Joelle A., Wall, Tamara L. Sleep deficits and cannabis use behaviors: an analysis of shared genetics using linkage disequilibrium score regression and polygenic risk prediction. Sleep 2021. 44 (3).

Coulston, Carissa M., Perdices, Michael, and Tennant, Christopher C. The neuropsychological correlates of cannabis use in schizophrenia: lifetime abuse/dependence, frequency of use, and recency of use. Schizophrenia Research 2007. 96 (1-3) 169-184.

Cousijn, J., Watson, P., Koenders, L., Vingerhoets, W. A. M., Goudriaan, A. E., and Wiers, R. W. Cannabis dependence, cognitive control and attentional bias for cannabis words. Addictive Behaviors 2013. 38 (12) 2825-2832.

Cousijn, Janna, Goudriaan, Anna E., and Wiers, Reinout W. Reaching out towards cannabis: approach-bias in heavy cannabis users predicts changes in cannabis use. Addiction (Abingdon, England) 2011. 106 (9) 1667-1674.

Cousijn, Janna, Goudriaan, Anna E., Ridderinkhof, K. Richard, van den Brink, Wim, Veltman, Dick J., and Wiers, Reinout W. Approach-bias predicts development of cannabis problem severity in heavy cannabis users: results from a prospective FMRI study. PLoS ONE 2012. 7 (9) e42394.

Cousijn, Janna, Goudriaan, Anna E., Ridderinkhof, K. Richard, van den Brink, Wim, Veltman, Dick J., and Wiers, Reinout W. Neural responses associated with cue-reactivity in frequent cannabis users. Addiction Biology 2013. 18 (3) 570-580.

Cousijn, Janna, Wiers, Reinout W., Ridderinkhof, K. Richard, van den Brink, Wim, Veltman, Dick J., and Goudriaan, Anna E. Effect of baseline cannabis use and working-memory network function on changes in cannabis use in heavy cannabis users: a prospective fMRI study. Human Brain Mapping 2014. 35 (5) 2470-2482.

Cousijn, Janna, Wiers, Reinout W., Ridderinkhof, K. Richard, van den Brink, Wim, Veltman, Dick J., and Goudriaan, Anna E. Grey matter alterations associated with cannabis use: Results of a VBM study in heavy cannabis users and healthy controls. NeuroImage 2012. 59 (4) 3845-3851.

Cousijn, Janna, Wiers, Reinout W., Ridderinkhof, K. Richard, van den Brink, Wim, Veltman, Dick J., Porrino, Linda J., and Goudriaan, Anna E. Individual differences in decision making and reward processing predict changes in cannabis use: a prospective functional magnetic resonance imaging study. Addiction Biology 2013. 18 (6) 1013-1023.

Cowan, R. L., Joers, J. M., and Dietrich, M. S. N-acetylaspartate (NAA) correlates inversely with cannabis use in a frontal language processing region of neocortex in MDMA (Ecstasy) polydrug users: A 3 T magnetic resonance spectroscopy study. Pharmacology Biochemistry and Behavior 2009. 92 (1) 105-110.

Crippa, Jose Alexandre S., Zuardi, Antonio Waldo, Hallak, Jaime Eduardo Cecilio, Bernardo, Sandra Aparecido, Miyazawa, Bruna, Donaduzzi, Carmem Maria, Da Rosa Zimmermann, Patricia Moura, Rechia, Leticia Mello, Jose Tondo Filho, Volnei, Brum Junior, Liberato, Guzzi, Silvane, Favreto, Wagner Alex Jann, Campos, Alline, Guimaraes, Francisco S., Queiroz, Maria Eugenia C. Oral Cannabidiol Does Not Convert to DELTA8-THC or DELTA9-THC in Humans: A Pharmacokinetic Study in Healthy Subjects. Cannabis and Cannabinoid Research 2020. 5 (1) 89-98.

Cunha, P. J., Rosa, P. G. P., Ayres, A. D. M., Duran, F. L. S., Santos, L. C., Scazufca, M., Menezes, P. R., dos, Santos B., Murray, R. M., Crippa, J. A. S., Busatto, G. F., and Schaufelberger, M. S. Cannabis use, cognition and brain structure in first-episode psychosis. Schizophrenia Research 2013. 147 (2-3) 209-215.

Cunradi, Carol B., Lee, Juliet, Pagano, Anna, Caetano, Raul, Alter, Harrison J. Gender Differences in Smoking Among an Urban Emergency Department Sample. Tobacco use insights 2019. 12, 1179173X19879136.

Da Silva, Tania, Hafizi, Sina, Watts, Jeremy J., Weickert, Cynthia Shannon, Meyer, Jeffrey H., Houle, Sylvain, Rusjan, Pablo, Mizrahi, Romina. "In vivo imaging of translocator protein in long-term cannabis users": Correction. JAMA Psychiatry 2019. 76 (12) 1319.

Da Silva, Tania, Hafizi, Sina, Watts, Jeremy J., Weickert, Cynthia Shannon, Meyer, Jeffrey H., Houle, Sylvain, Rusjan, Pablo, Mizrahi, Romina. In Vivo Imaging of Translocator Protein in Long-term Cannabis Users. JAMA psychiatry 2019. 76 (12) 1305-1313.

Da Silva, Tania, Watts, Jeremy J., Garani, Ranjini, Lalang, Nittha, Chavez, Sofia, Mizrahi, Romina. Evidence That Cannabis Exposure, Abuse, and Dependence Are Related to Glutamate Metabolism and Glial Function in the Anterior Cingulate Cortex: A 1H-Magnetic Resonance Spectroscopy Study. Frontiers in Psychiatry 2020. 11, 764.

Dafters, R. I., Hoshi, R., and Talbot, A. C. Contribution of cannabis and MDMA ("ecstasy") to cognitive changes in long-term polydrug users. Psychopharmacology 2004. 173 (3-4) 405-410.

Dahlgren, Mary Kathryn. Assessing within-task time course of cognitive interference processing in recreational marijuana users. Dissertation Abstracts International: Section B: The Sciences and Engineering 2018. 79 (10-B(E)) No-Specified.

Danielsson, Anna Karin, Lundin, Andreas, Allebeck, Peter, and Agardh, Emile. Cannabis use and psychological distress: An 8-year prospective population-based study among Swedish men and women. Addictive Behaviors 2016. 59, 18-23.

Darling, Mark R., Learmonth, Genevieve M., and Arendorf, Trevor M. Oral cytology in cannabis smokers. SADJ : journal of the South African Dental Association = tydskrif van die Suid-Afrikaanse Tandheelkundige Vereniging 2002. 57 (4) 132-135.

Daumann, J., Pelz, S., Becker, S., Tuchtenhagen, F., and Gouzoulis-Mayfrank, E. Psychological profile of abstinent recreational Ecstasy (MDMA) users and significance of concomitant cannabis use. Human psychopharmacology 2001. 16 (8) 627-633.

Davis, Christal N., Slutske, Wendy S., Martin, Nicholas G., Agrawal, Arpana, Lynskey, Michael T. Identifying subtypes of cannabis users based on simultaneous polysubstance use. Drug and alcohol dependence 2019. 205, 107696.

De Genna, Natacha M., Feske, Ulrike, Angiolieri, Teresa, and Gold, Melanie A. Race and sexually transmitted diseases in women with and without borderline personality disorder. Journal of women's health (2002) 2011. 20 (3) 333-340.

de Sousa Fernandes Perna, E. B., Theunissen, E. L., Kuypers, K. P. C., Toennes, S. W., and Ramaekers, J. G. Subjective aggression during alcohol and cannabis intoxication before and after aggression exposure. Psychopharmacology 2016. 233 (18) 3331-3340.

de Vos, Chloe, Leopold, Karolina, Blanke, Elisabeth S., Siebert, Stefan, Baumgardt, Johanna, Burkhardt, Eva, Bechdolf, Andreas. The relationship between cannabis use and cognition in people diagnosed with first-episode psychosis. Psychiatry research 2020. 293, 113424.

Degenhardt, Louisa, Roxburgh, Amanda, and McKetin, Rebecca. Hospital separations for cannabis- and methamphetamine-related psychotic episodes in Australia. The Medical journal of Australia 2007. 186 (7) 342-345.

Dekker, N., Meijer, J., Koeter, M., van den Brink, W., van Beveren, N., Kahn, R. S., Linszen, D. H., van Os, J., Wiersma, D., Bruggeman, R., Cahn, W., de Haan, L., Krabbendam, L., Myin-Germeys, I., and GROUP, investigators. Age at onset of non-affective psychosis in relation to cannabis use, other drug use and gender. Psychological Medicine 2012. 42 (9) 1903-1911.

Delgado-Sequera, Alejandra, Hidalgo-Figueroa, Maria, Berrocoso, Esther, Barrera-Conde, Marta, Fernandez-Aviles, Cristina, Robledo, Patricia, Duran-Ruiz, M. Carmen, Castro, Carmen, Sanchez-Gomar, Ismael, de la Torre, Rafael, Perez, Victor, Geribaldi-Doldan, Noelia. Olfactory Neuroepithelium Cells from Cannabis Users Display Alterations to the Cytoskeleton and to Markers of Adhesion, Proliferation and Apoptosis. Molecular Neurobiology 2021. 58 (4) 1695-1710.

Delvecchio, Giuseppe, Oldani, Lucio, Mandolini, Gian Mario, Pigoni, Alessandro, Ciappolino, Valentina, Schiena, Giandomenico, Lazzaretti, Matteo, Caletti, Elisabetta, Barbieri, Viviana, Cinnante, Claudia, Triulzi, Fabio, Brambilla, Paolo. Brain Morphology of Cannabis Users With or Without Psychosis: A Pilot MRI Study. Journal of visualized experiments : JoVE 2020. (162).

Demirakca, Traute, Sartorius, Alexander, Ende, Gabriele, Meyer, Nadja, Welzel, Helga, Skopp, Gisela, Mann, Karl, and Hermann, Derik. Diminished gray matter in the hippocampus of cannabis users: possible protective effects of cannabidiol. Drug and Alcohol Dependence 2011. 114 (2-3) 242-245.

Desrosiers, N. A., Ramaekers, J. G., Chauchard, E., Gorelick, D. A., and Huestis, M. A. Smoked cannabis' psychomotor and neurocognitive effects in occasional and frequent smokers. Journal of analytical toxicology 2015. 39 (4) 251-261.

Dierker, L., Mendoza, W., Goodwin, R., Selya, A., and Rose, J. Marijuana use disorder symptoms among recent onset marijuana users. Addictive Behaviors 2017. 68, 6-13.

Dovey, Terence M., Boyland, Emma J., Trayner, Penelope, Miller, Jo, Rarmoul-Bouhadjar, Amin, Cole, Jon, and Halford, Jason C. G. Alterations in taste perception due to recreational drug use are due to smoking a substance rather than ingesting it. Appetite 2016. 107, 1-8.

Dragt, S., Nieman, D. H., Schultze-Lutter, F., van der Meer, F., Becker, H., de Haan, L., Dingemans, P. M., Birchwood, M., Patterson, P., Salokangas, R. K. R., Heinimaa, M., Heinz, A., Juckel, G., Graf von Reventlow, H., French, P., Stevens, H., Ruhrmann, S., Klosterkotter, J., Linszen, D. H., and EPOS group. Cannabis use and age at onset of symptoms in subjects at clinical high risk for psychosis. Acta Psychiatrica Scandinavica 2012. 125 (1) 45-53.

Dregan, A. and Gulliford, M. C. Is illicit drug use harmful to cognitive functioning in the midadult years? A cohort-based investigation. American Journal of Epidemiology 2012. 175 (3) 218-227.

D'Souza, D. C., Cortes-Briones, J. A., Ranganathan, M., Thurnauer, H., Creatura, G., Surti, T., Planeta, B., Neumeister, A., Pittman, B., Normandin, M. D., Kapinos, M., Ropchan, J., Huang, Y., Carson, R. E., and Skosnik, P. D. Rapid Changes in Cannabinoid 1 Receptor Availability in Cannabis-Dependent Male Subjects after Abstinence from Cannabis. Biological Psychiatry: Cognitive Neuroscience and Neuroimaging 2016. 1 (1) 60-67.

Duckworth, J. C. and Lee, C. M. Associations among simultaneous and co-occurring use of alcohol and marijuana, risky driving, and perceived risk. Addictive Behaviors 2019. 96, 39-42.

Dugre, Jules R., Dellazizzo, Laura, Giguere, Charles Edouard, Potvin, Stephane, and Dumais, Alexandre. Persistency of Cannabis Use Predicts Violence following Acute Psychiatric Discharge. Frontiers in Psychiatry 2017. 8, 176.

Edwards, Chad R., Skosnik, Patrick D., Steinmetz, Adam B., O'Donnell, Brian F., and Hetrick, William P. Sensory gating impairments in heavy cannabis users are associated with altered neural oscillations. Behavioral neuroscience 2009. 123 (4) 894-904.

Egilmez, Oguzhan Bekir, Orum, Mehmet Hamdi, Kustepe, Ali, Karadag, Ayse Sevgi, Kalenderoglu, Aysun. Long-Term Substance Use Can Cause Irreversible Photopic Vision Changes in Substance Use Disorder in Remission. Psychiatry investigation 2020. 17 (10) 1037-1043.

Eiden, Celine, Cathala, Philippe, Mathieu-Daude, Jean Claude, Marson, Benjamin, Baccino, Eric, Leglise, Yves, and Peyriere, Helene. Methadone-related deaths in Montpellier and Region, from 2000 to 2010. Therapie 2012. 67 (6) 515-522.

El Maerrawi, Ilham and Carvalho, Heraclito Barbosa. Prevalence and risk factors associated with HIV infection, hepatitis and syphilis in a state prison of Sao Paulo. International journal of STD & AIDS 2015. 26 (2) 120-127.
[truncated: 660,895 more chars]
