# Supplementary material for: Impacts of medical and non-medical cannabis on the health of older adults: Findings from a scoping review of the literature
Source: PLoS One. 2023 Feb 17;18(2):e0281826. doi: 10.1371/journal.pone.0281826 (PMC9937508; doi:10.1371/journal.pone.0281826)
Supplement: S6 Text — (DOCX) [file pone.0281826.s009.docx]

S6 Text: Study Characteristics – Evidence Tables by Study Design

**Table. Manuscript and demographic data of the five publications of overviews of reviews**

| **Author/year** | **Country** | **Search year** | **Objective^a^** | **Funding** | **Age range** | **Patient demographic** | **Type of cannabis use** | **Recommendations regarding cannabis use in older adults** | **Included systematic reviews and whether they were included in our scoping review** |
| --- | --- | --- | --- | --- | --- | --- | --- | --- | --- |
| Montero-Oleas et al., 2020 ^36^ | Ecuador | 2017 | “…to identify the clinical questions about efficacy of medical cannabis assessed in the scientific literature, as well as to give an overview about their potential benefits and harms.” | Funding reported: Universidad UTE | NR | None | Medical use, overseen by a physician | “In conclusion, the evidence on medical uses of cannabis is broad and highly heterogeneous. However, due to methodological limitations, conclusions were reported as “probably beneficial” and “unclear” in most of the assessed comparisons.” (conclusions apply to all age groups) | **Bega et al., 2014**^176^**:** Excluded due to not meeting criteria for a SR  **Koppel et al., 2014**^177^**:** Excluded due to not meeting criteria for a SR  **Krishnan et al., 2009**^178^**:** Excluded due to not meeting criteria for a SR  **Mucke et al., 2016**^179^**:** Not included in search: published in German  **van den Elsen et al., 2014**^58^**:** Included  **Campbell et al., 2001**^180^**:** Excluded due to not meeting criteria for a SR |
| Bao et al., 2014 ^34^ | China | 2014 | To appraise the evidence level for the use of complementary and alternative medicine on adult cancer pain | Funding Reported: partially supported by the National Natural Science Foundation of China (no. 81273718 and no. 81302961) | NR | Cancer patients | Medical use, overseen by a physician | “Based on all evidence we collected, we could not recommend any CAM interventions for adult cancer pain because of small sample size, high heterogeneity across studies, and high risk of bias for primary studies.” | **Martin-Sanchez et al., 2009**^181^**:** Excluded due to no synthesis of older adult data |
| Hauser et al., 2017 ^32^  (companion of Hauser et al., 2018 ^33^) | Germany | 2017 | To identify potential indications for, but also risks of cannabinoids in pain management and palliative medicine, based on systematic reviews of RCTs and prospective long-term (≥ 6 months) observational studies. | NR | NR | Cancer patients | Medical use, overseen by a physician | “1.) According to the quality criteria of evidence-based medicine, the available evidence for cannabinoids is inadequate for the indications of loss of appetite in patients with cancer or HIV/AIDS, fibromyalgia syndrome, Crohn’s disease, musculoskeletal pain, rheumatoid arthritis, chronic pancreatitis, and cancer pain.  2.) The use of cannabinoids in pain management and palliative medicine should be regarded as individual therapeutic trials, except for chronic neuropathic pain.  3.) Cannabinoid use in pain management and palliative medicine may cause relevant central nervous system (e.g. dizziness) and psychiatric adverse events (e.g. confusion, psychosis).” | **Mucke et al., 2016**^179^**:** Not included in search: published in German  **Whiting et al., 2015**^57^**:** Included |
| Allan et al., 2018 ^35^ | Canada | 2017 | To provide evidence for a medical cannabinoid prescribing guideline...to have clear guidance for prescribers and their patients, as well as to provide adequate information to promote shared, informed decision making. | No funds provided | NR | Cancer patients | Medical use, overseen by a physician | “In cancer pain, the results of two systematic reviews are unclear.” | **Lobos Urbina et al., 2016**^182^**:** Excluded due to not meeting criteria for a SR (rapid review)  **Tateo, 2017**^183^**:** Excluded due to not meeting criteria for a SR |
| Hauser et al., 2018 ^33^  (companion of Hauser et al., 2017 ^32^) | Germany | 2017 | To summarize the efﬁcacy, tolerability and safety of cannabis-based medicines as a treatment for chronic pain (non-cancer and cancer pain) in patients of all ages compared to placebo or other analgesics as assessed by SRs of randomized controlled trials | No funds provided | NR | Cancer patients | Medical use, overseen by a physician | “The available evidence comparing patient outcomes following cannabis-based medicines treatment versus placebo appears insufﬁcient to make well-founded conclusions about the clinical advantage and use of cannabis-based medicines for the management of cancer and non-cancer pain.” | **Mucke et al., 2016**^179^**:** Not included in search: published in German  **Whiting et al., 2015**^57^**:** Included |
| ^a^Minor modifications may have been made to the wording of study objectives to adjust verb tense, etc. Although potentially paraphrased, we have placed objectives in quotes to emphasize that these are based upon the authors’ wording. | | | | | | | | | |

**Table. Manuscript and demographic data of the 22 included systematic reviews of primary studies.**

All included systematic reviews focused strictly on medical cannabis use.

| **Author/year**  **Country**  **Funding** | **Search year**  **Study designs included^a^**  **Types of syntheses conducted** | **Objective^b^** | **Patient demographic^c^** | **Recommendations of the review regarding cannabis use in older adults** | **Numbers of studies for which we extracted data from the systematic review that were included and excluded in our scoping review** | **AMSTAR-2 rating** |
| --- | --- | --- | --- | --- | --- | --- |
| Charernboon et al., 2021 ^37^  Thailand  Funding not reported | 2019  RCTs  Multi- and single-study NSs | “…to examine the effectiveness of cannabinoids in the treatment of dementia covering a range of symptoms including cognition and behavioral and psychological symptoms of dementia” | Dementia of any type | “Overall, limited evidence existed to support the effectiveness of these agents in treating behavioral and psychological symptoms of dementia or cognitive symptoms. However, the safety profile was favorable.” | Included: 4 ^72,86,87,89^  Excluded: 1^f 167^ | Critically low |
| Bahji et al., 2020 ^38^  Canada  Not funded | 2019  SRs, RCTs, Quasi-RCTs or NRSs  MAs | “…to assess the efficacy and acceptability of cannabinoids for the treatment of neuropsychiatric symptoms in individuals with dementia” | Dementia | “At present, the use of cannabinoids in individuals with dementia should still be considered an experimental treatment until more clinical data are available.”  “This systematic review and meta-analysis found consistent evidence that cannabinoids are efficacious for the treatment of neuropsychiatric symptoms associated with dementia and are well-tolerated for use in individuals with dementia. However, our findings were not robust and were particularly vulnerable to the small sample sizes as demonstrated in our sensitivity analyses. Thus, while there is growing neurobiological evidence that cannabinoids may be useful in modulating disease processes in dementia, more evidence is needed before they can be recommended for routine use in clinical practice.” | Included: 8 ^58,72,86,87,89,148,156,158^  Excluded: 1^f 167^ | Low |
| Boland et al., 2020 ^39^  UK  Not funded | 2018  RCTs  MAs, Multi- and single-study NSs | “…to determine the beneficial and adverse effects of cannabinoids compared with placebo or other active agents for the treatment of cancer-related pain in adults from RCTs” | Cancer | “Based on evidence with a low risk of bias, cannabinoids cannot be recommended for the treatment of cancer-related pain.” | Included: 3 ^70,76^  Excluded: 2^d 164,165^ | Low |
| Braud et al., 2020 ^40^  France  Funding not reported | 2019  RCTs  Single-study NSs | “...to assess the effects of palliative and curative interventions on taste recovery in light of recent literature.” | Taste disorder | “Further research is needed to assess the clinical benefit of palliative cannabinoid use in dysgeusic patients.” | Included: 1 ^63^  Excluded: 0 | Critically low |
| Fisher et al., 2020 ^41^  UK  Unclear funding source | 2019  RCTs  MAs, Single-study NSs | “…to provide a comprehensive summary of the evidence from primary RCTs of cannabinoids, cannabis, and cannabis-based medicines in clinical acute and chronic pain management, across the lifespan.” | Cancer | “The current available evidence provides us with no confidence that a defined cannabinoid, cannabis, or cannabis-based medicine product, at a defined dose, using a defined route of administration, reduces pain intensity in any condition, nor do we fully understand the long-term implications of taking cannabinoids, cannabis, and cannabis-based medicines.” | Included: 2 ^70,76^  Excluded: 4^d^ ^164,165,184,185^ | Low |
| Suraev et al., 2020 ^42^  Australia  University and government funded | 2019  Case-series  Single-study NS | “…to synthesise the extant research on cannabinoids as therapeutics for sleep in a manner that informs policy, research priorities, and clinical decision-making” | Parkinson’s disease with sleep disorder | For single study of Parkinson’s disease patients: “This clearly requires further placebo-controlled investigation to identify potentially more effective and safer therapies for patients with this neurodegenerative disease.”  For all ages: “At present, there is limited evidence to support the clinical use of cannabinoid therapies for the treatment of any sleep disorder given the dearth of published research and the moderate-to-high risk of bias identiﬁed within the majority of clinical and preclinical studies completed to-date.” | Included: 1 ^66^ | Critically low |
| Gaisl et al., 2019 ^43^  Switzerland  Not funded | 2018  RCTs  Single-study NSs | 1. “to summarize all available evidence from randomised controlled trials on the efﬁcacy of pharmacotherapy in obstructive sleep apnea, 2. to examine the underlying mechanisms of pharmacotherapy in obstructive sleep apnea, and 3. to ascertain the research activity in this ﬁeld to create a framework for future research on this topic.” | Obstructive sleep apnea | Not reported | Included: 0  Excluded: 1^d 186^ | Critically low |
| Ghasemiesfe et al., 2019 ^44^  USA  Government funded | 2019  NRSs  Single-study NSs | “…to improve the understanding of the association of marijuana use with developing cancers.” | Older general public | There were no recommendations specific to older adults  For all ages: “Low-strength evidence in the present systematic review and meta-analysis suggests that more than 10 years of marijuana use (joint-years were not reported) is associated with the development of testicular germ cell tumor. There was insufficient evidence to support an association between ever having used marijuana and other types of cancer.” | Included: 0  Excluded: 1^d^ ^187^ | Critically low |
| Gouveia et al., 2019 ^45^  Brazil  Government funded | 2018  RCTs  Multi-study NSs | “…to summarize current knowledge about the analgesic profile of newer natural products that are not clinically validated and already used in clinical practice for cancer pain.” | Cancer | Not reported | Included: 0  Excluded: 3^d^ ^165,185,188^ | Critically low |
| Hauser et al., 2019 ^46^  Germany  Funding not reported | 2018  RCTs  MAs, Single-study NSs | “…to assess the efﬁcacy, tolerability, and safety of medical cannabis and cannabis-based medicines (plant-based, synthetic) compared to placebo or conventional drugs for cancer pain in patients of any age.” | Cancer | No evidence-based argument can be made for the use of medical cannabis or nabilone. This absence of evidence should be clearly explained to persons requesting these treatments in jurisdictions where they are allowed, e. g. Canada, Germany and Israel.  The quality of evidence for all outcomes is very low. | Included: 3 ^63,70,76^  Excluded: 2^d^ ^164,165^ | Low |
| Hoch et al., 2019 ^47^  Germany  Government funded | 2018  SRs, RCTs  Multi- and single-study NSs | “…to assess the efficacy and safety of cannabis-based medicines as a treatment of mental disorders” | Alzheimer's disease | THC- and CBD-based medicines were associated with improvements of several symptoms of mental disorders, but not with remission. Side effects can occur, but severe AEs were mentioned in single cases only. The overall confidence in the evidence is low. | Included: 3 ^60,86,87^  Excluded: 1^e 179^ | Low |
| Millar et al., 2019 ^48^  UK  Mixed funding | 2018  RCTs, Case-series  Single-study NSs | “...to comprehensively collate all published data relating to CBD administration in clinical populations to describe the range of CBD doses assessed across different pathological states” | Parkinson’s disease | For Parkinson’s disease: “Results are mixed with Parkinson's studies.”  Generally, for CBD across conditions for all age groups: “However, it is vital to note that no conclusions can be drawn on the efficacy of CBD as larger phase III and conclusive efficacy trials have not been conducted, with exception of epilepsy.” | Included: 1 ^66^  Excluded: 3^d,f^ ^189–191^ | Critically low |
| Ruthirakuhan et al., 2019 ^49^  Canada  Government funded | 2018  RCTs, Quasi-RCTs or NRSs  MAs | “…to investigate he efficacy of cannabinoids on agitation and aggression in patients with Alzheimer's disease” | Alzheimer's disease | The results of this meta-analysis provide little evidence of efficacy for the effect of cannabinoids on agitation in Alzheimer’s disease. While our findings suggest that THC for the treatment of agitation has been consistently negative, results with synthetic cannabinoids are inconclusive due to substantial heterogeneity. | Included: 4 ^86,87,89,156^  Excluded: 2^f 166,167^ | Critically low |
| Wang et al., 2019 ^50^  China  Government funded | 2017  RCTs  MAs | “…to assess the positive and negative effects of cannabinoid in the treatment of cancer cachexia” | Cancer with cachexia | “Our analysis showed cannabinoid is effective in increasing appetite in cancer patients. However, it declines the quality of life, which may be due to the side effects of cannabinoid.”  “The results show that the clinical application of cannabinoid in the treatment of patients with CCA [cancer cachexia] may be at the expense of declining QOL.” | Included: 3 ^63,64,76^  Excluded: 0 | Critically low |
| Mucke et al., 2018 ^51^  Germany  Government funded | 2017  RCTs  MAs, Single-study NSs | “…to evaluate the efﬁcacy, tolerability, and safety of cannabinoids as an adjunct or complementary therapy in palliative medicine” | Cancer (one set of syntheses)  Alzheimer’s disease (second set of syntheses) | Following the GRADE methodology, no recommendations can be made for the use of cannabinoids in palliative care treatment for cancer, HIV/AIDS, or dementia. | Included: 5 ^63,64,74,76,89^  Excluded: 1^d^ ^165^ | Critically low |
| Goldenberg et al., 2017 ^52^  USA  Not funded | 2015  RCTs  Multi-study NSs | “…to evaluate the relationship between cannabis and cannabinoids used for medical conditions and health-related quality of life” | Cancer | Our systematic review and meta-analysis of studies evaluating cannabis or cannabinoids for medical conditions does not reveal a main effect on health-related quality of life. However, there are speciﬁc disease states, such as pain, that separate from the overall trend. | Included: 1 ^64^  Excluded: 1^d 165^ | Critically low |
| Lim et al., 2017 ^53^  Singapore  Government funded | 2017  RCTs  Multi- and single-study NSs | “…to provide an in-depth evaluation of the efficacy of medical cannabinoids by appraising the quality of evidences from clinical studies across a broad range of neurodegenerative disorders and psychiatric conditions.” | Alzheimer's, dementia, and Parkinson's disease patients (separately) | Although results were inconsistent, there appears to be some low-quality evidence of cannabinoids for anorexia nervosa, anxiety, post-traumatic stress disorder, psychotic symptoms, agitation in Alzheimer’s disease and dementia, Huntington’s disease, and Tourette syndrome, and dyskinesia in Parkinson’s disease. | Included: 6 ^65,66,83,86,87,89^  Excluded: 1^f 167^ | Critically low |
| Nielsen et al., 2017 ^54^  Australia  Government funded | 2015  RCTs, Quasi-RCTs or NRSs  Multi- and single-study NSs | “…to examine the strength of existing evidence demonstrating the opioid-sparing effect of cannabinoids in the context of analgesia.” | Cancer patients with pain | Not stated specifically for cancer pain. Generally, they said that "the findings from clinical trials are inconsistent, with some studies found to have important limitations such as a lack of placebo control. An opioid-sparing effect of cannabinoids in chronic pain patients was observed in only one very-low-quality clinical study." | Included: 1 ^76^  Excluded: 3^d,e,f 165,193,194^ | Critically low |
| Nugent et al., 2017 ^195^  USA  Government funded | 2017  RCTs  Multi-study NSs | “…to assess the efﬁcacy of cannabis for treating chronic pain and to provide a broad overview of the short- and long-term physical and mental health effects of cannabis use in chronic pain and general patient populations” | Cancer patients with pain | Insufficient evidence, given the small number of studies and their methodological limitations. | Included: 1 ^76^  Excluded: 2^d^ ^165,185^ | Critically low |
| van den Beuken-van Everdingen et al., 2017 ^56^  Netherlands  Funding not reported | 2014  SRs, RCTs  Multi- and single-study NSs | “…to update our guidelines for the treatment of pain in patients with cancer, we performed a systematic review on the use of adjuvant analgesics in pain in cancer” | Cancer patients with pain | Consider the use of cannabinoids (combination [THC/CBD]) in patients with cancer when pain cannot be treated sufﬁciently with opioids and/or the other adjuvant analgesics  GRADE = 2D (weak recommendation based on expert opinion) | Included: 0  Excluded: 2^d 180,196^ | Low |
| Whiting et al., 2015 ^57^  UK  Government funded | 2015  RCTs  MAs | “…to evaluate the evidence for the benefits and adverse events of medical cannabinoids across a broad range of indications” | Cancer | Based on the GRADE approach, there was moderate-quality evidence to suggest that cannabinoids maybe beneficial for the treatment of chronic neuropathic or cancer pain (smoked THC and nabiximols) and spasticity due to MS (nabiximols, nabilone, THC/CBD capsules, and dronabinol). | Included: 1 ^76^  Excluded: 1^d^ ^165^ | Moderate |
| van den Elsen et al., 2014 ^58^  Netherlands  Government funded | 2013  RCTs  Multi- and single-study NSs | “…to provide broader evidence on the safety and efﬁcacy of medical cannabinoids in older subjects, independent of the reasons for prescription or the patients’ cognitive status” | Patients with dementia, Parkinson's disease, chemotherapy-induced nausea and vomiting, or chronic obstructive pulmonary disease | Our review shows that there is a lack of evidence concerning the use of cannabinoids speciﬁcally in older patients, resulting in scarcity of data to guide treatment decisions. | Included: 3 ^65,80,89^  Excluded: 1^f^ ^167^ | Critically low |
| ^a^Study designs listed are those that were included in the systematic review and that met our age-related inclusion criteria for “older adults” and for which data were extracted. Studies with other designs may have also been included in the review, but they did not meet our older adult inclusion criteria.  ^b^Minor modifications may have been made to the wording of study objectives to adjust verb tense, etc. Although potentially paraphrased, we have placed objectives in quotes to emphasize that these are based upon the authors’ wording.  ^c^Reported is the patient demographic of primary studies included in the systematic review that met our age-related inclusion criteria for “older adults” and for which data were extracted. Primary studies with other patient demographics that did not meet our inclusion criteria may have also been included in the systematic review.  ^d^Less than 80% of participants over 50 years of age, and did not report findings specific to older adults  ^e^Excluded due to non-English or -French language  ^f^Excluded due to being published as a letter, commentary, or conference abstract  AMSTAR = Assessing the Methodological Quality of Systematic Reviews; CCA = cancer cachexia; CBD = cannabidiol; GRADE = Grading of Recommendations, Assessment, Development and Evaluations; HIV/AIDS = human immunodeficiency virus/acquired immunodeficiency syndrome; MAs = meta-analyses; NRSs = non-randomized studies; NSs = narrative summaries; RCTs = randomized controlled trials; SRs = systematic reviews; THC = delta-9-tetrahydrocannabinol | | | | | | |

**Table: Manuscript and demographic data of the 36 included RCTs.**

The residential status of trial participants was reported in only four included RCTs (see data spreadsheets). All but three RCTs compared cannabis products to placebo. Three RCTs compared a cannabis product to an antiemetic active comparator. One RCT reported two separate trials. All studies compared cannabis product(s) to placebo, except where noted^74,75,77^.

| **RCT**  **Country**  **Funding** | **NCT# (if applicable)** | **Patient condition**  **RCT design** | **Objective^a^** | **Older adult sample size**  **Proportion male (total or treatment group)**  **Race (total or treatment group)** | **Cannabis intervention and daily dose(s)** | **Follow-up time (time on cannabis)** |
| --- | --- | --- | --- | --- | --- | --- |
| Martins de Faria et al., 2020 ^69^  Brazil  Non-industry funding | NR | Parkinson’s disease  Crossover | “…to evaluate the impacts of acute CBD administration at a dose of 300 mg on anxiety measures and tremors induced by a Simulated Public Speaking Test (SPST) in individuals with Parkinson's Disease.” | n = 24  92%  NR | Synthetic CBD powder dissolved in corn oil and put in capsules  300 mg CBD one-time experimental dose | ~2 hours |
| Peball et al., 2020 ^79^  Austria  Mixed funding | NCT03769896 and EudraCT (2017-000192-86) | Parkinson’s disease  Parallel - enriched-enrollment randomized-withdrawal (EERW) | “…to assess the efﬁcacy and safety of nabilone a synthetic tetrahydrocannabinol analogue, as a treatment for non-motor symptoms in Parkinson’s disease” | 38  60%  100% Caucasian | Nabilone  Mean = 0.91 mg CBD (possible range: 0.25 - 2 mg) | 4 weeks |
| Xu et al., 2020 ^91^  USA  Not funded | NR | Peripheral neuropathy  Crossover | “…to investigate the effectiveness of topical CBD-enriched emu oil in the symptomatic treatment of chronic pain from peripheral neuropathy of the lower extremities” | n = 29 (all ages)  62.1% (all ages  NR | Synthetic CBD topical cream  83 mg CBD/fl. oz. up to 4 times daily | 4 weeks |
| Herrmann et al. 2019 ^72^  Canada  Non-industry funding | NCT02351882 | Alzheimer's disease/dementia  Crossover | “…to investigate the efficacy and safety of nabilone for agitation in patients with moderate-to-severe Alzheimer's disease” | n = 38  77%  NR | Nabilone  Up to 1–2 mg THC, as tolerated | 6 weeks on nabilone |
| Riva et al., 2019 ^81^  Italy  Non-industry funding | NCT01776970 | Amyotrophic lateral sclerosis  Parallel | “…to explore the safety and effects of a standardised oromucosal spray (nabiximols) containing a defined combination of THC and cannabidiol on spasticity related to motor neuron disease.” | n = 59 (all ages)  57.6% (all ages)  NR | Sativex  2.7 mg THC + 2.5 mg CBD to 32.4 mg THC + 30 mg CBD | 6 weeks (4 weeks on stable dose) |
| Abdallah et al., 2018 ^59^  Canada  Mixed funding | NCT03060993 | Chronic obstructive pulmonary disorder  Crossover | “…to test the hypothesis that inhaled vaporized cannabis would alleviate exertional breathlessness and improve exercise endurance by enhancing static and dynamic airway function in chronic obstructive pulmonary disorder  .” | n = 16  62.5%  NR | THC:CBD extracts  Arm 1: 35 mg THC + < 2 mg CBD  Arm 2: < 0.6 mg THC + < 2 mg CBD | Immediate |
| Turcott et al., 2018 ^85^  Mexico  Not funded | NCT02802540 | Cancer  Parallel | “…to evaluate the effect of nabilone vs. placebo in lung cancer patients diagnosed with anorexia using the AC/S of the Functional Assessment of Anorexia Cachexia Therapy (FAACT) tool” | n = 47  21.4%  NR | Nabilone  1 mg THC | 8 weeks |
| Fallon et al., 2017a ^70^  Multi-country  Industry funding | NCT01361607 | Cancer  Parallel | “…to assess the analgesic efficacy of adjunctive Sativex^®^ in advanced cancer patients with chronic pain unalleviated by optimized opioid therapy.” | n = 399  50.9%  96% Caucasian, 1.5% Black, 0.5% Asian, 1.5% Other | Sativex^®^  20 mg THC + 18.5 mg CBD | 5 weeks |
| Fallon et al., 2017b ^70^  Multi-country  Industry funding | NCT01424566 | Cancer  Parallel | “…to assess the analgesic efficacy of adjunctive Sativex^®^ in advanced cancer patients with chronic pain unalleviated by optimized opioid therapy.” | n = 206  57.3%  95.1% Caucasian, 0% Black, 1.9% Asian, 0% Other | Sativex^®^  17.6 mg THC + 16.3 mg CBD | 5 weeks |
| van den Elsen et al., 2017 ^88^  The Netherlands  Non-industry funding | NCT01302340 | Alzheimer's disease/dementia  Crossover | “…to evaluate the effects of THC on mobility in dementia patients” | n = 18  83%  94% Caucasian, 6% Asian | Namisol  Arm 1: 1.5 mg THC  Arm 2: 3.0 mg THC | 12 weeks |
| Cote et al., 2016 ^68^  Canada  Non-industry funding | NR | Cancer  Parallel | “…to assess the efficacy of nabilone in improving quality of life, especially pain, appetite, and nausea, of patients treated by radiotherapy for head and neck squamous cell carcinomas” | n = 56  82.1%  NR | Nabilone  2 mg or less THC | 9–11 weeks |
| Jadoon et al., 2016 ^73^  UK  Non-industry funding | NCT01217112 | Diabetes  Parallel | “…to investigate the effects of CBD and THC on dyslipidemia and glycemic control in subjects with type 2 diabetes” | n = 62  68%  NR | D9-Tetrahydrocannabivarin (THCV), CBD, and combinations of THCV and CBD extracts  Arm 1: 200 mg CBD  Arm 2: 10 mg THCV  Arm 3: 10 mg THCV + 10 mg CBD  Arm 4: 10 mg THCV + 200 mg CBD | 13 weeks |
| Ahmed et al., 2015 ^60^  The Netherlands  Non-industry funding | NCT01302340 | Alzheimer's disease/dementia  Crossover | “…to evaluate the safety, pharmacodynamics, and pharmacokinetics of multiple oral doses of THC in older persons with dementia” | n = 10  70%  90% Caucasian, 10% Other | Namisol  Period 1: 1.5 mg THC  Period 2: 3.0 mg THC | 18 days out of 12 weeks |
| van den Elsen et al., 2015a ^86^  The Netherlands  Non-industry funding | NCT01302340 | Alzheimer's disease/dementia  Crossover | “…to assess the efficacy and safety of oral THC in the treatment of neuropsychiatric symptoms in dementia.” | n = 22  68%  95.5% Caucasian, 4.5% Other | Namisol  Arm 1: 1.5 mg THC  Arm 2: 3.0 mg THC | 18 days out of 12 weeks |
| van den Elsen et al., 2015b ^87^  The Netherlands  Non-industry funding | NCT01608217 | Alzheimer's disease/dementia  Parallel | “…to study the efficacy and safety of low-dose oral THC in the treatment of dementia-related neuropsychiatric symptoms” | n = 50  50%  100% Caucasian | Namisol  4.5 mg THC | 3 weeks |
| Wallace et al., 2015 ^90^  USA  Non-industry funding | NCT00781001 | Diabetic neuropathy  Immediate - crossover | “…to evaluate the effects of low, medium, and high-dose inhaled vaporized cannabis on the pain and hyperalgesia of diabetic neuropathy” | n = 16  56%  50% Black, 44% Caucasian, 6% Hispanic | THC extracts  Arm 1: 4 mg THC (once in lab)  Arm 2: 16 mg THC (once in lab)  Arm 3: 28 mg THC (once in lab) | Immediate |
| Ahmed et al., 2014 ^19^  The Netherlands  Non-industry funding | NCT01740960 | Healthy older adults  Crossover | “…to assess the safety and tolerability of three oral doses of THC in healthy older subjects, to evaluate the pharmacokinetics of THC in older people, and to investigate the relationship between the drug's pharmacodynamic effects and the plasma concentrations of THC and its active metabolites.” | n = 12  50%  100% Caucasian | Namisol  Arm 1: 3 mg  Arm 2: 5 mg  Arm 3: 6.5 mg | Immediate |
| Chagas et al., 2014 ^66^  Brazil  Not funded | NR | Parkinson's disease  Parallel | “…to assess the effects of CBD in Parkinson's disease globally, including neurological assessments of motor and functional symptoms, a psychiatric assessment and complementary tests (brain-derived neurotrophic factor plasma levels and H1-MRS).” | n = 21  71.4%  NR | Unclear (CBD powder)  Arm 1: 75 mg CBD  Arm 2: 300 mg CBD | 6 weeks |
| Notcutt et al., 2012 ^78^  UK  Industry funding | NR | Multiple sclerosis  Parallel | “…to evaluate the maintenance of efficacy of Sativex^®^ in subjects who have gained long-term symptomatic relief of spasticity in multiple sclerosis, and to assess the impact of sudden medicine withdrawal.” | n = 36  41.6%  100% Caucasian | Sativex^®^  19.7 mg THC + 18.3 mg CBD | 4 weeks |
| Pickering et al., 2011 ^80^  UK  Non-industry funding | NR | COPD  Immediate - crossover | “…to explore the effect of cannabinoids on ventilation and breathlessness in COPD patients” | n = 9  66.6%  NR | Sativex^®^  7.6 mg THC + 7 mg CBD | Immediate |
| Zadikoff et al., 2011 ^92^  Canada  Non-industry funding | NCT00418925 | Cervical dystonia  Crossover | “…to determine the efficacy of dronabinol in cervical dystonia” | n = 9  0%  NR | Dronabinol  2.5 to 15 mg THC | 4 weeks |
| Brisbois et al., 2010 ^63^  Canada  Non-industry funding | NCT00316563 | Cancer  Crossover | “…to determine if THC can improve taste and smell (chemosensory) perception as well as appetite, caloric intake, and quality of life for cancer patients with chemosensory alterations” | n = 46  57.1%  NR | Dronabinol  5 mg or more as tolerated | 18 days |
| Johnson et al., 2010 ^76^  Multi-country  Industry funding | NR | Cancer  Parallel | “…to compare the efficacy of a THC:CBD extract, a nonopioid analgesic endocannabinoid system modulator, and a THC extract, with placebo, in relieving pain in patients with advanced cancer” | n = 177  54%  98% Caucasian, 2% Other | Sativex^®^ and THC extract  Arm 1: 23.6 mg THC + 21.9 mg CBD  Arm 2: 22.5 mg THC | 2 weeks |
| Selvarajah et al., 2010 ^82^  UK  Non-industry funding | NR | Diabetic neuropathy  Parallel | “…to assess the efficacy of Sativex^®^ as adjuvant treatment in painful diabetic peripheral neuropathy” | NR  63%  NR | Sativex^®^  18.9 mg THC + 17.5 mg CBD | 12 weeks |
| Conte et al., 2009 ^67^  Italy  Unclear funding | NR | Multiple sclerosis  Parallel | “…to acquire objective neurophysiological information on how cannabinoids modulate human pain perception in multiple sclerosis patients” | n = 11  33.3%  NR | Sativex^®^  21.6 mg THC + 20 mg CBD | 3 weeks |
| Beaulieu et al., 2006 ^61^  Canada  Mixed funding | NR | Surgery patients  Crossover | “…to explore the effects of nabilone on postoperative pain.” | n = 41  19.5%  NR | Nabilone  Arm 1: 6 mg  Arm 2: 3 mg | 24 hours |
| Strasser et al., 2006 ^62^  Multi-country  Non-industry funding | NR | Cancer  Parallel | “…to compare the effects of cannabis extract, THC, and placebo on appetite and quality of life in patients with cancer-related anorexia-cachexia syndrome” | n = 289  54.3%  NR | THC:CBD and THC extracts  Arm 1: 5 mg THC + 2 mg CBD  Arm 2: 5 mg THC | 6 weeks |
| Tomida et al., 2006 ^84^  UK  Non-industry funding | NR | Ocular hypertension  Parallel | “…to assess the effect on intraocular pressure and the safety and tolerability of oromucosal administration of a low dose of THC and cannabidiol CBD” | n = 6  100%  NR | THC and CBD extracts  Arm 1: 5 mg THC  Arm 2: 20 mg CBD  Arm 3: 40 mg CBD | 4 weeks |
| Blake et al., 2005 ^62^  UK  Industry funding | NR | Rheumatoid arthritis  Crossover | “…to assess the efficacy of a cannabis-based medicine in the treatment of pain due to rheumatoid arthritis” | n = 58  21%  NR | Sativex^®^  14.9 mg THC + 13.5 mg CBD | 5 weeks |
| Carroll et al., 2004 ^65^  UK  Funding not reported | NR | Parkinson's disease  Parallel | “…to examine the effect of cannabis extract on the severity and duration of dyskinesia in Parkinson's disease” | n = 19  63%  NR | THC:CBD extract (Cannador)  0.15 mg/kg THC + 0.08 mg/kg CBD | 4 weeks |
| Jatoi et al., 2002a ^74^  USA  Non-industry funding | NR | Cancer  Crossover | “…to determine whether Dronabinol administered alone or with megestrol acetate was more, less, or equal in efficacy to single-agent megestrol acetate for palliating cancer-associated anorexia.” | n = 469  66%  NR | Dronabinol vs megestrol acetate  5 mg THC | 4 weeks |
| Jatoi et al., 2002b ^75^  USA  Non-industry funding | NR | Cancer  Immediate - crossover | “…to examine the orexigenic effects of megestrol acetate, the cannabinoid dronabinol, versus the combination of both for the treatment of cancer-associated anorexia and weight loss” | n = 85  61.2%  NR | Dronabinol vs megestrol acetate  5 mg THC | 4 weeks |
| Sieradzan et al., 2001 ^83^  UK  Non-industry funding | NR | Parkinson's disease  Crossover | “…to assess whether nabilone could reduce GABA reuptake in the globus pallidus by activation of cannabinoid receptors and reduce levodopa-induced dyskinesia in patients with Parkinson's disease” | n = 9  44%  NR | Nabilone  0.06 mg/kg THC | Immediate |
| Volicer et al., 1997 ^89^  USA  Mixed funding | NR | Alzheimer's disease/dementia  Parallel | “…to investigate the effect of dronabinol on food refusal in dementia patients and to assess the effect of dronabinol on disturbed behavior in Alzheimer's disease patients” | n = 15  91.6%  NR | Dronabinol  5 mg THC | 6 weeks |
| Niiranen et al., 1985 ^77^  Finland  Funding not reported | NR | Cancer  Parallel | “…to evaluate the efficacy and safety of nabilone compared with prochlorperazine in the prophylaxis and treatment of cancer chemotherapy-induced emesis.” | n = 24  83%  NR | Nabilone vs prochlorperazine  2 mg THC | NR |
| Frytak et al., 1979 ^71^  USA  Funding not reported | NR | Cancer  Immediate - crossover | “…to compare the antiemetic effects and side-effects of THC with those of prochlorperazine in cancer patients undergoing chemotherapy” | n = 98  60.3%  NR | THC extract  15 mg THC | 4 days |
| ^a^Minor modifications may have been made to the wording of study objectives to adjust verb tense, etc. Although potentially paraphrased, we have placed objectives in quotes to emphasize that these are based upon the authors’ wording.  CBD = cannabidiol; EERW = enriched-enrollment randomized-withdrawal; NR = not reported; THC = delta-9-tetrahydrocannabinol | | | | | | |

**Table. Manuscript and demographic data of the 72 included non-randomized studies**

One publication reported three separate non-randomized studies^153^.

| **NRS**  **Country**  **Funding** | **Patient condition** | **Study design**  **Data source, if applicable** | **Objective^a^** | **Older adult sample size**  **Proportion male (total or exposed/case group)**  **Race (total or exposed/case group)** | **Marital status**  **Employment status**  **Primary residence** | **Type of cannabis use** | **Cannabis product or definition of current use**  **Daily dose, if reported** |
| --- | --- | --- | --- | --- | --- | --- | --- |
| Choi et al., 2021a ^5^  USA  Funding not reported | Older general public | Sequential^b^  National Surveys on Drug Use and Health (NSDUH), 2015–17 | “…to examine: (1) correlates of use-to-nonuse (use 13-24 months ago, but no past-year use), initiation/reinitiation (no use 13–24 months ago, but past-year use), and continued use over a 24-month period; and (2) associations of past-year marijuana use disorder with use/nonuse among older adults.” | n = 2,304  59.1%  Initiators/ reinitiators:  67.6% non-Hispanic White, 18.3% non-Hispanic Black, 11.0% Hispanic, 3.1% all other  Those who continued using:  78.2% non-Hispanic White, 12.3% non-Hispanic Black, 5.0% Hispanic, 4.6% all other | Initiators/ reinitiators:  43.3% married or partnered, 6.0% widowed, 37.1% divorced or separated, 13.7% never married  49.2% worked full- or part-time in past year  Those who continued using:  49% married or partnered, 6.7% widowed, 29.1% divorced or separated, 15.2% never married  57.5% worked full- or part-time in past year | Medical, self-medicated; Non-medical/ recreational | Any past-year use  NR |
| Choi et al., 2021b ^104^  USA  Not funded | Older general public | Sequential  NSDUH, 2015–18 | “…to examine rates and correlates of dual cannabis and prescription pain reliever use and misuse among U.S. individuals aged 50+ who reported past-year cannabis use.” | n = 37,861  46.8%  Exposed group:  75.7% non-Hispanic White, 13.0% non-Hispanic Black, 6.5% Hispanic, 4.8% other | Exposed group:  48.0% married, 6.8% widowed, 30.4% divorced or separated, 14.9% never married  40.8% worked full- or part-time in past year | Medical, self-medicated, Non-medical/ recreational | Past-year use  NR |
| Choi et al., 2021c ^108^  USA  Not funded | Older general public | Retrospective cohort  American Association of Poison Control Centers’ National Poison Data System, 2009–19 | “…to examine: (1) trends in cannabis forms that constituted poison control centre cases over the 11-year period; (2) demographic and clinical factors associated with different cannabis forms; and (3) associations between cannabis forms and medical outcomes.” | n = 5,201  53.5%  NR | NR  NR  NR | Medical, self-medicated, Non-medical/ recreational | NR  NR |
| Croker et al., 2021 ^110^  USA  Non-industry funding | Individuals using medical cannabis (medical use only or mixed-purpose use) | Cross-sectional^c^  Study-specific survey | “…to assess health-related outcomes associated with medical cannabis use among older patients in Colorado and Illinois enrolled in their home state’s medical cannabis program.” | n = 139  51%  88% Caucasian, 12% non-Caucasian | 69% married  84% retired  100% community living | Medical, overseen by a physician; Medical, self-medicated | Past-week use  NR |
| Denduluri et al., 2021 ^112^  USA  Funding not reported | Joint replacement | Retrospective cohort | “Use urine toxicology screening dataset to assess the recent prevalence and trends of cannabinoid use relative to opioid use and also to identify demographic characteristics of patients who screened positive for cannabinoids (THC) to detect an association between preoperative cannabinoid use and postoperative morbidity.” | n = 1,778  94.4%  NR | NR  NR  NR | Medical, self-medicated, Non-medical/ recreational | NR  NR |
| Ramadan et al., 2021 ^144^  USA  Funding not reported | Older general public | Sequential  NSDUH, 2002–14 | “…to examine the association between both medical and recreational marijuana use and different type of nonmedical prescription opioids use among older adults aged 50 years or older.” | n = 75,949  45.1%  Exposed group:  75.1% non-Hispanic White, 13.3% non-Hispanic Black, 4.4% Hispanic, 7.2% other | Exposed group:  46.3% married, 5.7% widowed, 34.4% divorced or separated, 13.6% never married | Medical, self-medicated, Non-medical/ recreational | Past-year use  NR |
| Sznitman et al., 2021 ^151^  Israel  Non-industry funding | Chronic non-cancer pain | Cross-sectional  Study-specific survey | …to assess “the relationship between long-term medical cannabis use and cognitive function in a sample of middle-aged and old chronic pain patients.” | n = 125  48.8%  NR | NR  NR  NR | Medical, self-medicated | Daily/almost daily use  Mean monthly dose 31.1 ± 12.1 g |
| Vozoris et al., 2021 ^155^  Canada  Mixed funding | Chronic obstructive pulmonary disease | Retrospective cohort  ICES and multiple Ontario healthcare administrative databases, 2006–2016 | “…to evaluate the association between new prescription synthetic oral cannabinoid drug use and respiratory-related morbidity and mortality among older adults with chronic obstructive pulmonary disease” | n = 4,212  39.3%  NR | Exposed group:  10.6% long-term care residence, 17.0% rural residence | Medical, self-medicated | Daily/almost daily use  NR |
| Whitfield et al., 2021 ^157^  Australia, France, Germany, Switzerland, UK, USA  Non-industry funding | Alcohol use | Case-control | to identify “potential clinical and phenotype factors that alter alcohol related cirrhosis risk including beverage preference, other substance use, family history, obesity, and diabetes.” | NR  NR  NR | NR  NR  NR | NR | NR  NR |
| Abbs et al., 2020 ^93^  USA  Non-industry funding | Homelessness | Prospective cohort | “…to examine the prevalence of and risk of falling in a cohort of older homeless adults” | n = 350  77.1%  79.7% Black, 10.9% Caucasian, 4.6% Hispanic, 4.9% other | NR  NR  100% homeless | Non-medical/ recreational | Past-six-month use  NR |
| Benitez et al., 2020 ^100^  USA  Funding not reported | Older general public | Cross-sectional  National Epidemiologic Survey on Alcohol and Related Conditions III (NESARC-III), 2012–13 | “…to test whether marijuana use was associated with self-reported cognition in adults ages ≥ 50 using data from the NESARC-III” | n = 14,678  44%  Exposed group:  73.8% non-Hispanic White, 15.3% non-Hispanic Black, 6.2% Hispanic, 0.8% non-Hispanic Asian, 3.9% Indigenous | Exposed group:  43.7% married/ cohabiting, 43.7% widowed/separated/ divorced, 12.6% never married  42.1% full- or part-time employment  100% community living | Medical, self-medicated, Non-medical/ recreational | Past-year use  NR |
| Fujii et al., 2020 ^114^  USA  Funding not reported | Trauma | Retrospective cohort  Natividad Medical Center Level II Trauma Center Registry, 2014–17 | “…to determine if positive marijuana toxicology screen is associated with worse outcomes following trauma”, including “higher mortality, higher injury severity, and longer length of stay.” | n = 112  NR  NR | NR  NR  100% community living | Non-medical/ recreational | NR  NR |
| Kwok et al., 2020 ^125^  UK  Mixed funding | Percutaneous coronary intervention | Retrospective cohort  National Inpatient Sample, 2004–14 | “…to evaluate the rate and trend of cannabis misuse and in-hospital outcomes for patients who misuse cannabis in a national cohort and to understand the impact of cannabis misuse among patients who undergo percutaneous coronary intervention” | NR  NR  NR | NR  NR  NR | Non-medical/ recreational | NR  NR |
| Li et al., 2020 ^128^  USA  Non-industry funding | Older general public | Sequential  Fatality Analysis Reporting System (FARS), 2011–16, and the National Roadside Survey of Drug Use by Drivers (NRS), 2013–14 | “…to assess the association of marijuana use with the odds of prescription opioid use based on toxicological testing data from two US national samples of drivers.” | n = 5,189  NR  NR | NR  NR  NR | NR | NR  NR |
| Linden-Carmichael et al., 2020 ^130^  USA  Non-industry | Alcohol use | Cross-sectional  Population Assessment of Tobacco and Health (PATH) study, 2013–14 | “This study aimed to (1) identify whether co-occurring use was linked with greater risk for prescription drug misuse across ages 18–60 and to determine ages at which this association was strongest, (2) determine whether associations remain after controlling for pain severity, and (3) test for gender differences.” | NR  NR  NR | NR  NR  NR | Non-medical/ recreational | Past-year use  NR |
| Matson et al., 2020 ^135^  USA  Non-industry funding | Older general public | Retrospective cohort  Kaiser Permanente Washington electronic health record data and insurance claims, 2015–16 | “…to examine the association between frequency of cannabis or other drug use and subsequent utilization of acute care, among patients visiting primary care clinics” | n = 33,367  NR  NR | NR  NR  NR | Medical, self-medicated, Non-medical/ recreational | Past-year use  NR |
| Mousa et al., 2020 ^138^  Canada  Funding not reported | Cancer | Cross-sectional  Study-specific survey | To evaluate “the prevalence and predictors of cannabis use, patients’ usage habits, perceived benefits, and opinions on cannabis in patients with advanced prostate cancer.” | n = 222  NR  Exposed group:  15% Afro-Caribbean, 8% Arab, 31% Eastern European, 38% Western European, 8% other | Exposed group:  23% single, 62% married, 8% divorced or separated, 8% widowed  45% full-time employment, 15% part-time employment, 8% retired due to disability, 23% retired due to age, 0% unemployed | Medical, self-medicated, Non-medical/ recreational | Past-six-month use  NR |
| Press et al., 2020 ^143^  USA  Mixed funding | Older general public | Cross-sectional  Baseline data from a prospective cohort study, 2013–18 | “…to examine the association between cigarette smoking, other tobacco use, and marijuana use on serum prostate-specific antigen levels” | n = 644  100%  100% Black | NR  NR  NR | NR | NR  NR |
| Sznitman et al., 2020 ^150^  Israel  Non-industry | Chronic non-cancer pain | Cross-sectional  Study-specific survey | “The aim of the current study was to examine the associations between use of whole plant cannabis and sleep problems among chronic pain patients.” | n = 128  49.2%  NR | NR  NR  100% community living | Medical, self-medicated | Past-week use  Mean monthly dose 31.2 ± 12.2 g |
| YorkWilliams et al., 2020 ^159^  USA  Non-industry funding | Sedentary lifestyle | Prospective cohort (as part of an RCT on exercise) | to examine “whether cannabis use impacted results of an intervention to increase physical activity in sedentary adults aged 60 and over.” | n = 151  37.7%  Exposed group:  88.5% Caucasian, 92.3% not Hispanic or Latino | Exposed group:  26.9% full-time employment | NR | Past-year use  Reported average daily amount used:  < 0.25 gram, n = 20; 0.25 gram, n = 4; 0.5 gram, n = 2; 1 gram, n = 0; >1 gram, n = 2 |
| Airagnes et al., 2019 ^96^  France  Mixed funding | Older general public | Prospective cohort | to search “for an increased risk of job loss according to substance use over 3 years” and to examine “whether substance use associations persist within different subgroups defined based on sociodemographic and occupational factors to identify those at greater risk for job loss.” | n = 6,014  NR  NR | NR  100% employed at baseline  NR | NR | Past-year use  NR |
| DiGuiseppi et al., 2019 ^113^  USA  Mixed funding | Older general public | Cross-sectional  Baseline data from a prospective cohort study, 2015–17 | “This study aims to describe the current epidemiology of cannabis use among drivers aged 65–79 years in Colorado and to examine its relationship to driving outcomes” | n = 598  48.5%  Exposed group:  96.1% not Hispanic or Latino, 3.9% Hispanic or Latino | Exposed group:  67.9% married or living with partner, 32.1% never married, divorced, separated, or widowed  61.% did not work for pay in past month | NR | Past-year use  NR |
| Han et al., 2019a ^117^  USA  Non-industry funding | Prescription opioid use | Sequential  NSDUH, 2015–16 | “…to investigate recent prescription opioid misuse by middle-aged and older adults, using revised terminology for "misuse" in the National Survey on Drug Use and Health (NSDUH) 2015 and 2016.” | n = 17,517  46.9%  73.2% Caucasian, 10.3% Black, 10.2% Hispanic, 4% Asian, 2.2% other | 61.9% married, 12.3% widowed, 18.2% divorced or separated, 7.6% never married  NR  NR | NR | Past-year cannabis use |
| Han et al., 2019b ^115^  USA  Non-industry funding | Alcohol use | Sequential  NSDUH, 2015–17 | “…to examine the most current national data on binge drinking to determine the recent prevalence of binge drinking among adults aged 65 years and older and identify sociodemographic and health-related factors regarding the risk of binge drinking.” | n = 10,927  44.7%  77.4% non-Hispanic White, 9.0% non-Hispanic Black, 8.2% Hispanic, 3.4% non-Hispanic Asian, 2.1% other | 59.9% married, 21.1% widowed, 14.6% divorced or separated, 4.5% never married  NR  NR | NR | Past-month use  NR |
| Howard et al., 2019 ^121^  USA  Not funded | Anorexia due to severe illness (and hospitalized) | Retrospective cohort | “…to analyze the effectiveness and safety of the appetite-stimulating medications available on formulary (dronabinol, mirtazapine, and megestrol) at a community teaching hospital initiated in an inpatient population.” | n = 38  34%  80% Caucasian, 20% Black, 0% Other | NR  NR  100% hospitalized | Medical, overseen by a physician | Dronabinol vs megestrol acetate vs mirtazapine |
| Jennings et al., 2019 ^122^  USA  Not funded | Joint replacement | Retrospective cohort | “…to report outcomes and complications in patients self-reporting cannabis use and undergoing primary knee replacement, but not using tobacco, opioids within 90 days, or illicit drug abuse.” | n = 142  67.6%  NR | NR  NR  NR | NR | Any self-reported cannabis use  NR |
| Lum et al., 2019 ^132^  USA  Non-industry funding | Older general public | Cross-sectional  Study-specific survey | “…to describe the patterns of marijuana use during later life for those who have access to recreational and medical sources of marijuana” | n = 196  35%  NR | 66% partnered, 16% divorced or separated, 13% widowed, 5% never married  87% retired  NR | Medical, overseen by a physician; Medical, self-medicated; Non-medical/ recreational | Past-year medical cannabis use |
| Neilson et al., 2019 ^139^  Canada  Non-industry funding | Older general public | Sequential  Canadian Community Health Survey, 2011–12 | “…to examine the association between use of cannabis and leisure-time sedentary behaviour using data from the 2011–2012 Canadian Community Health Survey” | NR  NR  NR | NR  NR  NR | NR | Any past-year use |
| Taha et al., 2019 ^152^  Israel  Funding not reported | Cancer | Retrospective cohort | “…to evaluate the clinical inﬂuence of cannabis use during immunotherapy treatment with nivolumab on response rate, progression-free survival, and overall survival.” | n = 140  75.7%  NR | NR  NR  NR | Medical, overseen by a physician | Medical cannabis of varying THC:CBD ratios  0.67 g to >1 g cannabis |
| Abuhasira et al., 2018 ^94^  Israel  Industry | Prescribed medical cannabis | Non-randomized study | “…to assess the characteristics of the older population receiving medical cannabis for a wide variety of diseases as well as evaluate the safety and eﬃcacy of short and medium-term use” | n = 2,736  46.5%  NR | NR  NR  NR | Medical, overseen by a physician | THC-rich C. indica (20% THC, 1% CBD), THC-rich C. sativa (20% THC, 1% CBD), CBD-rich C. indica (1% THC, 18% CBD), CBD-rich C. sativa (1% THC, 20% CBD), Equal THC and CBD C. indica (10% THC, 12% CBD), Equal THC and CBD C. sativa (7% THC, 7% CBD), or Other C. indica (THC 10%, CBD 5%)  0.96 g cannabis |
| Bellnier et al., 2018 ^99^  USA  Funded not reported | Chronic non-cancer pain | Non-randomized study | “…to investigate medical cannabis's utility in the treatment of chronic pain.” | n = 29  34%  NR | NR  NR  NR | Medical, overseen by a physician | THC:CBD extract (pills 1:1 and vapor 20:1)  20 to 30 mg (maintenance) |
| Choi et al., 2018 ^107^  USA  Not funded | Older general public | Cross-sectional  NESARC-III, 2012–13 | “…to examine marijuana use’s association with injury and emergency department visits among the 50+ age group.” | n = 14,715  46.9%  Exposed group:  73.2% Caucasian, 15.6% Black, 6.3% Hispanic, 0.8% Asian, 4.1% Native American | Exposed group:  43.2% married or cohabiting, 6.3% widowed, 37.8% divorced or separated, 12.78% never married  NR  NR | NR | Past-year cannabis smoking |
| Crowley et al., 2018 ^111^  USA  Funding not reported | Chronic non-cancer pain | Prospective cohort | “…to assess the ability of a standardized cannabis formulation containing polyethylene glycol (TrokieR lozenges) to deliver cannabinoids via the buccal/oral mucosa as well as associated adverse events” | n = 49  31%  NR | NR  NR  NR | Medical, overseen by a physician | Lozenges of THC, CBD and THC:CBD of varying strengths and unknown origin |
| Han et al., 2018 ^116^  USA  Non-industry funding | Older general public | Sequential  NSDUH, 2015–16 | “…to update estimates of the prevalence of marijuana use and to examine demographic and other drug use characteristics of users among middle-aged and older adults.” | n = 17,608  46.9%  Exposed group:  ~75% Caucasian, 14% Black, 6% Hispanic, 2.5% Asian, 2.5% Other | Exposed group:  ~47% married, 9% widowed, 31% divorced or separated, 13% never married  NR  NR | Medical, overseen by a physician; Non-medical/recreational | Any past-year use |
| Hickernell et al., 2018 ^120^  USA  Funding not reported | Joint replacement | Retrospective cohort | “…to assess the efﬁcacy of dronabinol, in addition to a standard multimodal pain regimen including opioids, on acute postoperative pain management following total hip and knee replacement” | n = 243  35.8%  NR | NR  NR  100% hospitalized | Medical, overseen by a physician | Dronabinol  10 mg THC |
| Johnson-Sasso et al., 2018 ^123^  USA  Non-industry funding | Acute myocardial infarction | Retrospective cohort | “…to quantify short-term outcomes in marijuana-using and non-using patients hospitalized with acute myocardial infarction” | n = 1,014,854  NR  NR | NR  NR  NR | NR | Not reported |
| Lane et al., 2018 ^126^  USA  Non-industry funding | Psychiatric illness and admission to psychiatric hospital | Retrospective cohort | “…to examine associations among substance use disorder and measures of length of stay and non‐psychiatric medical comorbidity in older‐adult inpatients with serious mental illness” | n = 7,258  57%  49% Caucasian, 43% Black, 6% Hispanic, 2% Asian, <1% Native American/Eskimo, <1% Other | NR  NR  100% hospitalized | NR | NR |
| Mondello et al., 2018 ^136^  Italy  Funding not reported | Failed back surgery syndrome | Retrospective cohort | “…to document the results obtained with oral administration of cannabinoids agonists, namely a combination of THC/CBD, in eleven refractory failed back surgery syndrome patients” | n = 11  55%  NR | NR  NR  NR | Medical, overseen by a physician | THC:CBD oil (19:<1)  50 to 100 mg |
| Morris et al., 2018 ^137^  USA  Non-industry funding | Tobacco use | Prospective cohort | “…to evaluate marijuana usage patterns and respiratory risks in older adults or in those with chronic obstructive pulmonary disease” | n = 2,304  53.1%  77.4% Caucasian | NR  NR  NR | Non-medical/ recreational | Past-month recreational smoking of cannabis, pot, or hashish |
| Nugent et al., 2018 ^140^  USA  Non-industry funding | Chronic non-cancer pain | Cross-sectional  Baseline data from a prospective cohort study | To describe the pain, substance use, and mental health related characteristics and adverse events among patients diagnosed with musculoskeletal pain who are prescribed long-term opioid therapy and are also using medical cannabis. | n = 371  54%  83% Caucasian | 58% married  38% retired, 33% working  NR | Medical, overseen by a physician; Medical, self-medicated | Any past-month medical cannabis use |
| Thayer, 2018 (Study 1) ^153,162^  USA  Non-industry funding | Healthy older adults | Cross-sectional | “…to assess structural MRI in older adults who are current marijuana users” | n = 56  51.8%  Exposed group:  96% Caucasian, 1% Hispanic, 0% Asian | NR  NR  NR | Non-medical/ recreational | At least weekly cannabis use |
| Thayer, 2018 (Study 2) ^153^  USA  Non-industry funding | Healthy older adults | Cross-sectional | “…to collect brief assessments representing major areas of cognitive function (e.g., executive function, attention, memory, language, processing speed) in order to investigate functional correlates of marijuana use and brain structure” | n = 38  51.8%  Exposed group:  96% Caucasian, 1% Hispanic, 0% Asian | NR  NR  NR | Non-medical/ recreational | At least weekly cannabis use |
| Thayer et al., 2018 (Study 3) ^153^  USA  Non-industry funding | Healthy older adults | Cross-sectional | “…to characterize associations between measures of marijuana use and brain structure and cognitive function within the group of marijuana users” | n = 28  51.8%  Exposed group:  96% Caucasian, 1% Hispanic, 0% Asian | NR  NR  NR | Non-medical/ recreational | At least weekly cannabis use |
| Zhang et al., 2018 ^161^  Canada  Funding source not reported | Cancer | Prospective cohort | “…to evaluate the effects of cannabis on psychosocial and quality of life outcomes among patients with newly diagnosed head and neck cancers.” | n = 148  83.3%  NR | NR  34% full-time work, 1% part-time work, 47% retired, 4% unemployed, 14% disability  NR | NR | Smoking of loose-leaf marijuana at least weekly |
| Adejumo et al., 2017 ^95^  USA  Non-industry funding | Older general public | Cross-sectional | “…to test the hypothesis that cannabis use is associated with reduced prevalence of non-alcoholic fatty liver disease given its suppressive effect against obesity and diabetes in humans.” | n = 2,966,345  NR  NR | NR  NR  NR | NR | Any use of Indian hemp, marijuana and other varieties of cannabis and cannabinoids |
| Balash et al., 2017 ^97^  Israel  Funding not reported | Parkinson's disease | Cross-sectional  Study-specific survey, 2013–15 | “…to assess the effect of medical cannabis as adjuvant symptomatic treatment for various Parkinson's Disease symptoms, and its adverse effects in patients who were granted a license for medical cannabis use by the Ministry of Health in response to a formal request submitted by the patients' neurologists.” | n = 47  85.1%  NR | NR  36.2% employed  NR | Medical, overseen by a physician; Non-medical/ recreational | Daily whole plant extracts smoked, vaped, or oil  0.2 to 2.25 g cannabis |
| Bohnert et al., 2017 ^103^  USA  Non-industry funding | Older general public | Retrospective cohort  National Veterans Hospital Administration (VHA) system, 2005–11 | “…to: (1) estimate the overall and SUD-speciﬁc suicide rates among the entire cohort, as well as by sex; (2) estimate associations between current SUD diagnoses and the risk of suicide in the 6 years of follow-up using a series of sex-stratiﬁed proportional hazards models; and (3) calculate, and compare by SUD diagnosis status and sex, percentages of method of suicide among cohort members who died by suicide during follow-up.” | n = 3,944,770  91.7%  NR | NR  NR  NR | NR | Current cannabis use disorder |
| Corroon et al., 2017 ^109^  USA  Non-industry funding | Older general public | Cross-sectional  Study-specific survey | “…to analyze self-reported data for frequency of prescription drug substitution with cannabis use across sociodemographic characteristics, prescription drug class, state legalization policies for medical cannabis and global quality of life health scores.” | n = 415  NR  NR | NR  NR  NR | Medical, overseen by a physician; Medical, self-medicated; Non-medical/ recreational | Any past-3-month use |
| Patanwala et al., 2017 ^142^  USA  Non-industry funding | Homelessness | Prospective cohort | “…to evaluate associations between sociodemographic characteristics, life conditions, health conditions, health-related behaviours, and other symptom domains, and moderate to severe physical symptoms.” | n = 283  75.6%  82.3% Black, 8.1% Caucasian, 9.5% Other | NR  NR  100% homeless | NR | Past-6-month cannabis use of moderate risk (ASSIST score >= 4) |
| Rumalla et al., 2017 ^145^  USA  Funding not reported | Subarachnoid hemorrhage | Retrospective cohort | “…to 1) report the ﬁrst national rates of 30-day and 90-day readmissions in patients after acute subarachnoid hemorrhage, 2) identify novel causes for both 30-day and 90-day readmission, 3) validate previous risk factors for 30-day readmission, and 4) report outcomes associated with readmissions” | n = 12,777  38.2%  NR | NR  NR  55% at home, 28.1% skilled nursing/intermediate care facility, 12.8% home health care, 3.4% short-term facility | NR | Current cannabis use disorder |
| Salas-Wright et al., 2017 ^146^  USA  Non-industry funding | Older general public | Sequential  NSDUH, 2002–14 | “…to provide a systematic examination of the trends and correlates of marijuana use among late middle-aged (ages 50–64) and older adults (ages 65 and older) in the US” | n = 76,018  46.5%  Exposed group:  ~75% Caucasian, 15% Black, 5.5% Hispanic, 4.5% Other | Exposed group:  ~50% married, 10% widowed, 31% divorced or separated, 9% never married | NR | Any past-year use |
| Shohet et al., 2017 ^149^  Israel  Not funded | Parkinson's disease | Prospective cohort | “…to determine whether the cannabis-induced decrease in pain scores is also manifested in physiological pain parameters, as measured by quantitative sensory testing” | n = 20  NR  NR | NR  NR  NR | Medical, overseen by a physician | Whole-plant medical cannabis that was smoked or vaped  1 g cannabis |
| Yust-Katz et al., 2017 ^160^  Israel  Funding not reported | Parkinson's disease | Cross-sectional  Study-specific survey | “…to examine the prevalence and characteristics of pain in Parkinson’s disease and Parkinson plus syndromes and patient use and response to pain medications.” | n = 377  60.6%  55.6% Ashkenazi Jewish, 29% Sephardic Jewish, 13.4% Yemenite Jewish, 1% Arabic, 0.8% Other | NR  NR  NR | NR | Not reported |
| Choi et al., 2016a ^105^  USA  Funding not reported | Older general public | Sequential  NSDUH, 2008–12 | “…to examine in adults aged 50+ the relationships between marijuana and other illicit drug use and major depressive episodes and serious suicidal thoughts” | n = 29,634  46.5%  76.1% Caucasian, 9.8% Black, 8.6% Hispanic, 3.5% Asian, 1.9% Other | 62.8% married, 37.2% not married  49.5% employed, 50.5% not employed  NR | NR | Past-year medical cannabis use |
| Choi et al., 2016b ^106^  USA  Funding not reported | Older general public | Cross-sectional  NESARC-III, 2012–13 | “…to examine whether never users (those who have never used marijuana in their lifetime), past-year users (those who used marijuana only during the past year or both in the past year and prior to the past year), and ex-users (those who used marijuana prior to but not in the past year) differ in sociodemographic characteristics and past-year and lifetime mental and substance use disorders; and (2) comparing past-year users and ex-users by initiation age, duration of use, and amount used during peak use periods and identifying the risk factors for lifetime mental and substance use disorders in these two groups” | n = 14,715  46.7%  75.1% Caucasian, 10.0% Black, 9% Hispanic, 4.5% Asian, 1.4% Native American/Alaskan | 63.4% married, 12.7% widowed, 17.5% divorced or separated, 6.4% never married  42.6% employed, 57.4% not employed  NR | Medical, overseen by a physician; Non-medical/ recreational | Past-year smoking of cannabis  Mean dose = 3.19 joints/day |
| Ko et al., 2016 ^124^  USA  Non-industry funding | Older general public | Sequential  National Health and Nutrition Examination Survey (NHANES), 2005–08 | “…to determine risk factors for glaucoma in a population-based study in the United States” | n = 5,746  47.5%  Glaucoma cases:  71.3% Caucasian, 17% Black, 4.8% Hispanic, 6.9% Other | NR  NR  NR | NR | Any past-month use (at least 5 days/month) |
| Shelef et al., 2016 ^148^  Israel  Funding not reported | Alzheimer's disease/ dementia and hospitalized | Non-randomized study | “…to test medical cannabis oil in a small group of patients suffering from Alzheimer’s dementia and co-morbid behavioural and psychological symptoms of dementia” | n = 10  50%  NR | NR  NR  100% hospitalized | Medical, overseen by a physician | THC:CBD extract oil (33:1)  5 to 15 mg THC |
| Hartz et al., 2014 ^119^  USA  Non-industry funding | Severe psychotic illness (schizophrenia, bipolar disorder with psychotic features, or schizoaffective disorder) for cases | Case-control | “…to compare substance use in individuals with severe psychotic illness to substance use in general population” | n = 5,946  NR  NR | NR  NR  NR | NR | Past-year smoking of cannabis |
| Lank et al., 2014 ^127^  USA  Not funded | Trauma | Retrospective cohort | “…to describe the outcomes of older trauma patients meeting trauma activation criteria who screened positive for use of drugs of abuse and for ethanol intoxication and to compare the severity of injury via hospital metrics between older trauma patients who screen positive for drugs of abuse or ethanol intoxication with those who screen negative” | n = 38,397  60.1%  73.8% Caucasian | NR  NR  NR | NR | NR |
| Lotan et al., 2014 ^131^  Israel  Funding not reported | Parkinson's disease | Prospective cohort | “…to evaluate the efﬁcacy of cannabis treatment in alleviating the motor and non–motor symptoms of Parkinson's disease in a clinical setting” | n = 20  55%  NR | NR  NR  NR | Medical, overseen by a physician | Smoking whole-plant cannabis |
| Parsons et al., 2014 ^141^  USA  Non-industry funding | HIV-positive and on HIV medication | Cross-sectional  Study-specific survey | “…to identify patterns in the types and frequency of substance use among older adults living with HIV, and to determine whether patterns of use are differentially associated with HIV medication adherence.” | n = 557  68.5%  68.6% Black, 11.3% Caucasian, 17.4% Hispanic, 2.7% Other | 42% partnered, 58% single  NR  NR | NR | Past-month self-medication use |
| Woodward et al., 2014 ^158^  USA  Funding not reported | Alzheimer's disease/ dementia and hospitalized | Retrospective cohort | “…to determine if dronabinol treatment was well tolerated and significantly improved noncognitive behavioural symptoms of agitation and aggression or resistance to care, in a retrospective chart review of elderly, severely demented inpatients with behavioural disturbances.” | n = 40  30%  NR | NR  NR  100% hospitalized | Medical, overseen by a physician | Dronabinol  7.03 mg THC |
| Bar-Sela et al., 2013 ^98^  Israel  Funding not reported | Cancer | Prospective cohort | “…to evaluate the advantage, side effects, and administrative problems concerning the daily use of cannabis in cancer patients” | n = 29  NR  NR | NR  NR  NR | Medical, overseen by a physician | Whole plant cultivars of varying % Sativa and Indica, smoked, vaped, or oil |
| Bestard et al., 2010 ^101^  Canada  Mixed funding | Chronic non-cancer pain | Prospective cohort | “…to compare the efficacy of nabilone as either monotherapy or adjuvant treatment for neuropathic pain” | n = 130  42%  NR | NR  NR  NR | Medical, overseen by a physician | Nabilone vs gabapentin  3.05 mg THC |
| Mann et al., 2010 ^134^  Canada  Non-industry funding | Older general public | Sequential  Centre for Addiction and Mental Health (CAMH) Monitor, 2002–05 | “…to examine differences in factors associated with self-reported collision involvement of three age groups of drivers based on a large representative sample of Ontario adults” | n = 1,576  47.7%  NR | 66.2% married or partnered, 32.9% not married or partnered  NR  NR | NR | Any past-year use |
| Blazer et al., 2009 ^102^  USA  Non-industry funding | Older general public | Sequential  NSDUH, 2005–06 | “…to estimate the frequency, distribution, and correlates of non-prescription use of pain relievers among middle aged and elderly persons in the United States” | n = 10,953  46.1%  77.9% Caucasian, 9.6% Black, 7.8% Hispanic, 3.2% Asian/Pacific Islander/native Hawaiian, 1.1% multiple race, 0.4% Native American/Alaskan | 64.1% married; 30.7% divorced, separated, or widowed; 5.3% never married  50% employed, 50% not employed  NR | NR | Past-year use of hashish, pot, or grass |
| Liang et al., 2009 ^129^  USA  Non-industry funding | Cancer | Case-control | “…to elucidate the association between marijuana use and head neck cancer risk” | n = 981  73.4%  Cases:  90.7% Caucasian, 3.5% Black, 5.8% Other | NR  NR  NR | NR | Implied past-year smoking of cannabis |
| Maida et al., 2008 ^133^  Canada  Industry funding | Cancer | Prospective cohort | “…to assess the efficacy of adjunctive cannabinoid therapy for managing multiple symptoms and side effects in advanced cancer patients” | n = 112  58%  Exposed group:  95.7% Caucasian, 4.3% Other | NR  NR  Exposed group:  72.3% at home, 27.7% hospitalized | Medical, overseen by a physician | Nabilone  1.79 mg THC |
| Hartel et al., 2006 ^118^  USA  Non-industry funding | Ever use of heroin or cocaine | Cross-sectional  Baseline data from a prospective cohort study | “…to assess gender in relation to heroin and cocaine use, which may underlie a continuing risk of HIV transmission.” | n = 627  84%  ~54% Black, 27% Hispanic, 12.5% Caucasian, 6.5% Other | NR  ~19.5% employed  ~6.5% homeless in the last 6 months | NR | Recreational smoking of cannabis |
| Walther et al., 2006 ^156^  Germany  Funding not reported | Alzheimer's disease/ dementia and hospitalized | Non-randomized study | “…to obtain the first objective data on the effects of dronabinol on behavioural and day-night rhythm disturbances in dementia, using biometric instruments” | n = 6  33%  NR | NR  NR  100% hospitalized | Medical, overseen by a physician | Dronabinol  2.5 mg THC |
| Venderova et al., 2004 ^154^  Czech Republic  Non-industry funding | Parkinson's disease | Cross-sectional  Study-specific survey | “…to evaluate the experiences of Parkinson's Disease patients who spontaneously started to take cannabis to alleviate their symptoms” | n = 339  65%  NR | NR  NR  NR | Medical, self-medicated | Self-medicated use of whole-plant cannabis |
| Sasco et al., 2002 ^147^  Morocco  Non-industry funding | Cancer | Case-control | “…to examine the relationship between lung cancer and several established and suspected risk factors” | n = 353  96.6%  Cases:  91.5% Caucasian, 8.5% Black or mixed race | Cases:  89% married, 3.4% single, 2.5% divorced or separated, 5.1% widowed  NR  NR | Non-medical/ recreational | Smoking of hashish or kiff, implied to be current |
| ^a^Minor modifications may have been made to the wording of study objectives to adjust verb tense, etc. Although potentially paraphrased, we have placed objectives in quotes to emphasize that these are based upon the authors’ wording.  ^b^A sequential design amalgamated population-level survey data from multiple survey years. Data were usually from annual national surveys, in which different individuals were included each year.  ^c^A cross-sectional design surveyed individuals at a single point in time.  CAMH = Centre for Addiction and Mental Health; CBD = cannabidiol; HIV = human immunodeficiency virus; NESARC = National Epidemiologic Survey on Alcohol and Related Conditions; NHANES = National Health and Nutrition Examination Survey; NR = not reported; NSDUH = National Surveys on Drug Use and Health; THC = delta-9-tetrahydrocannabinol | | | | | | | |

References

1. Statistics Canada, Rotermann M. What has changed since cannabis was legalized? Health Rep. 2020 Feb;31(2):11–20.

2. Statistics Canada. National Cannabis Survey, third quarter 2019 [Internet]. Government of Canada; 2019 May [cited 2021 Nov 29]. Available from: https://www150.statcan.gc.ca/n1/daily-quotidien/191030/dq191030a-eng.htm

3. DiNitto DM, Choi NG. Marijuana use among older adults in the U.S.A.: user characteristics, patterns of use, and implications for intervention. Int Psychogeriatr. 2011 Jun;23(5):732–41.

4. Choi NG, DiNitto DM. Marijuana use/nonuse among those aged 50+: comparisons of use-to-nonuse, initiation/reinitiation, and continued use over 24 months. Aging Ment Health. 2021 Jun 3;25(6):1134–42.

5. Tumati S, Lanctôt KL, Wang R, Li A, Davis A, Herrmann N. Medical Cannabis Use Among Older Adults in Canada: Self-Reported Data on Types and Amount Used, and Perceived Effects. Drugs Aging. 2022 Feb;39(2):153–63.

6. Lyness JM, Caine ED, King DA, Cox C, Yoediono Z. Psychiatric disorders in older primary care patients. J Gen Intern Med. 1999 Apr;14(4):249–54.

7. Ward BW, Schiller JS. Prevalence of Multiple Chronic Conditions Among US Adults: Estimates From the National Health Interview Survey, 2010. Prev Chronic Dis. 2013 Apr 25;10:120203.

8. Minerbi A, Häuser W, Fitzcharles MA. Medical Cannabis for Older Patients. Drugs Aging. 2019 Jan;36(1):39–51.

9. Hall W. Minimizing the adverse public health effects of cannabis legalization. Can Med Assoc J. 2018 Sep 4;190(35):E1031–2.

10. Lee C, Round JM, Klarenbach S, Hanlon JG, Hyshka E, Dyck JRB, et al. Gaps in evidence for the use of medically authorized cannabis: Ontario and Alberta, Canada. Harm Reduct J. 2021 Dec;18(1):61.

11. Allan GM, Ramji J, Perry D, Ton J, Beahm NP. Simplified guideline for prescribing medical cannabinoids in primary care. Can Fam Physician. 2018;64(2):111–20.

12. Flint AJ, Merali Z, Vaccarino FJ. Improving Quality of Life: Substance Use and Aging [Internet]. 2018 [cited 2019 May 28]. Available from: http://www.deslibris.ca/ID/10096147

13. Kelleher LM, Stough C, Sergejew AA, Rolfe T. The effects of cannabis on information-processing speed. Addict Behav. 2004 Aug;29(6):1213–9.

14. Ranganathan M, D’Souza DC. The acute effects of cannabinoids on memory in humans: a review. Psychopharmacology (Berl). 2006 Nov;188(4):425–44.

15. Kaag AM, Schulte MHJ, Jansen JM, van Wingen G, Homberg J, van den Brink W, et al. The relation between gray matter volume and the use of alcohol, tobacco, cocaine and cannabis in male polysubstance users. Drug Alcohol Depend. 2018 Jun;187:186–94.

16. Yamreudeewong W, Wong HK, Brausch LM, Pulley KR. Probable interaction between warfarin and marijuana smoking. Ann Pharmacother. 2009 Jul;43(7):1347–53.

17. McLeod AL, McKenna CJ, Northridge DB. Myocardial infarction following the combined recreational use of Viagra and cannabis. Clin Cardiol. 2002 Mar;25(3):133–4.

18. Wilens TE, Biederman J, Spencer TJ. Case study: adverse effects of smoking marijuana while receiving tricyclic antidepressants. J Am Acad Child Adolesc Psychiatry. 1997 Jan;36(1):45–8.

19. Kosel BW, Aweeka FT, Benowitz NL, Shade SB, Hilton JF, Lizak PS, et al. The effects of cannabinoids on the pharmacokinetics of indinavir and nelfinavir. AIDS Lond Engl. 2002 Mar 8;16(4):543–50.

20. Ahmed AIA, van den Elsen GAH, Colbers A, van der Marck MA, Burger DM, Feuth TB, et al. Safety and pharmacokinetics of oral delta-9-tetrahydrocannabinol in healthy older subjects: a randomized controlled trial. Eur Neuropsychopharmacol J Eur Coll Neuropsychopharmacol. 2014 Sep;24(9):1475–82.

21. Tayo B, Taylor L, Sahebkar F, Morrison G. A Phase I, Open-Label, Parallel-Group, Single-Dose Trial of the Pharmacokinetics, Safety, and Tolerability of Cannabidiol in Subjects with Mild to Severe Renal Impairment. Clin Pharmacokinet. 2020 Jun;59(6):747–55.

22. Levac D, Colquhoun H, O’Brien KK. Scoping studies: advancing the methodology. Implement Sci IS. 2010 Sep 20;5:69.

23. Peters MDJ, Godfrey CM, Khalil H, McInerney P, Parker D, Soares CB. Guidance for conducting systematic scoping reviews. Int J Evid Based Healthc. 2015 Sep;13(3):141–6.

24. Thomas A, Lubarsky S, Durning SJ, Young ME. Knowledge Syntheses in Medical Education: Demystifying Scoping Reviews. Acad Med J Assoc Am Med Coll. 2017;92(2):161–6.

25. Peters, MDJ, Godfrey, C, McInerney, P, Munn, Z, Tricco, AC, Khalil, H. Chapter 11: Scoping Reviews. In: Aromataris E, Munn Z (Editors). Joanna Briggs Institute Reviewer’s Manual. [Internet]. 2020 [cited 2020 Sep 30]. Available from: https://synthesismanual.jbi.global

26. Wolfe D, Corace K, Rice D, Smith A, Kanji S, Conn D, et al. Effects of medical and non-medical cannabis use in older adults: protocol for a scoping review. BMJ Open. 2020 Feb;10(2):e034301.

27. Morales P, Reggio PH, Jagerovic N. An Overview on Medicinal Chemistry of Synthetic and Natural Derivatives of Cannabidiol. Front Pharmacol [Internet]. 2017 Jun 28 [cited 2019 Jul 3];8. Available from: http://journal.frontiersin.org/article/10.3389/fphar.2017.00422/full

28. McGowan J, Sampson M, Salzwedel DM, Cogo E, Foerster V, Lefebvre C. PRESS Peer Review of Electronic Search Strategies: 2015 guideline statement. J Clin Epidemiol. 2016;75:40–6.

29. Shea BJ, Reeves BC, Wells G, Thuku M, Hamel C, Moran J, et al. AMSTAR 2: a critical appraisal tool for systematic reviews that include randomised or non-randomised studies of healthcare interventions, or both. BMJ. 2017 Sep 21;358:j4008.

30. Public Health Agency of Canada. Communicating about substance use in compassionate, safe and non-stigmatizing ways: a resource for Canadian health professional organizations and their membership. [Internet]. Ottawa; 2020 [cited 2021 Dec 1]. Available from: http://publications.gc.ca/collections/collection_2020/aspc-phac/HP35-127-3-2020-eng.pdf

31. Tricco AC, Lillie E, Zarin W, O’Brien KK, Colquhoun H, Levac D, et al. PRISMA Extension for Scoping Reviews (PRISMA-ScR): Checklist and Explanation. Ann Intern Med. 2018 Sep 4;

32. Häuser W, Fitzcharles MA, Radbruch L, Petzke F. Cannabinoids in Pain Management and Palliative Medicine. Dtsch Aerzteblatt Online [Internet]. 2017 Sep 22 [cited 2019 Aug 20]; Available from: https://www.aerzteblatt.de/10.3238/arztebl.2017.0627

33. Häuser W, Petzke F, Fitzcharles MA. Efficacy, tolerability and safety of cannabis-based medicines for chronic pain management - An overview of systematic reviews. Eur J Pain. 2018 Mar;22(3):455–70.

34. Bao Y, Kong X, Yang L, Liu R, Shi Z, Li W, et al. Complementary and Alternative Medicine for Cancer Pain: An Overview of Systematic Reviews. Evid Based Complement Alternat Med. 2014;2014:1–9.

35. Allan GM, Finley CR, Ton J, Perry D, Ramji J, Crawford K, et al. Systematic review of systematic reviews for medical cannabinoids. Can Fam Physician. 2018;64(2):e78–94.

36. Montero-Oleas N, Arevalo-Rodriguez I, Nuñez-González S, Viteri-García A, Simancas-Racines D. Therapeutic use of cannabis and cannabinoids: an evidence mapping and appraisal of systematic reviews. BMC Complement Med Ther. 2020 Dec;20(1):12.

37. Charernboon T, Lerthattasilp T, Supasitthumrong T. Effectiveness of Cannabinoids for Treatment of Dementia: A Systematic Review of Randomized Controlled Trials. Clin Gerontol. 2021 Jan 1;44(1):16–24.

38. Bahji A, Meyyappan AC, Hawken ER. Cannabinoids for the Neuropsychiatric Symptoms of Dementia: A Systematic Review and Meta-Analysis. Can J Psychiatry. 2020 Jun;65(6):365–76.

39. Boland EG, Bennett MI, Allgar V, Boland JW. Cannabinoids for adult cancer-related pain: systematic review and meta-analysis. BMJ Support Palliat Care. 2020 Mar;10(1):14–24.

40. Braud A, Boucher Y. Taste disorder’s management: a systematic review. Clin Oral Investig. 2020 Jun;24(6):1889–908.

41. Fisher E, Moore RA, Fogarty AE, Finn DP, Finnerup NB, Gilron I, et al. Cannabinoids, cannabis, and cannabis-based medicine for pain management: a systematic review of randomised controlled trials. Pain [Internet]. 2020 May 18 [cited 2021 Jul 30];Publish Ahead of Print. Available from: https://journals.lww.com/10.1097/j.pain.0000000000001929

42. Suraev AS, Marshall NS, Vandrey R, McCartney D, Benson MJ, McGregor IS, et al. Cannabinoid therapies in the management of sleep disorders: A systematic review of preclinical and clinical studies. Sleep Med Rev. 2020 Oct;53:101339.

43. Gaisl T, Haile SR, Thiel S, Osswald M, Kohler M. Efficacy of pharmacotherapy for OSA in adults: A systematic review and network meta-analysis. Sleep Med Rev. 2019 Aug;46:74–86.

44. Ghasemiesfe M, Barrow B, Leonard S, Keyhani S, Korenstein D. Association Between Marijuana Use and Risk of Cancer: A Systematic Review and Meta-analysis. JAMA Netw Open. 2019 Nov 27;2(11):e1916318.

45. Gouveia DN, Guimarães AG, Santos WB da R, Quintans-Júnior LJ. Natural products as a perspective for cancer pain management: A systematic review. Phytomedicine. 2019 May;58:152766.

46. Häuser W, Welsch P, Klose P, Radbruch L, Fitzcharles MA. Efficacy, tolerability and safety of cannabis-based medicines for cancer pain: A systematic review with meta-analysis of randomised controlled trials. Schmerz [Internet]. 2019 May 9 [cited 2019 Aug 20]; Available from: http://link.springer.com/10.1007/s00482-019-0373-3

47. Hoch E, Niemann D, von Keller R, Schneider M, Friemel CM, Preuss UW, et al. How effective and safe is medical cannabis as a treatment of mental disorders? A systematic review. Eur Arch Psychiatry Clin Neurosci. 2019 Feb;269(1):87–105.

48. Millar SA, Stone NL, Bellman ZD, Yates AS, England TJ, O’Sullivan SE. A systematic review of cannabidiol dosing in clinical populations. Br J Clin Pharmacol. 2019 Sep;85(9):1888–900.

49. Ruthirakuhan M, Lanctôt KL, Vieira D, Herrmann N. Natural and Synthetic Cannabinoids for Agitation and Aggression in Alzheimer’s Disease: A Meta-Analysis. J Clin Psychiatry [Internet]. 2019 Jan 29 [cited 2019 Aug 20];80(2). Available from: https://www.psychiatrist.com/JCP/article/Pages/2019/v80/18r12617.aspx

50. Wang J, Wang Y, Tong M, Pan H, Li D. Medical Cannabinoids for Cancer Cachexia: A Systematic Review and Meta-Analysis. BioMed Res Int. 2019 Jun 23;2019:1–6.

51. Mücke M, Weier M, Carter C, Copeland J, Degenhardt L, Cuhls H, et al. Systematic review and meta-analysis of cannabinoids in palliative medicine: Cannabinoids in palliative medicine. J Cachexia Sarcopenia Muscle. 2018 Apr;9(2):220–34.

52. Goldenberg M, Reid MW, IsHak WW, Danovitch I. The impact of cannabis and cannabinoids for medical conditions on health-related quality of life: A systematic review and meta-analysis. Drug Alcohol Depend. 2017 May;174:80–90.

53. Lim K, See YM, Lee J. A Systematic Review of the Effectiveness of Medical Cannabis for Psychiatric, Movement and Neurodegenerative Disorders. Clin Psychopharmacol Neurosci. 2017 Nov 30;15(4):301–12.

54. Nielsen S, Sabioni P, Trigo JM, Ware MA, Betz-Stablein BD, Murnion B, et al. Opioid-Sparing Effect of Cannabinoids: A Systematic Review and Meta-Analysis. Neuropsychopharmacol Off Publ Am Coll Neuropsychopharmacol. 2017 Aug;42(9):1752–65.

55. Nugent SM, Morasco BJ, O’Neil ME, Freeman M, Low A, Kondo K, et al. The Effects of Cannabis Among Adults With Chronic Pain and an Overview of General Harms: A Systematic Review. Ann Intern Med. 2017 Sep 5;167(5):319–31.

56. van den Beuken-van Everdingen MHJ, de Graeff A, Jongen JLM, Dijkstra D, Mostovaya I, Vissers KC, et al. Pharmacological Treatment of Pain in Cancer Patients: The Role of Adjuvant Analgesics, a Systematic Review. Pain Pract. 2017 Mar;17(3):409–19.

57. Whiting PF, Wolff RF, Deshpande S, Di Nisio M, Duffy S, Hernandez AV, et al. Cannabinoids for Medical Use: A Systematic Review and Meta-analysis. JAMA. 2015 Jun 23;313(24):2456.

58. van den Elsen GAH, Ahmed AIA, Lammers M, Kramers C, Verkes RJ, van der Marck MA, et al. Efficacy and safety of medical cannabinoids in older subjects: A systematic review. Ageing Res Rev. 2014 Mar;14:56–64.

59. Abdallah SJ, Smith BM, Ware MA, Moore M, Li PZ, Bourbeau J, et al. Effect of Vaporized Cannabis on Exertional Breathlessness and Exercise Endurance in Advanced Chronic Obstructive Pulmonary Disease. A Randomized Controlled Trial. Ann Am Thorac Soc. 2018;15(10):1146–58.

60. Ahmed AIA, van den Elsen GAH, Colbers A, Kramers C, Burger DM, van der Marck MA, et al. Safety, pharmacodynamics, and pharmacokinetics of multiple oral doses of delta-9-tetrahydrocannabinol in older persons with dementia. Psychopharmacology (Berl). 2015 Jul;232(14):2587–95.

61. Beaulieu P. Effects of nabilone, a synthetic cannabinoid, on postoperative pain. Can J Anaesth J Can Anesth. 2006 Aug;53(8):769–75.

62. Blake DR, Robson P, Ho M, Jubb RW, McCabe CS. Preliminary assessment of the efficacy, tolerability and safety of a cannabis-based medicine (Sativex) in the treatment of pain caused by rheumatoid arthritis. Rheumatol Oxf Engl. 2006 Jan;45(1):50–2.

63. Brisbois TD, de Kock IH, Watanabe SM, Mirhosseini M, Lamoureux DC, Chasen M, et al. Delta-9-tetrahydrocannabinol may palliate altered chemosensory perception in cancer patients: results of a randomized, double-blind, placebo-controlled pilot trial. Ann Oncol Off J Eur Soc Med Oncol. 2011 Sep;22(9):2086–93.

64. Cannabis-In-Cachexia-Study-Group, Strasser F, Luftner D, Possinger K, Ernst G, Ruhstaller T, et al. Comparison of orally administered cannabis extract and delta-9-tetrahydrocannabinol in treating patients with cancer-related anorexia-cachexia syndrome: a multicenter, phase III, randomized, double-blind, placebo-controlled clinical trial from the Cannabis-In-Cachexia-Study-Group. J Clin Oncol Off J Am Soc Clin Oncol. 2006 Jul 20;24(21):3394–400.

65. Carroll CB, Bain PG, Teare L, Liu X, Joint C, Wroath C, et al. Cannabis for dyskinesia in Parkinson disease: a randomized double-blind crossover study. Neurology. 2004 Oct 12;63(7):1245–50.

66. Chagas MHN, Zuardi AW, Tumas V, Pena-Pereira MA, Sobreira ET, Bergamaschi MM, et al. Effects of cannabidiol in the treatment of patients with Parkinson’s disease: an exploratory double-blind trial. J Psychopharmacol Oxf Engl. 2014 Nov;28(11):1088–98.

67. Conte A, Bettolo CM, Onesti E, Frasca V, Iacovelli E, Gilio F, et al. Cannabinoid-induced effects on the nociceptive system: a neurophysiological study in patients with secondary progressive multiple sclerosis. Eur J Pain Lond Engl. 2009 May;13(5):472–7.

68. Côté M, Trudel M, Wang C, Fortin A. Improving Quality of Life With Nabilone During Radiotherapy Treatments for Head and Neck Cancers: A Randomized Double-Blind Placebo-Controlled Trial. Ann Otol Rhinol Laryngol. 2016 Apr;125(4):317–24.

69. de Faria SM, de Morais Fabrício D, Tumas V, Castro PC, Ponti MA, Hallak JE, et al. Effects of acute cannabidiol administration on anxiety and tremors induced by a Simulated Public Speaking Test in patients with Parkinson’s disease. J Psychopharmacol (Oxf). 2020 Feb;34(2):189–96.

70. Fallon MT, Albert Lux E, McQuade R, Rossetti S, Sanchez R, Sun W, et al. Sativex oromucosal spray as adjunctive therapy in advanced cancer patients with chronic pain unalleviated by optimized opioid therapy: two double-blind, randomized, placebo-controlled phase 3 studies. Br J Pain. 2017 Aug;11(3):119–33.

71. Frytak S, Moertel CG, O’Fallon JR, Rubin J, Creagan ET, O’Connell MJ, et al. Delta-9-tetrahydrocannabinol as an antiemetic for patients receiving cancer chemotherapy. A comparison with prochlorperazine and a placebo. Ann Intern Med. 1979 Dec;91(6):825–30.

72. Herrmann N, Ruthirakuhan M, Gallagher D, Verhoeff NPLG, Kiss A, Black SE, et al. Randomized Placebo-Controlled Trial of Nabilone for Agitation in Alzheimer’s Disease. Am J Geriatr Psychiatry Off J Am Assoc Geriatr Psychiatry. 2019 Nov;27(11):1161–73.

73. Jadoon KA, Ratcliffe SH, Barrett DA, Thomas EL, Stott C, Bell JD, et al. Efficacy and Safety of Cannabidiol and Tetrahydrocannabivarin on Glycemic and Lipid Parameters in Patients With Type 2 Diabetes: A Randomized, Double-Blind, Placebo-Controlled, Parallel Group Pilot Study. Diabetes Care. 2016 Oct;39(10):1777–86.

74. Jatoi A, Windschitl HE, Loprinzi CL, Sloan JA, Dakhil SR, Mailliard JA, et al. Dronabinol versus megestrol acetate versus combination therapy for cancer-associated anorexia: a North Central Cancer Treatment Group study. J Clin Oncol Off J Am Soc Clin Oncol. 2002 Jan 15;20(2):567–73.

75. Jatoi A, Yamashita J ichi, Sloan JA, Novotny PJ, Windschitl HE, Loprinzi CL. Does megestrol acetate down-regulate interleukin-6 in patients with cancer-associated anorexia and weight loss? A North Central Cancer Treatment Group investigation. Support Care Cancer Off J Multinatl Assoc Support Care Cancer. 2002 Jan;10(1):71–5.

76. Johnson JR, Burnell-Nugent M, Lossignol D, Ganae-Motan ED, Potts R, Fallon MT. Multicenter, double-blind, randomized, placebo-controlled, parallel-group study of the efficacy, safety, and tolerability of THC:CBD extract and THC extract in patients with intractable cancer-related pain. J Pain Symptom Manage. 2010 Feb;39(2):167–79.

77. Niiranen A, Mattson K. A cross-over comparison of nabilone and prochlorperazine for emesis induced by cancer chemotherapy. Am J Clin Oncol. 1985 Aug;8(4):336–40.

78. Notcutt W, Langford R, Davies P, Ratcliffe S, Potts R. A placebo-controlled, parallel-group, randomized withdrawal study of subjects with symptoms of spasticity due to multiple sclerosis who are receiving long-term Sativex® (nabiximols). Mult Scler Houndmills Basingstoke Engl. 2012 Feb;18(2):219–28.

79. Peball M, Krismer F, Knaus H, Djamshidian A, Werkmann M, Carbone F, et al. Non‐Motor Symptoms in Parkinson’s Disease are Reduced by Nabilone. Ann Neurol. 2020 Oct;88(4):712–22.

80. Pickering EE, Semple SJ, Nazir MS, Murphy K, Snow TM, Cummin AR, et al. Cannabinoid effects on ventilation and breathlessness: a pilot study of efficacy and safety. Chron Respir Dis. 2011;8(2):109–18.

81. Riva N, Mora G, Sorarù G, Lunetta C, Ferraro OE, Falzone Y, et al. Safety and efficacy of nabiximols on spasticity symptoms in patients with motor neuron disease (CANALS): a multicentre, double-blind, randomised, placebo-controlled, phase 2 trial. Lancet Neurol. 2019 Feb;18(2):155–64.

82. Selvarajah D, Gandhi R, Emery CJ, Tesfaye S. Randomized placebo-controlled double-blind clinical trial of cannabis-based medicinal product (Sativex) in painful diabetic neuropathy: depression is a major confounding factor. Diabetes Care. 2010 Jan;33(1):128–30.

83. Sieradzan KA, Fox SH, Hill M, Dick JP, Crossman AR, Brotchie JM. Cannabinoids reduce levodopa-induced dyskinesia in Parkinson’s disease: a pilot study. Neurology. 2001 Dec 11;57(11):2108–11.

84. Tomida I, Azuara-Blanco A, House H, Flint M, Pertwee RG, Robson PJ. Effect of sublingual application of cannabinoids on intraocular pressure: a pilot study. J Glaucoma. 2006 Oct;15(5):349–53.

85. Turcott JG, Del Rocío Guillen Núñez M, Flores-Estrada D, Oñate-Ocaña LF, Zatarain-Barrón ZL, Barrón F, et al. The effect of nabilone on appetite, nutritional status, and quality of life in lung cancer patients: a randomized, double-blind clinical trial. Support Care Cancer Off J Multinatl Assoc Support Care Cancer. 2018 Sep;26(9):3029–38.

86. van den Elsen GAH, Ahmed AIA, Verkes RJ, Kramers C, Feuth T, Rosenberg PB, et al. Tetrahydrocannabinol for neuropsychiatric symptoms in dementia: A randomized controlled trial. Neurology. 2015 Jun 9;84(23):2338–46.

87. van den Elsen GAH, Ahmed AIA, Verkes RJ, Feuth T, van der Marck MA, Olde Rikkert MGM. Tetrahydrocannabinol in Behavioral Disturbances in Dementia: A Crossover Randomized Controlled Trial. Am J Geriatr Psychiatry Off J Am Assoc Geriatr Psychiatry. 2015 Dec;23(12):1214–24.

88. van den Elsen GA, Tobben L, Ahmed AI, Verkes RJ, Kramers C, Marijnissen RM, et al. Effects of tetrahydrocannabinol on balance and gait in patients with dementia: A randomised controlled crossover trial. J Psychopharmacol Oxf Engl. 2017;31(2):184–91.

89. Volicer L, Stelly M, Morris J, McLaughlin J, Volicer BJ. Effects of dronabinol on anorexia and disturbed behavior in patients with Alzheimer’s disease. Int J Geriatr Psychiatry. 1997 Sep;12(9):913–9.

90. Wallace MS, Marcotte TD, Umlauf A, Gouaux B, Atkinson JH. Efficacy of Inhaled Cannabis on Painful Diabetic Neuropathy. J Pain Off J Am Pain Soc. 2015 Jul;16(7):616–27.

91. Xu DH, Cullen BD, Tang M, Fang Y. The Effectiveness of Topical Cannabidiol Oil in Symptomatic Relief of Peripheral Neuropathy of the Lower Extremities. Curr Pharm Biotechnol. 2020 Apr 29;21(5):390–402.

92. Zadikoff C, Wadia PM, Miyasaki J, Chen R, Lang AE, So J, et al. Cannabinoid, CB1 agonists in cervical dystonia: Failure in a phase IIa randomized controlled trial. Basal Ganglia. 2011 Jul;1(2):91–5.

93. Abbs E, Brown R, Guzman D, Kaplan L, Kushel M. Risk Factors for Falls in Older Adults Experiencing Homelessness: Results from the HOPE HOME Cohort Study. J Gen Intern Med. 2020 Jun;35(6):1813–20.

94. Abuhasira R, Schleider LBL, Mechoulam R, Novack V. Epidemiological characteristics, safety and efficacy of medical cannabis in the elderly. Eur J Intern Med. 2018;49:44–50.

95. Adejumo AC, Alliu S, Ajayi TO, Adejumo KL, Adegbala OM, Onyeakusi NE, et al. Cannabis use is associated with reduced prevalence of non-alcoholic fatty liver disease: A cross-sectional study. PloS One. 2017;12(4):e0176416.

96. Airagnes G, Lemogne C, Meneton P, Plessz M, Goldberg M, Hoertel N, et al. Alcohol, tobacco and cannabis use are associated with job loss at follow-up: Findings from the CONSTANCES cohort. Niaura R, editor. PLOS ONE. 2019 Sep 9;14(9):e0222361.

97. Balash Y, Bar-Lev Schleider L, Korczyn AD, Shabtai H, Knaani J, Rosenberg A, et al. Medical Cannabis in Parkinson Disease: Real-Life Patients’ Experience. Clin Neuropharmacol. 2017 Dec;40(6):268–72.

98. Bar-Sela G, Vorobeichik M, Drawsheh S, Omer A, Goldberg V, Muller E. The medical necessity for medicinal cannabis: prospective, observational study evaluating the treatment in cancer patients on supportive or palliative care. Evid-Based Complement Altern Med ECAM. 2013;2013:510392.

99. Bellnier TJ, Brown G, Ortega T, Insull R. A Preliminary Evaluation of the Efficacy, Safety, and Costs Associated with the Treatment of Chronic Pain with Medical Marijuana in the Elderly. 2018 [cited 2019 Oct 29]; Available from: http://rgdoi.net/10.13140/RG.2.2.34542.92485

100. Benitez A, Lauzon S, Nietert PJ, McRae-Clark A, Sherman BJ. Self-reported cognition and marijuana use in older adults: Results from the national epidemiologic survey on alcohol and related conditions-III. Addict Behav. 2020 Sep;108:106437.

101. Bestard JA, Toth CC. An open-label comparison of nabilone and gabapentin as adjuvant therapy or monotherapy in the management of neuropathic pain in patients with peripheral neuropathy. Pain Pract Off J World Inst Pain. 2011 Aug;11(4):353–68.

102. Blazer DG, Wu LT. Nonprescription use of pain relievers by middle-aged and elderly community-living adults: National Survey on Drug Use and Health. J Am Geriatr Soc. 2009 Jul;57(7):1252–7.

103. Bohnert KM, Ilgen MA, Louzon S, McCarthy JF, Katz IR. Substance use disorders and the risk of suicide mortality among men and women in the US Veterans Health Administration. Addict Abingdon Engl. 2017 Jul;112(7):1193–201.

104. Choi NG, DiNitto DM, Choi BY. Prescription Pain Reliever Use and Misuse among Cannabis Users Aged 50+ Years. Clin Gerontol. 2021 Jan 1;44(1):53–65.

105. Choi NG, DiNitto DM, Marti CN, Choi BY. Relationship between marijuana and other illicit drug use and depression/suicidal thoughts among late middle-aged and older adults. Int Psychogeriatr. 2016 Apr;28(4):577–89.

106. Choi NG, DiNitto DM, Marti CN. Older-adult marijuana users and ex-users: Comparisons of sociodemographic characteristics and mental and substance use disorders. Drug Alcohol Depend. 2016 Aug 1;165:94–102.

107. Choi NG, Marti CN, DiNitto DM, Choi BY. Older adults’ marijuana use, injuries, and emergency department visits. Am J Drug Alcohol Abuse. 2018;44(2):215–23.

108. Choi NG, Marti CN, DiNitto DM, Baker SD. Cannabis and synthetic cannabinoid poison control center cases among adults aged 50+, 2009–2019. Clin Toxicol. 2021 Apr 3;59(4):334–42.

109. Corroon JM, Mischley LK, Sexton M. Cannabis as a substitute for prescription drugs - a cross-sectional study. J Pain Res. 2017;10:989–98.

110. Croker JA, Bobitt JL, Arora K, Kaskie B. Assessing Health-Related Outcomes of Medical Cannabis Use among Older Persons: Findings from Colorado and Illinois. Clin Gerontol. 2021 Jan 1;44(1):66–79.

111. Crowley K, de Vries ST, Moreno-Sanz G. Self-Reported Effectiveness and Safety of Trokie® Lozenges: A Standardized Formulation for the Buccal Delivery of Cannabis Extracts. Front Neurosci. 2018;12:564.

112. Denduluri SK, Woolson ST, Indelli PF, Mariano ER, Harris AHS, Giori NJ. Cannabinoid and Opioid Use Among Total Joint Arthroplasty Patients: A 6-Year, Single-Institution Study. Orthopedics [Internet]. 2021 Jan [cited 2021 Jul 30];44(1). Available from: http://journals.healio.com/doi/10.3928/01477447-20200928-02

113. DiGuiseppi CG, Smith AA, Betz ME, Hill L, Lum HD, Andrews H, et al. Cannabis use in older drivers in Colorado: The LongROAD Study. Accid Anal Prev. 2019 Nov;132:105273.

114. Fujii Q, Olsen I, McCague A. Marijuana Screening and Trauma Outcomes. J Emerg Trauma Shock. 2020 Mar;13(1):35–8.

115. Han BH, Moore AA, Ferris R, Palamar JJ. Binge Drinking Among Older Adults in the United States, 2015 to 2017. J Am Geriatr Soc. 2019 Oct;67(10):2139–44.

116. Han BH, Palamar JJ. Marijuana use by middle-aged and older adults in the United States, 2015-2016. Drug Alcohol Depend. 2018 01;191:374–81.

117. Han BH, Sherman SE, Palamar JJ. Prescription opioid misuse among middle-aged and older adults in the United States, 2015-2016. Prev Med. 2019 Apr;121:94–8.

118. Hartel DM, Schoenbaum EE, Lo Y, Klein RS. Gender differences in illicit substance use among middle-aged drug users with or at risk for HIV infection. Clin Infect Dis Off Publ Infect Dis Soc Am. 2006 Aug 15;43(4):525–31.

119. Hartz SM, Pato CN, Medeiros H, Cavazos-Rehg P, Sobell JL, Knowles JA, et al. Comorbidity of severe psychotic disorders with measures of substance use. JAMA Psychiatry. 2014 Mar;71(3):248–54.

120. Hickernell TR, Lakra A, Berg A, Cooper HJ, Geller JA, Shah RP. Should Cannabinoids Be Added to Multimodal Pain Regimens After Total Hip and Knee Arthroplasty? J Arthroplasty. 2018;33(12):3637–41.

121. Howard ML, Hossaini R, Tolar C, Gaviola ML. Efficacy and Safety of Appetite-Stimulating Medications in the Inpatient Setting. Ann Pharmacother. 2019 Mar;53(3):261–7.

122. Jennings JM, Angerame MR, Eschen CL, Phocas AJ, Dennis DA. Cannabis Use Does Not Affect Outcomes After Total Knee Arthroplasty. J Arthroplasty. 2019 Aug;34(8):1667–9.

123. Johnson-Sasso CP, Tompkins C, Kao DP, Walker LA. Marijuana use and short-term outcomes in patients hospitalized for acute myocardial infarction. PloS One. 2018;13(7):e0199705.

124. Ko F, Boland MV, Gupta P, Gadkaree SK, Vitale S, Guallar E, et al. Diabetes, Triglyceride Levels, and Other Risk Factors for Glaucoma in the National Health and Nutrition Examination Survey 2005-2008. Invest Ophthalmol Vis Sci. 2016 Apr 1;57(4):2152–7.

125. Kwok CS, Alraies MC, Mohamed M, Rashid M, Shoaib A, Nolan J, et al. Rates, predictors and the impact of cannabis misuse on in‐hospital outcomes among patients undergoing percutaneous coronary intervention (from the National Inpatient Sample). Int J Clin Pract [Internet]. 2020 May [cited 2021 Jul 30];74(5). Available from: https://onlinelibrary.wiley.com/doi/10.1111/ijcp.13477

126. Lane SD, da Costa SC, Teixeira AL, Reynolds CF, Diniz BS. The impact of substance use disorders on clinical outcomes in older-adult psychiatric inpatients. Int J Geriatr Psychiatry. 2018;33(2):e323–9.

127. Lank PM, Crandall ML. Outcomes for older trauma patients in the emergency department screening positive for alcohol, cocaine, or marijuana use. Am J Drug Alcohol Abuse. 2014 Mar;40(2):118–24.

128. Li G, Chihuri S. Is marijuana use associated with decreased use of prescription opioids? Toxicological findings from two US national samples of drivers. Subst Abuse Treat Prev Policy. 2020 Dec;15(1):12.

129. Liang C, McClean MD, Marsit C, Christensen B, Peters E, Nelson HH, et al. A population-based case-control study of marijuana use and head and neck squamous cell carcinoma. Cancer Prev Res Phila Pa. 2009 Aug;2(8):759–68.

130. Linden-Carmichael AN, Allen HK, Masters LD, Ansell EB, Lanza ST. Age-varying trends in alcohol and cannabis co-occurring use: Implications for prescription drug misuse. Am J Drug Alcohol Abuse. 2021 Mar 4;47(2):209–19.

131. Lotan I, Treves TA, Roditi Y, Djaldetti R. Cannabis (medical marijuana) treatment for motor and non-motor symptoms of Parkinson disease: an open-label observational study. Clin Neuropharmacol. 2014 Apr;37(2):41–4.

132. Lum HD, Arora K, Croker JA, Qualls SH, Schuchman M, Bobitt J, et al. Patterns of Marijuana Use and Health Impact: A Survey Among Older Coloradans. Gerontol Geriatr Med. 2019 Dec;5:2333721419843707.

133. Maida V, Ennis M, Irani S, Corbo M, Dolzhykov M. Adjunctive nabilone in cancer pain and symptom management: a prospective observational study using propensity scoring. J Support Oncol. 2008 Mar;6(3):119–24.

134. Mann RE, Stoduto G, Butters J, Ialomiteanu A, Boase P, Asbridge M, et al. Age group differences in collision risk. J Safety Res. 2010 Oct;41(5):445–9.

135. Matson TE, Lapham GT, Bobb JF, Johnson E, Richards JE, Lee AK, et al. Cannabis use, other drug use, and risk of subsequent acute care in primary care patients. Drug Alcohol Depend. 2020 Nov;216:108227.

136. Mondello E, Quattrone D, Cardia L, Bova G, Mallamace R, Barbagallo AA, et al. Cannabinoids and spinal cord stimulation for the treatment of failed back surgery syndrome refractory pain. J Pain Res. 2018;11:1761–7.

137. Morris MA, Jacobson SR, Kinney GL, Tashkin DP, Woodruff PG, Hoffman EA, et al. Marijuana Use Associations with Pulmonary Symptoms and Function in Tobacco Smokers Enrolled in the Subpopulations and Intermediate Outcome Measures in COPD Study (SPIROMICS). Chronic Obstr Pulm Dis Miami Fla. 2018 Jan 24;5(1):46–56.

138. Mousa A, Petrovic M, Fleshner NE. Prevalence and predictors of cannabis use among men receiving androgen-deprivation therapy for advanced prostate cancer. Can Urol Assoc J [Internet]. 2019 Apr 5 [cited 2021 Jul 30];14(1). Available from: https://cuaj.ca/index.php/journal/article/view/5911

139. Neilson HK, Lin Z. Is Cannabis Use Associated with Sedentary Behavior during Leisure Time? A Study in Canada, 2011-2012. Subst Use Misuse. 2019;54(5):852–62.

140. Nugent SM, Yarborough BJ, Smith NX, Dobscha SK, Deyo RA, Green CA, et al. Patterns and correlates of medical cannabis use for pain among patients prescribed long-term opioid therapy. Gen Hosp Psychiatry. 2018 Feb;50:104–10.

141. Parsons JT, Starks TJ, Millar BM, Boonrai K, Marcotte D. Patterns of substance use among HIV-positive adults over 50: implications for treatment and medication adherence. Drug Alcohol Depend. 2014 Jun 1;139:33–40.

142. Patanwala M, Tieu L, Ponath C, Guzman D, Ritchie CS, Kushel M. Physical, Psychological, Social, and Existential Symptoms in Older Homeless-Experienced Adults: An Observational Study of the Hope Home Cohort. J Gen Intern Med. 2018;33(5):635–43.

143. Press DJ, Pierce B, Lauderdale DS, Aschebrook-Kilfoy B, Lin Gomez S, Hedeker D, et al. Tobacco and marijuana use and their association with serum prostate-specific antigen levels among African American men in Chicago. Prev Med Rep. 2020 Dec;20:101174.

144. Ramadan MM, Banta JE, Bahjri K, Montgomery SB. Marijuana users are likely to report opioid misuse among adults over 50 years in representative sample of the United States (2002–2014). J Addict Dis. 2020 Dec 1;39(1):66–73.

145. Rumalla K, Smith KA, Arnold PM, Mittal MK. Subarachnoid Hemorrhage and Readmissions: National Rates, Causes, Risk Factors, and Outcomes in 16,001 Hospitalized Patients. World Neurosurg. 2018 Feb;110:e100–11.

146. Salas-Wright CP, Vaughn MG, Cummings-Vaughn LA, Holzer KJ, Nelson EJ, AbiNader M, et al. Trends and correlates of marijuana use among late middle-aged and older adults in the United States, 2002-2014. Drug Alcohol Depend. 2017 Feb 1;171:97–106.

147. Sasco AJ, Merrill RM, Dari I, Benhaïm-Luzon V, Carriot F, Cann CI, et al. A case-control study of lung cancer in Casablanca, Morocco. Cancer Causes Control CCC. 2002 Sep;13(7):609–16.

148. Shelef A, Barak Y, Berger U, Paleacu D, Tadger S, Plopsky I, et al. Safety and Efficacy of Medical Cannabis Oil for Behavioral and Psychological Symptoms of Dementia: An-Open Label, Add-On, Pilot Study. J Alzheimers Dis JAD. 2016;51(1):15–9.

149. Shohet A, Khlebtovsky A, Roizen N, Roditi Y, Djaldetti R. Effect of medical cannabis on thermal quantitative measurements of pain in patients with Parkinson’s disease. Eur J Pain Lond Engl. 2017;21(3):486–93.

150. Sznitman SR, Vulfsons S, Meiri D, Weinstein G. Medical cannabis and insomnia in older adults with chronic pain: a cross-sectional study. BMJ Support Palliat Care. 2020 Dec;10(4):415–20.

151. Sznitman SR, Vulfsons S, Meiri D, Weinstein G. Medical cannabis and cognitive performance in middle to old adults treated for chronic pain. Drug Alcohol Rev. 2021 Feb;40(2):272–80.

152. Taha T, Meiri D, Talhamy S, Wollner M, Peer A, Bar-Sela G. Cannabis Impacts Tumor Response Rate to Nivolumab in Patients with Advanced Malignancies. The Oncologist. 2019 Apr;24(4):549–54.

153. Thayer RE. Marijuana Use in an Aging Population: Global Brain Structure and Cognitive Function. 2018.

154. Venderová K, Růzicka E, Vorísek V, Visnovský P. Survey on cannabis use in Parkinson’s disease: subjective improvement of motor symptoms. Mov Disord Off J Mov Disord Soc. 2004 Sep;19(9):1102–6.

155. Vozoris NT, Pequeno P, Li P, Austin PC, Stephenson AL, O’Donnell DE, et al. Morbidity and mortality associated with prescription cannabinoid drug use in COPD. Thorax. 2021 Jan;76(1):29–36.

156. Walther S, Mahlberg R, Eichmann U, Kunz D. Delta-9-tetrahydrocannabinol for nighttime agitation in severe dementia. Psychopharmacology (Berl). 2006 May;185(4):524–8.

157. Whitfield JB, Masson S, Liangpunsakul S, Mueller S, Aithal GP, Eyer F, et al. Obesity, Diabetes, Coffee, Tea, and Cannabis Use Alter Risk for Alcohol-Related Cirrhosis in 2 Large Cohorts of High-Risk Drinkers. Am J Gastroenterol. 2021 Jan;116(1):106–15.

158. Woodward MR, Harper DG, Stolyar A, Forester BP, Ellison JM. Dronabinol for the Treatment of Agitation and Aggressive Behavior in Acutely Hospitalized Severely Demented Patients with Noncognitive Behavioral Symptoms. Am J Geriatr Psychiatry. 2014 Apr;22(4):415–9.

159. YorkWilliams SL, Gibson LP, Gust CJ, Giordano G, Hutchison KE, Bryan AD. Exercise Intervention Outcomes with Cannabis Users and Nonusers Aged 60 and Older. Am J Health Behav. 2020 Jul 1;44(4):420–31.

160. Yust-Katz S, Hershkovitz R, Gurevich T, Djaldetti R. Pain in Extrapyramidal Neurodegenerative Diseases. Clin J Pain. 2017;33(7):635–9.

161. Zhang H, Xie M, Archibald SD, Jackson BS, Gupta MK. Association of Marijuana Use With Psychosocial and Quality of Life Outcomes Among Patients With Head and Neck Cancer. JAMA Otolaryngol-- Head Neck Surg. 2018 01;144(11):1017–22.

162. Thayer RE, YorkWilliams SL, Hutchison KE, Bryan AD. Preliminary results from a pilot study examining brain structure in older adult cannabis users and nonusers. Psychiatry Res Neuroimaging. 2019 Mar;285:58–63.

163. Klumpers LE, Beumer TL, van Hasselt JGC, Lipplaa A, Karger LB, Kleinloog HD, et al. Novel Δ9-tetrahydrocannabinol formulation Namisol® has beneficial pharmacokinetics and promising pharmacodynamic effects: PK and PD of novel THC tablet Namisol. Br J Clin Pharmacol. 2012 Jul;74(1):42–53.

164. Lichtman AH, Lux EA, McQuade R, Rossetti S, Sanchez R, Sun W, et al. Results of a Double-Blind, Randomized, Placebo-Controlled Study of Nabiximols Oromucosal Spray as an Adjunctive Therapy in Advanced Cancer Patients with Chronic Uncontrolled Pain. J Pain Symptom Manage. 2018;55(2):179-188.e1.

165. Portenoy RK, Ganae-Motan ED, Allende S, Yanagihara R, Shaiova L, Weinstein S, et al. Nabiximols for opioid-treated cancer patients with poorly-controlled chronic pain: a randomized, placebo-controlled, graded-dose trial. J Pain Off J Am Pain Soc. 2012 May;13(5):438–49.

166. Lanctot K, Ruthirakuhan M, Gallagher D. Nabilone significantly improves agitation/ aggression in patients with moderate-to- severe Alzheimer’s disease: preliminary results of a placebo-controlled, double-blind, cross-over trial. In Chicago, USA; 2018.

167. Walther S, Schüpbach B, Seifritz E, Homan P, Strik W. Randomized, controlled crossover trial of dronabinol, 2.5 mg, for agitation in 2 patients with dementia. J Clin Psychopharmacol. 2011 Apr;31(2):256–8.

168. Choi NG, DiNitto DM, Marti CN. Older marijuana users: Life stressors and perceived social support. Drug Alcohol Depend. 2016 Dec;169:56–63.

169. Han BH, Sherman S, Mauro PM, Martins SS, Rotenberg J, Palamar JJ. Demographic trends among older cannabis users in the United States, 2006-13: Cannabis use among older adults. Addiction. 2017 Mar;112(3):516–25.

170. Han BH, Palamar JJ. Marijuana use by middle-aged and older adults in the United States, 2015–2016. Drug Alcohol Depend. 2018 Oct;191:374–81.

171. Katz I, Katz D, MaACR YS. Clinical Evidence for Utilizing Cannabinoids in the Elderly. 2017;19:5.

172. McGowan J, Sampson M, Salzwedel DM, Cogo E, Foerster V, Lefebvre C. PRESS Peer Review of Electronic Search Strategies: 2015 Guideline Statement. J Clin Epidemiol. 2016 Jul;75:40–6.

173. O’Blenis P. One Simple Way To Speed Up Your Screening Process [Internet]. 2017 [cited 2019 May 21]. Available from: https://blog.evidencepartners.com/one-simple-way-to-speed-up-your-screening-process

174. Arksey H, O’Malley L. Scoping studies: towards a methodological framework. Int J Soc Res Methodol. 2005 Feb 1;8(1):19–32.

175. Wan X, Wang W, Liu J, Tong T. Estimating the sample mean and standard deviation from the sample size, median, range and/or interquartile range. BMC Med Res Methodol. 2014 Dec;14(1):135.

176. Bega D, Gonzalez-Latapi P, Zadikoff C, Simuni T. A Review of the Clinical Evidence for Complementary and Alternative Therapies in Parkinson’s Disease. Curr Treat Options Neurol. 2014 Oct;16(10):314.

177. Koppel BS, Brust JCM, Fife T, Bronstein J, Youssof S, Gronseth G, et al. Systematic review: efficacy and safety of medical marijuana in selected neurologic disorders: report of the Guideline Development Subcommittee of the American Academy of Neurology. Neurology. 2014 Apr 29;82(17):1556–63.

178. Krishnan S, Cairns R, Howard R. Cannabinoids for the treatment of dementia. Cochrane Database Syst Rev. 2009 Apr 15;(2):CD007204.

179. Mücke M, Carter C, Cuhls H, Prüß M, Radbruch L, Häuser W. [Cannabinoids in palliative care: Systematic review and meta-analysis of efficacy, tolerability and safety]. Schmerz Berl Ger. 2016 Feb;30(1):25–36.

180. Campbell FA, Tramèr MR, Carroll D, Reynolds DJ, Moore RA, McQuay HJ. Are cannabinoids an effective and safe treatment option in the management of pain? A qualitative systematic review. BMJ. 2001 Jul 7;323(7303):13–6.

181. Martín-Sánchez E, Furukawa TA, Taylor J, Martin JLR. Systematic Review and Meta-analysis of Cannabis Treatment for Chronic Pain. Pain Med. 2009 Nov;10(8):1353–68.

182. Lobos Urbina D, Peña Durán J. Are cannabinoids effective for treatment of pain in patients with active cancer? Medwave. 2016 Sep 14;16(Suppl3):e6539–e6539.

183. Tateo S. State of the evidence: Cannabinoids and cancer pain—A systematic review. J Am Assoc Nurse Pract. 2017 Feb;29(2):94–103.

184. Jochimsen PR, Lawton RL, VerSteeg K, Noyes R. Effect of benzopyranoperidine, a delta-9-THC congener, on pain. Clin Pharmacol Ther. 1978 Aug;24(2):223–7.

185. Noyes R, Brunk SF, Avery DA, Canter AC. The analgesic properties of delta-9-tetrahydrocannabinol and codeine. Clin Pharmacol Ther. 1975 Jul;18(1):84–9.

186. Carley DW, Prasad B, Reid KJ, Malkani R, Attarian H, Abbott SM, et al. Pharmacotherapy of Apnea by Cannabimimetic Enhancement, the PACE Clinical Trial: Effects of Dronabinol in Obstructive Sleep Apnea. Sleep. 2018 01;41(1).

187. Efird JT, Friedman GD, Sidney S, Klatsky A, Habel LA, Udaltsova NV, et al. The Risk for Malignant Primary Adult-Onset Glioma in a Large, Multiethnic, Managed-Care Cohort: Cigarette Smoking and Other Lifestyle Behaviors. J Neurooncol. 2004 May;68(1):57–69.

188. Noyes R, Brunk SF, Baram DA, Canter A. Analgesic effect of delta-9-tetrahydrocannabinol. J Clin Pharmacol. 1975 Mar;15(2–3):139–43.

189. Chagas MHN, Eckeli AL, Zuardi AW, Pena-Pereira MA, Sobreira-Neto MA, Sobreira ET, et al. Cannabidiol can improve complex sleep-related behaviours associated with rapid eye movement sleep behaviour disorder in Parkinson’s disease patients: a case series. J Clin Pharm Ther. 2014 Oct;39(5):564–6.

190. Snider SR, Consroe P. Beneficial and adverse effects of cannabidiol in a Parkinson patient with sinemet‐induced dystonic dyskinesia. Neurology. 1985;35:201.

191. Zuardi AW, Crippa JAS, Hallak JEC, Pinto JP, Chagas MHN, Rodrigues GGR, et al. Cannabidiol for the treatment of psychosis in Parkinson’s disease. J Psychopharmacol (Oxf). 2009 Nov;23(8):979–83.

192. Mücke M, Weier M, Carter C, Copeland J, Degenhardt L, Cuhls H, et al. Systematic review and meta-analysis of cannabinoids in palliative medicine: Cannabinoids in palliative medicine. J Cachexia Sarcopenia Muscle. 2018 Apr;9(2):220–34.

193. Seeling W, Kneer L, Büchele B, Gschwend JE, Maier L, Nett C, et al. [Delta(9)-tetrahydrocannabinol and the opioid receptor agonist piritramide do not act synergistically in postoperative pain]. Anaesthesist. 2006 Apr;55(4):391–400.

194. Lissoni P, Porro G, Messina G, Porta E, Rovelli F, Roselli M. Morphine, melatonin, Marijuana, Magnolia and MYRRH as the “five m” schedule in the treatment of cancer pain and the possible dose-dependency of the antitumor and analgesic effects of the pineal hormone melatonin. Anticancer Res. 2014;34:6033–4.

195. Nugent SM, Morasco BJ, O’Neil ME, Freeman M, Low A, Kondo K, et al. The Effects of Cannabis Among Adults With Chronic Pain and an Overview of General Harms: A Systematic Review. Ann Intern Med. 2017 Sep 5;167(5):319–31.

196. Johnson JR, Lossignol D, Burnell-Nugent M, Fallon MT. An open-label extension study to investigate the long-term safety and tolerability of THC/CBD oromucosal spray and oromucosal THC spray in patients with terminal cancer-related pain refractory to strong opioid analgesics. J Pain Symptom Manage. 2013 Aug;46(2):207–18.

197. van den Elsen G a. H, Ahmed AIA, Lammers M, Kramers C, Verkes RJ, van der Marck MA, et al. Efficacy and safety of medical cannabinoids in older subjects: a systematic review. Ageing Res Rev. 2014 Mar;14:56–64.
