# Supplementary material for: Impacts of medical and non-medical cannabis on the health of older adults: Findings from a scoping review of the literature
Source: PLoS One. 2023 Feb 17;18(2):e0281826. doi: 10.1371/journal.pone.0281826 (PMC9937508; doi:10.1371/journal.pone.0281826)
Supplement: S7 Text — (DOCX) [file pone.0281826.s010.docx]

S7 Text: Effect Direction Plots – Healthy Adults and General Population

Summaries in this appendix present findings for studies in healthy adults and the general population according to outcome and nature of effect (including direction and statistical significance). Findings for cross-sectional and sequential studies should be interpreted as associations not effects. Cells split into two colours indicate more than one analysis for the outcome with differing findings. These summaries are intended to provide a high-level comprehensive mapping of available data for this sub-population.

**Non-randomized Studies of Healthy Older Adults and the Older General Public: Physical and Mental Health Outcomes**

**
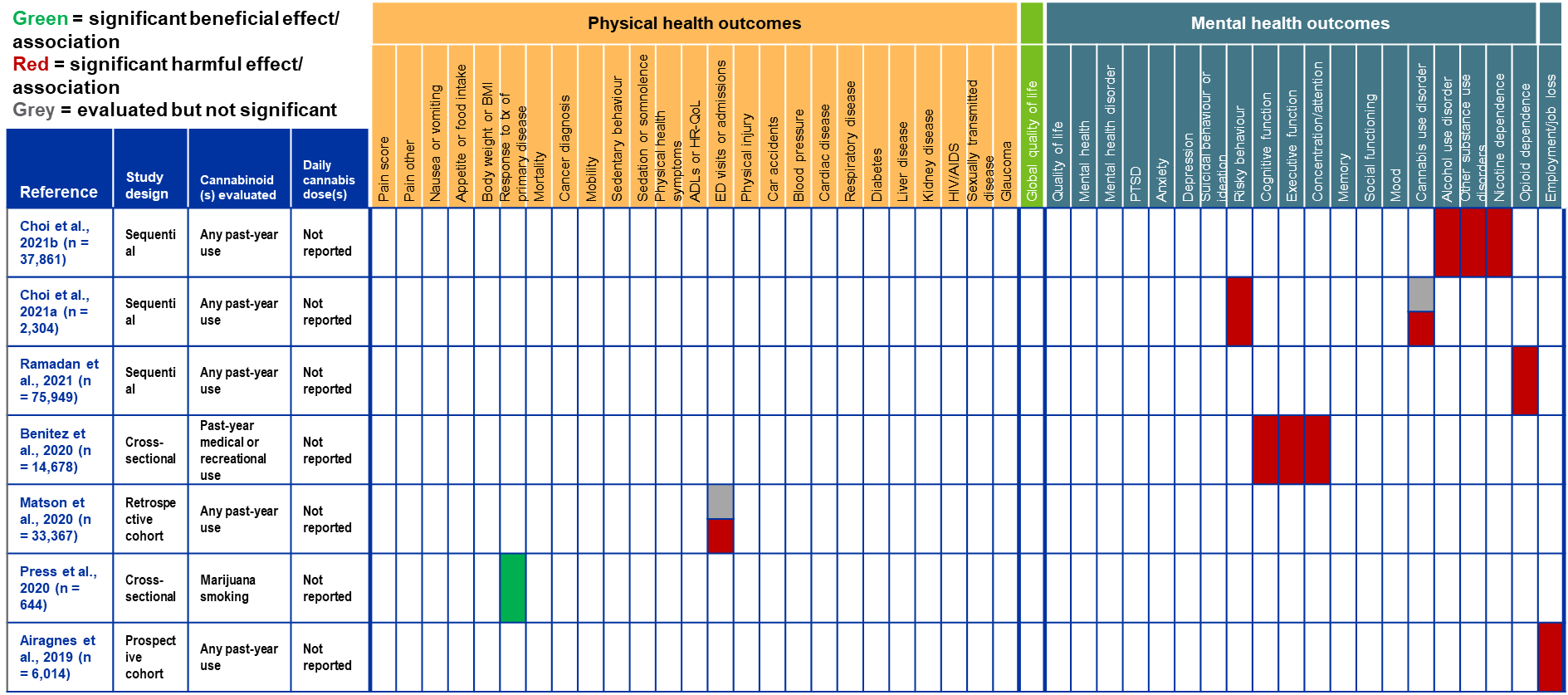
**

**Non-randomized Studies of Healthy Older Adults and the Older General Public: Physical and Mental Health Outcomes (continued)**

**
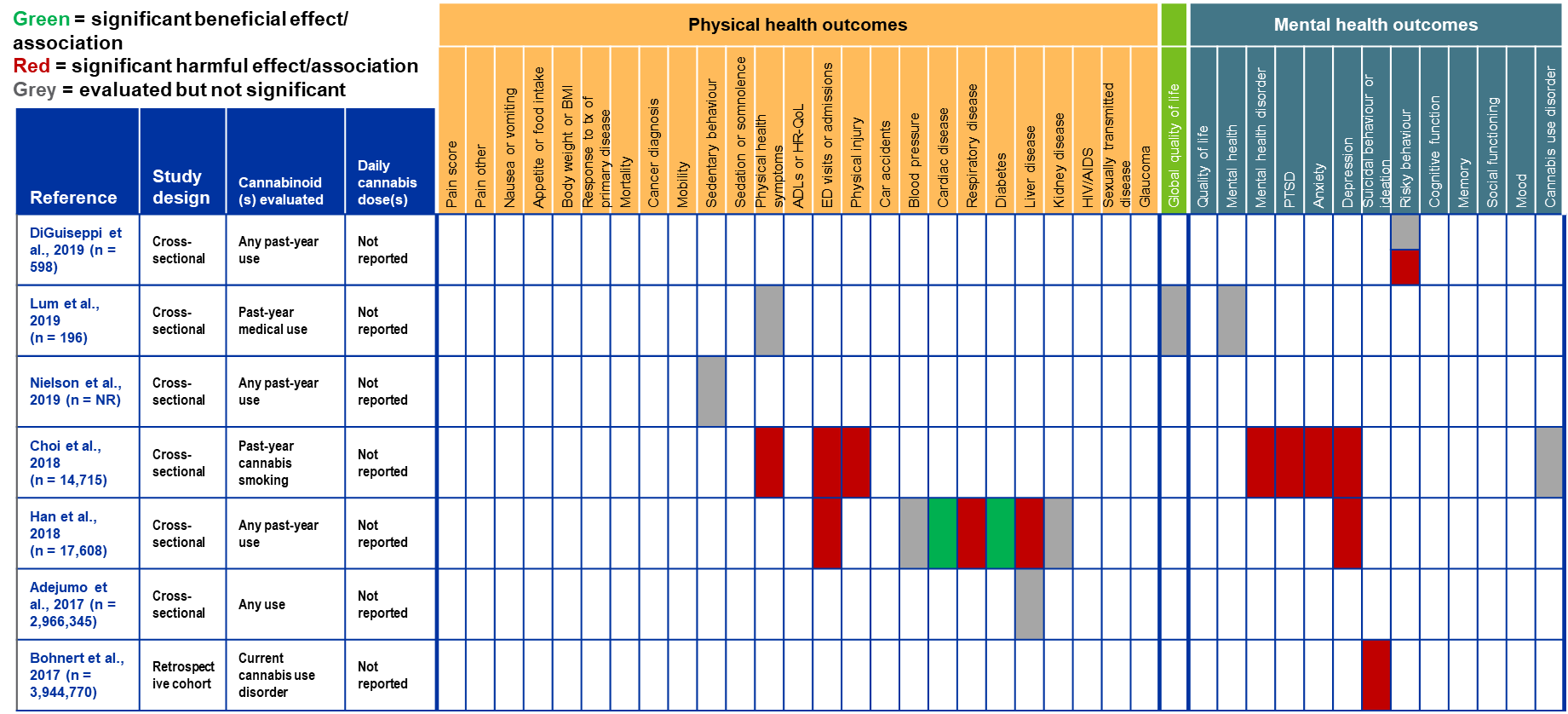
**

**Non-randomized Studies of Healthy Older Adults and the Older General Public: Physical and Mental Health Outcomes (continued)**

**
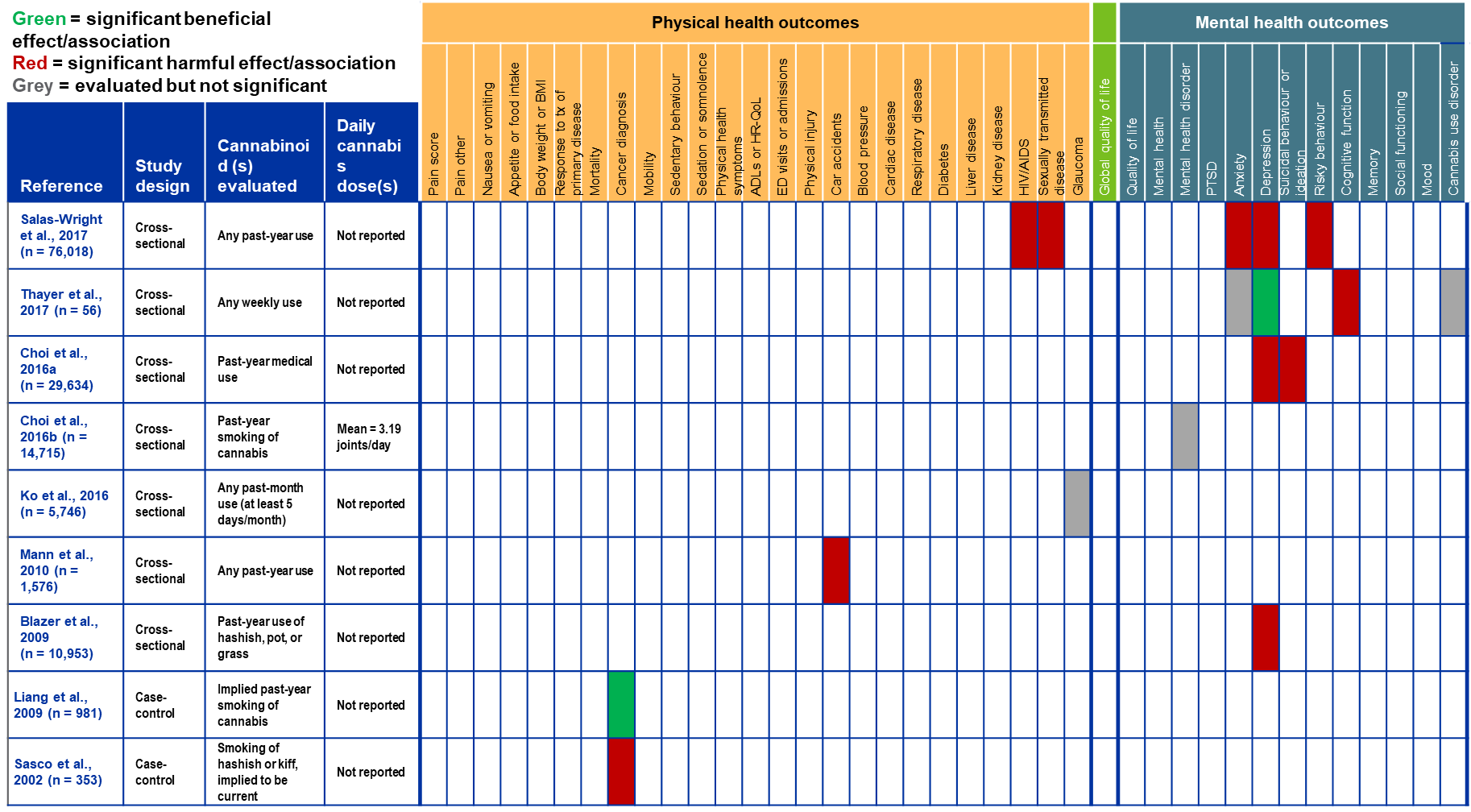
**

**Non-randomized Studies of Healthy Older Adults and the Older General Public: Drug and Alcohol Outcomes**

**
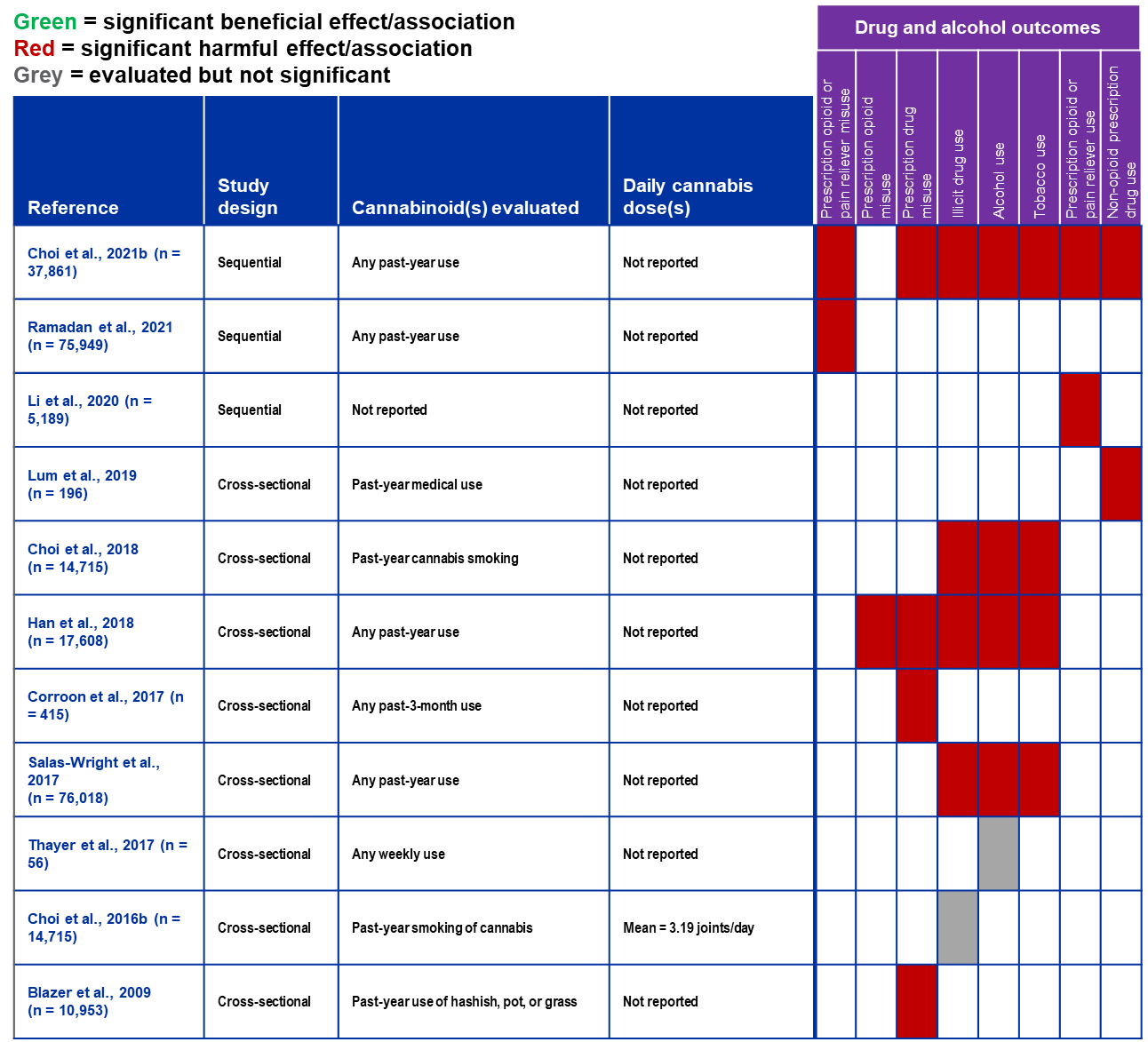
**
