# Supplementary material for: Impacts of medical and non-medical cannabis on the health of older adults: Findings from a scoping review of the literature
Source: PLoS One. 2023 Feb 17;18(2):e0281826. doi: 10.1371/journal.pone.0281826 (PMC9937508; doi:10.1371/journal.pone.0281826)
Supplement: S10 Text — (DOCX) [file pone.0281826.s013.docx]

S10 Text: Effect Direction Plots – Parkinson’s Disease

Summaries in this appendix present findings for studies in patients with Parkinson’s Disease according to outcome and nature of effect (including direction and statistical significance). Findings for cross-sectional and sequential studies should be interpreted as associations not effects. Cells split into two colours indicate more than one analysis for the outcome with differing findings. These summaries are intended to provide a high-level comprehensive mapping of available data for this sub-population.

**Systematic Reviews of Parkinson’s Disease: All Outcomes**


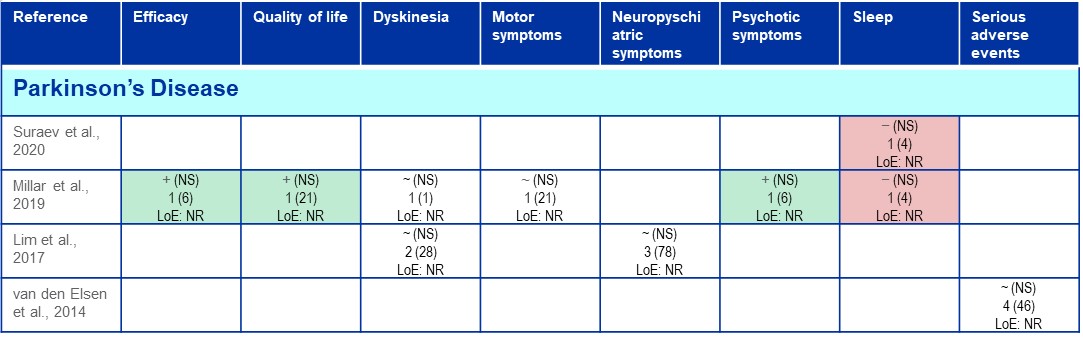


**RCTs of Parkinson’s Disease: All Outcomes**


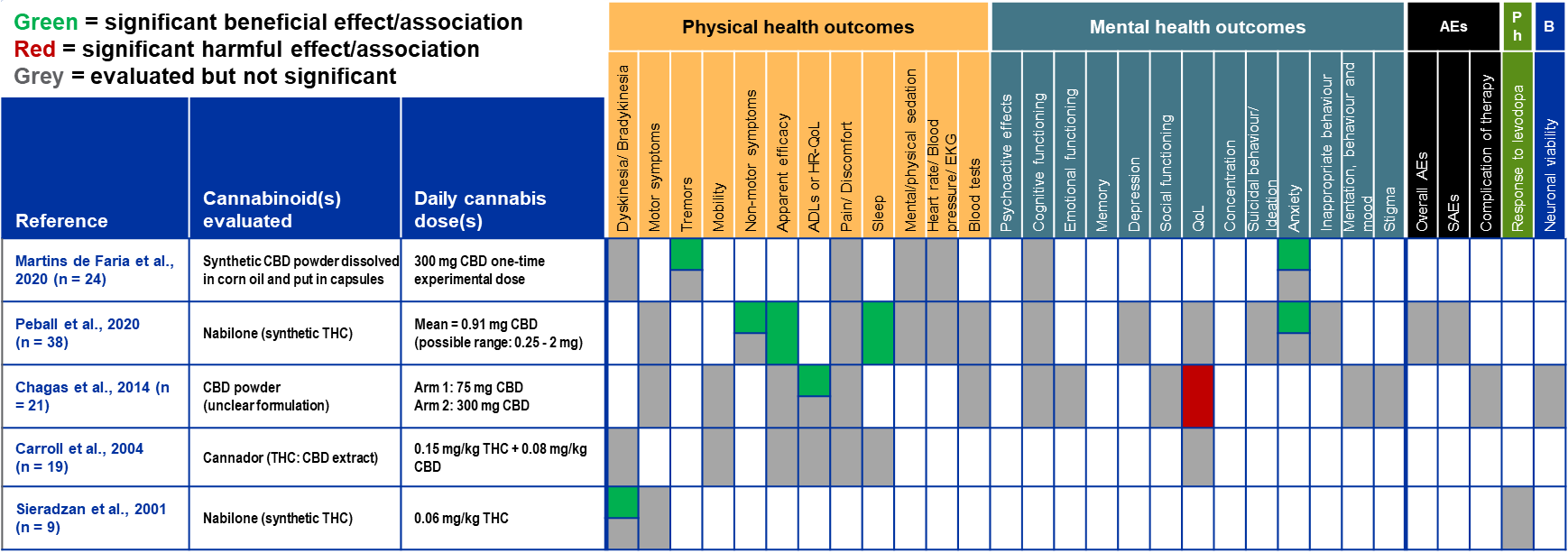


**Non-randomized Studies of Parkinson’s Disease: All Outcomes**

**
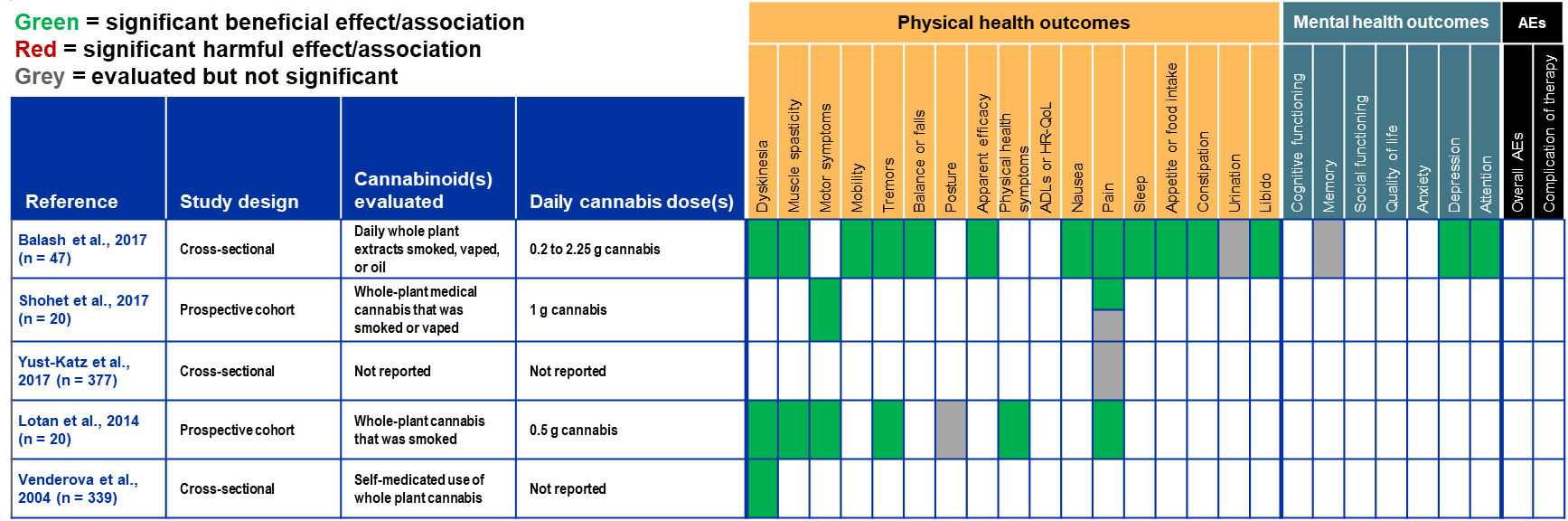
**
