# Supplementary material for: Impacts of medical and non-medical cannabis on the health of older adults: Findings from a scoping review of the literature
Source: PLoS One. 2023 Feb 17;18(2):e0281826. doi: 10.1371/journal.pone.0281826 (PMC9937508; doi:10.1371/journal.pone.0281826)

S11 Text: Effect Direction Plots, Other Patient Conditions

Summaries in this appendix present findings for studies in patients with other conditions (other than end stage cancer, dementia and Parkinson’s disease) according to outcome and nature of effect (including direction and statistical significance). Findings for cross-sectional and sequential studies should be interpreted as associations not effects. Cells split into two colours indicate more than one analysis for the outcome with differing findings. These summaries are intended to provide a high-level comprehensive mapping of available data for this sub-population.

**RCTs of Other Patient Conditions: All Outcomes**


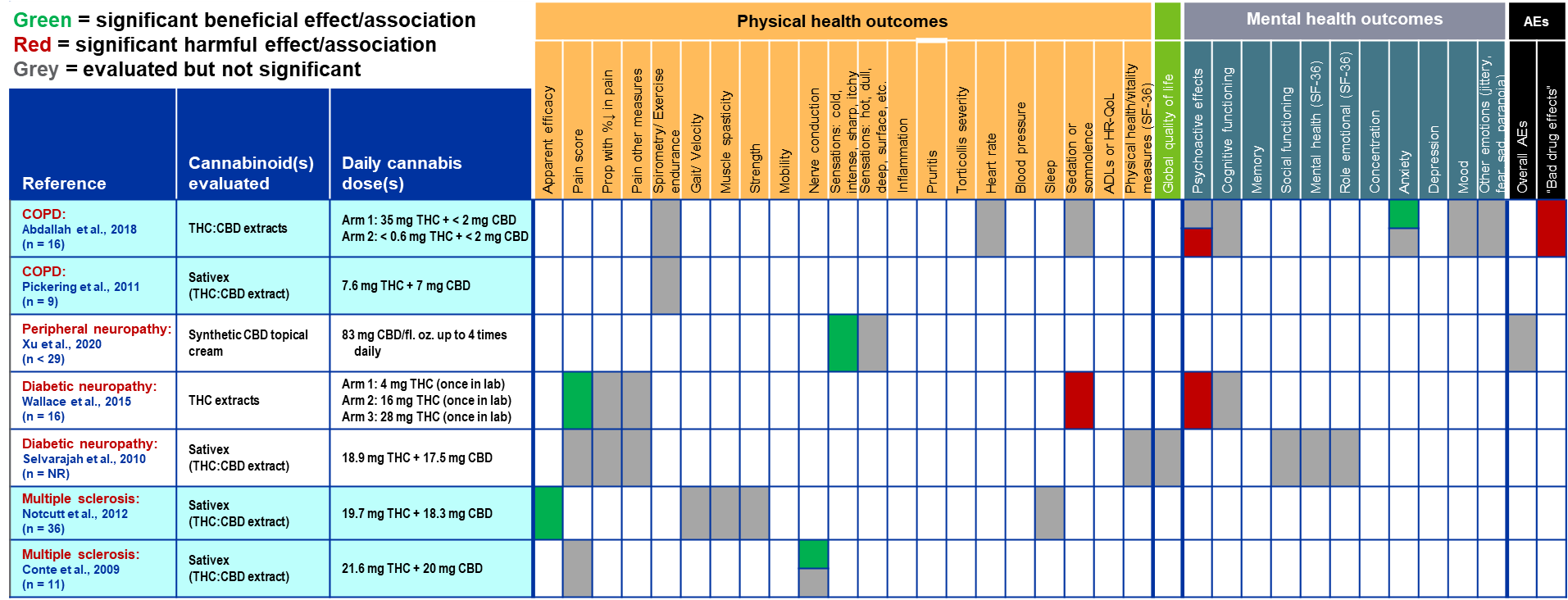


**RCTs of Other Patient Conditions: All Outcomes (continued)**


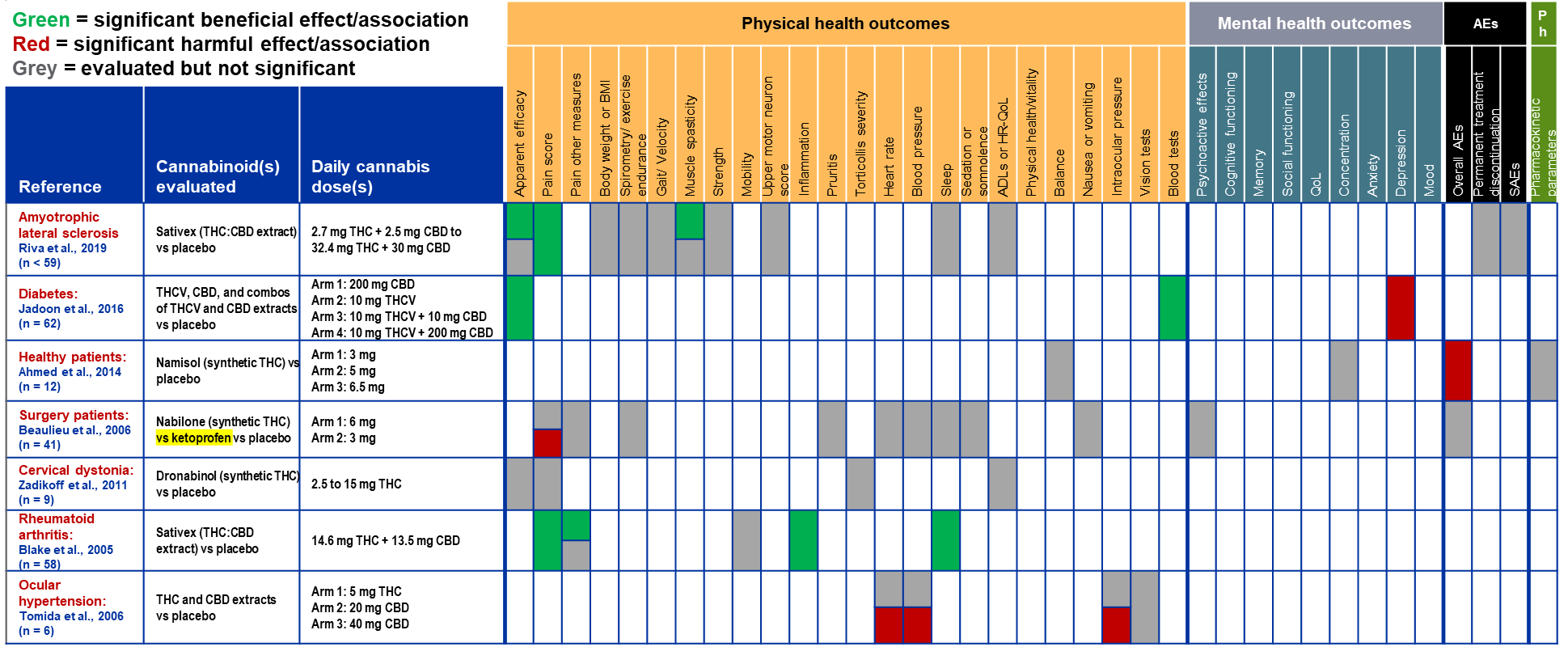


**Non-randomized Studies of Other Patient Conditions: Physical and Mental Health Outcomes**


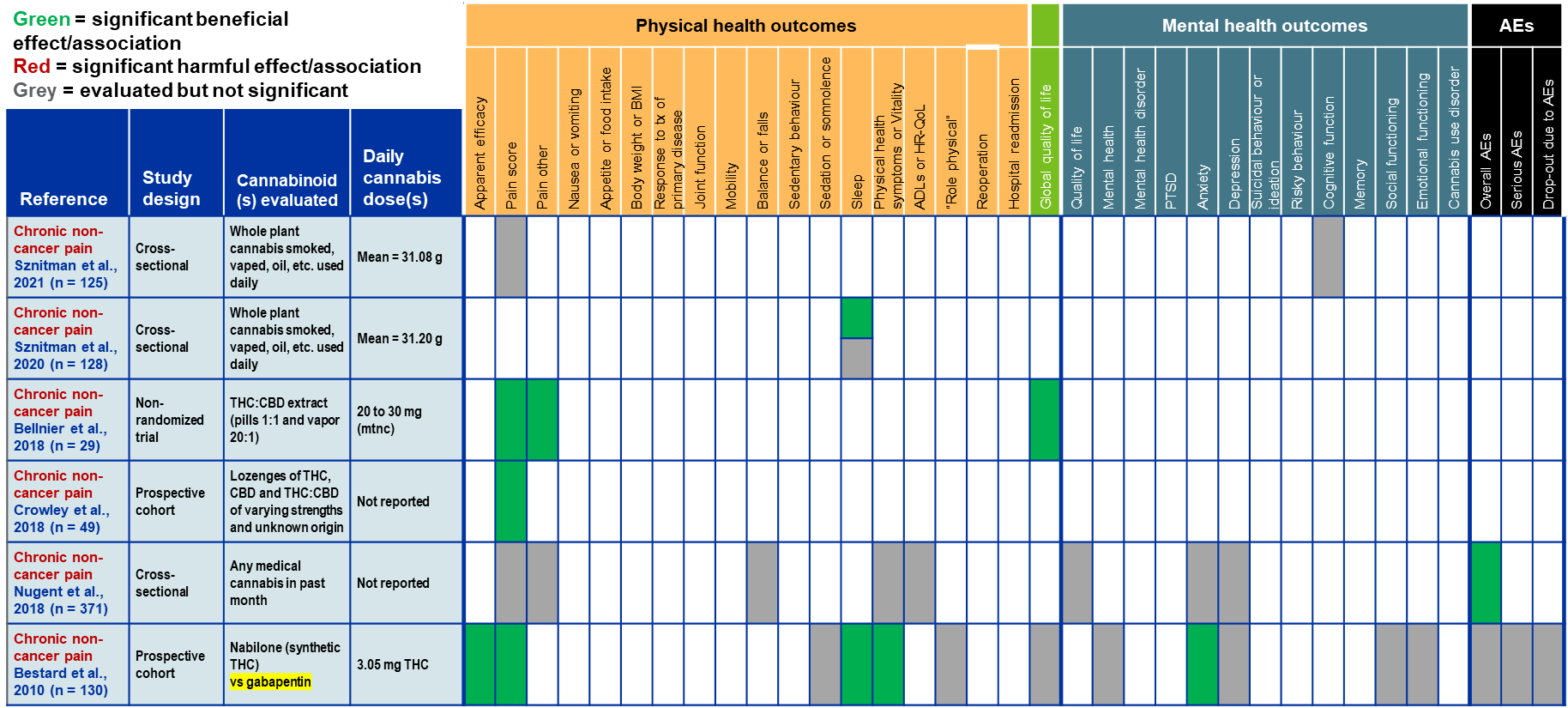


Non-randomized Studies of Other Patient Conditions: Physical and Mental Health Outcomes (continued)


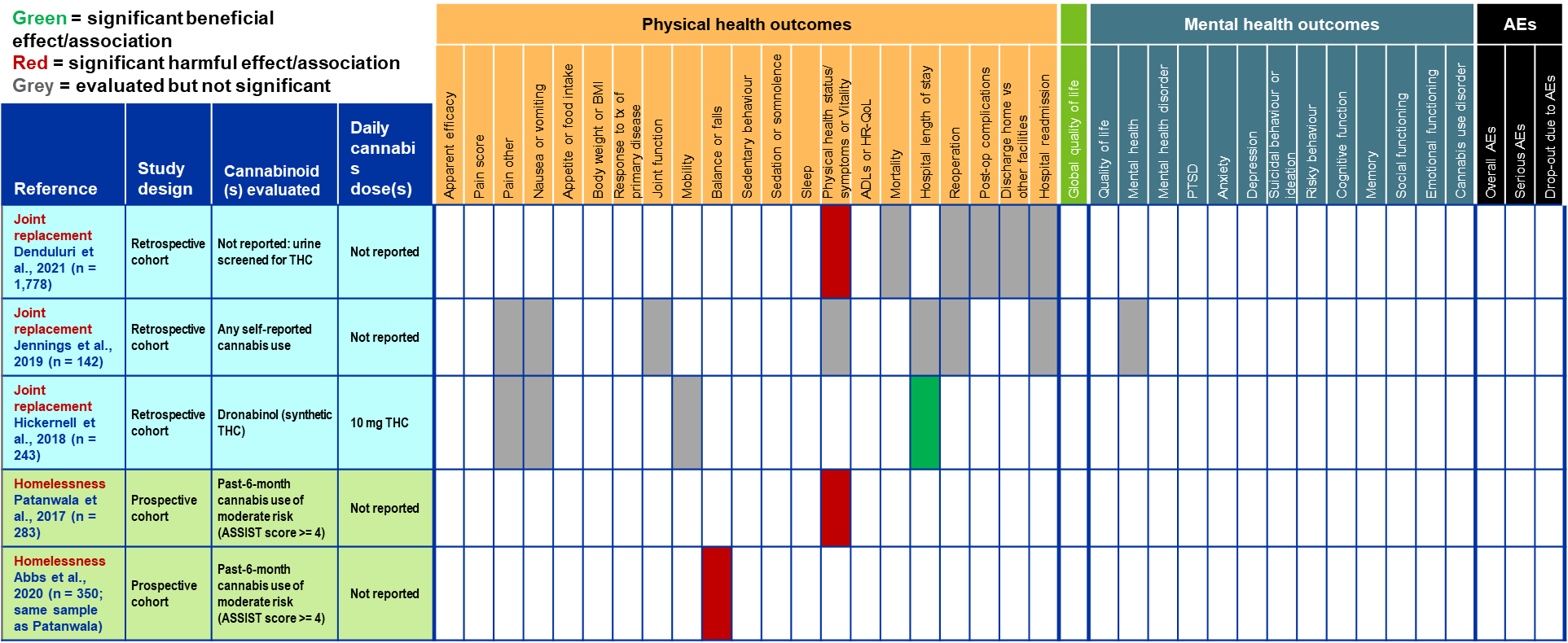


**Non-randomized Studies of Other Patient Conditions: Physical and Mental Health Outcomes (continued)**


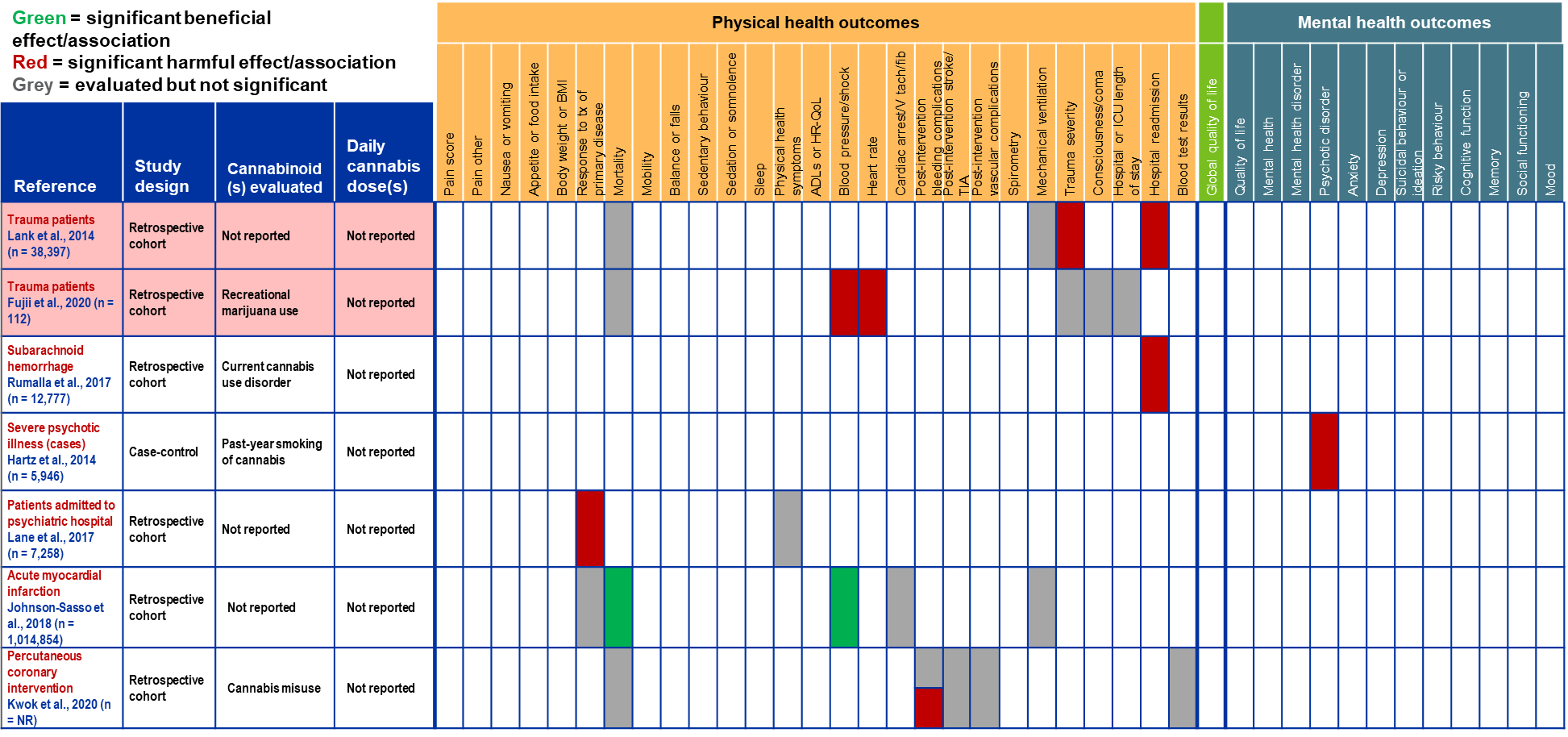


**Non-randomized Studies of Other Patient Conditions: Physical and Mental Health Outcomes (continued)**


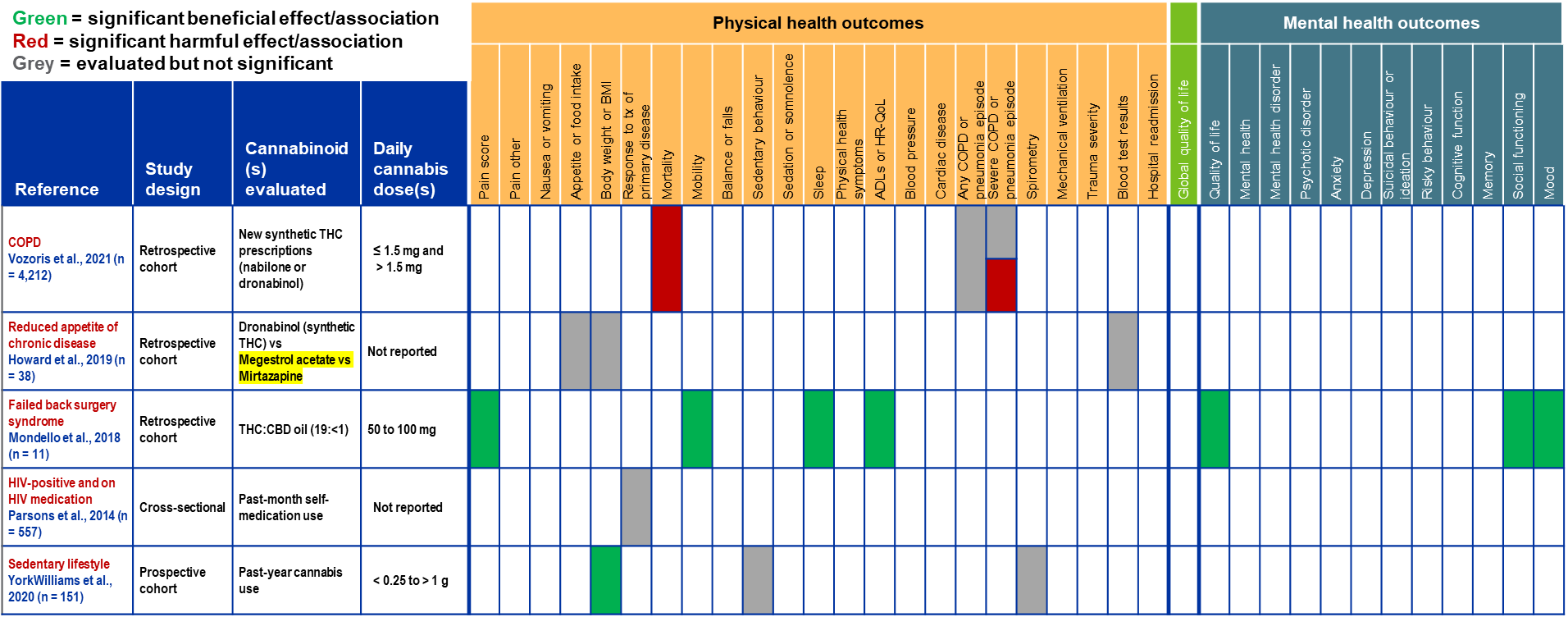


**Non-randomized Studies of Other Patient Conditions: Physical and Mental Health Outcomes (continued)**


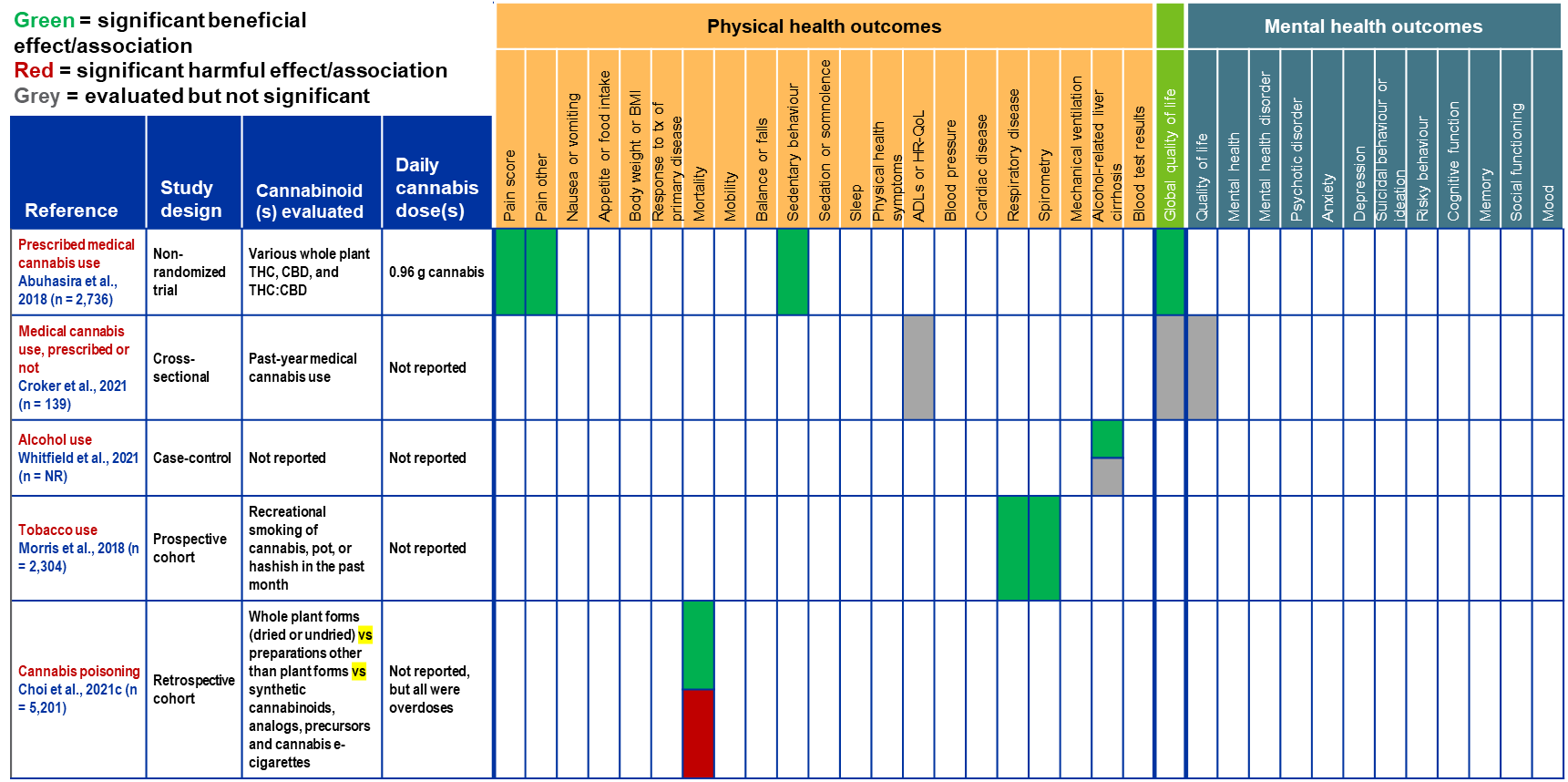


**Non-randomized Studies of Other Patient Conditions: Drug and Alcohol Outcomes**


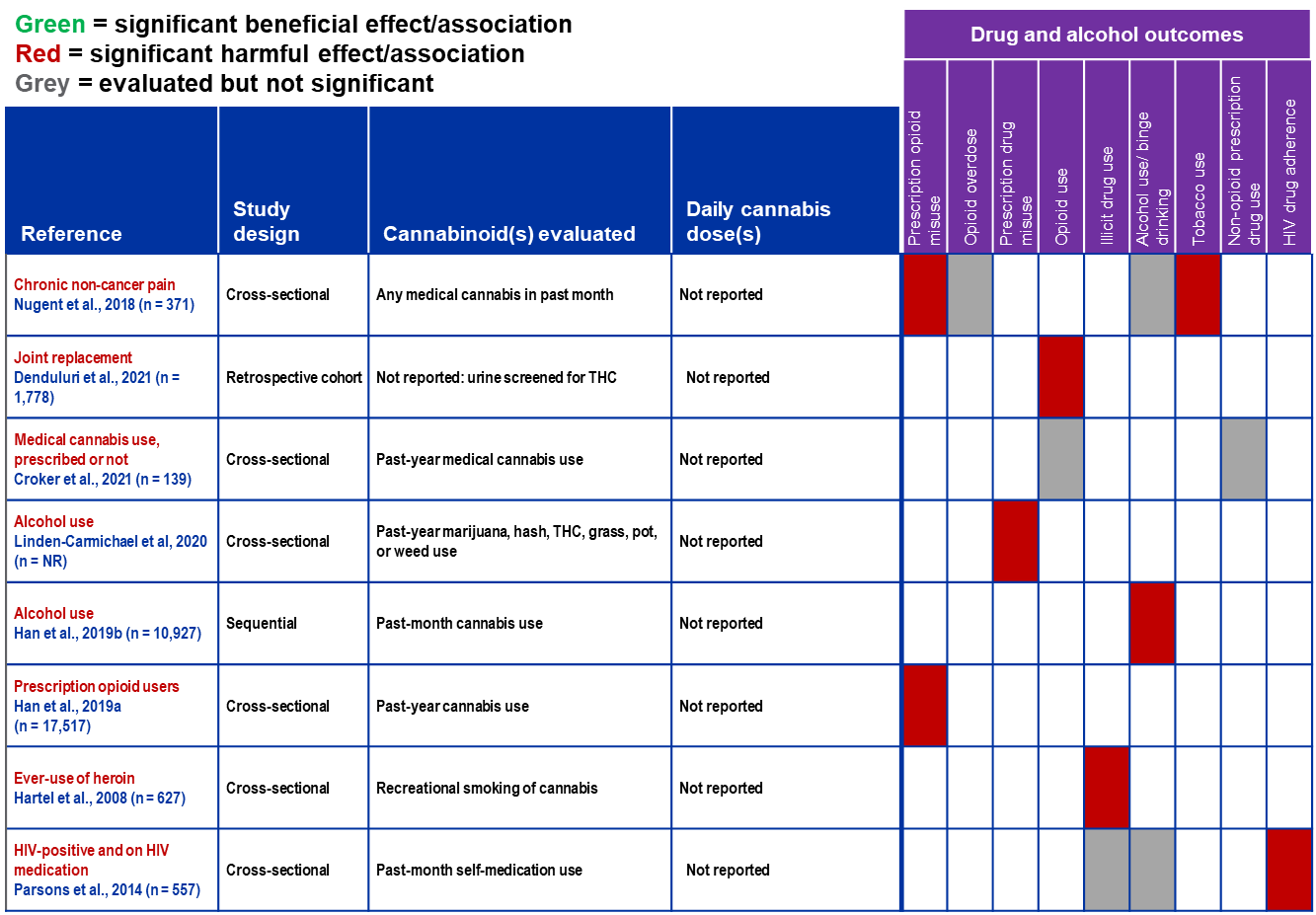

Supplement: S11 Text — (DOCX) [file pone.0281826.s014.docx]
