# Supplementary material for: Impacts of medical and non-medical cannabis on the health of older adults: Findings from a scoping review of the literature
Source: PLoS One. 2023 Feb 17;18(2):e0281826. doi: 10.1371/journal.pone.0281826 (PMC9937508; doi:10.1371/journal.pone.0281826)
Supplement: S12 Text — (DOCX) [file pone.0281826.s015.docx]

S12 Text: Summary of Available Subgroup Data

Data related to our a priori clinical subpopulations of interest were limited, although some data were found related to subgroups of sex, older adult age group (i.e., 50–64 years, 65+ years, etc.), residential setting (e.g., ambulatory versus inpatient care), and illicit drug use. A narrative summary of the subgroup data, with an accompanying series of detailed tables have been provided below. The following findings were noted, stratified by patient condition:

*Older general public:*

- ***Age:*** Cannabis was significantly and positively associated with the following outcomes for adults aged 50–64 years but not for those ≥ 65 years: ED visits, liver cirrhosis, COPD, attacking another person to harm, drug selling, and theft^115,145^. It’s unclear if smaller sample sizes in the ≥ 65 years age group may have reduced the power to detect a significant cannabis effect. However, a significant negative association of cannabis was found for the ≥ 65 years group with cardiac disease, which was non-significant for those 50–64 years^115^, and a significant positive association was reported in both age categories for many other negative health outcomes^115,145^. Medical cannabis use was reported to significantly increase the risk of persons 51–65 years of age substituting cannabis for another prescription drug over those who used cannabis for non-medical purposes^108^.
- ***Illicit drug use:*** When individuals who used medical cannabis and other illicit drugs were compared to those who did not use any substance, they were significantly more likely to have suicidal thoughts in adjusted analyses^104^, but no significant associations were found when they were compared to those who used other illicit drugs or when those using medical cannabis only were compared to either those who used no drugs or those who used other illicit drugs, respectively^104^.

*End-stage cancer:*

- ***Sex:*** women had significantly more “highs” than men after taking THC^70^, and daily appetite scores were improved for both cannabis products over placebo, although significance was not reported^63^.

*Alzheimer’s disease/dementia:*

- ***Residential setting:*** The effects of 4.5 mg namisol on agitation and neuropsychiatric symptoms, respectively, did not differ by whether the patient was in ambulatory care or an inpatient^85,86^.

*Use of health services:*

- ***Sex:*** Compared to older adults who used cannabis without CUD, male and female cannabis consumers with CUD were significantly more likely to commit suicide in multivariable models; however, the effect in women became non-significant, when further adjustments for psychiatric diagnoses were made^102^
- ***Age:*** Daily or almost daily cannabis use was associated with a significantly elevated risk of acute care use compared to those who did not use cannabis, in those 50–64 years but not in those ≥ 65 years^134^.

*Use of alcohol:*

- ***Sex:*** In both men and women, co-use of alcohol and cannabis was associated with a significantly greater risk of problematic prescription drug use compared to use of either substance alone^129^.
- ***Age:*** Cannabis use was associated with a significantly lower risk of alcohol-related liver cirrhosis in 50–59 year-olds who used alcohol, but not in those ≥ 60 years of age^156^.

*Percutaneous coronary intervention:*

- ***Age:*** Compared to those who did not use cannabis, older adults who used recreational cannabis were significantly more likely to have post-intervention bleeding complications if they were 66–75 years of age, but not if they were younger or older^124^. Significant effects of cannabis use were not found for any other post-intervention complications in any age category.

*Acute myocardial infarction:*

- ***Age:*** Older adults who used cannabis were significantly less likely to die during an acute myocardial infarction compared to those who did not, if they were 50–59 years of age, but there was no significant difference between cannabis use groups for those 60–69 years of age^122^. Significant benefits of cannabis use were also found against shock for both age categories^122^.

*Older drivers:*

- ***Age:*** Cannabis use was associated with a significantly increased risk of prescription opioid use in older adult drivers over the age of 64 years^127^.

In the series of tables below, data are presented from a priori subgroups of clinical interest as described in our protocol. Each table is focused on all available findings for a given subpopulation of interest (i.e., sex, age group amongst older adults, residential setting, and illicit drug use). Effects reported in the studies are presented in the right-most column, with color coding used to reflect the direction of effect (i.e., green for beneficial and red for harmful effects, grey for no effect) and significance (i.e., dark for significant and light for non-significant effects).

Table: Sex subgroup data

| Patient condition | Study | Outcome category | Outcome definition | Comparison | Analysis type | Subgroup stratum | Direction of effect and significance |
| --- | --- | --- | --- | --- | --- | --- | --- |
| **End-stage cancer** | Strasser et al., 2006^63^  RCT | Appetite | Daily appetite VAS scores | Arm 1: 5 mg THC + 2 mg CBD  Arm 2: 5 mg THC  Arm 3: placebo | Randomized, univariable | Women | Both arms beneficial over placebo, significance not reported |
|  | Frytak et al., 1979^70^  RCT | Psychoactive effects | “Highs” | 15 mg THC | Chi-squared test | Women vs men | Women had significantly more highs than men |
| **Use of health services** | Bohnert et al., 2017^102^  Retrospective cohort | Suicidal behaviour or ideation | Committed suicide | CUD vs no CUD | Multivariable: age and comorbidity index | Women | Significantly harmful |
|  |  |  |  |  |  | Men | Significantly harmful |
|  |  | Suicidal behaviour or ideation | Committed suicide | CUD vs no CUD | Multivariable: age, comorbidity index, and psychiatric diagnoses | Women | Harmful |
|  |  |  |  |  |  | Men | Significantly harmful |
| **Alcohol use** | Linden-Carmichael et al., 2020^129^  Cross-sectional | Problematic prescription drug use | Past-year problematic prescription drug use | Co-use (alcohol + cannabis) vs single-substance use (either alcohol or cannabis) | Multivariable | Women | Significant positive association |
|  |  |  |  |  |  | Men | Significant positive association |

Table. Age subgroup data

| Patient condition | Study | Outcome category | Outcome definition | Comparison | Analysis type | Subgroup stratum | Direction of effect and significance |
| --- | --- | --- | --- | --- | --- | --- | --- |
| **Older general public** | Han et al., 2018^115^  Sequential NSDUH data 2015–16 | Blood pressure | Hypertension | Marijuana use vs no use | Univariable | 50–64 years | Positive association |
|  |  |  |  |  |  | 65+ years | Negative association |
|  |  | Cardiac disease | Heart condition | Marijuana use vs no use | Univariable | 50–64 years | Positive association |
|  |  |  |  |  |  | 65+ years | Significant negative association |
|  |  | Diabetes | Diabetes | Marijuana use vs no use | Univariable | 50–64 years | Significant negative association |
|  |  |  |  |  |  | 65+ years | Significant negative association |
|  |  | ED visits | Past-year all-cause emergency department visits | Marijuana use vs no use | Univariable | 50–64 years | Significant positive association |
|  |  |  |  |  |  | 65+ years | Negative association |
|  |  | Kidney disease | Kidney disease | Marijuana use vs no use | Univariable | 50–64 years | Negative association |
|  |  |  |  |  |  | 65+ years | Positive association |
|  |  | Liver disease | Cirrhosis | Marijuana use vs no use | Univariable | 50–64 years | Significant positive association |
|  |  |  |  |  |  | 65+ years | Negative association |
|  |  |  | Hepatitis B or C | Marijuana use vs no use | Univariable | 50–64 years | Significant positive association |
|  |  |  |  |  |  | 65+ years | Significant positive association |
|  |  | Respiratory disease | Asthma | Marijuana use vs no use | Univariable | 50–64 years | Negative association |
|  |  |  |  |  |  | 65+ years | Positive association |
|  |  |  | COPD | Marijuana use vs no use | Univariable | 50–64 years | Significant positive association |
|  |  |  |  |  |  | 65+ years | Positive association |
|  |  | Depression | Past-year major depressive episode based on DSM-IV | Marijuana use vs no use | Univariable | 50–64 years | Significant positive association |
|  |  |  |  |  |  | 65+ years | Significant positive association |
|  |  | Alcohol use | Past-year major alcohol use disorder based on DSM-IV | Marijuana use vs no use | Univariable | 50–64 years | Significant positive association |
|  |  |  |  |  |  | 65+ years | Significant positive association |
|  |  | Illicit drug use | Past-year cocaine use | Marijuana use vs no use | Univariable | 50–64 years | Significant positive association |
|  |  |  |  |  |  | 65+ years | Significant positive association |
|  |  | Nicotine use | Nicotine dependence | Marijuana use vs no use | Univariable | 50–64 years | Significant positive association |
|  |  |  |  |  |  | 65+ years | Significant positive association |
|  |  | Problematic prescription drug use | Problematic past-year opioid use | Marijuana use vs no use | Univariable | 50–64 years | Significant positive association |
|  |  |  |  |  |  | 65+ years | Significant positive association |
|  |  |  | Problematic past-year sedative use | Marijuana use vs no use | Univariable | 50–64 years | Significant positive association |
|  |  |  |  |  |  | 65+ years | Significant positive association |
|  |  |  | Problematic past-year tranquilizer use | Marijuana use vs no use | Univariable | 50–64 years | Significant positive association |
|  |  |  |  |  |  | 65+ years | Significant positive association |
|  | Salas-Wright et al., 2017^145^  Sequential NSDUH data 2002–14 | HIV/AIDS | Past-year HIV/AIDS diagnosed by a doctor | Cannabis use vs no use | Multivariable | 50–64 years | Significant positive association |
|  |  |  |  |  |  | 50–64 years | Significant positive association |
|  |  | Sexually transmitted disease | Past-year sexually transmitted disease diagnosed by a doctor | Cannabis use vs no use | Multivariable | 65+ years | Significant positive association |
|  |  | Anxiety | Past-year anxiety diagnosed by a doctor | Cannabis use vs no use | Multivariable | 50–64 years | Significant positive association |
|  |  |  |  |  |  | 65+ years | Significant positive association |
|  |  | Depression | Past-year depression diagnosed by a doctor | Cannabis use vs no use | Multivariable | 50–64 years | Significant positive association |
|  |  |  |  |  |  | 65+ years | Significant positive association |
|  |  | Risky behaviour | Arrest/criminal justice system involvement | Cannabis use vs no use | Multivariable | 50–64 years | Significant positive association |
|  |  |  |  |  |  | 65+ years | Significant positive association |
|  |  |  | Attack another person to harm | Cannabis use vs no use | Multivariable | 50–64 years | Significant positive association |
|  |  |  |  |  |  | 65+ years | Positive association |
|  |  |  | Driving under the influence | Cannabis use vs no use | Multivariable | 50–64 years | Significant positive association |
|  |  |  |  |  |  | 65+ years | Significant positive association |
|  |  |  | Drug selling | Cannabis use vs no use | Multivariable | 50–64 years | Significant positive association |
|  |  |  |  |  |  | 65+ years | Positive association |
|  |  |  | Risk propensity index | Cannabis use vs no use | Multivariable | 50–64 years | Significant positive association |
|  |  |  |  |  |  | 65+ years | Significant positive association |
|  |  |  | Theft | Cannabis use vs no use | Multivariable | 50–64 years | Significant positive association |
|  |  |  |  |  |  | 65+ years | Positive association |
|  |  | Problematic alcohol use | Past-month binge alcohol use (≥ 5 drinks on the same occasion) | Cannabis use vs no use | Multivariable | 50–64 years | Significant positive association |
|  |  |  |  |  |  | 65+ years | Significant positive association |
|  |  | Illicit drug use | Past-year use of illicit drugs other than marijuana | Cannabis use vs no use | Multivariable | 50–64 years | Significant positive association |
|  |  |  |  |  |  | 65+ years | Significant positive association |
|  |  | Nicotine use | Past-year use of tobacco | Cannabis use vs no use | Multivariable | 65+ years | Significant positive association |
|  | Corroon et al., 2017^108^  Cross-sectional | Problematic prescription drug use | Substituting cannabis for a prescription drug | Medical cannabis either overseen by a physician or self-medicated vs non-medical | Univariable | 51–65 years | Significant positive association |
| **Percutaneous coronary intervention** | Kwok et al., 2020^124^  Retrospective cohort | In-hospital complications | Bleeding | Non-medical/ recreational cannabis use vs no use | Chi-squared test | 56–65 years | Beneficial |
|  |  |  |  |  |  | 66–75 years | Significantly harmful |
|  |  |  |  |  |  | 75+ years | Harmful |
|  |  |  | In-hospital stroke/TIA | Non-medical/ recreational cannabis use vs no use | Chi-squared test | 56–65 years | Harmful |
|  |  |  |  |  |  | 66–75 years | Harmful |
|  |  |  |  |  |  | 75+ years | Harmful |
|  |  |  | In-hospital vascular complications | Non-medical/ recreational cannabis use vs no use | Chi-squared test | 56–65 years | Beneficial |
|  |  |  |  |  |  | 66–75 years | Harmful |
|  |  |  |  |  |  | 75+ years | Beneficial |
|  |  |  | In-hospital death | Non-medical/ recreational cannabis use vs no use | Chi-squared test | 56–65 years | Harmful |
|  |  |  |  |  |  | 66–75 years | Harmful |
|  |  |  |  |  |  | 75+ years | Harmful |
| **Acute myocardial infarction** | Johnson-Sasso et al., 2018^122^  Retrospective cohort | Blood pressure | Shock | Cannabis use vs no use | Multivariable | 50–59 years | Significantly beneficial |
|  |  |  |  |  |  | 60–69 years | Significantly beneficial |
|  |  | Cardiac | Ventricular tachycardia/ ventricular fibrillation/ cardiac arrest | Cannabis use vs no use | Multivariable | 50–59 years | Beneficial |
|  |  |  |  |  |  | 60–69 years | Harmful |
|  |  | Mechanical ventilation | Mechanical ventilation | Cannabis use vs no use | Multivariable | 50–59 years | Harmful |
|  |  |  |  |  |  | 60–69 years | Harmful |
|  |  | Mortality |  | Cannabis use vs no use | Multivariable | 50–59 years | Significantly beneficial |
|  |  |  |  |  |  | 60–69 years | Beneficial |
|  |  | Response to treatment of another therapy | Adverse AMI outcomes: death, mechanical ventilation, cardiac arrest, intra-aortic balloon pump, shock | Cannabis use vs no use | Multivariable | 50–59 years | Beneficial |
|  |  |  |  |  |  | 60–69 years | Harmful |
| **Older drivers** | Li et al., 2020^127^  Sequential | Prescription drug use | Use of opioids available by prescription | Marijuana use vs no use | Univariable | 65+ years | Significant positive association |
| **Primary care use** | Matson et al., 2020^134^  Retrospective cohort | ED visits | Acute care use (urgent care, emergency department visit, hospital admission) | Daily/almost daily cannabis use vs no use | Multivariable | 50–64 years | Significantly harmful |
|  |  |  |  |  |  | 65+ years | No effect |
| **Alcohol use** | Whitfield et al., 2021^156^  Case-control | Liver disease | Alcohol-related cirrhosis | Cannabis use vs no use | Multivariable: % of alcohol as wine, BMI, diabetes, green tea, coffee, smoking | 50–59 years | Significantly beneficial |
|  |  |  |  |  |  | 60+ years | Beneficial |

Table. Residential setting subgroup data

| Patient condition | Study | Outcome category | Outcome definition | Comparison | Analysis type | Subgroup stratum | Direction of effect and significance |
| --- | --- | --- | --- | --- | --- | --- | --- |
| **Alzheimer’s disease/ dementia** | van den Elsen et al., 2015a^85^  RCT | Agitation | Cohen-Mansfield Agitation Inventory | 4.5 mg namisol (synthetic THC) | Randomized, univariable | Ambulatory care | Beneficial |
|  |  |  |  |  |  | Inpatient | Beneficial |
|  |  | Agitation/ aggression | NPI agitation/ aggression subscale | 4.5 mg namisol (synthetic THC) | Randomized, univariable | Ambulatory care | Beneficial |
|  |  |  |  |  |  | Inpatient | Beneficial |
|  |  | Neuropsychiatric symptoms | NPI, total score | 4.5 mg namisol (synthetic THC) | Randomized, univariable | Ambulatory care | Beneficial |
|  |  |  |  |  |  | Inpatient | Beneficial |
|  | van den Elsen et al., 2015b^86^  RCT | Neuropsychiatric symptoms | Neuropsychiatric Inventory (NPI), total score | 1.5 or 3.0 mg namisol (synthetic THC) | Randomized, multivariable | Community dwelling | Harmful |
|  |  |  |  |  |  | Inpatient | Harmful |

Table. Illicit drug use subgroup data

| Patient condition | Study | Outcome category | Outcome definition | Comparison | Analysis type | Subgroup stratum | Direction of effect and significance |
| --- | --- | --- | --- | --- | --- | --- | --- |
| **Older general public** | Choi et al., 2016a^104^  Sequential NSDUH data 2015–17 | Suicidal behaviour or ideation | Past-year serious suicidal thoughts | Past-year medical cannabis use vs no use of cannabis or other illicit drugs | Multivariable | Neither those who used nor those who did not use cannabis used other illicit drugs | Positive association |
|  |  |  |  |  |  | Those who used cannabis also used other illicit drugs (but not those who did not use cannabis) | Significant positive association |
|  |  |  |  | Past-year medical cannabis use vs other illicit drug use | Multivariable | Those who used cannabis did not use other illicit drugs | Negative association |
|  |  |  |  |  |  | Both those who used and did not use cannabis used other illicit drugs | Positive association |
