# Supplementary material for: Impacts of medical and non-medical cannabis on the health of older adults: Findings from a scoping review of the literature
Source: PLoS One. 2023 Feb 17;18(2):e0281826. doi: 10.1371/journal.pone.0281826 (PMC9937508; doi:10.1371/journal.pone.0281826)
Supplement: S13 Text — (DOCX) [file pone.0281826.s016.docx]

S13 Text: Summary table of cannabis comparisons for older adults who used cannabis

Data from a priori identified cannabis comparisons of interest (i.e., cannabis comparisons made amongst those who used cannabis) as described in our protocol are presented below as a narrative summary, stratified by patient condition, with a detailed table. We sought data related to the effects of types (e.g., medical vs recreational), frequencies, and durations of use; doses; concentrations; modes of consumption; plant characteristics, and co-use of other substances. Findings from studies reporting this information included the following (stratified by patient condition):

**Older general public:**

- ***Types of use:*** There was no significant difference between any medical use and recreational-only cannabis use regarding the presence of either marijuana abuse or dependence per DSM-IV criteria^4^. However, compared to non-medical use, medical use overseen by a physician was significantly associated with the occurrence of mental health disorders, increased numbers of chronic medical conditions, injuries, and substituting cannabis for a prescription drug^106^, although there were no significant differences in the groups regarding past-year marijuana use disorder, and ED visits with hospital admission^106^. Amongst those who used medical cannabis, there as no significant difference in past-year opioid use between those who used for mixed purposes and those who used strictly for medical purposes^109^.
- ***Doses:*** The number of joints usually smoked in a day in the past 12 months was not significantly associated with past-year ED visits or past-year injuries^106^; however, a higher number of joints smoked when using the most was significantly associated with a greater risk of lifetime occurrence of any substance use disorder, including CUD^105^, but not with lifetime occurrence of any mental health disorders^105^. Higher estimates of THC consumption in the previous 90 days were associated with significantly harmful effects on regional brain volume measures^152^.
- ***Frequency of use:*** High frequency of use in the past year was associated with a significantly higher risk of CUD^4^; lifetime occurrence of any substance use disorders, including CUD^105^; impairments in cognitive functioning^104^, executive functioning^104^, and concentration^99^; past-year serious suicidal thoughts^104^; and measures of regional brain volume^152^. However, there was no effect on past-year ED visits^106^, injuries^106^, depression^104^, lifetime occurrence of any mental health disorder^105^, or total brain volume^152^.
- ***Duration of use:*** Continuous use for the previous 24 months was associated with a significantly higher risk of driving under the influence of marijuana, compared to those who used cannabis in the past year but who did not use in the prior 13–24 months^4^. As the estimated years of use increased, measures of cognitive functioning declined, although not significantly^152^; several measures of regional brain volume significantly decreased^152^. And compared to lifetime use, short-term use demonstrated significantly better measures of cognitive function, but there were no significant differences between short-term use and lifetime use with respect to measures of anxiety, depression, alcohol use, CUD, or any measures of regional or total brain volume^152^.
- ***Use of other substances:*** When older adults who used medical cannabis were compared to those who used both medical cannabis and other illicit drugs, a significantly beneficial association with serious suicidal thoughts in adjusted analyses (i.e., when other illicit drugs were used in addition to medical cannabis, those who used cannabis were more likely to consider suicide than if they didn’t use other illicit drugs).

**End-stage cancer:**

- ***Doses:*** There were no significant differences in the proportion of advanced lung, renal, or skin cancer patients responding to nivolumab, regardless if they were taking 20 g/month or ≥30 g/month of medical cannabis of varying THC:CBD ratios^151^.
- ***Concentrations:*** The concentration of CBD (≥ 1% vs < 1%) in the medical cannabis product consumed had no association with the proportion of advanced lung, renal, or skin cancer patients responding to nivolumab^151^.
- ***Modes of consumption:*** The mode of consumption (smoking/inhalation, oil, combined smoking/inhalation + oil) was not associated with the proportion of patients with advanced lung, renal, or skin cancer that responded to nivolumab^151^.

**Parkinson’s disease**

- ***Frequency of use:*** In those with Parkinson’s disease who used cannabis to self-medicate, at least once a day use was significantly associated with improved dyskinesia compared to less than once a day use^153^.
- ***Plant characteristics:*** There were no differences in dyskinesia in those with Parkinson’s disease who used cannabis to self-medicate, whether they used flowers vs leaves or whether they used fresh vs dried plant^153^.

The table below is stratified by the cannabis comparisons of interest (i.e., types, frequencies, and durations of use; doses; concentrations; modes of consumption; and plant characteristics). The column of cannabis comparisons reported in the studies has been highlighted in grey. Effects reported in the studies are presented in the right-most column, with color coding used to reflect the direction of effect (i.e., green for beneficial and red for harmful effects, grey for no effect) and significance (i.e., dark for significant and light for non-significant effects).

Summary of findings for cannabis comparisons of interest amongst older adults who used cannabis

| Cannabis comparison category | Patient condition | Study | Cannabis comparison | Cannabis product | Outcome category | Outcome definition | Subgroup (if applicable) | Analysis type | Direction of effect and significance |
| --- | --- | --- | --- | --- | --- | --- | --- | --- | --- |
| **Types of use** | Older general public | Choi et al., 2021a^4^  Sequential NSDUH 2015–18 | Any medical vs recreational only | NA | Cannabis use disorder (CUD) | Either marijuana abuse or dependence per DSM-IV | NA | Multivariable | Positive association |
|  |  | Choi et al., 2018^106^  Cross-sectional NESARC-III 2012-13 | Medical (with a medical recommendation status) vs non-medical | NA | CUD | Past-year marijuana use disorder | NA | Univariable | Positive association |
|  |  |  |  |  | ED visits/ admissions | Past-year ED visit and hospital admission | NA | Univariable | Non-significant effect |
|  |  |  |  |  | Mental health disorder | Occurrence of mental disorders | NA | Univariable | Significant positive association |
|  |  |  |  |  | Physical health | Number of chronic medical conditions | NA | Univariable | Significant positive association |
|  |  |  |  |  | Physical injury | Any past-year injury that caused the person to seek medical help or cut down usual activities for > half a day | NA | Univariable | Significant positive association |
|  |  | Corroon et al., 2017^108^  Cross-sectional (study-specific data) | Medical (overseen by a physician or self-medicated) vs non-medical | NA | Problematic prescription drug use | Substituting cannabis for a prescription drug | 51–65 years | Univariable | Significant positive association |
|  | Medical cannabis use | Croker et al., 2021^109^  Cross-sectional (study-specific survey) | Mixed-purpose use vs medical use | NA | Opioid use | Past-year opioid use | NA | Univariable | Positive association |
| **Doses** | End-stage cancer | Taha et al., 2019^151^  Retrospective cohort | 20 g/month vs ≥ 30 g/month | Medical cannabis of varying THC:CBD ratios, supplied from six different companies in Israel | Response to treatment of another therapy | Percent of patients with complete or partial response to nivolumab for advanced lung cancer, renal carcinoma, or malignant melanoma | NA | Univariable | Non-significant effect |
|  | Cannabis use in the older general public | Choi et al., 2018^106^  Cross-sectional NESARC-III 2012-13 | Number of joints usually smoked in a day in the past 12 months | NA | ED visits/ admissions | Past-year ED visit | NA | Multivariable | Negative association |
|  |  |  |  |  | Physical injury | Past-year any injury that caused the person to seek medical help or cut down usual activities for > half a day | NA | Multivariable | Positive association |
|  |  | Choi et al., 2016b^105^  Cross-sectional NESARC-III 2012-13 | Number of joints when using the most | NA | Illicit drug use | Lifetime occurrence of any drug use disorders, including marijuana and other drug use disorders | NA | Multivariable | Significant positive association |
|  |  |  |  |  | Mental health disorder | Lifetime occurrence of any mental disorders, including major depressive disorder, anxiety disorder, PTSD, and bipolar 1 disorder and/or manic or hypomanic episodes | NA | Multivariable | Positive association |
|  | Healthy older adults who used recreational cannabis | Thayer et al., 2018^152^  Cross-sectional | Estimated total THC consumption in the previous 90 days | NA | Regional brain volume | Left caudal middle frontal cortical volume (Surface-based morphometry; ≥ 100 voxels corrected p < 0.05; whole brain general linear models) | NA | Multivariable | Significant negative association |
|  |  |  |  |  |  | Left lateral occipital cortical thickness (Surface-based morphometry; ≥ 100 voxels; uncorrected p < 0.001; whole brain general linear models) | NA | Multivariable | Significant negative association |
|  |  |  |  |  |  | Left occipital fusiform gyrus volume (Voxel-based morphometry; ≥ 100 voxels; uncorrected p < 0.001; whole brain general linear models) | NA | Multivariable | Significant negative association |
|  |  |  |  |  |  | Left superior frontal gyrus volume (Voxel-based morphometry; ≥ 100 voxels; uncorrected p < 0.001; whole brain general linear models) | NA | Multivariable | Significant negative association |
|  |  |  |  |  |  | Left supramarginal gyrus volume (Voxel-based morphometry; ≥ 100 voxels; uncorrected p < 0.001; whole brain general linear models) | NA | Multivariable | Significant negative association |
|  |  |  |  |  |  | Right accumbens volume (Voxel-based morphometry automated segmentations) | NA | Multivariable | Significant negative association |
|  |  |  |  |  |  | Right inferior temporal gyrus volume (Voxel-based morphometry; ≥ 100 voxels; uncorrected p < 0.001; whole brain general linear models) | NA | Multivariable | Significant negative association |
|  |  |  |  |  |  | Right precentral gyrus volume (Voxel-based morphometry; ≥ 100 voxels; uncorrected p < 0.001; whole brain general linear models) | NA | Multivariable | Significant negative association |
|  |  |  |  |  |  | Right superior temporal cortical volume (Surface-based morphometry; ≥ 100 voxels corrected p < 0.05; whole brain general linear models) | NA | Multivariable | Significant negative association |
|  |  |  |  |  |  | Right thalamus volume (Voxel-based morphometry; ≥ 100 voxels; uncorrected p < 0.001; whole brain general linear models) | NA | Multivariable | Significant but unclear effect (increased volume) |
|  |  |  |  |  |  | Total grey matter volume (both Voxel- and Surface-based morphometry automated segmentations) | NA | Multivariable | Significant negative association |
| **Concentrations** | End-stage cancer | Taha et al., 2019^151^  Retrospective cohort | ≥ 1% CBD vs < 1% CBD | Medical cannabis of varying THC:CBD ratios, supplied from six different companies in Israel | Response to treatment of another therapy | Percent of patients with complete or partial response to nivolumab for advanced lung cancer, renal carcinoma, or malignant melanoma | NA | Univariable | Harmful |
|  |  |  | ≥ 10% THC vs < 10% THC | Medical cannabis of varying THC:CBD ratios, supplied from six different companies in Israel | Response to treatment of another therapy | Percent of patients with complete or partial response to nivolumab for advanced lung cancer, renal carcinoma, or malignant melanoma | NA | Univariable | Beneficial |
| **Frequency of use** | Parkinson’s disease | Venderova et al., 2004^153^  Cross-sectional (study specific data) | At least once a day vs less than once daily | Self-medicated with whole Sativa plant or extracts | Dyskinesia | Dyskinesia improvement | NA | Univariable | Significant negative association |
|  | Cannabis use in the older general public | Choi et al., 2021a^4^  Sequential NSDUH 2015–18 | Past-year high (100–365 days of use) vs low (1–99 days of use) frequency | NA | CUD | Past-year either marijuana abuse or dependence per DSM-IV | NA | Univariable | Significant positive association |
|  |  | Benitez et al., 2020^99^  Cross-sectional NESARC-III 2012–13 | Overall effect comparing 1–11 times per year vs 1–3 times per month vs 1–4 times per week vs every or nearly every day | NA | Cognitive functioning | Total scale of Executive Function Index (EFI) | NA | Multivariable | Significant negative association |
|  |  |  |  |  | Executive functioning | Executive function subscale of the EFI | NA | Multivariable | Significant negative association |
|  |  |  |  |  | Concentration/ focus | Attention subscale of the EFI | NA | Multivariable | Significant negative association |
|  |  | Choi et al., 2018^106^  Cross-sectional NESARC-III 2012-13 | Overall effect comparing once in the past year vs twice in the past year vs 3–6 times in the past year vs 7–11 times in the past year vs once a month vs 2–3 times a month vs 1–2 times a week vs 3–4 times a week vs nearly every day vs every day | NA | ED visits/ admission | Past-year ED visit | NA | Multivariable | Non-significant (effect direction not reported) |
|  |  |  |  |  | Physical injury | Any past-year injury that caused the person to seek medical help or cut down usual activities for > half a day | NA | Multivariable | Non-significant (effect direction not reported) |
|  |  | Choi et al., 2016a^104^  Sequential NSDUH 2008–12 | Number of weeks used marijuana in the past year | NA | Depression | Past-year self-reported major depressive episode per DSM-IV | NA | Multivariable | Negative association |
|  |  |  |  |  | Suicidal behaviour or ideation | Past-year serious suicidal thoughts (“At any time in the past 12 months… including today, did you seriously think about trying to kill yourself?”) | NA | Multivariable | Significant positive association |
|  |  | Choi et al., 2016b^105^  Cross-sectional NESARC-III 2012–13 | Currently using more than once a month vs once a month of less | NA | Substance use disorder | Lifetime occurrence of any drug use disorders, including marijuana and other drug use disorders | NA | Multivariable | Significant positive association |
|  |  |  |  |  | Mental health disorder | Lifetime occurrence of any mental disorders, including major depressive disorder, anxiety disorder, PTSD, bipolar 1 disorder and/or manic or hypomanic episodes | NA | Multivariable | Negative association |
|  | Healthy older adults who use recreational | Thayer et al., 2018^152^  Cross-sectional | Number of days used in the past 90 days | NA | Regional brain volume | All subcortical volumes (Voxel-based morphometry automated segmentations) | NA | Multivariable | Non-significant (effect direction not reported) |
|  |  |  |  |  |  | Left caudal middle frontal cortical volume (Surface-based morphometry; ≥ 100 voxels; uncorrected p < 0.001; whole brain general linear models) | NA | Multivariable | Significant negative association |
|  |  |  |  |  |  | Right cerebellum crus I volume (Voxel-based morphometry; ≥ 100 voxels; corrected p < 0.05; whole brain general linear models) | NA | Multivariable | Significant negative association |
|  |  |  |  |  |  | Right occipital fusiform gyrus volume (Voxel-based morphometry; ≥ 100 voxels; corrected p < 0.05; whole brain general linear models) | NA | Multivariable | Significant negative association |
|  |  |  |  |  | Total brain volume | Global structural volume (Voxel-based morphometry automated segmentations) | NA | Multivariable | Non-significant (effect direction not reported) |
| **Duration of use** | Cannabis use in the older general public | Choi et al., 2021a^4^  Sequential NSDUH 2015–18 | Continuous use for 24 months vs initiation/re-initiation (i.e., no use 13–24 months ago, but past-year use) | NA | CUD | Past-year either marijuana abuse or dependence per DSM-IV | NA | Univariable | Positive association |
|  |  |  |  |  | Risky behaviour | Driving under the influence of marijuana | NA | Univariable | Significant positive association |
|  | Healthy older adults who use recreational | Thayer et al., 2018^152^  Cross-sectional | Estimated years of use | NA | Regional brain volume | All subcortical volumes (Voxel-based morphometry automated segmentations) | NA | Multivariable | Non-significant (effect direction not reported) |
|  |  |  |  |  |  | Left frontal orbital cortex volume (Voxel-based morphometry; ≥ 100 voxels; uncorrected p < 0.001; whole brain general linear models) | NA | Multivariable | Significant negative association |
|  |  |  |  |  |  | Right lateral occipital cortex volume (Voxel-based morphometry; ≥ 100 voxels; uncorrected p < 0.001; whole brain general linear models) | NA | Multivariable | Significant negative association |
|  |  |  |  |  |  | Global structural volume (Voxel-based morphometry automated segmentations) | NA | Multivariable | Non-significant (effect direction not reported) |
|  |  |  | Short-term vs lifetime use | NA | Anxiety | Beck Anxiety Inventory total score | NA | Univariable | Negative association |
|  |  |  |  |  | Depression | Beck Depression Inventory, Second Edition (BID-II) total score | NA | Univariable | Negative association |
|  |  |  |  |  | Cognitive functioning | Dimensional Change Card Sort score | NA | Univariable | Negative association |
|  |  |  |  |  |  | Flanker Inhibitory Control score | NA | Univariable | Negative association |
|  |  |  |  |  |  | List Sorting Working Memory score | NA | Univariable | Negative association |
|  |  |  |  |  |  | Oral Reading score | NA | Univariable | Negative association |
|  |  |  |  |  |  | Pattern Comparison Processing Speed test | NA | Univariable | Negative association |
|  |  |  |  |  |  | Picture Sequence Memory score | NA | Univariable | Negative association |
|  |  |  |  |  |  | Picture Vocabulary score | NA | Univariable | Negative association |
|  |  |  |  |  |  | Total Composite score | NA | Univariable | Negative association |
|  |  |  |  |  | Alcohol use | Alcohol Use Disorder Identification Test (AUDIT) total score | NA | Univariable | Negative association |
|  |  |  |  |  |  | Timeline follow-back, alcohol-use days | NA | Univariable | Positive association |
|  |  |  |  |  |  | Timeline follow-back, drinks/drinking day | NA | Univariable | Negative association |
|  |  |  |  |  |  | Timeline follow-back, total drinks | NA | Univariable | Negative association |
|  |  |  |  |  | CUD | Marijuana dependence scale | NA | Univariable | Positive association |
|  |  |  |  |  | Regional brain volume | All regional volumes (Surface-based morphometry, ≥ 100 voxels; uncorrected p<0.001; whole brain general linear model) | NA | Multivariable | Non-significant (effect direction not reported) |
|  |  |  |  |  |  | Left occipital pole (Voxel-based morphometry, ≥ 100 voxels; uncorrected p<0.001; whole brain general linear model) | NA | Multivariable | Lifetime use was significant negative association |
|  |  |  |  |  |  | Left postcentral gyrus (Voxel-based morphometry, ≥ 100 voxels; uncorrected p<0.001; whole brain general linear model) | NA | Multivariable | Lifetime use was significant negative association |
|  |  |  |  |  |  | Right occipital pole (Voxel-based morphometry, ≥ 100 voxels; uncorrected p<0.001; whole brain general linear model) | NA | Multivariable | Lifetime use was significant negative association |
|  |  |  |  |  |  | Right superior parietal lobule; postcentral gyrus (Voxel-based morphometry, ≥ 100 voxels; uncorrected p<0.001; whole brain general linear model) | NA | Multivariable | Lifetime use was significant negative association |
|  |  |  |  |  |  | All subcortical volumes; surface-based morphometry automated segmentation | NA | Univariable | Non-significant (effect direction not reported) |
|  |  |  |  |  |  | All subcortical volumes; Voxel-based morphometry automated segmentation | NA | Univariable | Non-significant (effect direction not reported) |
|  |  |  |  |  | Total brain volume | Global structural volume; surface-based morphometry automated segmentation | NA | Univariable | Non-significant (effect direction not reported) |
|  |  |  |  |  |  | Global structural volume; Voxel-based morphometry automated segmentation | NA | Univariable | Non-significant (effect direction not reported) |
| **Modes of consumption** | End-stage cancer | Taha et al., 2019^151^  Retrospective cohort | Overall effect comparing smoked/ inhaled vs oil vs combined smoked + oil | Medical cannabis of varying THC:CBD ratios, supplied from six different companies in Israel | Response to treatment of another therapy | Percent of patients with complete or partial response to nivolumab for advanced lung cancer, renal carcinoma, or malignant melanoma | NA | Univariable | Non-significant (effect direction not reported) |
| **Plant characteristics** | Parkinson’s disease | Venderova et al., 2004^153^  Cross-sectional (study specific data) | Flowers vs leaves | Self-medicated with whole Sativa plant or extracts | Dyskinesia | Dyskinesia improvement | NA | Univariable | Non-significant (effect direction not reported) |
|  |  |  | Fresh vs dried | Self-medicated with whole Sativa plant or extracts | Dyskinesia | Dyskinesia improvement | NA | Univariable | Non-significant (effect direction not reported) |
| **Use of other substances** | Older general public | Choi et al., 2016a^104^  Sequential NSDUH data 2015–17 | Use vs use + other illicit drug use | Medical cannabis | Suicidal ideation or behaviour | Past-year serious suicidal thoughts | NA | Multivariable | Significant negative association |
